# Synthesis of 3-Alkylideneisoindolin-1-ones via Sonogashira Cyclocarbonylative Reactions of 2-Ethynylbenzamides

**DOI:** 10.1021/acs.joc.0c01282

**Published:** 2020-07-03

**Authors:** Gianluigi Albano, Stefano Giuntini, Laura Antonella Aronica

**Affiliations:** † Dipartimento di Chimica e Chimica Industriale, Università di Pisa, Via Giuseppe Moruzzi 13, 56124 Pisa, Italy; ‡ Dipartimento di Chimica “Ugo Schiff”, Università degli Studi di Firenze, Via della Lastruccia 3, 50019 Sesto Fiorentino, Italy; § Centro di Risonanze Magnetiche (CERM), Università degli Studi di Firenze and Consorzio Interuniversitario Risonanze Magnetiche di Metallo Proteine (CIRMMP), Via Luigi Sacconi 6, 50019 Sesto Fiorentino, Italy

## Abstract

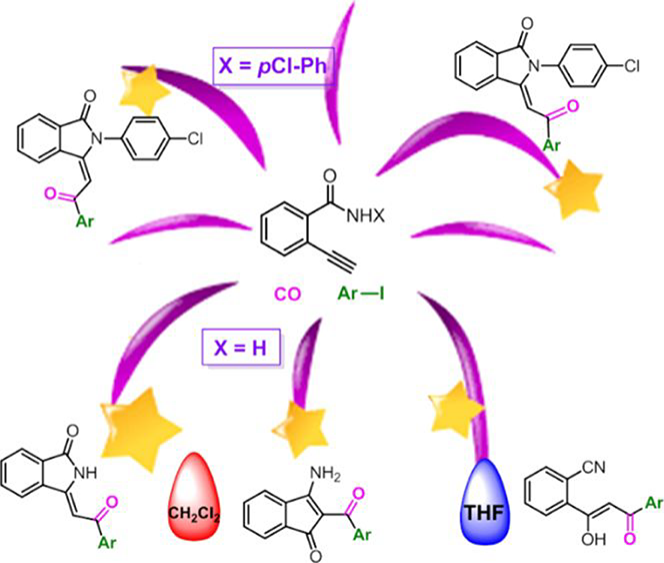

Cyclocarbonylative Sonogashira reactions of *ortho*-ethynylbenzamides have been investigated. The process is carried out under CO pressure, in the presence of a very small amount of PdCl_2_(PPh_3_)_2_ (0.4 mol %) as a catalytic precursor and without the need for a Cu salt as the co-catalyst. 2-Ethynylbenzamide reacted successfully with iodoarenes bearing electron-withdrawing and electron-donating groups, giving rise to different classes of compounds depending on the solvent used. On the contrary, *N*-(4-chlorophenyl)-2-ethynylbenzamide afforded exclusively polyfunctionalized isoindolinones with high stereoselectivity toward (*E*) isomers.

## Introduction

N-containing heterocycles are structural motifs frequently found in a large number of biologically active compounds. For instance, isoindolinone is the core structural unit in several natural products such as chilenine,^1^ lennoxamine,^2^ nuevamine,^3^ chaetosisoindolinone,^4^ stachybotrisan,^5^ erinacerin,^6^ meyeroguilline,^7^ and caputmedusin.^8^ In particular, 3-methyleneisoindolin-1-ones have been recognized as nuclei of natural and synthetic compounds such as fumaridine,^9^ narceine imide,^10^ stigmalactam,^11^ magallinesine,^12^ chartarlactam L,^13^ aristoyagonine,^14^ aristolactams,^15^ and AKS-186.^16^ These heterocycles have been found to possess antimycobacterial^17^ and antifungal^18^ activities and antiplatelet^11,19^ properties, to act as anti-inflammatory^20−22^ and neuroprotective^23^ agents, to inhibit vasoconstriction,^24,25^ and to show cytotoxic and antitumoral activities.^26−30^

Owing to their great importance, there has been a continuous interest in developing metal-promoted cyclization methods for the syntheses of 3-methyleneisoindolin-1-ones.^31^ Transition-metal-catalyzed cyclocarbonylation reaction is a useful approach to the formation of the lactame moiety.^32−41^ Mancuso and co-workers developed an interesting synthesis of 3-methyleneisoindolin-1-ones based on a PdI_2_-catalyzed cyclization of 2-alkynylbenzamides with secondary amines under oxidative carbonylation conditions;^42,43^ Huang^44^ and Hua^45^ proposed cyclocarbonylation of ketimines under CO pressure as a valuable approach to isoindolinones; the Wu’s group^46^ described an elegant procedure based on the cyclization of arylketimine using Mo(CO)_6_ as a CO source and Jiang and co-workers^47^ developed a palladium-catalyzed carbonylation reaction of aromatic oxime for the synthesis of isoindolinone derivatives.

In the last years, our research group has acquired a large experience in the synthesis of heterocyclic compounds via transition-metal-promoted cyclocarbonylative coupling.^48−53^ Because of the large interest of the isoindolinone scaffold, in the present work we explored a new approach for the synthesis of 3-alkylideneisoindolin-1-ones via a copper-free Pd-catalyzed Sonogashira cyclocarbonylative reaction^54−58^ between 2-ethynylbenzamides and various iodoarenes.

## Results and Discussion

**Figure 1:**
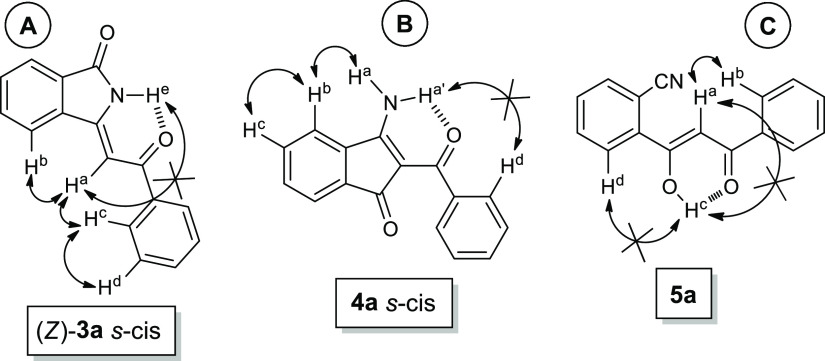
Chemical structure of the products of the cyclocarbonylative Sonogashira reaction between **1** and **2a**: (A) 3-(2-oxo-2-phenylethylidene)isoindolin-1-one **3a**; (B) 3-amino-2-benzoyl-1*H*-inden-1-one **4a**; and (C) (*Z*)-2-(1-hydroxy-3-oxo-3-phenylprop-1-en-1-yl)benzonitrile **5a**.

We started our study with the synthesis of 2-ethynylbenzamide (**1**), which was easily obtained from commercially available 2-bromobenzamide according to a sequence of the Sonogashira reaction with trimethylsilylacetylene followed by desilylation process performed with CsF in MeOH (Scheme S1 in Supporting Information). Then, the first cyclocarbonylative Sonogashira reaction was carried out with equimolar quantities of 2-ethynylbenzamide **1** and iodobenzene **2a**, in a stainless steel autoclave placed under CO pressure (20 atm) using a very low amount of PdCl_2_(PPh_3_) (0.4 mol %), a mixture of CH_2_Cl_2_ and triethylamine for 4 h at 100 °C (Table 1, entry 1). The analysis of the ^1^H NMR spectrum of the crude product showed the partial conversion of precursors and the presence of proton signals that indicated the formation of two different compounds. The first product was the expected 3-(2-oxo-2-phenylethylidene)isoindolin-1-one **3a**, recovered chemically pure with a 34% yield. Its structure was confirmed by spectroscopic (^1^H NMR and ^13^C NMR), spectrometric (LC–MS), and elemental analysis (see Experimental Section). Moreover, NOE (nuclear Overhauser effect) experiments (Figure 1A) highlighted not only a strong dipolar coupling between the vinyl proton H^a^ and the aromatic protons H^b^ and H^c^ but also the absence of interactions of the amide proton H^e^ with other hydrogens. This evidence allowed the attribution of *Z* configuration to **3a** obtained also as a single conformational isomer, the s-cis, probably due to the hydrogen bond between amide proton and carbonyl oxygen (Figure 1A).

**Table 1:**
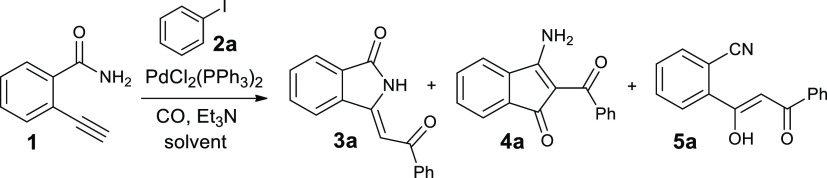
Optimization Study of the Cyclocarbonylative Sonogashira Reaction Between 2-Ethynylbenzamide **1** and Iodobenzene **2a**

					selectivity^c^ (%)		
entry^a^	solvent	*T* (°C)	*t* (h)	conversion^b^ (%)	**3a**	**4a**	**5a**
1	CH_2_Cl_2_	100	4	80	57 (34)	43 (21)	
2	CH_2_Cl_2_	100	8	78	39	61	
3^d^	CH_2_Cl_2_	100	4	69	28	72	
4	CH_2_Cl_2_	70	24	79	29	71	
5	CH_2_Cl_2_	50	24	78	21		79 (42)
6	THF	100	4	94	29		71 (44)
7	THF	50	24	79	21		79
8	THF	30	24	16	26		74
9	CH_3_CN	100	4	85	78		22
10	CH_3_CN	50	24	83	38		62
11	DMF	100	4	100	33	32	35

a All reactions were carried with 2-ethynylbenzamide **1** (1.0 mmol), iodobenzene **2a** (1.0 mmol), CO (20 atm), PdCl_2_(PPh_3_)_2_ (0.4 mol %), Et_3_N (1.5 mL), and the solvent (4.0 mL), unless otherwise stated.

b Conversion was determined by the ^1^H NMR peak integration on the crude product.

c Selectivity was estimated by ^1^H NMR spectroscopy; isolated yields of pure products are reported in parentheses.

d Reaction performed with 1 mol % of PdCl_2_(PPh_3_)_2_.

The second product (21% yield), required a more in-depth structural study. First, the analysis of the ^1^H NMR spectrum highlighted the presence of two broad singlet signals at particularly low fields (10.09 and 10.21) which were attributed to amino protons H^a,a′^ (Figure 1B). Moreover, the ^13^C NMR spectrum indicated the presence of two signals corresponding to two carbonyl carbons (186.73 and 190.22 ppm). Two peaks corresponding to a double bond were also detected: the first at 172.21 ppm was related to a carbon atom linked to the NH_2_ group and the other at 103.02 ppm was due to ≡C–CO. All these data confirmed the formation of 3-amino-2-benzoyl-1*H*-inden-1-one **4a** (Table 1, entry 1). Moreover, NOE experiments conducted on the pure product highlighted a dipolar coupling between the protons H^a^, H^b^, and H^c^ and the absence of couplings between H^c′^ and H^d^ (Figure 1B), thus indicating also for product **4a** a s-cis conformation.

With the aim to increase the conversion and the selectivity toward desired compound **3a**, cyclocarbonylative Sonogashira tests at different reaction times, temperatures, and amounts of the catalytic precursor were performed. As is evident from the results described in Table 1, increasing the reaction time from 4 to 8 h (Table 1, entry 2) or the amount of PdCl_2_(PPh_3_)_2_ (Table 1, entry 3, 1 mol %) did not affect the conversion significantly. On the contrary, an increase in selectivity toward the amino product **4a** was observed (up to ∼70%). A similar result was obtained by conducting the reaction at 70 °C for 24 h (Table 1, entry 4).

**Scheme 1:**
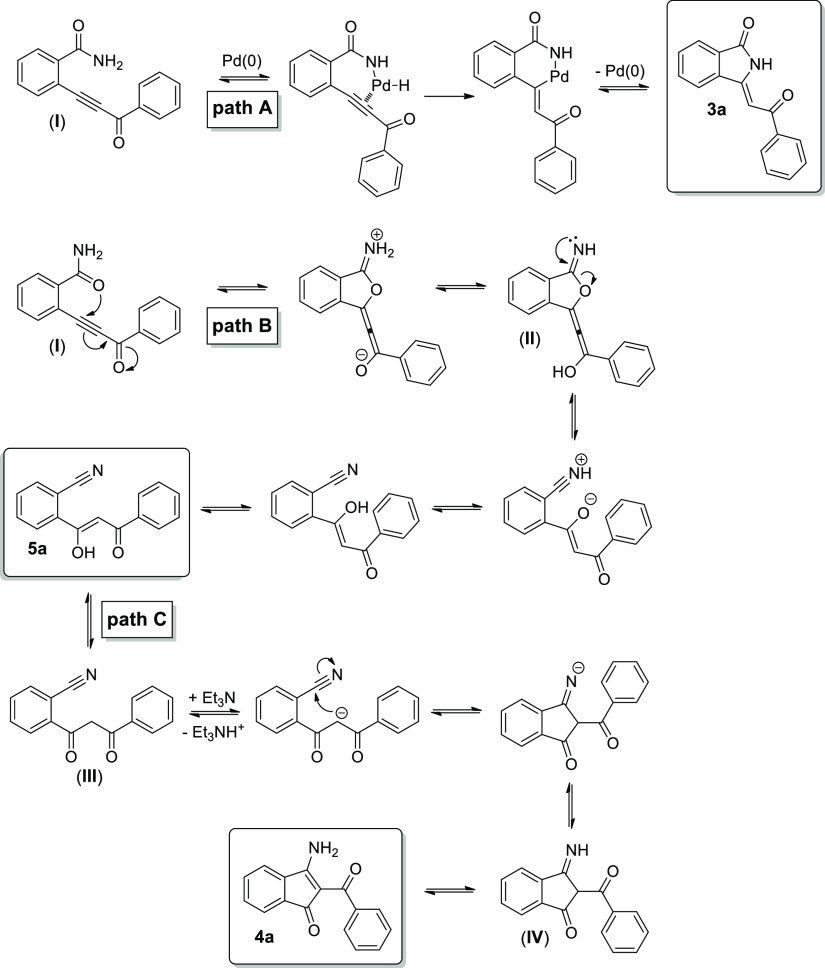
Plausible Mechanism for the Formation of Products **3a**, **4a**, and **5a** via the Sonogashira Cyclocarbonylative Reaction between 2-Ethynylbenzamide **1** and Iodobenzene **2a**

A further reduction in temperature to 50 °C (Table 1, entry 5) gave instead an unexpected result. The analysis of the ^1^H NMR spectrum of the crude product showed, in addition to the presence of isoindolinonic derivative **3a**, the disappearance of the typical signals of **4a** and the appearance of a new olefinic proton signal at 6.95 ppm. After purification, the new compound was subjected to ^1^H NMR, ^13^C NMR, LC–MS, and elemental analyses in order to determine its exact structure, which resulted to be (*Z*)-2-(1-hydroxy-3-oxo-3-phenylprop-1-en-1-yl)benzonitrile **5a** (Figure 1C). In fact, the analysis of the ^1^H NMR spectrum indicated the presence of a signal that resonates at very low fields (16.44 ppm), a characteristic of a 1,3-diketonic system in the enolic form.^59^ Furthermore, in the ^13^C NMR spectrum, olefinic carbon (96.00 ppm), a signal corresponding to C≡N (118.00 ppm) and two peaks at 183.14 and 186.21 ppm (carbonyl and enolic carbon atoms) were clearly observed, thus confirming the structure of **5a** (42% yield of isolated product). The formation of the three products **3a**, **4a,** and **5a** can be tentatively explained by the mechanism described in Scheme 1. First, expected isoindolin-1-one **3a** was generated via the initial formation of the Sonogashira product (**I**), which is in situ cyclized as previously observed (Scheme 1, path A).^48,49^ On the other hand, in the case of **4a**, a process of addition of carbonyl oxygen to the triple bond can be hypothesized with the formation of an allenyl species (**II**). After prototropic exchange and subsequent opening of the cycle, 1,3-diketone **5a** in the enolic form can be generated (Scheme 1, path B). Finally, indenone **4a** can derive directly from **5a**. Indeed, under experimental conditions (*i.e.*, high temperature and excess of Et_3_N), the diketone (**III**) can be deprotonated and the obtained carbanion can attack the −CN functionality forming the cycle. Finally, after protonation (e.g., by Et_3_NH^+^) and subsequent imine–enamine rearrangement of (**IV**), **4a** product is formed.

A confirmation of the above mechanism was given by treating **5a** under the cyclocarbonylative Sonogashira conditions of Table 1, entry 1 (0.4 mol % of PdCl_2_(PPh_3_)_2_, CH_2_Cl_2_, and Et_3_N, 20 atm of CO, 100 °C, for 4 h). Indeed, indenone **4a** was exclusively formed (Scheme S2 in Supporting Information). In order to obtain more information regarding the reactivity of benzamide **1**, further experiments were performed under different experimental conditions.

**Figure 2:**
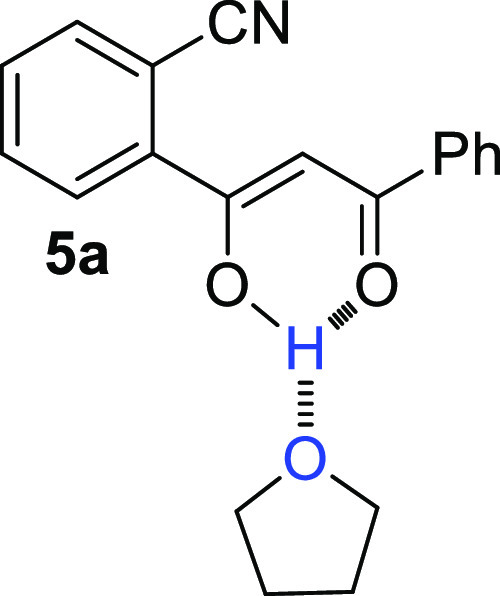
Stabilization of (*Z*)-2-(1-hydroxy-3-oxo-3-phenylprop-1-en-1-yl)benzonitrile (**5a**) by coordination with THF.

As reported in Table 1, the nature of the solvents seemed to influence markedly the chemoselectivity of the reactions. In fact, when CH_2_Cl_2_ is used as the solvent, the chemoselectivity depends on the experimental conditions (Table 1, entries 1–5). On the contrary, tests carried out in tetrahydrofuran (THF) (Table 1, entries 6–8) afforded **5a** with high selectivity (71–79%), regardless of the temperature and duration of the reactions. The particular behavior of THF can be ascribed to a strong coordination effect (hydrogen bond) between ketoenol hydrogen and THF oxygen atoms as depicted in Figure 2.

As far as acetonitrile and dimethylformamide (DMF) are concerned, in the first case the preferential formation of **5a** was observed at high temperatures (Table 1, entry 9), while the use of DMF involved the formation of a mixture of products.

Given the data obtained in the reactions between 2-ethinylbenzamide **1** and iodobenzene **2a**, the extension of Sonogashira cyclocarbonylative reactions to iodoarenes having different steric and electronic requirements was subsequently investigated. The reactions were carried out under the reaction conditions which provided the desired isoindolinone **3a** with better conversion and chemoselectivity, that is operating in dichloromethane and triethylamine, with 0.4 mol % of PdCl_2_(PPh_3_)_2_, 20 atm of CO, at 100 °C. The main results are described in Table 2. In all cases, a mixture of isoindolinone **3** and indenone **4** was obtained. However, the products could be easily separated and isolated chemically pure with satisfactory yields. As reported in Table 2, compared to preliminary reactions conducted with iodobenzene **2a**, the use of *p*-methoxy derivative **2b** showed a similar trend in terms of chemoselectivity and yield. Moreover, increasing the reaction time, an increase in the conversion was observed while the ratio between **3b** and **4b** remained substantially the same (Table 2, entries 2–4). Instead, when the reaction was performed with the more sterically hindered *ortho*-methoxyiodobenzene **2c**, a decrease in the reaction rate was observed even if the reaction was carried out with 1 mol % of PdCl_2_(PPh_3_)_2_ (Table 2, entry 5, 73% conversion). Finally the reaction could be performed successfully also in the case of iodoarenes characterized by the electron-withdrawing groups such as −Cl and −CN (**2d–e**) (Table 2, entries 6–7).

**Table 2:**
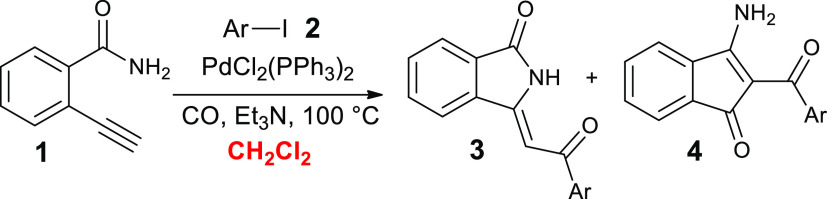
Cyclocarbonylative Sonogashira Reactions of 2-Ethynylbenzamide **1** with Iodoarenes **2** Performed in CH_2_Cl_2_

						selectivity^c^ (%)	
entry^a^	Ar	**2**	*t* (h)	conversion^b^ (%)	**3**, **4**	**3**	**4**
1	Ph	**a**	4	80	**a**	57 (34)	43 (21)
2	4-OMePh	**b**	4	79	**b**	55 (38)	45 (30)
3	4-OMePh	**b**	8	84	**b**	56	44
4	4-OMePh	**b**	24	100	**b**	58	42
5^d^	2-OMePh	**c**	24	73	**c**	50 (37)	50 (29)
6	4-ClPh	**d**	4	90	**d**	58 (32)	42 (37)
7	4-CNPh	**e**	4	86	**e**	36 (15)	64 (44)

a All reactions were carried with 2-ethynylbenzamide **1** (1.0 mmol), iodoarene **2** (1.0 mmol), CO (20 atm), PdCl_2_(PPh_3_)_2_ (0.4 mol %), Et_3_N (1.5 mL), and CH_2_Cl_2_ (4.0 mL) at 100 °C, unless otherwise stated.

b Conversion was determined by ^1^H NMR peak integration on the crude product.

c Selectivity was estimated by ^1^H NMR spectroscopy; isolated yields of pure products are reported in parentheses.

d Reaction performed with 1 mol % of PdCl_2_(PPh_3_)_2_.

Considering the interesting synthetic potentialities of compound **5a** possessing a diketo group^60−66^ in the keto-enolic form, a few cyclocarbonylative Sonogashira reactions between 2-ethynylbenzamide **1** and different iodoarenes **2** were also performed in THF. As reported in Table 3, a high conversion of the reactants (75–100%) was observed and the formation of a mixture of isoindolinone **3** and ketoenol **5** was obtained. Nevertheless, compounds **5a–e** could be easily isolated chemically pure in moderate yields. The composition of crude products depended on the nature of the functional group on iodoarene **2**. Indeed, using 4-iodoanisole **2b**, we obtained almost the same result of iodobenzene **2a** (Table 3, entries 1–2), while in the reaction between **1** and 1-chloro-4-iodobenzene **2c** (Table 3 entry 3) the selectivity changed slightly (40/60).

**Table 3:**
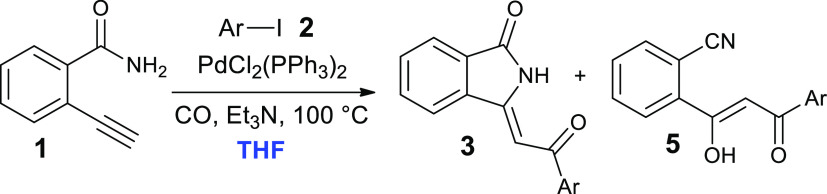
Cyclocarbonylative Sonogashira Reactions of 2-Ethynylbenzamide **1** with Iodoarenes **2** Performed in THF

						selectivity^c^ (%)	
entry^a^	Ar	**2**	*t* (h)	conversion^b^ (%)	**3**, **5**	**3**	**5**
1	Ph	**a**	4	94	**a**	29	71 (44)
2	4-OMePh	**b**	4	75	**b**	29	71 (41)
3	4-ClPh	**d**	4	89	**d**	40	60 (39)
4	4-CNPh	**e**	4	100	**e**	72	28 (22)

a All reactions were carried with 2-ethynylbenzamide **1** (1.0 mmol), iodoarene **2** (1.0 mmol), CO (20 atm), PdCl_2_(PPh_3_)_2_ (0.4 mol %), Et_3_N (1.5 mL) and CH_2_Cl_2_ (4.0 mL) at 100 °C, unless otherwise stated.

b Conversion was determined by ^1^H NMR peak integration on the crude product.

c Selectivity was estimated by ^1^H NMR spectroscopy; isolated yields of pure products are reported in parentheses.

Finally, using 4-iodobenzonitrile **2e** as reactant, there was a clear prevalence of the isoindolinone **3e** (72%) (Table 3, entry 4). These data clearly showed the need for fine-tuning of the cyclocarbonylation process in the event that keto-enols **5** possessing strong electron-withdrawing groups are desired.

The obtained results described so far indicated that the free −NH_2_ group is involved in the formation of different products depending on the experimental conditions used. In order to increase the chemoselectivity toward isoindolinone compounds, *N*-(4-chlorophenyl)-2-ethynylbenzamide **6** was prepared from 2-iodobenzoic acid according to the four-step synthetic procedure depicted in Scheme S3 of Supporting Information.

**Scheme 2:**
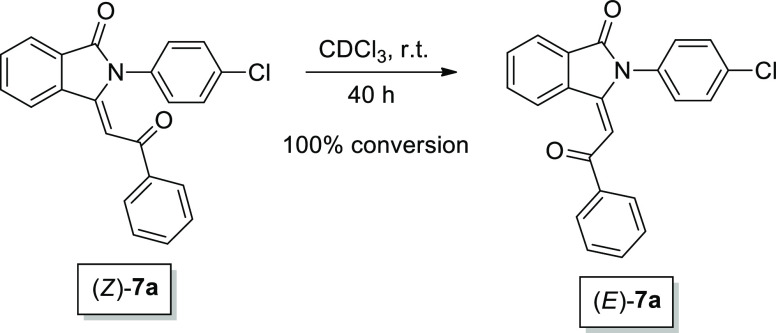
Interconversion of 2-(4-Chlorophenyl)-3-(2-oxo-2-phenylethylidene)isoindolin-1-one **7a** from the (*Z*)-Isomer to the (*E*)-Isomer, Performed in CDCl_3_ at Room Temperature

Initially, *N*-(4-chlorophenyl)-2-ethynylbenzamide **6** was submitted to a Sonogashira cyclocarbonylation reaction with iodobenzene **2a** under experimental conditions which generally favored isoindolinone formation, that is, CH_2_Cl_2_, 100 °C, 20 atm CO, 0.4 mol % of PdCl_2_(PPh_3_)_2_ (Table 4, entry 1). To our delight, the analysis of the crude product indicated the complete conversion of starting materials and the formation of two isomers which were identified as (*Z*)- and (*E*)-2-(4-chlorophenyl)-3-(2-oxo-2-phenylethylidene)isoindolin-1-one **7a**. Moreover the synthesis was highly stereoselective, that is, with a (*E*)-**7a**/(*Z*)-**7a** molar ratio of 89/11, probably due to the lower steric hindrance of the (*E*)-isomer. Indeed, when a sample of (*Z*)-**7a** in CDCl_3_ was maintained for 40 h at room temperature, its complete conversion into (*E*)-isomer was observed (Scheme 2). As previously observed,^48^ the presence of acid traces in chloroform could cause the interconversion to occur.

**Table 4:**
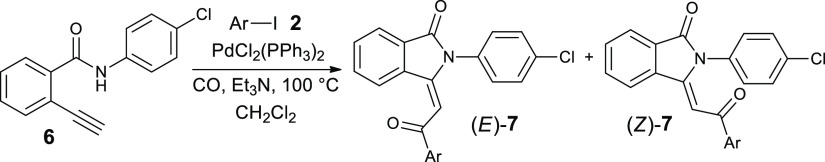
Cyclocarbonylative Sonogashira Reactions of *N*-(4-Chlorophenyl)-2-ethynylbenzamide **6** with Iodoarenes **2**

						selectivity^c^ (%)	
entry^a^	Ar	**2**	*t* (h)	conversion^b^ (%)	**7**	(*E*)-**7**	(Z)-**7**
1	Ph	**a**	4	100	**a**	89 (66)	11 (6)
2^d^	Ph	**a**	24	84	**a**	90	10
3	4-OMePh	**b**	4	100	**b**	88 (69)	12 (10)
4	2-OMePh	**c**	4	100	**c**	93 (74)	7 (3)
5	4-ClPh	**d**	4	100	**d**	84^f^ (51)	10^f^ (7)
6^e^	4-ClPh	**d**	4	100	**d**	86^f^	10^f^
7	1-Napht	**f**	4	100	**f**	89 (69)	11 (5)
8	4-MePh	**g**	4	100	**g**	90 (67)	10 (7)
9	2-MePh	**h**	4	100	**h**	88 (60)	12 (5)

a All reactions were carried with *N*-(4-chlorophenyl)-2-ethynylbenzamide **6** (1.0 mmol), iodoarene **2** (1.0 mmol), CO (20 atm), PdCl_2_(PPh_3_)_2_ (0.4 mol %), Et_3_N (1.5 mL), and CH_2_Cl_2_ (4.0 mL) at 100 °C, unless otherwise stated.

b Conversion was determined by ^1^H NMR peak integration on the crude product.

c Selectivity was estimated by ^1^H NMR spectroscopy; isolated yields of pure products are reported in parentheses.

d Reaction performed at 50 °C.

e Reaction performed under 40 atm of CO.

f The remainder of the product was (*Z*)-3-(4-chlorobenzylidene)-2-(4-chlorophenyl)isoindolin-1-one **8**.

When the cyclocarbonylative reaction of amide **6** with iodobenzene **2a** was carried out for a longer reaction time (24 h) but at 50 °C, a reduction of conversion was observed (84%) while stereoselectivity resulted in almost the same (Table 4, entry 2, (*E*)-**7a**/(*Z*)-**7a** molar ratio 90/10). Therefore, all subsequent reactions were carried out at 100 °C for 4 h (Table 4, entries 3–9). In all cases, a quantitative conversion of reagents was observed and generally a mixture of two *E*/*Z* isomers (ca. 90/10) was obtained. Both compounds could be easily separated and isolated chemically pure by neutral alumina column chromatography.

**Scheme 3:**
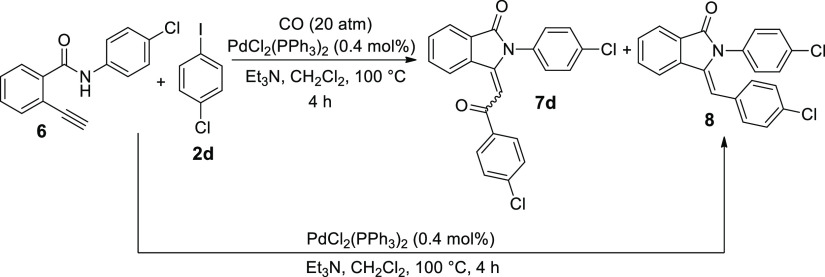
Cyclocarbonylative Sonogashira and Cyclic Sonogashira Reactions of Amide **6** with 1-Chloro-4-iodobenzene **2d**

The reactions performed between amide **6** and iodoarenes possessing electron-donating groups (*i.e.*, **2b–c** and **2f–h**) (Table 4, entries 3–4 and 7–9) afforded the (*E*)-isomers as principle products in good yields (60–74%). In the case of cross-coupling with 1-chloro-4-iodobenzene **2d** (Table 4, entry 5), a small amount of (*Z*)-3-(4-chlorobenzylidene)-2-(4-chlorophenyl)isoindolin-1-one **8** was obtained. Its structure has been assigned by comparison with a pure sample prepared via the cyclic Sonogashira reaction as depicted in Scheme 3. The product composition did not change even performing the cyclocarbonylative reaction of amide **6** with 1-chloro-4-iodobenzene **2d** under 40 atm of carbon monoxide pressure (Table 4, entry 6).

A cyclocarbonylative Sonogashira reaction of *N*-(4-chlorophenyl)-2-ethynylbenzamide **6** was also carried out in the presence of the electron-poor 4-iodobenzonitrile **2e**: in this case, only small amounts of (*E*)-4-(2-(2-(4-chlorophenyl)-3-oxoisoindolin-1-ylidene)acetyl)benzonitrile (*E*)-**7e** (yield 18%) and (*Z*)-4-(2-(2-(4-chlorophenyl)-3-oxoisoindolin-1-ylidene)acetyl)benzonitrile (*Z*)-**7e** (yield 5%), together with (*Z*)-4-((2-(4-chlorophenyl)-3-oxoisoindolin-1-ylidene)methyl)benzonitrile **9** (yield 4%) were isolated (Scheme S4 in Supporting Information).

**Scheme 4:**
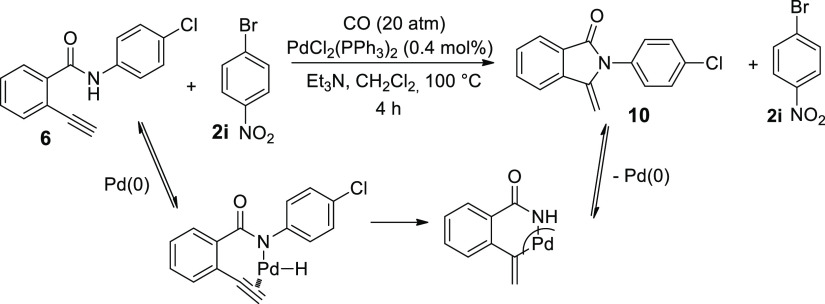
Cyclocarbonylative Sonogashira and Cyclic Sonogashira Reactions of Amide **6** with 1-Chloro-4-iodobenzene **2d**

Finally, considering the general toxicity of iodoarenes, a test between 4-bromonitrobenzene **2i** and ethynylbenzamide **6** was performed under 20 atm of CO, at 100 °C for 4 h (Scheme 4). Unfortunately, bromoderivative **2i** was recovered unreacted, while benzamide **6** was completely consumed. After purification of the crude mixture, 2-(4-chlorophenyl)-3-methyleneisoindolin-1-one (**10**)^45^ was isolated in an 85% yield. The formation of cyclisation product **10** could be explained considering the insertion of Pd(0) into the N–H bond, Pd-hydride addition to the triple bond followed by reductive elimination with the generation of methyleneisoindolinone **10** and Pd(0).

## Conclusions

In conclusion, we have developed an atom-efficient approach to alkylidene isoindolin-1-ones through a Pd-catalyzed copper-free cyclocarbonylative Sonogashira reaction between benzamides and aryl iodides. In particular, when 2-ethynylbenzamide **1** was used in CH_2_Cl_2_, the reaction generally afforded (*Z*)-isoindolinones in a major amount together with indenone derivatives. Changing the solvent to THF determined the preferential formation of keto-enol compounds. On the other hand, the Sonogashira cyclocarbonylative reaction of *N*-(4-chlorophenyl)-2-ethynylbenzamide **6** with iodoarenes generated almost exclusively the corresponding (*E*)-isoindolinones in satisfactory yields.

## Experimental Section

### General Information

Solvents were purified by conventional methods, distilled, and stored over activated molecular sieves under argon. All the chemicals were purchased from commercial sources and used as received without purification. All the operations under an inert atmosphere were carried out using standard Schlenk techniques and employing dried nitrogen. Reactions that required heating were performed in an oil bath. For all reactions, conversion was monitored by thin-layer chromatography analysis on pre-coated silica gel plates (VWR Macherey-Nagel, 0.2 mm thick) or pre-coated neutral alumina plates (Sigma-Aldrich, 0.25 mm thick). Column chromatography was performed with Fluka silica gel, pore size 60 c5, 70–230 mesh, 63–200 μm or Sigma-Aldrich activated, neutral alumina. ^1^H NMR and ^13^C NMR spectra were recorded at room temperature in CDCl_3_ or DMSO-*d*_6_ solution with a Varian INOVA-600 spectrometer, operating at a frequency of 600 MHz for ^1^H and 150 MHz for ^13^C, using the residual solvent peak as the internal reference; chemical shift (δ) values are given in parts per million (ppm) and coupling constants (*J*) in Hz. Mass spectra were obtained with an Applied Biosystems-MDS Sciex API 4000 triple quadrupole mass spectrometer (Concord, Ont., Canada), equipped with a Turbo-V ion-spray (TIS) source. Elemental analyses were performed on a Elementar Vario Micro Cube CHN-analyzer.

### Synthesis of Ethynylbenzamides

#### 2-((Trimethylsilyl)ethynyl)benzamide (**B**)^67^

2-Bromobenzamide (**A**) (7.00 g, 35.0 mmol), PdCl_2_(PPh_3_)_2_ (983 mg, 1.40 mmol), CuI (267 mg, 1.40 mmol), Et_3_N (160 mL), and DMF (50 mL) were mixed together, then trimethylsilylacetylene (7.4 mL, 52.5 mmol) was added dropwise. The resulting mixture was refluxed under stirring for 24 h, then it was cooled to room temperature, hydrolyzed with saturated ammonium chloride solution (150 mL) and extracted with CH_2_Cl_2_ (3 × 150 mL). The combined organic phases were washed with brine (150 mL), dried over anhydrous Na_2_SO_4_, and the solvent was removed under vacuum. The crude product was purified by column chromatography (SiO_2_, *n*-hexane/AcOEt 1:1) to give 3.18 g (yield 42%) of 2-((trimethylsilyl)ethynyl)benzamide (**B**).^67^

^1^H NMR (600 MHz, CDCl_3_) δ (ppm): 0.28 (9H, s), 6.04 (1H, br s), 7.43–7.46 (2H, m), 7.55–7.56 (1H, m), 7.76 (1H, br s), 8.13–8.17 (1H, m). ^13^C NMR (150 MHz, CDCl_3_) δ (ppm): −0.2 (3C), 102.3, 104.4, 120.1, 129.3, 130.6, 131.1, 134.1, 134.7, 168.2. LC–MS (APCI^+^) *m*/*z*: 218.1 [M + H]^+^.

#### 2-Ethynylbenzamide (**1**)^67^

2-((Trimethylsilyl)ethynyl)benzamide (**B**) (3.18 g, 14.6 mmol), cesium fluoride (3.33 g, 21.9 mmol), and methanol (100 mL) were mixed together. The resulting mixture was left under stirring for 3 h at room temperature, then it was hydrolyzed with brine (100 mL) and extracted with AcOEt (3 × 50 mL). The combined organic phases were washed with brine (100 mL), dried over anhydrous Na_2_SO_4_, and the solvent was removed under vacuum. The crude product was purified by column chromatography (SiO_2_, *n*-hexane/AcOEt 1:1) to give 1.51 g (yield 71%) of 2-ethynylbenzamide (**1**).^67^

^1^H NMR (600 MHz, CDCl_3_) δ (ppm): 3.52 (1H, s), 6.10 (1H, br s), 7.34 (1H, br s), 7.45–7.49 (2H, m), 7.59–7.61 (1H, m), 8.08–8.09 (1H, m). ^13^C NMR (150 MHz, CDCl_3_) δ (ppm): 82.5, 84.2, 119.1, 129.6, 130.3, 131.2, 134.5, 135.5, 168.3. LC–MS (APCI^+^) *m*/*z*: 146.1 [M + H]^+^.

#### 2-Iodobenzoyl chloride (**D**)^68^

2-Iodobenzoic acid (**C**) (4.97 g, 20.0 mmol), DMF (46 μL, 0.6 mmol), and CH_2_Cl_2_ (50 mL) were mixed together, then oxalyl chloride (3.5 mL, 40.8 mmol) was added dropwise to the solution at 0 °C. The mixture was left under stirring for 2 h at room temperature, then it was evaporated under vacuum to give 2-iodobenzoyl chloride (**D**)^68^ (4.66 g, yield 87%), which was used without further purification.

^1^H NMR (600 MHz, CDCl_3_) δ (ppm): 7.23–7.26 (1H, m), 7.49–7.51 (1H, m), 8.04–8.05 (1H, m), 8.07–8.08 (1H, m). ^13^C NMR (150 MHz, CDCl_3_) δ (ppm): 94.1, 128.2, 133.9, 134.3, 138.2, 141.7, 166.8.

#### *N*-(4-Chlorophenyl)-2-iodobenzamide (**E**)^69^

4-Chloroaniline (3.19 g, 25.0 mmol), Et_3_N (3.5 mL, 25.0 mmol), and CH_2_Cl_2_ (25 mL) were mixed together, then a solution of 2-iodobenzoyl chloride (**D**) (6.67 g, 25.0 mmol) in CH_2_Cl_2_ (25 mL) was added dropwise to the solution at 0 °C. The mixture was left under stirring for 90 min at room temperature, then it was hydrolyzed with water (100 mL) and extracted with CH_2_Cl_2_ (3 × 50 mL). The combined organic phases were washed, in order, with HCl 1 M solution (100 mL), water (100 mL), saturated NaHCO_3_ solution (100 mL), and brine (100 mL), then dried over anhydrous Na_2_SO_4_, and the solvent was removed under vacuum to give *N*-(4-chlorophenyl)-2-iodobenzamide (**E**)^69^ (7.24 g, yield 81%) which was used without further purification.

^1^H NMR (600 MHz, CDCl_3_) δ (ppm): 7.13–7.15 (1H, m), 7.32 (2H, d, *J* = 8.7 Hz), 7.39–7.42 (1H, m), 7.46–7.48 (1H, m), 7.57 (2H, d, *J* = 8.7 Hz), 7.70 (1H, br s), 7.88–7.89 (1H, m). ^13^C NMR (150 MHz, CDCl_3_) δ (ppm): 92.4, 121.4 (2C), 128.2, 128.4, 129.0 (2C), 129.8, 131.5, 136.1, 139.9, 141.6, 167.4. LC–MS (APCI^+^) *m*/*z*: 358.0 [M + H]^+^.

#### *N*-(4-Chlorophenyl)-2-((trimethylsilyl)ethynyl)benzamide (**F**)^70^

*N*-(4-Chlorophenyl)-2-iodobenzamide (**E**) (6.80 g, 19.0 mmol), PdCl_2_(PPh_3_)_2_ (534 mg, 0.76 mmol), CuI (145 mg, 0.76 mmol), Et_3_N (5.3 mL, 38.0 mmol), and THF (120 mL) were mixed together, then trimethylsilylacetylene (4.1 mL, 28.9 mmol) was added dropwise. The resulting mixture was left under stirring for 24 h at room temperature, then it was hydrolyzed with saturated ammonium chloride solution (100 mL) and extracted with CH_2_Cl_2_ (3 × 100 mL). The combined organic phases were washed with brine (100 mL), dried over anhydrous Na_2_SO_4_, and the solvent was removed under vacuum. The crude product was purified by column chromatography (SiO_2_, *n*-hexane/AcOEt 6:1) to give 4.95 g (yield 79%) of *N*-(4-chlorophenyl)-2-((trimethylsilyl)ethynyl)benzamide (**F**).^70^

^1^H NMR (600 MHz, CDCl_3_) δ (ppm): 0.25 (9H, s), 7.34 (2H, d, *J* = 9.0 Hz), 7.44–7.48 (2H, m), 7.58–7.59 (1H, m), 7.62 (2H, d, *J* = 9.0 Hz), 8.10–8.12 (1H, m), 9.33 (1H, br s). ^13^C NMR (150 MHz, CDCl_3_) δ (ppm): −0.2 (3C), 102.9, 103.0, 119.3, 121.3 (2C), 129.0 (2C), 129.4, 129.5, 130.3, 130.9, 134.1, 135.4, 136.5, 164.0. LC–MS (APCI^+^) *m*/*z*: 328.1 [M + H]^+^.

#### *N*-(4-Chlorophenyl)-2-ethynylbenzamide (**6**)

*N*-(4-Chlorophenyl)-2-((trimethylsilyl)ethynyl)benzamide (**F**) (3.94 g, 12.0 mmol) and methanol (100 mL) were mixed together, then a solution of tetrabutylammonium fluoride trihydrate (4.54 g, 14.4 mmol) in methanol (100 mL) was added dropwise to the solution. The resulting mixture was left under stirring for 1 h at room temperature, then it was hydrolyzed with brine (200 mL), and extracted with AcOEt (3 × 150 mL). The combined organic phases were washed with brine (100 mL), dried over anhydrous Na_2_SO_4_, and the solvent was removed under vacuum. The crude product was purified by column chromatography (SiO_2_, *n*-hexane/AcOEt 4:1) to give 1.78 g (yield 58%) of *N*-(4-chlorophenyl)-2-ethynylbenzamide (**6**).

^1^H NMR (600 MHz, CDCl_3_) δ (ppm): 3.57 (1H, s), 7.27 (2H, d, *J* = 9.0 Hz), 7.39–7.42 (2H, m), 7.54–7.56 (1H, m), 7.59 (2H, d, *J* = 9.0 Hz), 7.91–7.92 (1H, m), 9.10 (1H, br s). ^13^C NMR (150 MHz, CDCl_3_) δ (ppm): 81.9, 84.4, 118.4, 121.2 (2C), 129.0 (2C), 129.5, 129.6, 129.9, 130.9, 134.2, 136.4, 136.5, 164.3. LC–MS (APCI^+^) *m*/*z*: 256.0 [M + H]^+^. Anal. Calcd for C_15_H_10_ClNO: C, 70.46, H, 3.94, N, 5.48. Found: C, 70.39; H, 3.99; N, 5.47.

### Cyclocarbonylative Sonogashira Reactions of 2-Ethynylbenzamide (**1**)

#### General Procedure

A Pyrex Schlenk tube under a CO atmosphere was charged with 2-ethynylbenzamide (**1**) (1.0 mmol), iodoarene (1.0 mmol), Et_3_N (1.5 mL), and the solvent (4.0 mL). This solution was introduced by a steel siphon into a 25 mL stainless steel autoclave, fitted with a Teflon inner crucible, and a stirring bar, previously carried with PdCl_2_(PPh_3_)_2_ (0.4–1.0 mol %) and placed under vacuum (0.1 Torr). The reactor was pressurized with CO (20 atm) and the mixture was stirred for a selected time at a selected temperature. After removal of excess CO (fume hood), the reaction mixture was diluted with CH_2_Cl_2_ (20 mL), washed with brine (15 mL), dried over anhydrous Na_2_SO_4_, and the solvent was removed under vacuum. The reagent conversion and the product composition were determined by the ^1^H NMR spectroscopic analysis. All crude products were purified through column chromatography on silica gel and characterized with ^1^H NMR, ^13^C NMR, LC–MS, and elemental analysis techniques.

#### Cyclocarbonylative Sonogashira of 2-Ethynylbenzamide (**1**) and Iodobenzene (**2a**) in CH_2_Cl_2_ at 100 °C (Table 1, Entry 1 and Table 2, Entry 1)

Following the general procedure, 2.8 mg (0.004 mmol) of PdCl_2_(PPh_3_)_2_, 145.2 mg (1.0 mmol) of 2-ethynylbenzamide (**1**), 204.0 mg (1.0 mmol) of iodobenzene (**2a**), 1.5 mL of Et_3_N, and 4 mL of CH_2_Cl_2_ were put in the autoclave. The resulting mixture was stirred for 4 h at 100 °C. The crude product was purified through column chromatography (SiO_2_, *n*-hexane/AcOEt 1:1), obtaining 85 mg (yield 34%) of (*Z*)-3-(2-oxo-2-phenylethylidene)isoindolin-1-one (**3a**), and 52 mg (yield 21%) of 3-amino-2-benzoyl-1*H*-inden-1-one (**4a**).

**3a**. ^1^H NMR (600 MHz, DMSO-*d*_6_) δ (ppm): 7.41 (1H, s), 7.58–7.61 (2H, m), 7.67–7.70 (1H, m), 7.72–7.74 (1H, m), 7.81–7.83 (1H, m), 7.85–7.86 (1H, m), 8.20–8.21 (2H, m), 8.35–8.37 (1H, m), 10.92 (1H, br s). ^13^C NMR (150 MHz, DMSO-*d*_6_) δ (ppm): 95.7, 122.6, 123.4, 128.1 (2C), 128.5, 128.8 (2C), 132.1, 133.1, 133.2, 137.2, 137.8, 147.6, 168.8, 189.8. LC–MS (APCI^+^) *m*/*z*: 250.1 [M + H]^+^. Anal. Calcd for C_16_H_11_NO_2_: C, 77.10, H, 4.45, N, 5.62. Found: C, 77.19; H, 4.41; N, 5.63.

**4a**. ^1^H NMR (600 MHz, DMSO-*d*_6_) δ (ppm): 7.39–7.42 (2H, m), 7.47–7.51 (2H, m), 7.60–7.62 (2H, m), 7.63–7.67 (2H, m), 8.06–8.10 (1H, m), 10.09 (1H, br s), 10.21 (1H, br s). ^13^C NMR (150 MHz, DMSO-*d*_6_) δ (ppm): 103.0, 121.4, 121.5, 127.2 (2C), 128.5 (2C), 130.5, 132.3, 133.5, 134.9, 135.5, 140.1, 172.2, 186.7, 190.2. LC–MS (APCI^+^) *m*/*z*: 250.1 [M + H]^+^. Anal. Calcd for C_16_H_11_NO_2_: C, 77.10, H, 4.45, N, 5.62. Found: C, 77.17; H, 4.37; N, 5.61.

#### Cyclocarbonylative Sonogashira of 2-Ethynylbenzamide (**1**) and Iodobenzene (**2a**) in CH_2_Cl_2_ at 50 °C (Table 1, Entry 5)

Following the general procedure, 2.8 mg (0.004 mmol) of PdCl_2_(PPh_3_)_2_, 145.2 mg (1.0 mmol) of 2-ethynylbenzamide (**1**), 204.0 mg (1.0 mmol) of iodobenzene (**2a**), 1.5 mL of Et_3_N, and 4 mL of CH_2_Cl_2_ were put in the autoclave. The resulting mixture was stirred for 24 h at 50 °C. The crude product was purified through column chromatography (SiO_2_, CH_2_Cl_2_), obtaining 105 mg (yield 42%) of (*Z*)-2-(1-hydroxy-3-oxo-3-phenylprop-1-en-1-yl)benzonitrile (**5a**).

**5a**. ^1^H NMR (600 MHz, CDCl_3_) δ (ppm): 6.95 (1H, s), 7.48–7.51 (2H, m), 7.56–7.59 (1H, m), 7.61–7.64 (1H, m), 7.71–7.74 (1H, m), 7.82–7.83 (1H, m), 7.97–8.01 (3H, m), 16.44 (1 H, br s). ^13^C NMR (150 MHz, CDCl_3_) δ (ppm): 96.0, 110.6, 118.0, 127.4 (2C), 128.8 (2C), 129.0, 131.5, 132.8, 133.0, 134.7, 134.7, 139.1, 183.1, 186.2. LC–MS (APCI^+^) *m*/*z*: 250.1 [M + H]^+^. Anal. Calcd for C_16_H_11_NO_2_: C, 77.10, H, 4.45, N, 5.62. Found: C, 77.18; H, 4.39; N, 5.61.

#### Cyclocarbonylative Sonogashira of 2-Ethynylbenzamide (**1**) and Iodobenzene (**2a**) in THF at 100 °C (Table 1, Entry 6 and Table 3, Entry 1)

Following the general procedure, 2.8 mg (0.004 mmol) of PdCl_2_(PPh_3_)_2_, 145.2 mg (1.0 mmol) of 2-ethynylbenzamide (**1**), 204.0 mg (1.0 mmol) of iodobenzene (**2a**), 1.5 mL of Et_3_N, and 4 mL of THF were put in the autoclave. The resulting mixture was stirred for 4 h at 100 °C. The crude product was purified through column chromatography (SiO_2_, CH_2_Cl_2_), obtaining 110 mg (yield 44%) of (*Z*)-2-(1-hydroxy-3-oxo-3-phenylprop-1-en-1-yl) benzonitrile (**5a**).

#### Cyclocarbonylative Sonogashira of 2-Ethynylbenzamide (**1**) and Iodobenzene (**2a**) in Acetonitrile at 100 °C (Table 1, Entry 9)

Following the general procedure, 2.8 mg (0.004 mmol) of PdCl_2_(PPh_3_)_2_, 145.2 mg (1.0 mmol) of 2-ethynylbenzamide (**1**), 204.0 mg (1.0 mmol) of iodobenzene (**2a**), 1.5 mL of Et_3_N, and 4 mL of acetonitrile were put in the autoclave. The resulting mixture was stirred for 4 h at 100 °C. The composition of the crude product was determined by the ^1^H NMR analysis, resulting in a mixture of (*Z*)-3-(2-oxo-2-phenylethylidene)isoindolin-1-one (**3a**) and (*Z*)-2-(1-hydroxy-3-oxo-3-phenylprop-1-en-1-yl) benzonitrile (**5a**) in the molar ratio 78/22.

#### Cyclocarbonylative Sonogashira of 2-Ethynylbenzamide (**1**) and Iodobenzene (**2a**) in DMF at 100 °C (Table 1, Entry 11)

Following the general procedure, 2.8 mg (0.004 mmol) of PdCl_2_(PPh_3_)_2_, 145.2 mg (1.0 mmol) of 2-ethynylbenzamide (**1**), 204.0 mg (1.0 mmol) of iodobenzene (**2a**), 1.5 mL of Et_3_N, and 4 mL of DMF were put in the autoclave. The resulting mixture was stirred for 4 h at 100 °C. The composition of the crude product was determined by the ^1^H NMR analysis, resulting in a mixture of (*Z*)-3-(2-oxo-2-phenylethylidene)isoindolin-1-one (**3a**), 3-amino-2-benzoyl-1*H*-inden-1-one (**4a**) and (*Z*)-2-(1-hydroxy-3-oxo-3-phenylprop-1-en-1-yl) benzonitrile (**5a**) in the molar ratio 33/32/35.

#### Cyclocarbonylative Sonogashira of 2-Ethynylbenzamide (**1**) and 4-Iodoanisole (**2b**) in CH_2_Cl_2_ (Table 2, Entry 2)

Following the general procedure, 2.8 mg (0.004 mmol) of PdCl_2_(PPh_3_)_2_, 145.2 mg (1.0 mmol) of 2-ethynylbenzamide (**1**), 234.0 mg (1.0 mmol) of 4-iodoanisole (**2b**), 1.5 mL of Et_3_N, and 4 mL of CH_2_Cl_2_ were put in the autoclave. The resulting mixture was stirred for 4 h at 100 °C. The crude product was purified through column chromatography (SiO_2_, *n*-hexane/AcOEt 1:1), obtaining 106 mg (yield 38%) of (*Z*)-3-(2-(4-methoxyphenyl)-2-oxoethylidene)isoindolin-1-one (**3b**) and 84 mg (yield 30%) of 3-amino-2-(4-methoxybenzoyl)-1*H*-inden-1-one (**4b**).

**3b**. ^1^H NMR (600 MHz, CDCl_3_) δ (ppm): 3.84 (3H, s), 6.79 (1H, s), 6.91–6.93 (2H, m), 7.56–7.58 (1H, m), 7.61–7.64 (1H, m), 7.77–7.78 (1H, m), 7.82–7.83 (1H, m), 7.96–7.99 (2H, m), 10.57 (1H, br s). ^13^C NMR (150 MHz, CDCl_3_) δ (ppm): 55.5, 94.75 113.9 (2C), 121.0, 124.0, 129.2, 130.2 (2C), 131.2, 131.7, 132.7, 137.1, 147.6, 163.5, 169.0, 189.4. LC–MS (APCI^+^) *m*/*z*: 280.1 [M + H]^+^. Anal. Calcd for C_17_H_13_NO_3_: C, 73.11, H, 4.69, N, 5.02. Found: C, 73.07; H, 4.78; N, 5.02.

**4b**. ^1^H NMR (600 MHz, DMSO-*d*_6_) δ (ppm): 3.82 (3H, s), 6.93–6.95 (2H, m), 7.49–7.51 (1H, m), 7.62–7.66 (4H, m), 8.02–8.05 (1H, m), 9.96 (1H, br s), 10.14 (1H, br s). ^13^C NMR (150 MHz, DMSO-*d*_6_) δ (ppm): 55.3, 103.1, 112.5, 121.3, 121.3 (2C), 130.9 (2C), 132.2, 132.4, 133.4, 135.0, 135.5, 161.4, 172.2, 186.8, 189.18. LC–MS (APCI^+^) *m*/*z*: 280.1 [M + H]^+^. Anal. Calcd for C_17_H_13_NO_3_: C, 73.11, H, 4.69, N, 5.02. Found: C, 73.05; H, 4.74; N, 5.01.

#### Cyclocarbonylative Sonogashira of 2-Ethynylbenzamide (**1**) and 2-Iodoanisole (**2c**) in CH_2_Cl_2_ (Table 2, Entry 5)

Following the general procedure, 7.1 mg (0.01 mmol) of PdCl_2_(PPh_3_)_2_, 145.2 mg (1.0 mmol) of 2-ethynylbenzamide (**1**), 234.0 mg (1.0 mmol) of 2-iodoanisole (**2c**), 1.5 mL of Et_3_N, and 4 mL of CH_2_Cl_2_ were put in the autoclave. The resulting mixture was stirred for 24 h at 100 °C. The crude product was purified through column chromatography (SiO_2_, *n*-hexane/AcOEt 1:1), obtaining 104 mg (yield 37%) of (*Z*)-3-(2-(2-methoxyphenyl)-2-oxoethylidene)isoindolin-1-one (**3c**) and 81 mg (yield 29%) of 3-amino-2-(2-methoxybenzoyl)-1*H*-inden-1-one (**4c**).

**3c**. ^1^H NMR (600 MHz, CDCl_3_) δ (ppm): 3.95 (3H, s), 6.94 (1H, s), 7.00–7.01 (1H, m), 7.03–7.06 (1H, m), 7.47–7.50 (1H, m), 7.58–7.64 (2H, m), 7.73–7.76 (2H, m), 7.87–7.88 (1H, m), 10.48 (1H, br s). ^13^C NMR (150 MHz, CDCl_3_) δ (ppm): 55.8, 100.2, 111.7, 120.9, 121.1, 123.5, 124.1, 130.6, 131.6, 132.7, 133.5, 135.9, 137.4, 146.5, 158.1, 169.1, 192.3. LC–MS (APCI^+^) *m*/*z*: 280.1 [M + H]^+^. Anal. Calcd for C_17_H_13_NO_3_: C, 73.11, H, 4.69, N, 5.02. Found: C, 73.22; H, 4.63; N, 5.03.

**4c**. ^1^H NMR (600 MHz, DMSO-*d*_6_) δ (ppm): 3.64 (3H, s), 6.91–6.93 (1H, m), 7.00–7.01 (1H, m), 7.08–7.10 (1H, m), 7.34–7.36 (1H, m), 7.40–7.42 (1H, m), 7.60–7.62 (2H, m), 8.03–8.04 (1H, m), 9.98 (1H, s), 10.06 (1H, s). ^13^C NMR (150 MHz, DMSO-*d*_6_) δ (ppm): 55.4, 104.5, 111.1, 119.7, 121.2, 121.6, 127.6, 130.0, 131.6, 132.3, 133.4, 135.1, 135.6, 156.4, 170.7, 186.6, 189.2. LC–MS (APCI^+^) *m*/*z*: 280.1 [M + H]^+^. Anal. Calcd for C_17_H_13_NO_3_: C, 73.11; H, 4.69; N, 5.02. Found: C, 73.18; H, 4.64; N, 5.02.

#### Cyclocarbonylative Sonogashira of 2-Ethynylbenzamide (**1**) and 1-Chloro-4-iodobenzene (**2d**) in CH_2_Cl_2_ (Table 2, Entry 6)

Following the general procedure, 2.8 mg (0.004 mmol) of PdCl_2_(PPh_3_)_2_, 145.2 mg (1.0 mmol) of 2-ethynylbenzamide (**1**), 238.5 mg (1.0 mmol) of 1-chloro-4-iodobenzene (**2d**), 1.5 mL of Et_3_N, and 4 mL of CH_2_Cl_2_ were put in the autoclave. The resulting mixture was stirred for 4 h at 100 °C. The crude product was purified through column chromatography (SiO_2_, *n*-hexane/AcOEt 1:1), obtaining 91 mg (yield 32%) of (*Z*)-3-(2-(4-chlorophenyl)-2-oxoethylidene)isoindolin-1-one (**3d**) and 105 mg (yield 37%) of 3-amino-2-(4-chlorobenzoyl)-1*H*-inden-1-one (**4d**).

**3d**. ^1^H NMR (600 MHz, CDCl_3_) δ (ppm): 6.81 (1H, s), 7.49 (2H, d, *J* = 8.7 Hz), 7.64–7.70 (2H, m), 7.82–7.83 (1H, m), 7.90–7.91 (1H, m), 7.98 (2H, d, *J* = 8.7 Hz), 10.57 (1H, br s). ^13^C NMR (150 MHz, CDCl_3_) δ (ppm): 94.2, 121.1, 124.3, 129.0 (2C), 129.3 (2C), 130.6, 132.1, 132.9, 136.7, 137.00, 139.4, 148.9, 169.00, 189.6. LC–MS (APCI^+^) *m*/*z*: 283.9 [M + H]^+^. Anal. Calcd for C_16_H_10_ClNO_2_: C, 67.74; H, 3.55; N, 4.94. Found: C, 67.68; H, 3.61; N, 4.95.

**4d**. ^1^H NMR (600 MHz, DMSO-*d*_6_) δ (ppm): 7.46 (2H, d, *J* = 8.4 Hz), 7.50–7.51 (1H, m), 7.61 (2H, d, *J* = 8.4 Hz), 7.64–7.66 (2H, m), 8.06–8.08 (1H, m), 10.14 (1H, br s), 10.19 (1H, br s). ^13^C NMR (150 MHz, DMSO-*d*_6_) δ (ppm): 102.9, 121.5, 121.7, 127.3 (2C), 130.4 (2C), 132.5, 133.8, 134.9, 135.2, 135.5, 138.8, 172.2, 186.8, 188.7. LC–MS (APCI^+^) *m*/*z*: 283.9 [M + H]^+^. Anal. Calcd for C_16_H_10_ClNO_2_: C, 67.74; H, 3.55; N, 4.94. Found: C, 67.63; H, 3.59; N, 4.95.

#### Cyclocarbonylative Sonogashira of 2-Ethynylbenzamide (**1**) and 4-Iodobenzonitrile (**2e**) in CH_2_Cl_2_ (Table 2, Entry 7)

Following the general procedure, 2.8 mg (0.004 mmol) of PdCl_2_(PPh_3_)_2_, 145.2 mg (1.0 mmol) of 2-ethynylbenzamide (**1**), 229.0 mg (1.0 mmol) of 4-iodobenzonitrile (**2e**), 1.5 mL of Et_3_N, and 4 mL of CH_2_Cl_2_ were put in the autoclave. The resulting mixture was stirred for 4 h at 100 °C. The crude product was purified through column chromatography (SiO_2_, *n*-hexane/AcOEt 1:1), obtaining 41 mg (yield 15%) of (*Z*)-4-(2-(3-oxoisoindolin-1-ylidene) acetyl)benzonitrile (**3e**) and 121 mg (yield 44%) of 4-(3-amino-1-oxo-1*H*-indene-2-carbonyl)benzonitrile (**4e**).

**3e**. ^1^H NMR (600 MHz, CDCl_3_) δ (ppm): 6.81 (1H, s), 7.67–7.73 (2H, m), 7.82–7.85 (3H, m), 7.92–7.94 (1H, m), 8.12 (2H, d, *J* = 8.4 Hz), 10.56 (1H, br s). ^13^C NMR (150 MHz, CDCl_3_) δ (ppm): 93.8, 116.1, 117.9, 121.3, 124.4, 128.3 (2C), 132.4, 132.5 (2C), 132.8, 133.1, 136.8, 141.6, 150.0, 168.9, 189.3. LC–MS (APCI^+^) *m*/*z*: 275.1 [M + H]^+^. Anal. Calcd for C_17_H_10_N_2_O_2_: C, 74.44; H, 3.67; N, 10.21. Found: C, 74.52; H, 3.60; N, 10.22.

**4e**. ^1^H NMR (600 MHz, DMSO-*d*_6_) δ (ppm): 7.50–7.52 (1H, m), 7.66–7.68 (2H, m), 7.70 (2H, d, *J* = 8.7 Hz), 7.87 (2H, d, *J* = 8.7 Hz), 8.09–8.11 (1H, m), 10.23 (1H, br s), 10.26 (1H, br s). ^13^C NMR (150 MHz, DMSO-*d*_6_) δ (ppm): 112.4, 118.7, 121.5, 121.9, 128.9 (2C), 131.4 (2C), 132.0, 132.6, 133.8, 134.8, 135.6, 144.3, 172.2, 186.7, 188.3. LC–MS (APCI^+^) *m*/*z*: 275.1 [M + H]^+^. Anal. Calcd for C_17_H_10_N_2_O_2_: C, 74.44; H, 3.67; N, 10.21. Found: C, 74.51; H, 3.59; N, 10.21.

#### Cyclocarbonylative Sonogashira of 2-Ethynylbenzamide (**1**) and 4-Iodoanisole (**2b**) in THF (Table 3, Entry 2)

Following the general procedure, 2.8 mg (0.004 mmol) of PdCl_2_(PPh_3_)_2_, 145.2 mg (1.0 mmol) of 2-ethynylbenzamide (**1**), 234.0 mg (1.0 mmol) of 4-iodoanisole (**2b**), 1.5 mL of Et_3_N, and 4 mL of THF were put in the autoclave. The resulting mixture was stirred for 4 h at 100 °C. The crude product was purified through column chromatography (SiO_2_, CH_2_Cl_2_), obtaining 115 mg (yield 41%) of (*Z*)-2-(1-hydroxy-3-(4-methoxyphenyl)-3-oxoprop-1-en-1-yl) benzonitrile (**5b**).

**5b**. ^1^H NMR (600 MHz, CDCl_3_) δ (ppm): 3.88 (3H, s), 6.91 (1H, s), 6.98 (2H, d, *J* = 9.0 Hz), 7.60 (1H, t), 7.71 (1H, t), 7.82 (1H, d, *J* = 7.8 Hz), 7.96–7.99 (3H, m), 16.59 (1H, br s). ^13^C NMR (150 MHz, CDCl_3_) δ (ppm): 55.5, 95.4, 110.4, 114.1 (2C), 118.1, 127.4, 128.9, 129.7 (2C), 131.2, 132.8, 134.7, 139.2, 163.7, 181.2, 186.5. LC–MS (APCI^+^) *m*/*z*: 280.1 [M + H]^+^. Anal. Calcd for C_17_H_13_NO_3_: C, 73.11; H, 4.69; N, 5.02. Found: C, 73.21; H, 4.62; N, 5.03.

#### Cyclocarbonylative Sonogashira of 2-Ethynylbenzamide (**1**) and 1-Chloro-4-iodobenzene (**2d**) in THF (Table 3, Entry 3)

Following the general procedure, 2.8 mg (0.004 mmol) of PdCl_2_(PPh_3_)_2_, 145.2 mg (1.0 mmol) of 2-ethynylbenzamide (**1**), 238.5 mg (1.0 mmol) of 1-chloro-4-iodobenzene (**2d**), 1.5 mL of Et_3_N, and 4 mL of THF were put in the autoclave. The resulting mixture was stirred for 4 h at 100 °C. The crude product was purified through column chromatography (SiO_2_, CH_2_Cl_2_), obtaining 111 mg (yield 39%) of (*Z*)-2-(3-(4-chlorophenyl)-1-hydroxy-3-oxoprop-1-en-1-yl)benzonitrile (**5d**).

**5d**. ^1^H NMR (600 MHz, CDCl_3_) δ (ppm): 6.93 (1H, s), 7.47 (2H, d, *J* = 8.4 Hz), 7.62–7.65 (1H, m), 7.72–7.75 (1H, m), 7.84 (1H, d, *J* = 7.8 Hz), 7.94 (2H, d, *J* = 8.4 Hz), 7.98 (1H, d, *J* = 7.8 Hz), 16.35 (1H, br s). ^13^C NMR (150 MHz, CDCl_3_) δ (ppm): 96.0, 110.6, 118.0, 128.8 (2C), 129.1, 129.2 (2C), 131.6, 132.8, 133.2, 134.8, 138.9, 139.4, 183.0, 185.1. LC–MS (APCI^+^) *m*/*z*: 284.1 [M + H]^+^. Anal. Calcd for C_16_H_10_ClNO_2_: C, 67.74; H, 3.55; N, 4.94. Found: C, 67.82; H, 3.49; N, 4.93.

#### Cyclocarbonylative Sonogashira of 2-Ethynylbenzamide (**1**) and 4-Iodobenzonitrile (**2e**) in THF (Table 3, Entry 4)

Following the general procedure, 2.8 mg (0.004 mmol) of PdCl_2_(PPh_3_)_2_, 145.2 mg (1.0 mmol) of 2-ethynylbenzamide (**1**), 229.0 mg (1.0 mmol) of 4-iodobenzonitrile (**2e**), 1.5 mL of Et_3_N, and 4 mL of THF were put in the autoclave. The resulting mixture was stirred for 4 h at 100 °C. The crude product was purified through column chromatography (SiO_2_, CH_2_Cl_2_), obtaining 61 mg (yield 22%) of (*Z*)-2-(3-(4-cyanophenyl)-1-hydroxy-3-oxoprop-1-en-1-yl)benzonitrile (**5e**).

**5e**. ^1^H NMR (600 MHz, CDCl_3_) δ (ppm): 7.00 (1H, s), 7.66–7.69 (1H, m), 7.75–7.77 (1H, m), 7.81 (2H, d, *J* = 8.4 Hz), 7.86–7.87 (1H, m), 8.01 (1H, d, *J* = 7.8 Hz), 8.09 (2H, d, *J* = 8.4 Hz), 16.16 (1H, br s). ^13^C NMR (150 MHz, CDCl_3_) δ (ppm): 96.7, 110.6, 116.0, 117.9, 117.9, 127.8 (2C), 129.2, 132.0, 132.5 (2C), 132.9, 134.8, 138.5, 138.6, 182.9, 184.7. LC–MS (APCI^+^) *m*/*z*: 275.1 [M + H]^+^. Anal. Calcd for C_17_H_10_N_2_O_2_: C, 74.44; H, 3.67; N, 10.21. Found: C, 74.55; H, 3.61; N, 10.21.

### Cyclocarbonylative Sonogashira Reactions of *N*-(4-Chlorophenyl)-2-ethynylbenzamide (**6**)

#### General Procedure

A Pyrex Schlenk tube under a CO atmosphere was charged with *N*-(4-chlorophenyl)-2-ethynylbenzamide (**6**) (1.0 mmol), haloarene (1.0 mmol), Et_3_N (1.5 mL), and CH_2_Cl_2_ (4.0 mL). This solution was introduced by a steel siphon into a 25 mL stainless steel autoclave, fitted with a Teflon inner crucible, and a stirring bar, previously carried with PdCl_2_(PPh_3_)_2_ (0.4 mol %) and placed under vacuum (0.1 Torr). The reactor was pressurized with CO (20–40 atm) and the mixture was stirred for a selected time at a selected temperature. After removal of excess CO (fume hood), the reaction mixture was diluted with CH_2_Cl_2_ (20 mL), washed with brine (15 mL), dried over anhydrous Na_2_SO_4_, and the solvent was removed under vacuum. The reagent conversion and the product composition were determined by ^1^H NMR spectroscopic analysis. All crude products were purified through column chromatography on neutral alumina and characterized with ^1^H NMR, ^13^C NMR, LC–MS, and elemental analysis techniques.

#### Cyclocarbonylative Sonogashira of *N*-(4-Chlorophenyl)-2-ethynylbenzamide (**6**) and Iodobenzene (**2a**) at 100 °C (Table 4, Entry 1)

Following the general procedure, 2.8 mg (0.004 mmol) of PdCl_2_(PPh_3_)_2_, 255.7 mg (1.0 mmol) of *N*-(4-chlorophenyl)-2-ethynylbenzamide (**6**), 204.0 mg (1.0 mmol) of iodobenzene (**2a**), 1.5 mL of Et_3_N, and 4 mL of CH_2_Cl_2_ were put in the autoclave charged with 20 atm of CO. The resulting mixture was stirred for 4 h at 100 °C. The crude product was purified through column chromatography (neutral Al_2_O_3_, *n*-hexane/AcOEt 4:1), obtaining 238 mg (yield 66%) of (*E*)-2-(4-chlorophenyl)-3-(2-oxo-2-phenylethylidene)isoindolin-1-one [(*E*)-**7a**] and 22 mg (yield 6%) of (*Z*)-2-(4-chlorophenyl)-3-(2-oxo-2-phenylethylidene)isoindolin-1-one [(*Z*)-**7a**].

(*E*)-**7a**. ^1^H NMR (600 MHz, CDCl_3_) δ (ppm): 6.53 (1H, s), 7.36 (2H, d, *J* = 7.6 Hz), 7.46 (2H, t, *J* = 7.8 Hz), 7.55–7.59 (3H, m), 7.67–7.69 (1H, m), 7.72–7.75 (1H, m), 7.84 (2H, d, *J* = 7.6 Hz), 7.97 (1H, d, *J* = 7.2 Hz), 8.94 (1H, d, *J* = 7.8 Hz). ^13^C NMR (150 MHz, CDCl_3_) δ (ppm): 105.3, 123.6, 127.3, 128.1 (2C), 128.6 (2C), 129.4, 130.1 (4C), 131.9, 132.3, 132.9, 133.6, 133.8, 135.0, 138.8, 149.2, 166.8, 189.6. LC–MS (APCI^+^) *m*/*z*: 360.1 [M + H]^+^. Anal. Calcd for C_22_H_14_ClNO_2_: C, 73.44; H, 3.92; N, 3.89. Found: C, 73.41; H, 3.98; N, 3.88.

(*Z*)-**7a**. ^1^H NMR (600 MHz, CDCl_3_) δ (ppm): 6.61 (1H, s), 6.99 (2H, d, *J* = 8.4 Hz), 7.15 (2H, d, *J* = 8.4 Hz), 7.36–7.38 (2H, m), 7.49–7.52 (1H, m), 7.63–7.65 (2H, m), 7.68 (1H, d, *J* = 7.8 Hz), 7.73–7.76 (1H, m), 7.88 (1H, d, *J* = 7.8 Hz), 7.96 (1H, d, *J* = 7.8 Hz). ^13^C NMR (150 MHz, CDCl_3_) δ (ppm): 101.6, 105.4, 120.3, 124.3, 128.3 (2C), 128.4 (2C), 128.5 (2C), 128.9 (2C), 130.1, 130.2, 131.2, 133.1, 133.2, 137.2, 138.0, 143.0, 167.5, 191.2. LC–MS (APCI^+^) *m*/*z*: 360.1 [M + H]^+^. Anal. Calcd for C_22_H_14_ClNO_2_: C, 73.44; H, 3.92; N, 3.89. Found: C, 73.40; H, 3.99; N, 3.88.

#### Cyclocarbonylative Sonogashira of *N*-(4-Chlorophenyl)-2-ethynylbenzamide (**6**) and Iodobenzene (**2a**) at 50 °C (Table 4, Entry 2)

Following the general procedure, 2.8 mg (0.004 mmol) of PdCl_2_(PPh_3_)_2_, 255.7 mg (1.0 mmol) of *N*-(4-chlorophenyl)-2-ethynylbenzamide (**6**), 204.0 mg (1.0 mmol) of iodobenzene (**2a**), 1.5 mL of Et_3_N. and 4 mL of CH_2_Cl_2_ were put in the autoclave charged with 20 atm of CO. The resulting mixture was stirred for 24 h at 50 °C. The composition of the crude product was determined by the ^1^H NMR analysis, resulting in a mixture of (*E*)-2-(4-chlorophenyl)-3-(2-oxo-2-phenylethylidene)isoindolin-1-one [(*E*)-**7a**] and (*Z*)-2-(4-chlorophenyl)-3-(2-oxo-2-phenyl-ethylidene)isoindolin-1-one [(*Z*)-**7a**] in the molar ratio 90/10.

#### Cyclocarbonylative Sonogashira of *N*-(4-Chlorophenyl)-2-ethynylbenzamide (**6**) and 4-Iodoanisole (**2b**) (Table 4, Entry 3)

Following the general procedure, 2.8 mg (0.004 mmol) of PdCl_2_(PPh_3_)_2_, 255.7 mg (1.0 mmol) of *N*-(4-chlorophenyl)-2-ethynylbenzamide (**6**), 234.0 mg (1.0 mmol) of 4-iodoanisole (**2b**), 1.5 mL of Et_3_N, and 4 mL of CH_2_Cl_2_ were put in the autoclave charged with 20 atm of CO. The resulting mixture was stirred for 4 h at 100 °C. The crude product was purified through column chromatography (neutral Al_2_O_3_, *n*-hexane/AcOEt 3:1), obtaining 269 mg (yield 69%) of (*E*)-2-(4-chlorophenyl)-3-(2-(4-methoxyphenyl)-2-oxoethylidene)isoindolin-1-one [(*E*)-**7b**] and 39 mg (yield 10%) of (*Z*)-2-(4-chlorophenyl)-3-(2-(4-methoxyphenyl)-2-oxoethylidene)isoindolin-1-one [(*Z*)-**7b**].

(*E*)-**7b**. ^1^H NMR (600 MHz, CDCl_3_) δ (ppm): 3.85 (3H, s), 6.49 (1H, s), 6.91 (2H, d, *J* = 9.0 Hz), 7.36 (2H, d, *J* = 8.7 Hz), 7.56 (2H, d, *J* = 8.7 Hz), 7.63–7.65 (1H, m), 7.68–7.71 (1H, m), 7.83 (2H, d, *J* = 9.0 Hz), 7.94 (1H, d, *J* = 7.2 Hz), 8.86 (1H, d, *J* = 7.8 Hz). ^13^C NMR (150 MHz, CDCl_3_) δ (ppm): 55.5, 105.7, 113.8 (2C), 123.6, 127.38, 129.5, 130.1 (2C), 130.1 (2C), 130.6 (2C), 131.7, 131.7, 132.5, 133.6, 133.9, 135.0, 148.4, 163.5, 166.8, 188.3. LC–MS (APCI^+^) *m*/*z*: 390.1 [M + H]^+^. Anal. Calcd for C_23_H_16_ClNO_3_: C, 70.86; H, 4.14; N, 3.59. Found: C, 70.75; H, 4.21; N, 3.59.

(*Z*)-**7b**. ^1^H NMR (600 MHz, CDCl_3_) δ (ppm): 3.86 (3H, s), 6.58 (1H, s), 6.84 (2H, d, *J* = 9.0 Hz), 7.00 (2H, d, *J* = 8.7 Hz), 7.15 (2H, d, *J* = 8.7 Hz), 7.62 (2H, d, *J* = 9.0 Hz), 7.66 (1H, d, *J* = 7.2 Hz), 7.72–7.74 (1H, m), 7.86 (1H, d, *J* = 7.8 Hz), 7.95 (1H, d, *J* = 7.8 Hz). ^13^C NMR (150 MHz, CDCl_3_) δ (ppm): 55.5, 102.1, 113.5 (2C), 120.2, 124.2, 127.9, 128.6 (2C), 128.8 (2C), 130.8 (2C), 131.0, 131.1, 133.1, 133.3, 134.3, 137.2, 142.0, 163.6, 167.5, 189.8. LC–MS (APCI^+^) *m*/*z*: 390.1 [M + H]^+^. Anal. Calcd for C_23_H_16_ClNO_3_: C, 70.86; H, 4.14; N, 3.59. Found: C, 70.78; H, 4.22; N, 3.59.

#### Cyclocarbonylative Sonogashira of *N*-(4-Chlorophenyl)-2-ethynylbenzamide (**6**) and 2-Iodoanisole (**2c**) (Table 4, Entry 4)

Following the general procedure, 2.8 mg (0.004 mmol) of PdCl_2_(PPh_3_)_2_, 255.7 mg (1.0 mmol) of *N*-(4-chlorophenyl)-2-ethynylbenzamide (**6**), 234.0 mg (1.0 mmol) of 2-iodoanisole (**2c**), 1.5 mL of Et_3_N, and 4 mL of CH_2_Cl_2_ were put in the autoclave charged with 20 atm of CO. The resulting mixture was stirred for 4 h at 100 °C. The crude product was purified through column chromatography (neutral Al_2_O_3_, *n*-hexane/AcOEt 3:1), obtaining 289 mg (yield 74%) of (*E*)-2-(4-chlorophenyl)-3-(2-(2-methoxyphenyl)-2-oxoethylidene)isoindolin-1-one [(*E*)-**7c**] and 12 mg (yield 3%) of (*Z*)-2-(4-chlorophenyl)-3-(2-(2-methoxyphenyl)-2-oxoethylidene)isoindolin-1-one [(*Z*)-**7c**].

(*E*)-**7c**. ^1^H NMR (600 MHz, CDCl_3_) δ (ppm): 3.72 (3H, s), 6.63 (1H, s), 6.91 (1H, d, *J* = 8.4 Hz), 7.01–7.04 (1H, m), 7.33 (2H, d, *J* = 8.7 Hz), 7.44–7.47 (1H, m), 7.53 (2H, d, *J* = 8.7 Hz), 7.66–7.68 (1H, m), 7.73–7.77 (2H, m), 7.96 (1H, d, *J* = 7.8 Hz), 9.15 (1H, d, *J* = 8.4 Hz). ^13^C NMR (150 MHz, CDCl_3_) δ (ppm): 55.3, 110.5, 111.6, 120.8, 123.6, 127.5, 129.5, 129.6, 129.7 (2C), 130.3 (2C), 130.8, 131.7, 132.7, 133.6, 133.7, 133.9, 134.8, 147.6, 158.1, 167.0, 189.7. LC–MS (APCI^+^) *m*/*z*: 390.1 [M + H]^+^. Anal. Calcd for C_23_H_16_ClNO_3_: C, 70.86; H, 4.14; N, 3.59. Found: C, 70.77; H, 4.20; N, 3.59.

(*Z*)-**7c**. ^1^H NMR (600 MHz, CDCl_3_) δ (ppm): 3.89 (3H, s), 6.77 (1H, s), 6.92 (2H, t, *J* = 7.8 Hz), 7.13 (2H, d, *J* = 8.7 Hz), 7.25 (2H, d, *J* = 8.7 Hz), 7.38–7.40 (2H, m), 7.62–7.65 (1H, m), 7.69–7.72 (1H, m), 7.82 (1H, d, *J* = 7.8 Hz), 7.94 (1H, d, *J* = 7.8 Hz).

#### Cyclocarbonylative Sonogashira of *N*-(4-Chlorophenyl)-2-ethynylbenzamide (**6**) and 1-Chloro-4-iodobenzene (**2d**) with 20 atm of CO (Table 4, Entry 5)

Following the general procedure, 2.8 mg (0.004 mmol) of PdCl_2_(PPh_3_)_2_, 255.7 mg (1.0 mmol) of *N*-(4-chlorophenyl)-2-ethynylbenzamide (**6**), 238.5 mg (1.0 mmol) of 1-chloro-4-iodobenzene (**2d**), 1.5 mL of Et_3_N, and 4 mL of CH_2_Cl_2_ were put in the autoclave charged with 20 atm of CO. The resulting mixture was stirred for 4 h at 100 °C. The crude product was purified through column chromatography (neutral Al_2_O_3_, *n*-hexane/AcOEt 3:1), obtaining 201 mg (yield 51%) of (*E*)-2-(4-chlorophenyl)-3-(2-(4-chlorophenyl)-2-oxoethylidene)isoindolin-1-one [(*E*)-**7d**], 28 mg (yield 7%) of (*Z*)-2-(4-chlorophenyl)-3-(2-(4-chlorophenyl)-2-oxoethylidene)isoindolin-1-one [(*Z*)-**7d**], and 11 mg (yield 3%) of (*Z*)-3-(4-chlorobenzylidene)-2-(4-chlorophenyl)isoindolin-1-one (**8**).

(*E*)-**7d**. ^1^H NMR (600 MHz, CDCl_3_) δ (ppm): 6.45 (1H, s), 7.35 (2H, d, *J* = 9.0 Hz), 7.42 (2H, d, *J* = 8.4 Hz), 7.57 (2H, d, *J* = 8.4 Hz), 7.68 (1H, t, *J* = 7.8 Hz), 7.72–7.77 (3H, m), 7.96 (1H, d, *J* = 7.2 Hz), 8.94 (1H, d, *J* = 7.8 Hz). ^13^C NMR (150 MHz, CDCl_3_) δ (ppm): 104.7, 123.8, 127.5, 128.9 (2C), 129.6 (2C), 130.1 (2C), 130.2 (2C), 132.1, 132.3, 133.7, 133.8, 134.5, 135.2, 137.2, 139.5, 149.9, 166.9, 188.3. LC–MS (APCI^+^) *m*/*z*: 394.1 [M + H]^+^. Anal. Calcd for C_22_H_13_Cl_2_NO_2_: C, 67.02; H, 3.32; N, 3.55. Found: C, 67.08; H, 3.35; N, 3.55.

(*Z*)-**7d**. ^1^H NMR (600 MHz, CDCl_3_) δ (ppm): 6.56 (1H, s), 7.00 (2H, d, *J* = 8.4 Hz), 7.18 (2H, d, *J* = 8.4 Hz), 7.34 (2H, d, *J* = 8.4 Hz), 7.58 (2H, d, *J* = 8.4 Hz), 7.67 (1H, t, *J* = 7.2 Hz), 7.75 (1H, t, *J* = 7.2 Hz), 7.87 (1H, d, *J* = 7.2 Hz), 7.96 (1H, d, *J* = 7.8 Hz). ^13^C NMR (150 MHz, CDCl_3_) δ (ppm): 100.8, 120.3, 124.4, 127.8, 128.4 (2C), 128.6, 128.7 (2C), 129.0 (2C), 129.7 (2C), 131.4, 133.3, 133.5, 134.4, 136.3, 137.1, 139.5, 143.5, 189.8. LC–MS (APCI^+^) *m*/*z*: 394.1 [M + H]^+^. Anal. Calcd for C_22_H_13_Cl_2_NO_2_: C, 67.02; H, 3.32; N, 3.55. Found: C, 67.10; H, 3.37; N, 3.55.

**8**. ^1^H NMR (600 MHz, CDCl_3_) δ (ppm): 6.75 (1H, s), 6.80 (2H, d, *J* = 8.4 Hz), 6.97 (2H, d, *J* = 8.4 Hz), 7.01 (2H, d, *J* = 8.7 Hz), 7.11 (2H, d, *J* = 8.7 Hz), 7.57 (1H, t, *J* = 7.2 Hz), 7.69 (1H, t, *J* = 7.2 Hz), 7.84 (1H, d, *J* = 7.8 Hz), 7.95 (1H, d, *J* = 7.8 Hz). ^13^C NMR (150 MHz, CDCl_3_) δ (ppm): 106.1, 110.6, 119.4, 124.0, 127.4, 127.5 (2C), 128.3 (2C), 128.4 (2C), 129.5, 129.8, 130.3 (2C), 130.7, 132.7, 134.2, 134.7, 138.3, 167.7. LC–MS (APCI^+^) *m*/*z*: 366.0 [M + H]^+^. Anal. Calcd for C_21_H_13_Cl_2_NO: C, 68.87; H, 3.58; N, 3.82. Found: C, 68.81; H, 3.54; N, 3.81.

#### Cyclocarbonylative Sonogashira of *N*-(4-Chlorophenyl)-2-ethynylbenzamide (**6**) and 1-Chloro-4-iodobenzene (**2d**) with 40 atm of CO (Table 4, Entry 6)

Following the general procedure, 2.8 mg (0.004 mmol) of PdCl_2_(PPh_3_)_2_, 255.7 mg (1.0 mmol) of *N*-(4-chlorophenyl)-2-ethynylbenzamide (**6**), 238.5 mg (1.0 mmol) of 1-chloro-4-iodobenzene (**2d**), 1.5 mL of Et_3_N, and 4 mL of CH_2_Cl_2_ were put in the autoclave charged with 40 atm of CO. The resulting mixture was stirred for 4 h at 100 °C. The composition of the crude product was determined by ^1^H NMR analysis, resulting in a mixture of (*E*)-2-(4-chlorophenyl)-3-(2-(4-chlorophenyl)-2-oxoethylidene)isoindolin-1-one [(*E*)-**7d**], (*Z*)-2-(4-chlorophenyl)-3-(2-(4-chlorophenyl)-2-oxoethylidene)isoindolin-1-one [(*Z*)-**7d**], and (*Z*)-3-(4-chlorobenzylidene)-2-(4-chlorophenyl)isoindolin-1-one (**8**) in the molar ratio 86/10/4.

#### Cyclocarbonylative Sonogashira of *N*-(4-Chlorophenyl)-2-ethynylbenzamide (**6**) and 1-Iodonaphthalene (**2f**) (Table 4, Entry 7)

Following the general procedure, 2.8 mg (0.004 mmol) of PdCl_2_(PPh_3_)_2_, 255.7 mg (1.0 mmol) of *N*-(4-chlorophenyl)-2-ethynylbenzamide (**6**), 254.1 mg (1.0 mmol) of 1-iodonaphthalene (**2f**), 1.5 mL of Et_3_N, and 4 mL of CH_2_Cl_2_ were put in the autoclave charged with 20 atm of CO. The resulting mixture was stirred for 4 h at 100 °C. The crude product was purified through column chromatography (neutral Al_2_O_3_, *n*-hexane/AcOEt 6:1), obtaining 283 mg (yield 69%) of (*E*)-2-(4-chlorophenyl)-3-(2-(naphthalen-1-yl)-2-oxoethylidene)isoindolin-1-one [(*E*)-**7f**] and 21 mg (yield 5%) of (*Z*)-2-(4-chlorophenyl)-3-(2-(naphthalen-1-yl)-2-oxoethylidene)isoindolin-1-one [(*Z*)-**7f**].

(*E*)-**7f**. ^1^H NMR (600 MHz, CDCl_3_) δ (ppm): 6.41 (1H, s), 7.33 (2H, d, *J* = 8.7 Hz), 7.46 (1H, t, *J* = 7.8 Hz), 7.49 (2H, d, *J* = 8.7 Hz), 7.54 (1H, t, *J* = 7.2 Hz), 7.59 (1H, t, *J* = 7.2 Hz), 7.69–7.72 (2H, m), 7.76 (1H, t, *J* = 7.8 Hz), 7.89 (1H, d, *J* = 8.4 Hz), 7.96–8.00 (2H, m), 8.56 (1H, d, *J* = 8.4 Hz), 9.11 (1H, d, *J* = 7.8 Hz). ^13^C NMR (150 MHz, CDCl_3_) δ (ppm): 109.0, 123.7, 124.4, 125.4, 126.5, 127.6, 127.7, 127.7, 128.5, 129.1, 129.5, 130.0 (4C), 130.1, 132.0, 132.2, 132.6, 133.8, 133.8, 135.0, 137.7, 149.1, 166.9, 192.9. LC–MS (APCI^+^) *m*/*z*: 410.0 [M + H]^+^. Anal. Calcd for C_26_H_16_ClNO_2_: C, 76.19; H, 3.93; N, 3.42. Found: C, 76.07; H, 3.99; N, 3.43.

(*Z*)-**7f**. ^1^H NMR (600 MHz, CDCl_3_) δ (ppm): 6.62 (1H, s), 6.84 (2H, d, *J* = 8.4 Hz), 6.88 (2H, d, *J* = 8.4 Hz), 7.40 (1H, t, *J* = 7.8 Hz), 7.49–7.54 (2H, m), 7.68 (1H, t, *J* = 7.8 Hz), 7.76 (1H, t, *J* = 7.8 Hz), 7.79–7.82 (2H, m), 7.89 (1H, d, *J* = 7.2 Hz), 7.95 (2H, t, *J* = 6.6 Hz), 8.32–8.33 (1H, m). ^13^C NMR (150 MHz, CDCl_3_) δ (ppm): 104.3 120.4, 124.0, 124.3, 125.6, 126.7, 127.5 (2C), 127.7, 127.9, 128.0, 129.1 (2C), 130.1, 130.1, 131.3, 133.0, 133.3, 133.5, 133.9, 135.5, 137.4, 142.8, 167.6, 193.5. LC–MS (APCI^+^) *m*/*z*: 410.0 [M + H]^+^. Anal. Calcd for C_26_H_16_ClNO_2_: C, 76.19; H, 3.93; N, 3.42. Found: C, 76.11; H, 3.97; N, 3.43.

#### Cyclocarbonylative Sonogashira of *N*-(4-Chlorophenyl)-2-ethynylbenzamide (**6**) and 4-Iodotoluene (**2g**) (Table 4, Entry 8)

Following the general procedure, 2.8 mg (0.004 mmol) of PdCl_2_(PPh_3_)_2_, 255.7 mg (1.0 mmol) of *N*-(4-chlorophenyl)-2-ethynylbenzamide (**6**), 92.1 mg (1.0 mmol) of 4-iodotoluene (**2g**), 1.5 mL of Et_3_N, and 4 mL of CH_2_Cl_2_ were put in the autoclave charged with 20 atm of CO. The resulting mixture was stirred for 4 h at 100 °C. The crude product was purified through column chromatography (neutral Al_2_O_3_, *n*-hexane/AcOEt 3:1), obtaining 251 mg (yield 67%) of (*E*)-2-(4-chlorophenyl)-3-(2-oxo-2-(*p*-tolyl)ethylidene)isoindolin-1-one [(*E*)-**7g**] and 26 mg (yield 7%) of (*Z*)-2-(4-chlorophenyl)-3-(2-oxo-2-(*p*-tolyl)ethylidene)isoindolin-1-one [(*Z*)-**7g**].

(*E*)-**7g**. ^1^H NMR (600 MHz, CDCl_3_) δ (ppm): 2.39 (3H, s), 6.51 (1H, s), 7.24 (2H, d, *J* = 8.4 Hz), 7.36 (2H, d, *J* = 8.4 Hz), 7.56 (2H, d, *J* = 8.4 Hz), 7.65 (1H, t, *J* = 7.8 Hz), 7.70 (1H, t, *J* = 7.8 Hz), 7.74 (2H, d, *J* = 8.4 Hz), 7.94 (1H, d, *J* = 7.2 Hz), 8.91 (1H, d, *J* = 7.8 Hz). ^13^C NMR (150 MHz, CDCl_3_) δ (ppm): 21.6, 105.6, 123.6, 127.4, 128.4 (2C), 129.3 (2C), 129.5, 130.1 (2C), 130.1 (2C), 131.8, 132.5, 133.6, 133.9, 135.0, 136.4, 143.9, 148.8, 166.9, 189.4. LC–MS (APCI^+^) *m*/*z*: 374.1 [M + H]^+^. Anal. Calcd for C_23_H_16_ClNO_2_: C, 73.90; H, 4.31; N, 3.75. Found: C, 74.01; H, 4.25; N, 3.76.

(*Z*)-**7g**. ^1^H NMR (600 MHz, CDCl_3_) δ (ppm): 2.40 (3H, s), 6.61 (1H, s), 7.01 (2H, d, *J* = 9.0 Hz), 7.15 (2H, d, *J* = 9.0 Hz), 7.17 (2H, d, *J* = 8.1 Hz), 7.56 (2H, d, *J* = 8.1 Hz), 7.66 (1H, t, *J* = 7.2 Hz), 7.74 (1H, t, *J* = 7.8 Hz), 7.87 (1H, d, *J* = 7.8 Hz), 7.96 (1H, d, *J* = 7.8 Hz). ^13^C NMR (150 MHz, CDCl_3_) δ (ppm): 21.7, 101.8, 120.2, 124.3, 127.9, 128.5 (2C), 128.6 (2C), 128.9 (2C), 129.0 (2C), 131.1, 133.1, 133.3, 134.4, 135.5, 137.3, 142.6, 144.0, 167.5, 190.7. LC–MS (APCI^+^) *m*/*z*: 374.1 [M + H]^+^. Anal. Calcd for C_23_H_16_ClNO_2_: C, 73.90; H, 4.31; N, 3.75. Found: C, 73.99; H, 4.23; N, 3.76.

#### Cyclocarbonylative Sonogashira of *N*-(4-Chlorophenyl)-2-ethynylbenzamide (**6**) and 2-Iodotoluene (**2h**) (Table 4, Entry 9)

Following the general procedure, 2.8 mg (0.004 mmol) of PdCl_2_(PPh_3_)_2_, 255.7 mg (1.0 mmol) of *N*-(4-chlorophenyl)-2-ethynylbenzamide (**6**), 92.1 mg (1.0 mmol) of 2-iodotoluene (**2h**), 1.5 mL of Et_3_N, and 4 mL of CH_2_Cl_2_ were put in the autoclave charged with 20 atm of CO. The resulting mixture was stirred for 4 h at 100 °C. The crude product was purified through column chromatography (neutral Al_2_O_3_, *n*-hexane/AcOEt 3:1), obtaining 225 mg (yield 60%) of (*E*)-2-(4-chlorophenyl)-3-(2-oxo-2-(*o*-tolyl)ethylidene)isoindolin-1-one [(*E*)-**7h**] and 19 mg (yield 5%) of (*Z*)-2-(4-chlorophenyl)-3-(2-oxo-2-(*o*-tolyl)ethylidene)isoindolin-1-one [(*Z*)-**7h**].

(*E*)-**7h**. ^1^H NMR (600 MHz, CDCl_3_) δ (ppm): 2.51 (3H, s), 6.24 (1H, s), 7.20–7.26 (2H, m), 7.31 (2H, d, *J* = 8.4 Hz), 7.36 (1H, t, *J* = 7.8 Hz), 7.45 (1H, d, *J* = 7.2 Hz), 7.52 (2H, d, *J* = 8.4 Hz), 7.69 (1H, t, *J* = 7.8 Hz), 7.75 (1H, t, *J* = 7.2 Hz), 7.97 (1H, d, *J* = 7.2 Hz), 9.00 (1H, d, *J* = 7.8 Hz). ^13^C NMR (150 MHz, CDCl_3_) δ (ppm): 20.7, 108.7, 123.7, 125.8, 127.5, 128.5, 129.6, 130.1 (2C), 130.1 (2C), 131.2, 131.7, 132.0, 132.3, 133.8, 133.9, 135.1, 137.5, 1140.0, 148.8, 166.9, 193.4. LC–MS (APCI^+^) *m*/*z*: 374.1 [M + H]^+^. Anal. Calcd for C_23_H_16_ClNO_2_: C, 73.90; H, 4.31; N, 3.75. Found: C, 74.00; H, 4.24; N, 3.76.

(*Z*)-**7h**. ^1^H NMR (600 MHz, CDCl_3_) δ (ppm): 2.24 (3H, s), 6.47 (1H, s), 6.96 (2H, d, *J* = 9.0 Hz), 7.15 (2H, d, *J* = 9.0 Hz), 7.18–7.20 (2H, m), 7.56–7.62 (2H, m), 7.66 (1H, t, *J* = 7.2 Hz), 7.74 (1H, t, *J* = 7.8 Hz), 7.85 (1H, d, *J* = 7.8 Hz), 7.95 (1H, d, *J* = 7.8 Hz).

#### Cyclocarbonylative Sonogashira of *N*-(4-Chlorophenyl)-2-ethynylbenzamide (**6**) and 4-Iodobenzonitrile (**2e**) (Scheme S4)

Following the general procedure, 2.8 mg (0.004 mmol) of PdCl_2_(PPh_3_)_2_, 255.7 mg (1.0 mmol) of *N*-(4-chlorophenyl)-2-ethynylbenzamide (**6**), 229.0 mg (1.0 mmol) of 4-iodobenzonitrile (**2e**), 1.5 mL of Et_3_N, and 4 mL of CH_2_Cl_2_ were put in the autoclave charged with 20 atm of CO. The resulting mixture was stirred for 4 h at 100 °C. The crude product was purified through column chromatography (neutral Al_2_O_3_, *n*-hexane/AcOEt 3:1), obtaining 70 mg (yield 18%) of (*E*)-4-(2-(2-(4-chlorophenyl)-3-oxoisoindolin-1-ylidene)acetyl)benzonitrile [(*E*)-**7e**], 20 mg (yield 5%) of (*Z*)-4-(2-(2-(4-chlorophenyl)-3-oxoisoindolin-1-ylidene)acetyl)benzonitrile [(*Z*)-**7e**], and 15 mg (yield 4%) of (*Z*)-4-((2-(4-chlorophenyl)-3-oxoisoindolin-1-ylidene)methyl)benzonitrile (**9**).

(*E*)-**7e**. ^1^H NMR (600 MHz, CDCl_3_) δ (ppm): 6.45 (1H, s), 7.35 (2H, d, *J* = 8.4 Hz), 7.59 (2H, d, *J* = 8.4 Hz), 7.72–7.80 (4H, m), 7.90 (2H, d, *J* = 8.4 Hz), 8.00 (1H, d, *J* = 7.8 Hz), 9.04 (1H, d, *J* = 7.8 Hz). ^13^C NMR (150 MHz, CDCl_3_) δ (ppm): 103.7, 116.0, 117.9, 123.9, 127.7, 128.5 (2C), 129.4, 130.0 (2C), 130.3 (2C), 132.1, 132.5 (2C), 132.6, 133.6, 134.0, 135.4, 142.3, 151.3, 166.9, 187.8. LC–MS (APCI^+^) *m*/*z*: 385.2 [M + H]^+^. Anal. Calcd for C_23_H_13_ClN_2_O_2_: C, 71.79; H, 3.41; N, 7.28. Found: C, 71.71; H, 3.46; N, 7.27.

(*Z*)-**7e**. ^1^H NMR (600 MHz, CDCl_3_) δ (ppm): 6.57 (1H, s), 7.00 (2H, d, *J* = 8.4 Hz), 7.20 (2H, d, *J* = 8.4 Hz), 7.67 (2H, d, *J* = 8.4 Hz), 7.70–7.78 (4H, m), 7.88 (1H, d, *J* = 7.2 Hz), 7.97 (1H, d, *J* = 7.2 Hz).

**9**. ^1^H NMR (600 MHz, CDCl_3_) δ (ppm): 6.75 (1H, s), 6.97 (2H, d, *J* = 8.4 Hz), 7.00 (2H, d, *J* = 8.7 Hz), 7.11 (2H, d, *J* = 8.7 Hz), 7.27 (2H, d, *J* = 8.4 Hz), 7.61 (1H, t, *J* = 7.8 Hz), 7.72 (1H, t, *J* = 7.2 Hz), 7.86 (1H, d, *J* = 7.8 Hz), 7.96 (1H, d, *J* = 7.8 Hz). ^13^C NMR (150 MHz, CDCl_3_) δ (ppm): 104.7, 110.1, 118.5, 119.6, 124.2, 127.5, 128.3 (2C), 128.6 (2C), 129.6 (2C), 130.1, 130.9 (2C), 132.0, 133.1, 134.1, 136.5, 138.0, 138.4, 167.6. LC–MS (APCI^+^) *m*/*z*: 357.0 [M + H]^+^. Anal. Calcd for C_22_H_13_ClN_2_O: C, 74.06; H, 3.67; N, 7.85. Found: C, 73.97; H, 3.74; N, 7.86.

#### Cyclocarbonylative Sonogashira of *N*-(4-Chlorophenyl)-2-ethynylbenzamide (**6**) and 4-Bromonitrobenzene (**2i**) (Scheme 4)

Following the general procedure, 2.8 mg (0.004 mmol) of PdCl_2_(PPh_3_)_2_, 255.7 mg (1.0 mmol) of *N*-(4-chlorophenyl)-2-ethynylbenzamide (**6**), 202.0 mg (1.0 mmol) of 4-bromonitrobenzene (**2i**), 1.5 mL of Et_3_N, and 4 mL of CH_2_Cl_2_ were put in the autoclave charged with 20 atm of CO. The resulting mixture was stirred for 4 h at 100 °C. The crude product was purified through column chromatography (SiO_2_, *n*-hexane/AcOEt 9:1), obtaining 218 mg (yield 85%) of 2-(4-chlorophenyl)-3-methyleneisoindolin-1-one (**10**).^45^

^1^H NMR (600 MHz, CDCl_3_) δ (ppm): 4.83 (1H, d, *J* = 2.4 Hz), 5.28 (1H, d, *J* = 2.4 Hz), 7.36 (2H, d, *J* = 9.0 Hz), 7.51 (2H, d, *J* = 9.0 Hz), 7.58–7.61 (1H, m), 7.67–7.69 (1H, m), 7.79 (1H, d, *J* = 7.6 Hz), 7.94 (1H, d, *J* = 7.6 Hz). ^13^C NMR (150 MHz, CDCl_3_) δ (ppm): 90.4, 120.1, 123.6, 129.3 (2C), 129.6 (2C), 129.9, 132.5, 133.1, 133.8, 135.0, 136.2, 142.8, 166.5. LC–MS (APCI^+^) *m*/*z*: 256.0 [M + H]^+^. Anal. Calcd for C_15_H_10_ClNO: C, 70.46; H, 3.94; N, 5.48. Found: 70.54; H, 3.88; N, 5.47.

### Cyclic Sonogashira Reaction of *N*-(4-Chlorophenyl)-2-ethynylbenzamide (**6**) and 1-Chloro-4-iodobenzene (**2d**)

In a 25 mL Carius tube sealed with a Teflon valve, *N*-(4-chlorophenyl)-2-ethynylbenzamide (**6**) (255.7 mg, 1.0 mmol), 1-chloro-4-iodobenzene (**2d**) (238.5 mg, 1.0 mmol), PdCl_2_(PPh_3_)_2_ (2.8 mg, 0.004 mmol), Et_3_N (1.5 mL), and CH_2_Cl_2_ (4 mL) were mixed together. The resulting mixture was left under stirring for 4 h at 100 °C, then it was hydrolyzed with H_2_O (20 mL) and extracted with CH_2_Cl_2_ (3 × 30 mL). The combined organic phases were washed with brine (50 mL), dried over anhydrous Na_2_SO_4_ and the solvent was removed under vacuum. The crude product was purified by column chromatography (neutral Al_2_O_3_, *n*-hexane/AcOEt 6:1), to give 319 mg (yield 87%) of (*Z*)-3-(4-chlorobenzylidene)-2-(4-chlorophenyl)isoindolin-1-one (**8**).

## Supporting Information Available

Supplementary schemes, ^1^H NMR and ^13^C NMR spectra for all the pure compounds (PDF)
