# Supplementary material for: Synthesis of 3-Alkylideneisoindolin-1-ones via Sonogashira Cyclocarbonylative Reactions of 2-Ethynylbenzamides
Source: J Org Chem. 2020 Jul 3;85(15):10022–34. doi: 10.1021/acs.joc.0c01282 (PMC8154568; doi:10.1021/acs.joc.0c01282)
Supplement: Supplementary file 1 — jo0c01282_si_001.pdf [file jo0c01282_si_001.pdf]

Supporting Information

Synthesis of 3-alkylideneisoindolin-1-ones via Sonogashira cyclocarbonylative reactions of 2-ethynylbenzamides

Gianluigi Albano,[a,b] Stefano Giuntini[c,d] and Laura Antonella Aronica[a,*]

[a] Dipartimento di Chimica e Chimica Industriale, Università di Pisa, Via Giuseppe Moruzzi 13, 56124 Pisa, Italy.

[b] Present address: Dipartimento di Chimica, Università degli Studi di Bari "Aldo Moro", Via Edoardo Orabona 4, 70126 Bari, Italy.

[c] Dipartimento di Chimica "Ugo Schiff", Università degli Studi di Firenze, Via della Lastruccia 3, 50019 Sesto Fiorentino, Italy.

[d] Centro di Risonanze Magnetiche (CERM), Università degli Studi di Firenze and Consorzio Interuniversitario Risonanze Magnetiche di Metallo Proteine (CIRMMP), Via Luigi Sacconi 6, 50019 Sesto Fiorentino, Italy

Table of contents

Supplementary Schemes S5

Scheme S1. Synthesis of 2-ethynylbenzamide (1).......................................S5

Scheme S2. Reaction of (*Z*)-2-(1-hydroxy-3-oxo-3-phenylprop-1-en-1-yl) benzonitrile (5a) under the cyclocarbonylative Sonogashira conditions of Table 1, entry 1. ....S5

Scheme S3. Synthesis of *N*-(4-chlorophenyl)-2-ethynylbenzamide (6). S5

Scheme S4. Cyclocarbonylative Sonogashira reaction of *N*-(4-chlorophenyl)-2-ethynylbenzamide (6) with 4-iodobenzonitrile (2e) S5

NMR spectra of pure compounds S6

Figure S1. ^{1}H-NMR spectrum (600 MHz, $CDCl_3$) of 2-((trimethylsilyl)ethynyl)benzamide (B): full scale spectrum (top) and spectrum expansions (bottom). S6

Figure S2. ^{13}C-NMR spectrum (150 MHz, $CDCl_3$) of 2-((trimethylsilyl)ethynyl)benzamide (B): full scale spectrum (top) and spectrum expansions (bottom). S7

Figure S3. ^{1}H-NMR spectrum (600 MHz, $CDCl_3$) of 2-ethynylbenzamide (1): full scale spectrum (top) and spectrum expansions (bottom). S8

Figure S4. ^{13}C-NMR spectrum (150 MHz, $CDCl_3$) of 2-ethynylbenzamide (1): full scale spectrum (top) and spectrum expansions (bottom). S9

Figure S5. ^{1}H-NMR spectrum (600 MHz, $CDCl_3$) of 2-iodobenzoyl chloride (D): full scale spectrum (top) and spectrum expansions (bottom). S10

Figure S6. ^{13}C-NMR spectrum (150 MHz, $CDCl_3$) of 2-iodobenzoyl chloride (D): full scale spectrum (top) and spectrum expansions (bottom). S11

Figure S7. ^{1}H-NMR spectrum (600 MHz, $CDCl_3$) of *N*-(4-chlorophenyl)-2-iodobenzamide (E): full scale spectrum (top) and spectrum expansions (bottom). S12

Figure S8. ^{13}C-NMR spectrum (150 MHz, $CDCl_3$) of *N*-(4-chlorophenyl)-2-iodobenzamide (E): full scale spectrum (top) and spectrum expansions (bottom). S13

Figure S9. ^{1}H-NMR spectrum (600 MHz, $CDCl_3$) of *N*-(4-chlorophenyl)-2-((trimethylsilyl)ethynyl)benzamide (F): full scale spectrum (top) and spectrum expansions (bottom). S14

Figure S10. ^{13}C-NMR spectrum (150 MHz, $CDCl_3$) of *N*-(4-chlorophenyl)-2-((trimethylsilyl)ethynyl)benzamide (F): full scale spectrum (top) and spectrum expansions (bottom). S15

Figure S11. ^{1}H-NMR spectrum (600 MHz, $CDCl_3$) of *N*-(4-chlorophenyl)-2-ethynylbenzamide (6): full scale spectrum (top) and spectrum expansions (bottom). S16

Figure S12. ^{13}C-NMR spectrum (150 MHz, $CDCl_3$) of *N*-(4-chlorophenyl)-2-ethynylbenzamide (6): full scale spectrum (top) and spectrum expansions (bottom). S17

Figure S13. ^{1}H-NMR spectrum (600 MHz, $CDCl_3$) of (*Z*)-3-(2-oxo-2-phenylethylidene)isoindolin-1-one (3a): full scale spectrum (top) and spectrum expansions (bottom). S18

Figure S14. ^{13}C-NMR spectrum (150 MHz, $CDCl_3$) of (*Z*)-3-(2-oxo-2-phenylethylidene)isoindolin-1-one (3a): full scale spectrum (top) and spectrum expansions (bottom). S19

Figure S15. ^{1}H-NMR spectrum (600 MHz, $CDCl_3$) of 3-amino-2-benzoyl-1*H*-inden-1-one (4a): full scale spectrum (top) and spectrum expansions (bottom). S20

Figure S16. ^{13}C-NMR spectrum (150 MHz, $CDCl_3$) of 3-amino-2-benzoyl-1*H*-inden-1-one (4a): full scale spectrum (top) and spectrum expansions (bottom). S21

Figure S17. ^{1}H-NMR spectrum (600 MHz, $CDCl_3$) of (*Z*)-2-(1-hydroxy-3-oxo-3-phenylprop-1-en-1-yl)benzonitrile (5a): full scale spectrum (top) and spectrum expansions (bottom). S22

Figure S18. ^{13}C-NMR spectrum (150 MHz, $CDCl_3$) of (*Z*)-2-(1-hydroxy-3-oxo-3-phenylprop-1-en-1-yl)benzonitrile (5a): full scale spectrum (top) and spectrum expansions (bottom). S23

Figure S19. ^{1}H-NMR spectrum (600 MHz, $CDCl_3$) of (*Z*)-3-(2-(4-methoxyphenyl)-2-oxoethylidene)isoindolin-1-one (3b): full scale spectrum (top) and spectrum expansions (bottom). S24

Figure S20. ^{13}C-NMR spectrum (150 MHz, $CDCl_3$) of (*Z*)-3-(2-(4-methoxyphenyl)-2-oxoethylidene)isoindolin-1-one (3b): full scale spectrum (top) and spectrum expansions (bottom). S25

Figure S21. ^{1}H-NMR spectrum (600 MHz, $CDCl_3$) of 3-amino-2-(4-methoxybenzoyl)-1*H*-inden-1-one (4b): full scale spectrum (top) and spectrum expansions (bottom). S26

Figure S22. ^{13}C-NMR spectrum (150 MHz, $CDCl_3$) of 3-amino-2-(4-methoxybenzoyl)-1*H*-inden-1-one (4b): full scale spectrum (top) and spectrum expansions (bottom). S27

Figure S23. ^{1}H-NMR spectrum (600 MHz, $CDCl_3$) of (*Z*)-3-(2-(2-methoxyphenyl)-2-oxoethylidene)isoindolin-1-one (3c): full scale spectrum (top) and spectrum expansions (bottom). S28

Figure S24. ^{13}C-NMR spectrum (150 MHz, $CDCl_3$) of (*Z*)-3-(2-(2-methoxyphenyl)-2-oxoethylidene)isoindolin-1-one (3c): full scale spectrum (top) and spectrum expansions (bottom). S29

Figure S25. ^{1}H-NMR spectrum (600 MHz, $CDCl_3$) of 3-amino-2-(2-methoxybenzoyl)-1*H*-inden-1-one (4c): full scale spectrum (top) and spectrum expansions (bottom). S30

Figure S26. ^{13}C-NMR spectrum (150 MHz, $CDCl_3$) of 3-amino-2-(2-methoxybenzoyl)-1*H*-inden-1-one (4c): full scale spectrum (top) and spectrum expansions (bottom). S31

Figure S27. ^{1}H-NMR spectrum (600 MHz, $CDCl_3$) of (*Z*)-3-(2-(4-chlorophenyl)-2-oxoethylidene)isoindolin-1-one (3d): full scale spectrum (top) and spectrum expansions (bottom). S32

Figure S28. ^{13}C-NMR spectrum (150 MHz, $CDCl_3$) of (*Z*)-3-(2-(4-chlorophenyl)-2-oxoethylidene)isoindolin-1-one (3d): full scale spectrum (top) and spectrum expansions (bottom). S33

Figure S29. ^{1}H-NMR spectrum (600 MHz, $CDCl_3$) of 3-amino-2-(4-chlorobenzoyl)-1*H*-inden-1-one (4d): full scale spectrum (top) and spectrum expansions (bottom). S34

Figure S30. ^{13}C-NMR spectrum (150 MHz, $CDCl_3$) of 3-amino-2-(4-chlorobenzoyl)-1*H*-inden-1-one (4d): full scale spectrum (top) and spectrum expansions (bottom). S35

Figure S31. ^{1}H-NMR spectrum (600 MHz, $CDCl_3$) of (*Z*)-4-(2-(3-oxoisoindolin-1-ylidene)acetyl)benzonitrile (3e): full scale spectrum (top) and spectrum expansions (bottom). S36

Figure S32. ^{13}C-NMR spectrum (150 MHz, $CDCl_3$) of (*Z*)-4-(2-(3-oxoisoindolin-1-ylidene)acetyl)benzonitrile (3e): full scale spectrum (top) and spectrum expansions (bottom). S37

Figure S33. ^{1}H-NMR spectrum (600 MHz, $CDCl_3$) of 4-(3-amino-1-oxo-1*H*-indene-2-carbonyl)benzonitrile (4e): full scale spectrum (top) and spectrum expansions (bottom). S38

Figure S34. ^{13}C-NMR spectrum (150 MHz, $CDCl_3$) of 4-(3-amino-1-oxo-1*H*-indene-2-carbonyl)benzonitrile (4e): full scale spectrum (top) and spectrum expansions (bottom). S39

Figure S35. ^{1}H-NMR spectrum (600 MHz, $CDCl_3$) of (*Z*)-2-(1-hydroxy-3-(4-methoxyphenyl)-3-oxoprop-1-en-1-yl)benzonitrile (5b): full scale spectrum (top) and spectrum expansions (bottom). S40

Figure S36. ^{13}C-NMR spectrum (150 MHz, $CDCl_3$) of (*Z*)-2-(1-hydroxy-3-(4-methoxyphenyl)-3-oxoprop-1-en-1-yl)benzonitrile (5b): full scale spectrum (top) and spectrum expansions (bottom). S41

Figure S37. ^{1}H-NMR spectrum (600 MHz, $CDCl_3$) of (*Z*)-2-(3-(4-chlorophenyl)-1-hydroxy-3-oxoprop-1-en-1-yl)benzonitrile (5d): full scale spectrum (top) and spectrum expansions (bottom). S42

Figure S38. ^{13}C-NMR spectrum (150 MHz, $CDCl_3$) of (*Z*)-2-(3-(4-chlorophenyl)-1-hydroxy-3-oxoprop-1-en-1-yl)benzonitrile (5d): full scale spectrum (top) and spectrum expansions (bottom). S43

Figure S39. ^{1}H-NMR spectrum (600 MHz, $CDCl_3$) of (*Z*)-2-(3-(4-cyanophenyl)-1-hydroxy-3-oxoprop-1-en-1-yl)benzonitrile (5e): full scale spectrum (top) and spectrum expansions (bottom). S44

Figure S40. ^{13}C-NMR spectrum (150 MHz, $CDCl_3$) of (*Z*)-2-(3-(4-cyanophenyl)-1-hydroxy-3-oxoprop-1-en-1-yl)benzonitrile (5e): full scale spectrum (top) and spectrum expansions (bottom). S45

Figure S41. ^{1}H-NMR spectrum (600 MHz, $CDCl_3$) of (*E*)-2-(4-chlorophenyl)-3-(2-oxo-2-phenylethylidene)isoindolin-1-one ((*E*)-7a): full scale spectrum (top) and spectrum expansions (bottom). S46

Figure S42. ^{13}C-NMR spectrum (150 MHz, $CDCl_3$) of (*E*)-2-(4-chlorophenyl)-3-(2-oxo-2-phenylethylidene)isoindolin-1-one ((*E*)-7a): full scale spectrum (top) and spectrum expansions (bottom). S47

Figure S43. ^{1}H-NMR spectrum (600 MHz, $CDCl_3$) of (*Z*)-2-(4-chlorophenyl)-3-(2-oxo-2-phenylethylidene)isoindolin-1-one ((*Z*)-7a): full scale spectrum (top) and spectrum expansions (bottom). S48

Figure S44. ^{13}C-NMR spectrum (150 MHz, $CDCl_3$) of (*Z*)-2-(4-chlorophenyl)-3-(2-oxo-2-phenylethylidene)isoindolin-1-one ((*Z*)-7a): full scale spectrum (top) and spectrum expansions (bottom). S49
Figure S45. ^{1}H-NMR spectrum (600 MHz, $CDCl_3$) of (*E*)-2-(4-chlorophenyl)-3-(2-(4-methoxyphenyl)-2-oxoethylidene) isoindolin-1-one ((*E*)-7b): full scale spectrum (top) and spectrum expansions (bottom). S50
Figure S46. ^{13}C-NMR spectrum (150 MHz, $CDCl_3$) of (*E*)-2-(4-chlorophenyl)-3-(2-(4-methoxyphenyl)-2-oxoethylidene) isoindolin-1-one ((*E*)-7b): full scale spectrum (top) and spectrum expansions (bottom). S51
Figure S47. ^{1}H-NMR spectrum (600 MHz, $CDCl_3$) of (*Z*)-2-(4-chlorophenyl)-3-(2-(4-methoxyphenyl)-2-oxoethylidene) isoindolin-1-one ((*Z*)-7b): full scale spectrum (top) and spectrum expansions (bottom). S52
Figure S48. ^{13}C-NMR spectrum (150 MHz, $CDCl_3$) of (*Z*)-2-(4-chlorophenyl)-3-(2-(4-methoxyphenyl)-2-oxoethylidene) isoindolin-1-one ((*Z*)-7b): full scale spectrum (top) and spectrum expansions (bottom). S53
Figure S49. ^{1}H-NMR spectrum (600 MHz, $CDCl_3$) of (*E*)-2-(4-chlorophenyl)-3-(2-(2-methoxyphenyl)-2-oxoethylidene) isoindolin-1-one ((*E*)-7c): full scale spectrum (top) and spectrum expansions (bottom). S54
Figure S50. ^{13}C-NMR spectrum (150 MHz, $CDCl_3$) of (*E*)-2-(4-chlorophenyl)-3-(2-(2-methoxyphenyl)-2-oxoethylidene) isoindolin-1-one ((*E*)-7c): full scale spectrum (top) and spectrum expansions (bottom). S55
Figure S51. ^{1}H-NMR spectrum (600 MHz, $CDCl_3$) of (*Z*)-2-(4-chlorophenyl)-3-(2-(2-methoxyphenyl)-2-oxoethylidene) isoindolin-1-one ((*Z*)-7c): full scale spectrum (top) and spectrum expansions (bottom). S56
Figure S52. ^{1}H-NMR spectrum (600 MHz, $CDCl_3$) of (*E*)-2-(4-chlorophenyl)-3-(2-(4-chlorophenyl)-2-oxoethylidene)isoindolin-1-one ((*E*)-7d): full scale spectrum (top) and spectrum expansions (bottom). S57
Figure S53. ^{13}C-NMR spectrum (150 MHz, $CDCl_3$) of (*E*)-2-(4-chlorophenyl)-3-(2-(4-chlorophenyl)-2-oxoethylidene)isoindolin-1-one ((*E*)-7d): full scale spectrum (top) and spectrum expansions (bottom). S58
Figure S54. ^{1}H-NMR spectrum (600 MHz, $CDCl_3$) of (*Z*)-2-(4-chlorophenyl)-3-(2-(4-chlorophenyl)-2-oxoethylidene)isoindolin-1-one ((*Z*)-7d): full scale spectrum (top) and spectrum expansions (bottom). S59
Figure S55. ^{13}C-NMR spectrum (150 MHz, $CDCl_3$) of (*Z*)-2-(4-chlorophenyl)-3-(2-(4-chlorophenyl)-2-oxoethylidene)isoindolin-1-one ((*Z*)-7d): full scale spectrum (top) and spectrum expansions (bottom). S60
Figure S56. ^{1}H-NMR spectrum (600 MHz, $CDCl_3$) of (*Z*)-3-(4-chlorobenzylidene)-2-(4-chlorophenyl)isoindolin-1-one (8): full scale spectrum (top) and spectrum expansions (bottom). S61
Figure S57. ^{13}C-NMR spectrum (150 MHz, $CDCl_3$) of (*Z*)-3-(4-chlorobenzylidene)-2-(4-chlorophenyl)isoindolin-1-one (8): full scale spectrum (top) and spectrum expansions (bottom). S62
Figure S58. ^{1}H-NMR spectrum (600 MHz, $CDCl_3$) of (*E*)-2-(4-chlorophenyl)-3-(2-(naphthalen-1-yl)-2-oxoethylidene)isoindolin-1-one ((*E*)-7f): full scale spectrum (top) and spectrum expansions (bottom). S63
Figure S59. ^{13}C-NMR spectrum (150 MHz, $CDCl_3$) of (*E*)-2-(4-chlorophenyl)-3-(2-(naphthalen-1-yl)-2-oxoethylidene)isoindolin-1-one ((*E*)-7f): full scale spectrum (top) and spectrum expansions (bottom). S64
Figure S60. ^{1}H-NMR spectrum (600 MHz, $CDCl_3$) of (*Z*)-2-(4-chlorophenyl)-3-(2-(naphthalen-1-yl)-2-oxoethylidene)isoindolin-1-one ((*Z*)-7f): full scale spectrum (top) and spectrum expansions (bottom). S65
Figure S61. ^{13}C-NMR spectrum (150 MHz, $CDCl_3$) of (*Z*)-2-(4-chlorophenyl)-3-(2-(naphthalen-1-yl)-2-oxoethylidene)isoindolin-1-one ((*Z*)-7f): full scale spectrum (top) and spectrum expansions (bottom). S66
Figure S62. ^{1}H-NMR spectrum (600 MHz, $CDCl_3$) of (*E*)-2-(4-chlorophenyl)-3-(2-oxo-2-(*p*-tolyl)ethylidene)isoindolin-1-one ((*E*)-7g): full scale spectrum (top) and spectrum expansions (bottom). S67
Figure S63. ^{13}C-NMR spectrum (150 MHz, $CDCl_3$) of (*E*)-2-(4-chlorophenyl)-3-(2-oxo-2-(*p*-tolyl)ethylidene)isoindolin-1-one ((*E*)-7g): full scale spectrum (top) and spectrum expansions (bottom). S68
Figure S64. ^{1}H-NMR spectrum (600 MHz, $CDCl_3$) of (*Z*)-2-(4-chlorophenyl)-3-(2-oxo-2-(*p*-tolyl)ethylidene)isoindolin-1-one ((*Z*)-7g): full scale spectrum (top) and spectrum expansions (bottom). S69
Figure S65. ^{13}C-NMR spectrum (150 MHz, $CDCl_3$) of (*Z*)-2-(4-chlorophenyl)-3-(2-oxo-2-(*p*-tolyl)ethylidene)isoindolin-1-one ((*Z*)-7g): full scale spectrum (top) and spectrum expansions (bottom). S70
Figure S66. ^{1}H-NMR spectrum (600 MHz, $CDCl_3$) of (*E*)-2-(4-chlorophenyl)-3-(2-oxo-2-(*o*-tolyl)ethylidene)isoindolin-1-one ((*E*)-7h): full scale spectrum (top) and spectrum expansions (bottom). S71
Figure S67. ^{13}C-NMR spectrum (150 MHz, $CDCl_3$) of (*E*)-2-(4-chlorophenyl)-3-(2-oxo-2-(*o*-tolyl)ethylidene)isoindolin-1-one ((*E*)-7h): full scale spectrum (top) and spectrum expansions (bottom). S72
Figure S68. ^{1}H-NMR spectrum (600 MHz, $CDCl_3$) of (*Z*)-2-(4-chlorophenyl)-3-(2-oxo-2-(*o*-tolyl)ethylidene)isoindolin-1-one ((*Z*)-7h): full scale spectrum (top) and spectrum expansions (bottom). S73
Figure S69. ^{1}H-NMR spectrum (600 MHz, $CDCl_3$) of (*E*)-4-(2-(2-(4-chlorophenyl)-3-oxoisoindolin-1-ylidene)acetyl)benzonitrile ((*E*)-7e): full scale spectrum (top) and spectrum expansions (bottom). S74
Figure S70. ^{13}C-NMR spectrum (150 MHz, $CDCl_3$) of (*E*)-4-(2-(2-(4-chlorophenyl)-3-oxoisoindolin-1-ylidene)acetyl)benzonitrile ((*E*)-7e): full scale spectrum (top) and spectrum expansions (bottom). S75
Figure S71. ^{1}H-NMR spectrum (600 MHz, $CDCl_3$) of (*Z*)-4-(2-(2-(4-chlorophenyl)-3-oxoisoindolin-1-ylidene)acetyl)benzonitrile ((*Z*)-7e): full scale spectrum (top) and spectrum expansions (bottom). S76
Figure S72. ^{1}H-NMR spectrum (600 MHz, $CDCl_3$) of (*Z*)-4-((2-(4-chlorophenyl)-3-oxoisoindolin-1-ylidene)methyl)benzonitrile (9): full scale spectrum (top) and spectrum expansions (bottom). S77
Figure S73. ^{13}C-NMR spectrum (150 MHz, $CDCl_3$) of (*Z*)-4-((2-(4-chlorophenyl)-3-oxoisoindolin-1-ylidene)methyl)benzonitrile (9): full scale spectrum (top) and spectrum expansions (bottom). S78

Figure S74. ^{1}H-NMR spectrum (600 MHz, $CDCl_3$) of 2-(4-chlorophenyl)-3-methyleneisoindolin-1-one (10): full scale spectrum (top) and spectrum expansions (bottom). S79
Figure S75. ^{13}C-NMR spectrum (150 MHz, $CDCl_3$) of 2-(4-chlorophenyl)-3-methyleneisoindolin-1-one (10): full scale spectrum (top) and spectrum expansions (bottom). S80

Supplementary Schemes

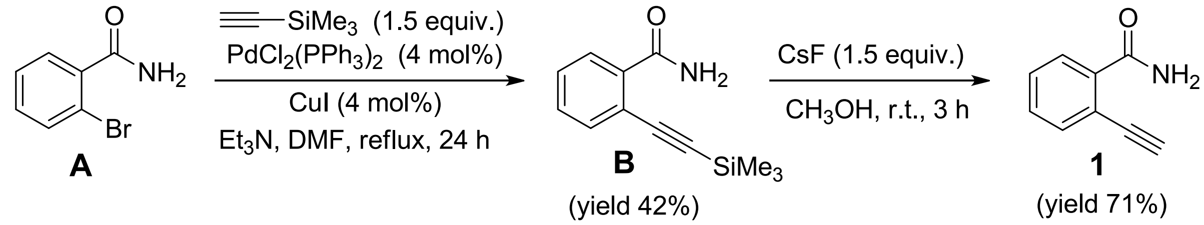

Scheme S1. Synthesis of 2-ethynylbenzamide (**1**).

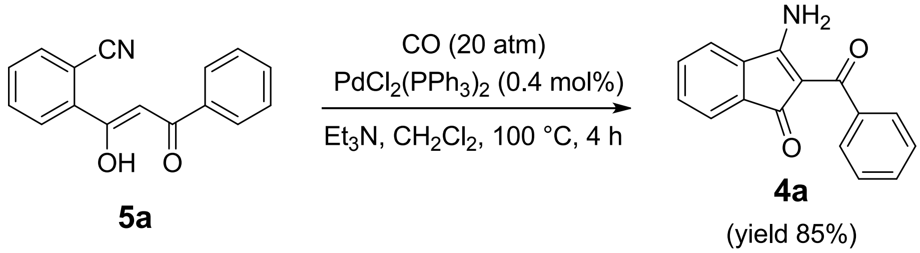

Scheme S2. Reaction of (*Z*)-2-(1-hydroxy-3-oxo-3-phenylprop-1-en-1-yl) benzonitrile (**5a**) under the cyclocarbonylative Sonogashira conditions of Table 1, entry 1.

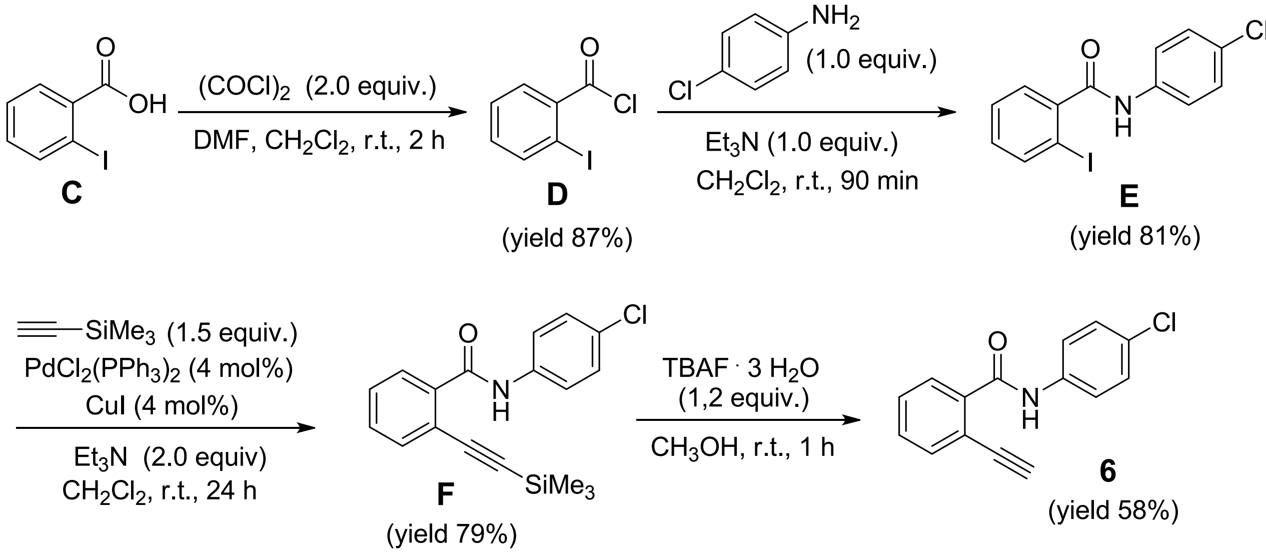

Scheme S3. Synthesis of *N*-(4-chlorophenyl)-2-ethynylbenzamide (**6**).

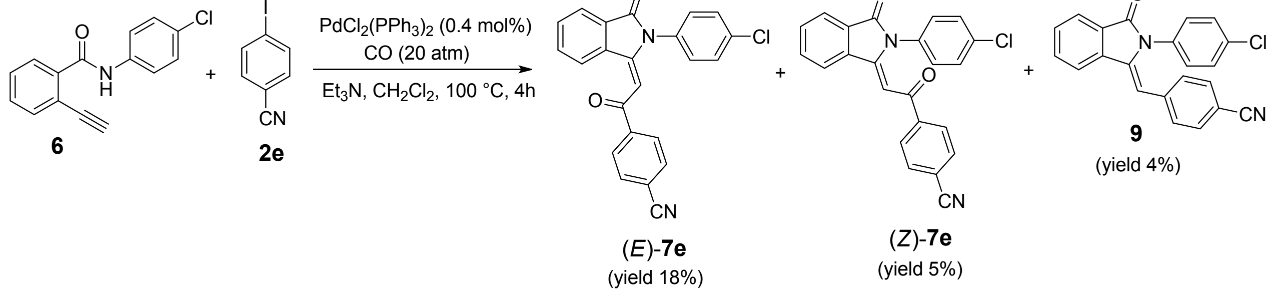

Scheme S4. Cyclocarbonylative Sonogashira reaction of *N*-(4-chlorophenyl)-2-ethynylbenzamide (**6**) with 4-iodobenzonitrile (**2e**) .

NMR spectra of pure compounds

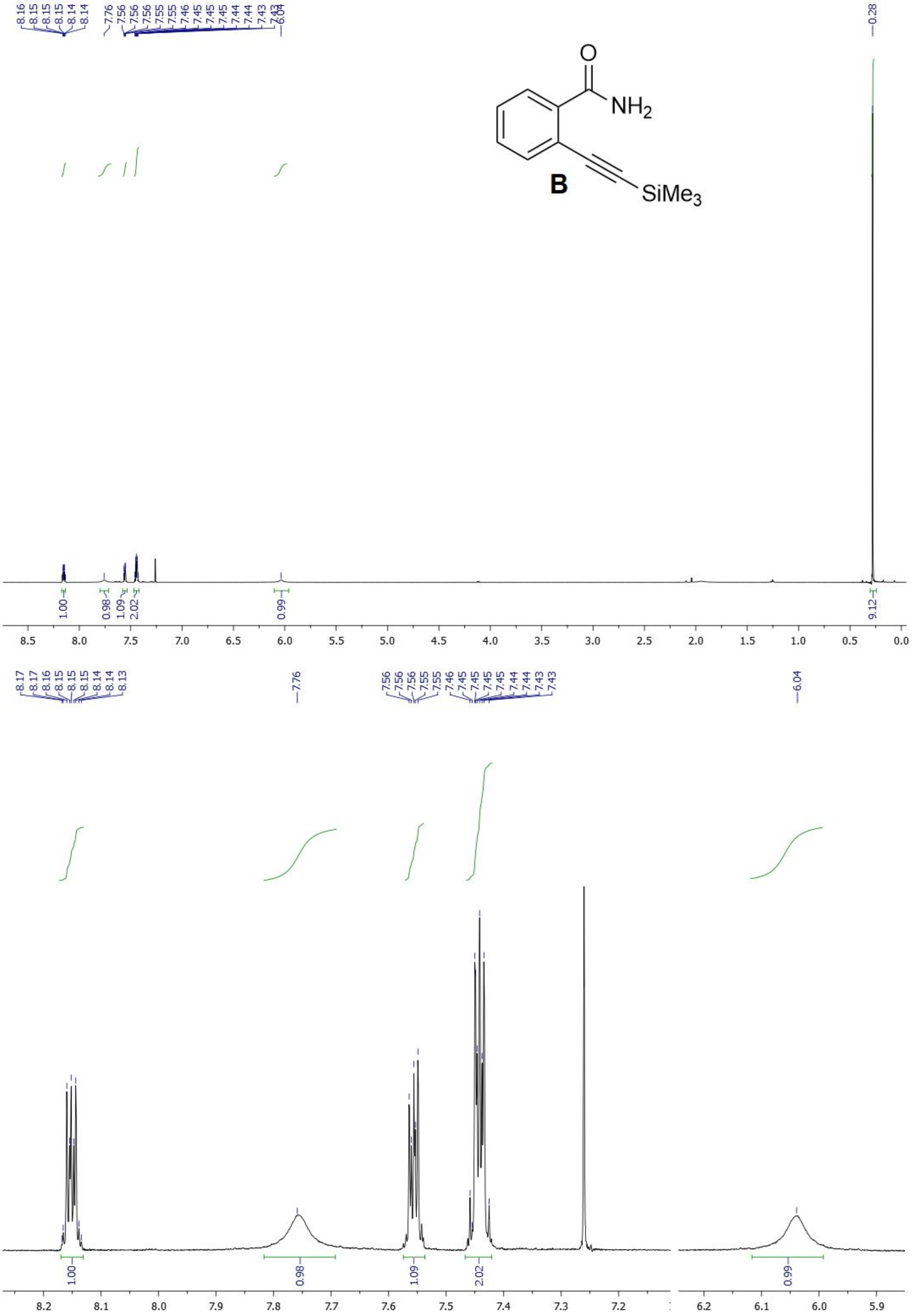

Figure S1. ^{1}H-NMR spectrum (600 MHz, $CDCl_3$) of 2-((trimethylsilyl)ethynyl)benzamide (**B**): full scale spectrum (top) and spectrum expansions (bottom).

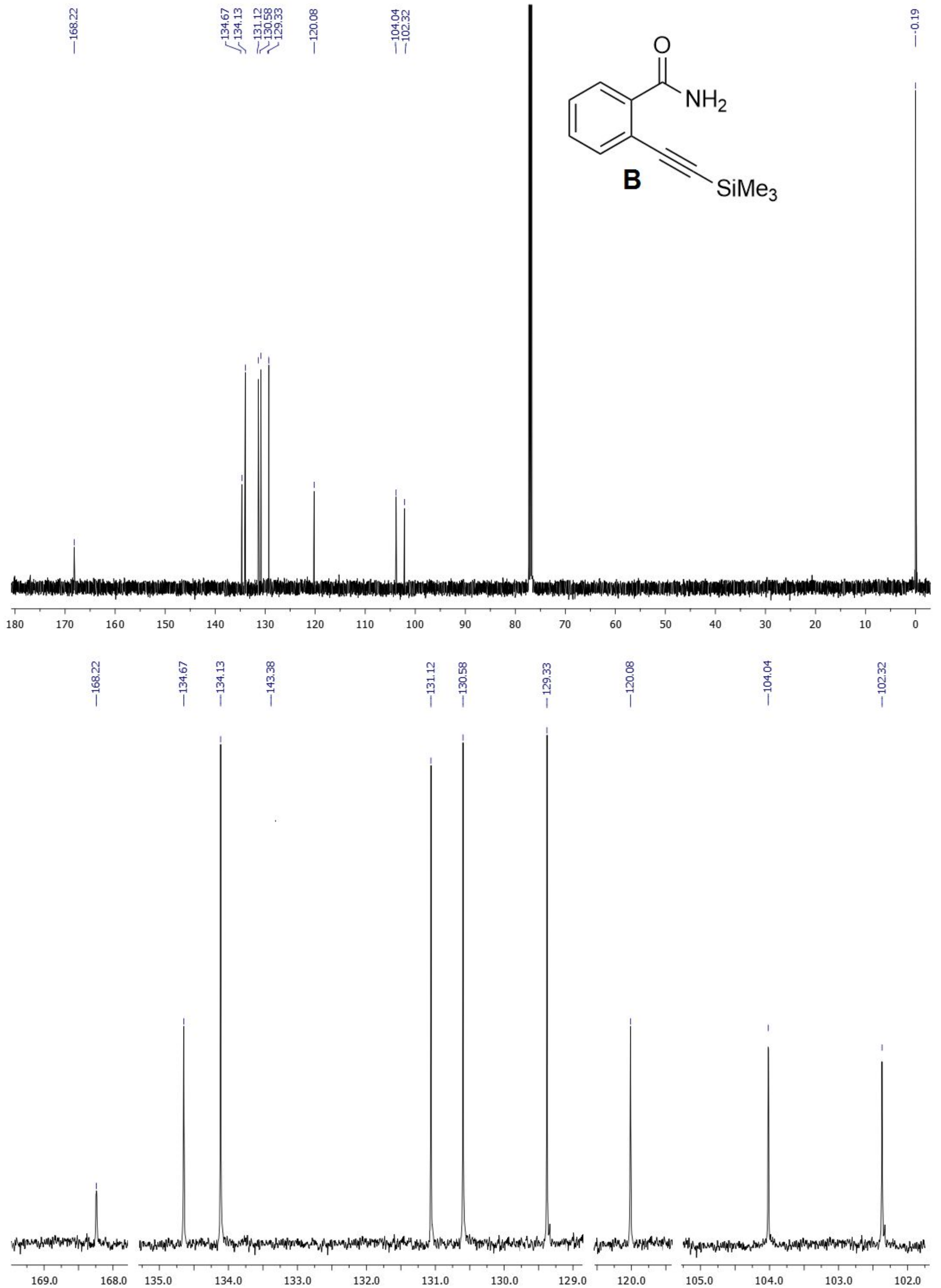

Figure S2. ^{13}C-NMR spectrum (150 MHz, $CDCl_3$) of 2-((trimethylsilyl)ethynyl)benzamide (**B**): full scale spectrum (top) and spectrum expansions (bottom).

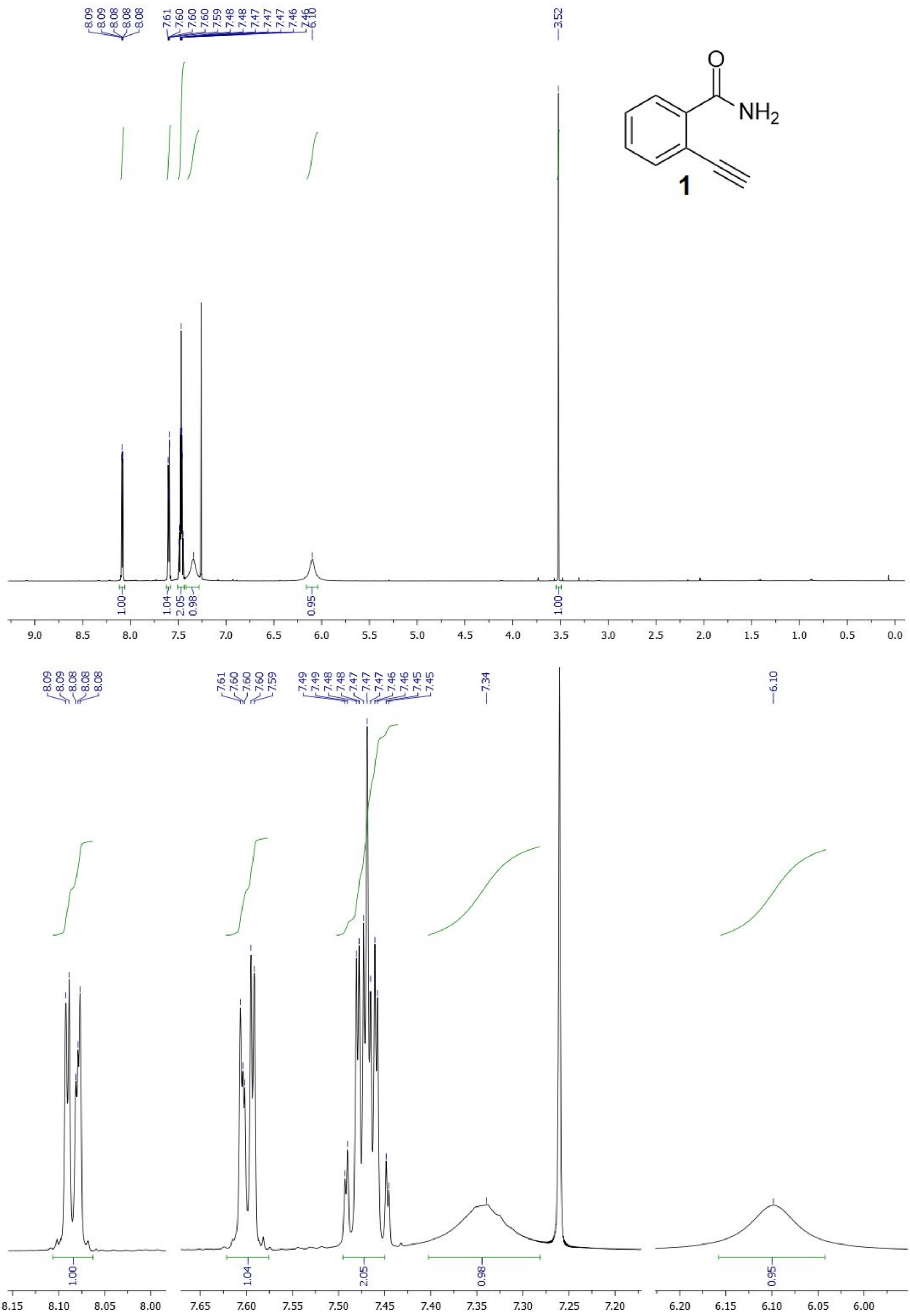

Figure S3. ^{1}H-NMR spectrum (600 MHz, $CDCl_3$) of 2-ethynylbenzamide (**1**): full scale spectrum (top) and spectrum expansions (bottom).

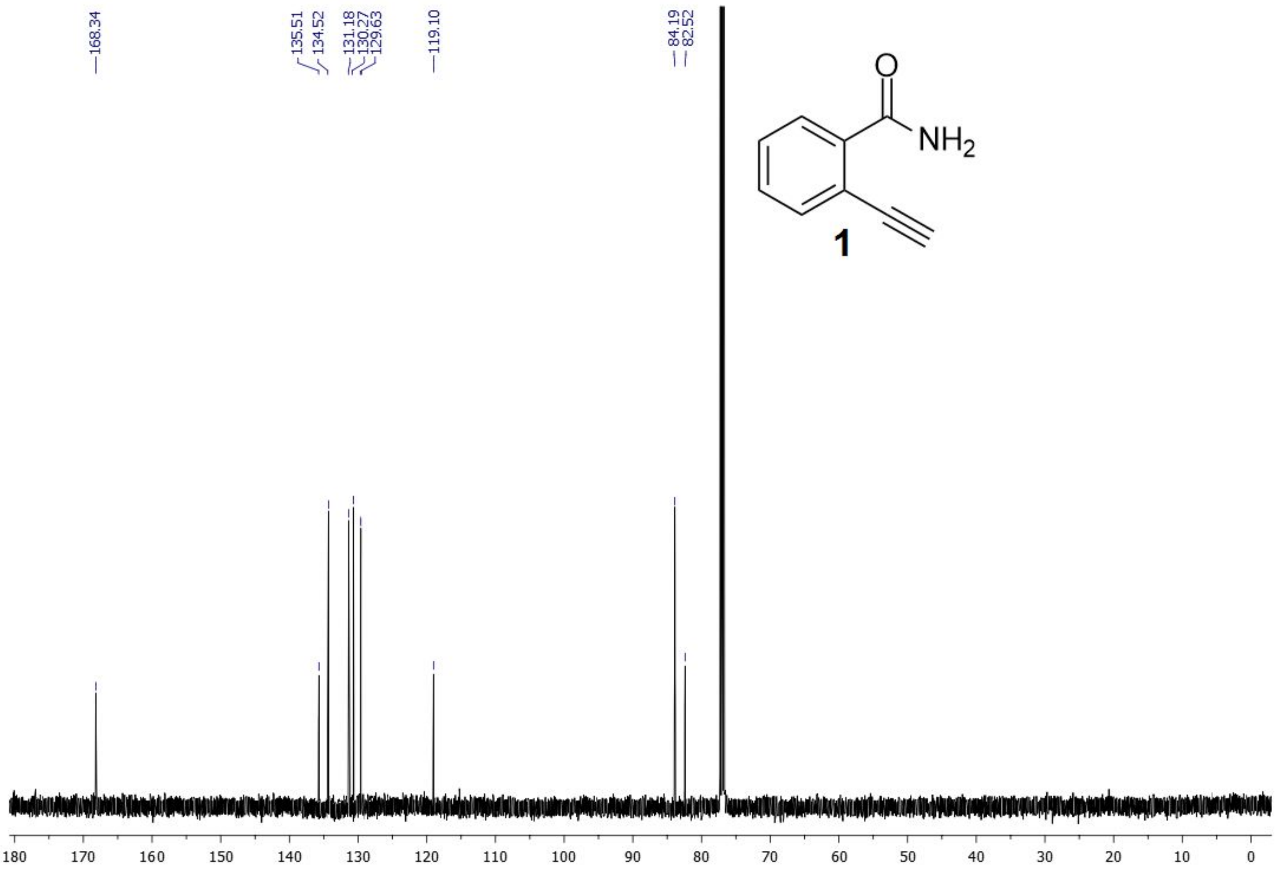

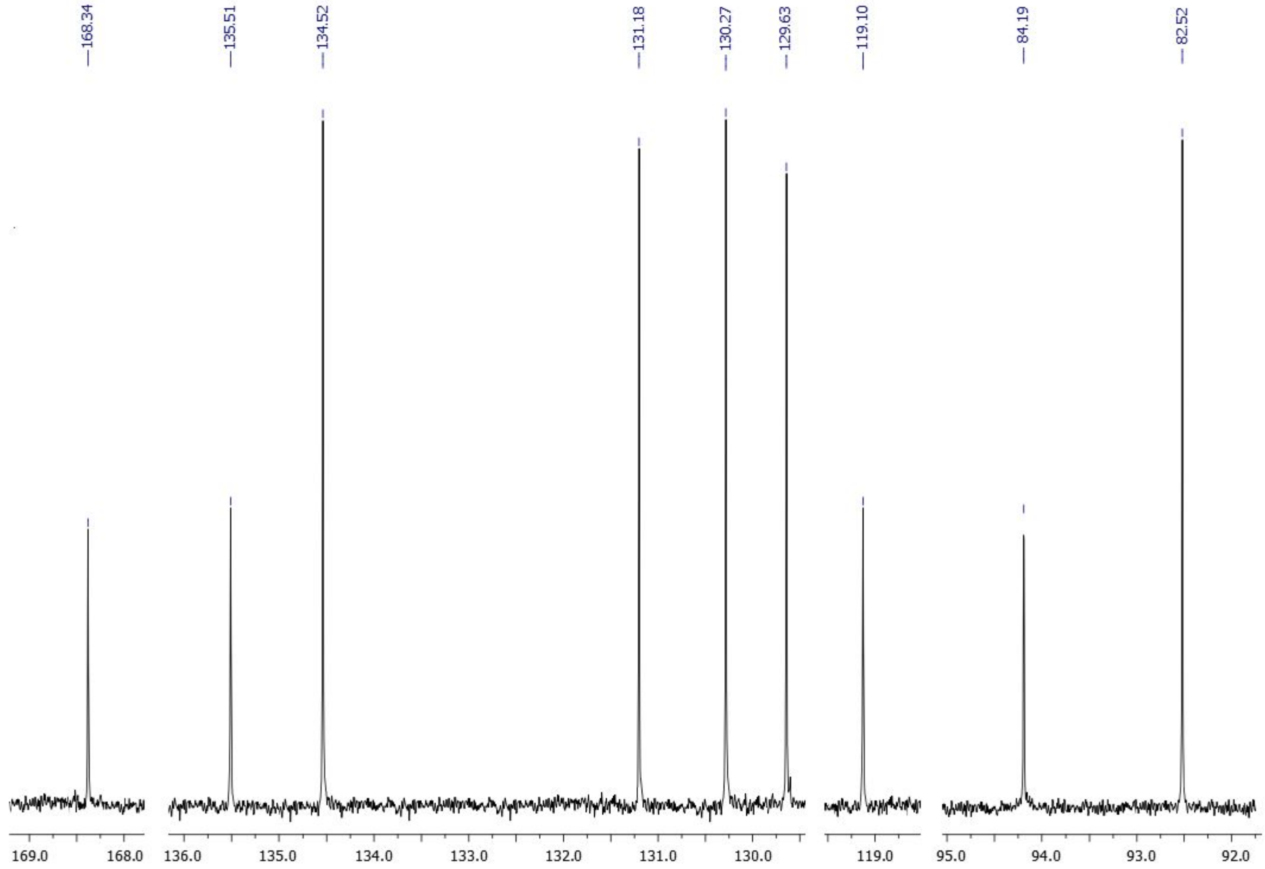

Figure S4. ^{13}C-NMR spectrum (150 MHz, $CDCl_3$) of 2-ethynylbenzamide (**1**): full scale spectrum (top) and spectrum expansions (bottom).

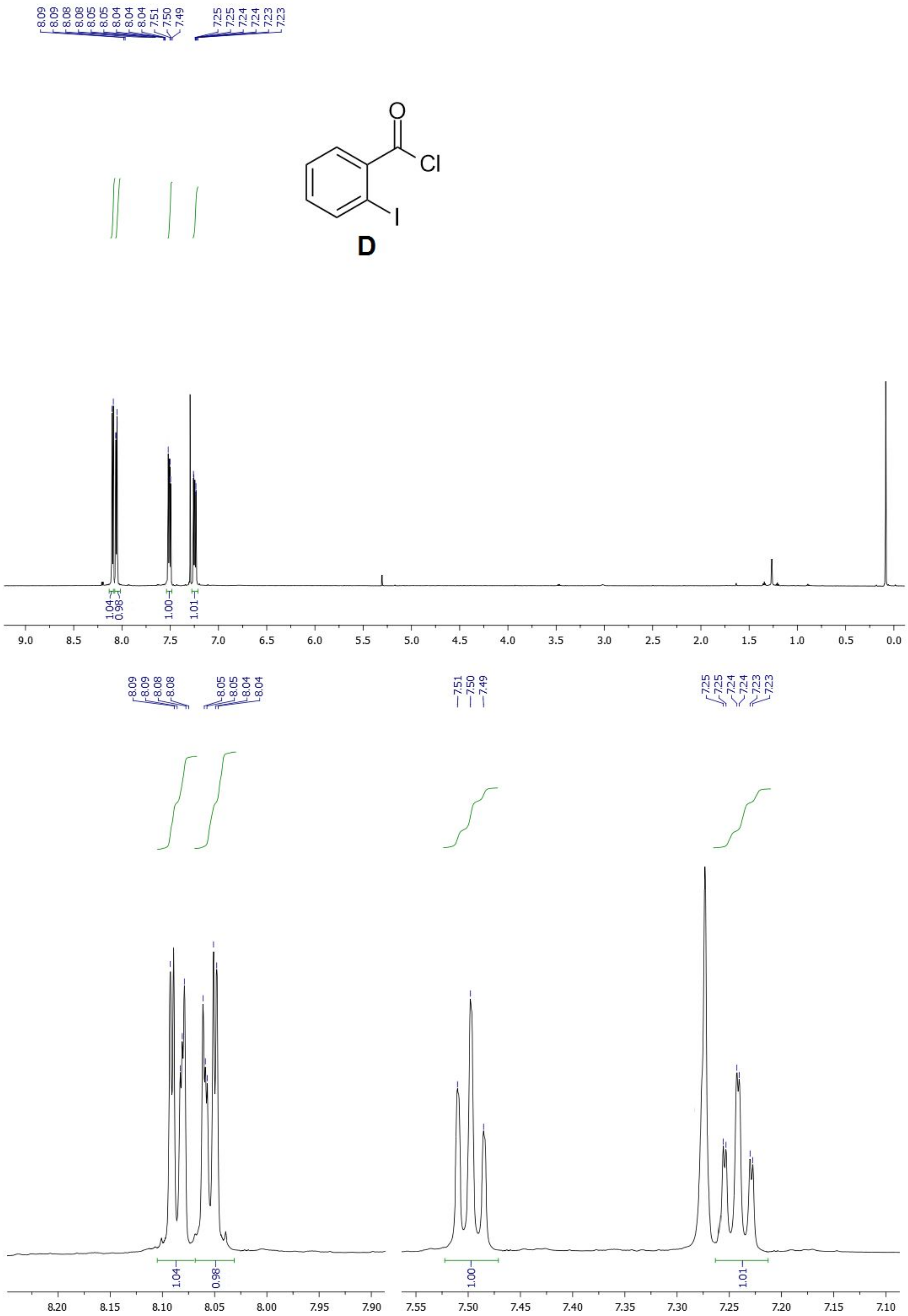

Figure S5. ^{1}H-NMR spectrum (600 MHz, $CDCl_3$) of 2-iodobenzoyl chloride (**D**): full scale spectrum (top) and spectrum expansions (bottom).

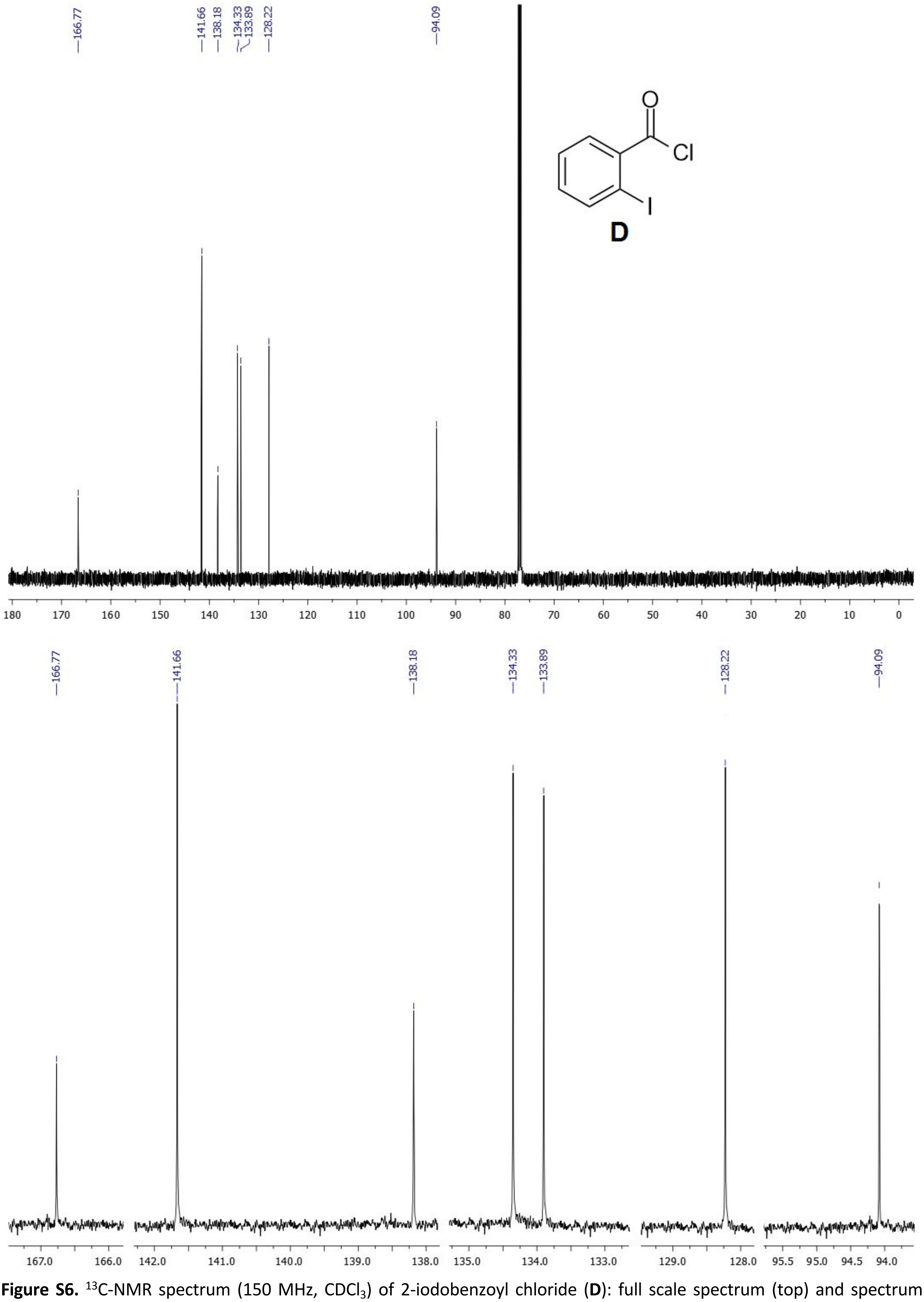

Figure S6. ^{13}C-NMR spectrum (150 MHz, $CDCl_3$) of 2-iodobenzoyl chloride (**D**): full scale spectrum (top) and spectrum expansions (bottom).

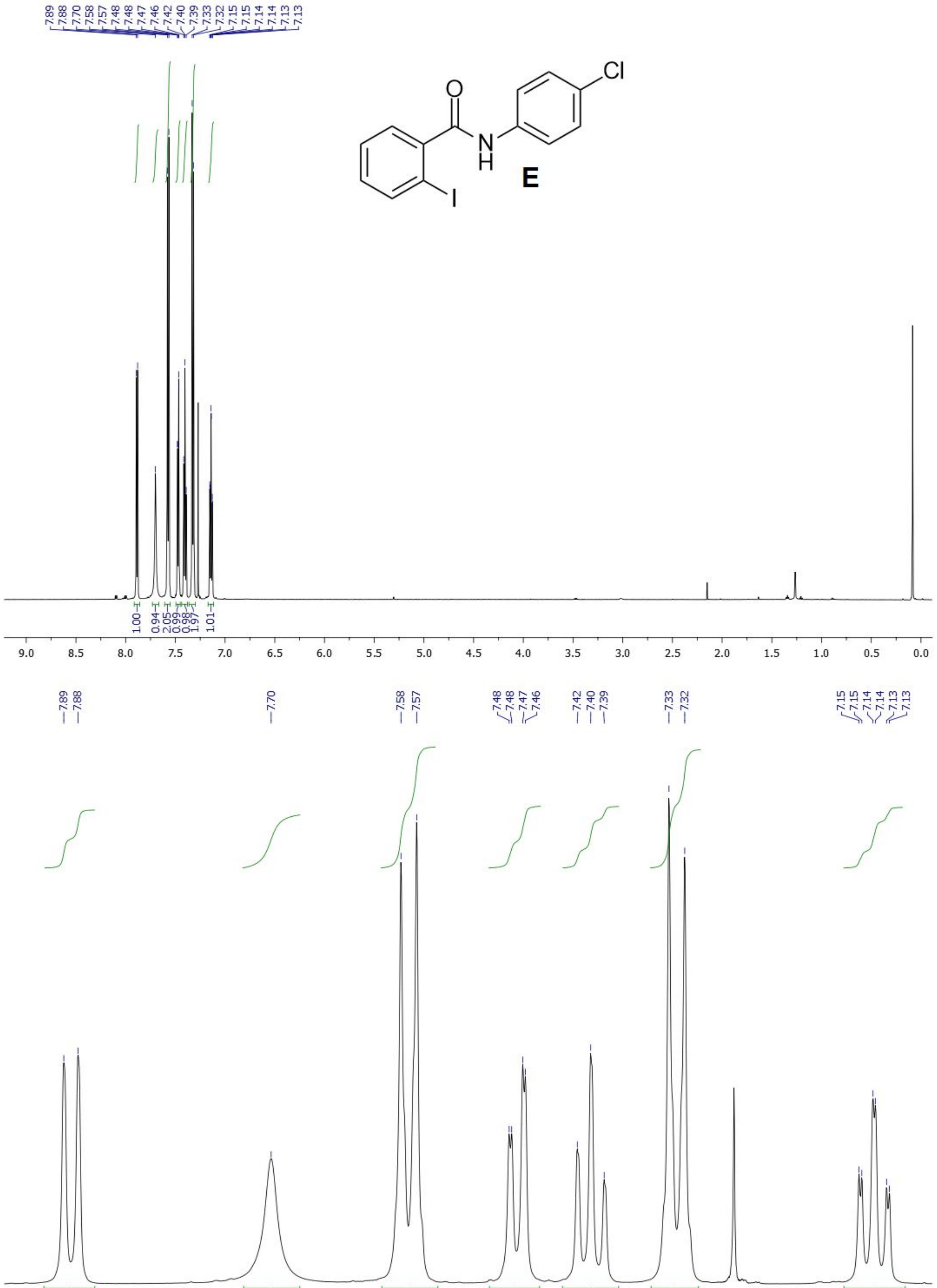

Figure S7. ^{1}H-NMR spectrum (600 MHz, $CDCl_3$) of *N*-(4-chlorophenyl)-2-iodobenzamide (**E**): full scale spectrum (top) and spectrum expansions (bottom).

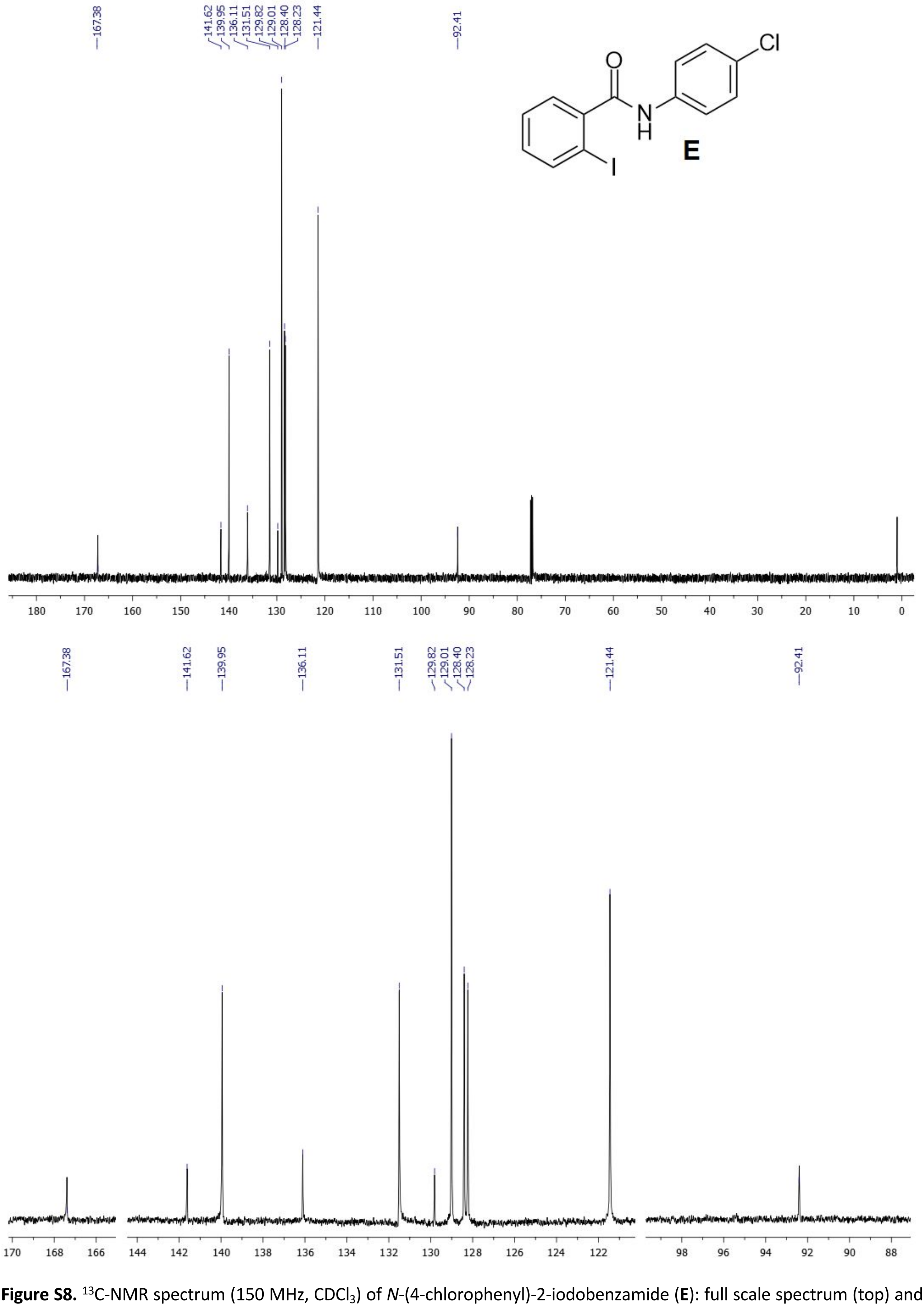

Figure S8. ^{13}C-NMR spectrum (150 MHz, $CDCl_3$) of *N*-(4-chlorophenyl)-2-iodobenzamide (**E**): full scale spectrum (top) and spectrum expansions (bottom).

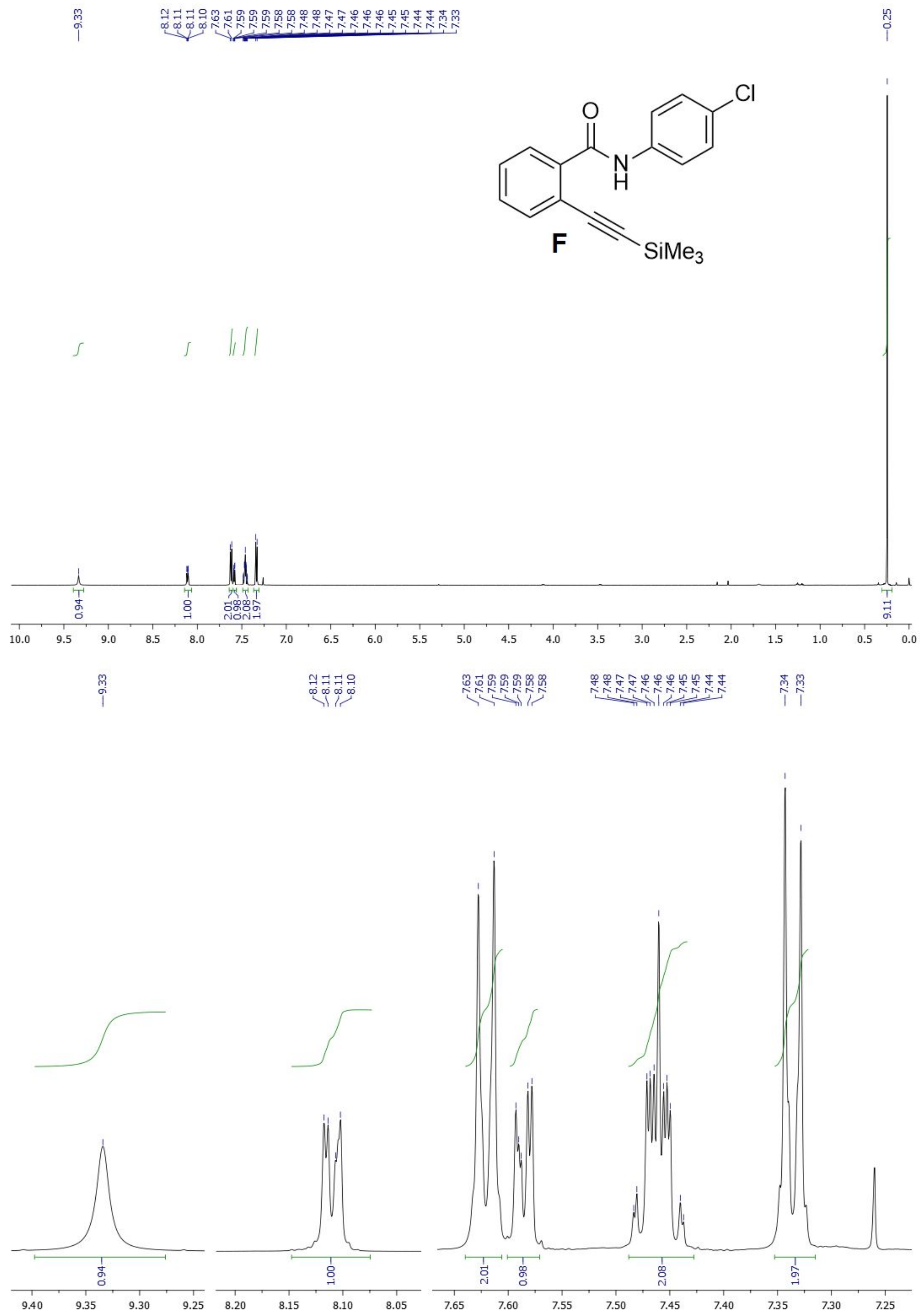

Figure S9. ^{1}H-NMR spectrum (600 MHz, $CDCl_3$) of *N*-(4-chlorophenyl)-2-((trimethylsilyl)ethynyl)benzamide (**F**): full scale spectrum (top) and spectrum expansions (bottom).

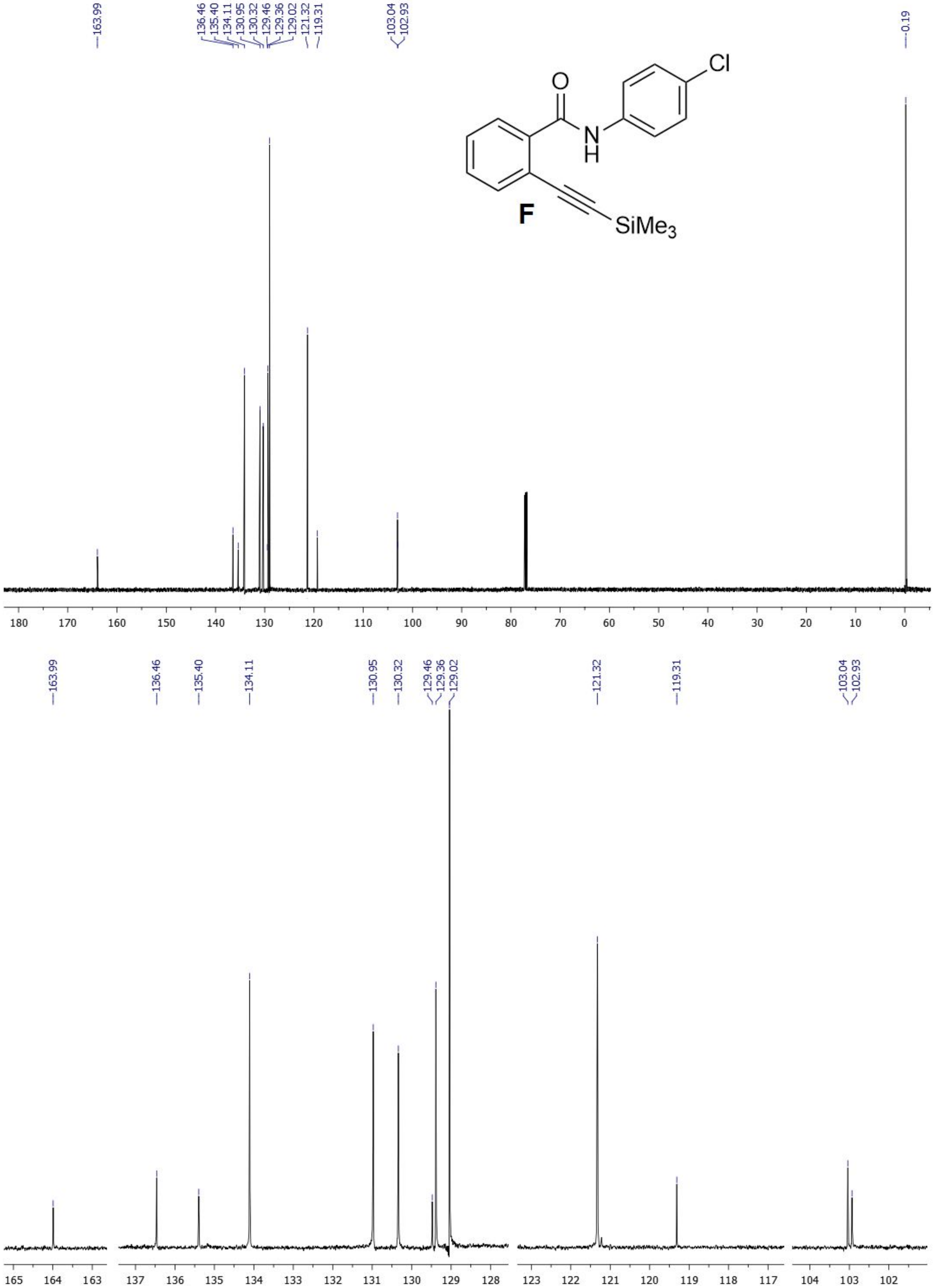

Figure S10. ^{13}C-NMR spectrum (150 MHz, $CDCl_3$) of *N*-(4-chlorophenyl)-2-((trimethylsilyl)ethynyl)benzamide (**F**): full scale spectrum (top) and spectrum expansions (bottom).

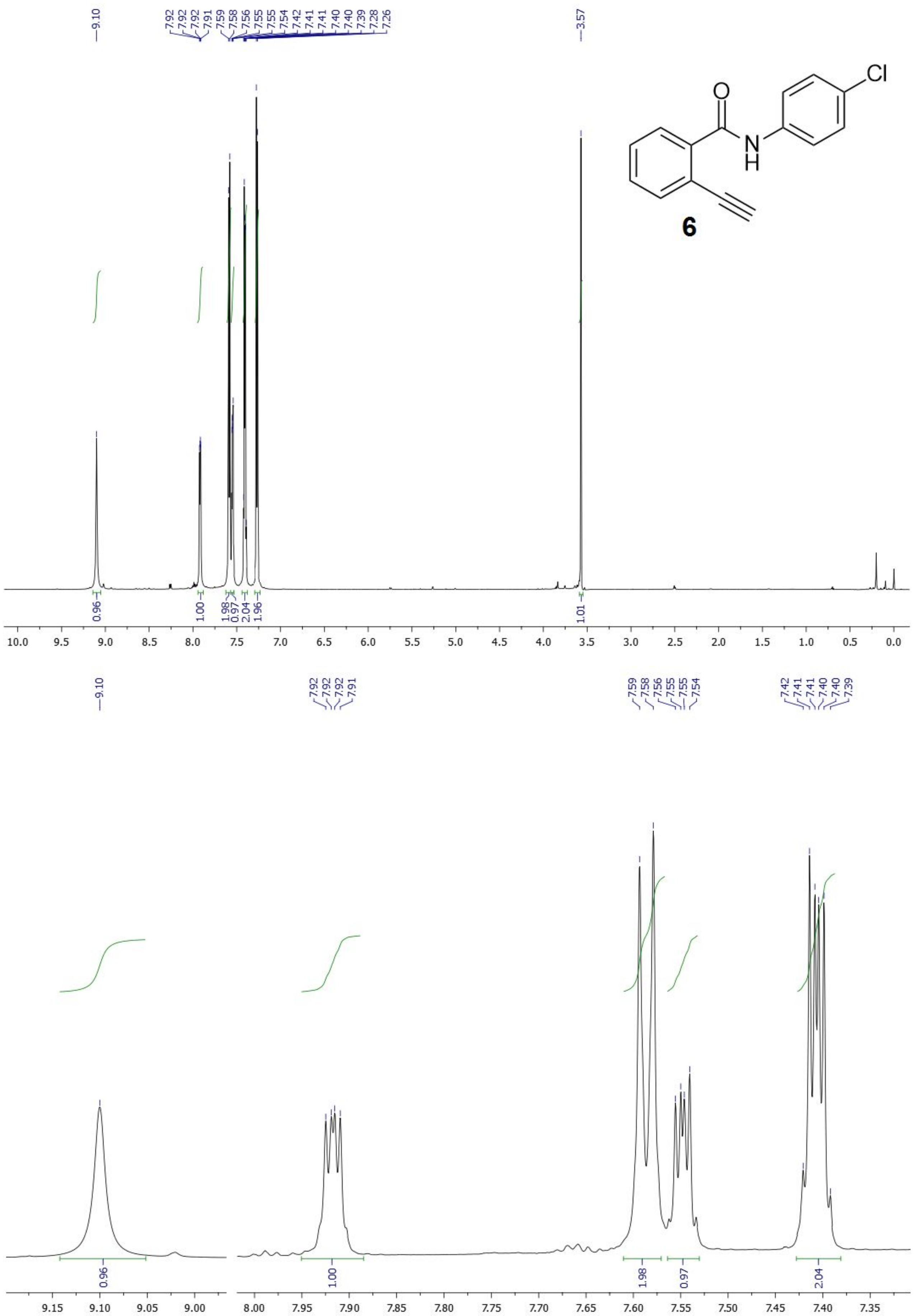

Figure S11. ^{1}H-NMR spectrum (600 MHz, $CDCl_3$) of *N*-(4-chlorophenyl)-2-ethynylbenzamide (**6**): full scale spectrum (top) and spectrum expansions (bottom).

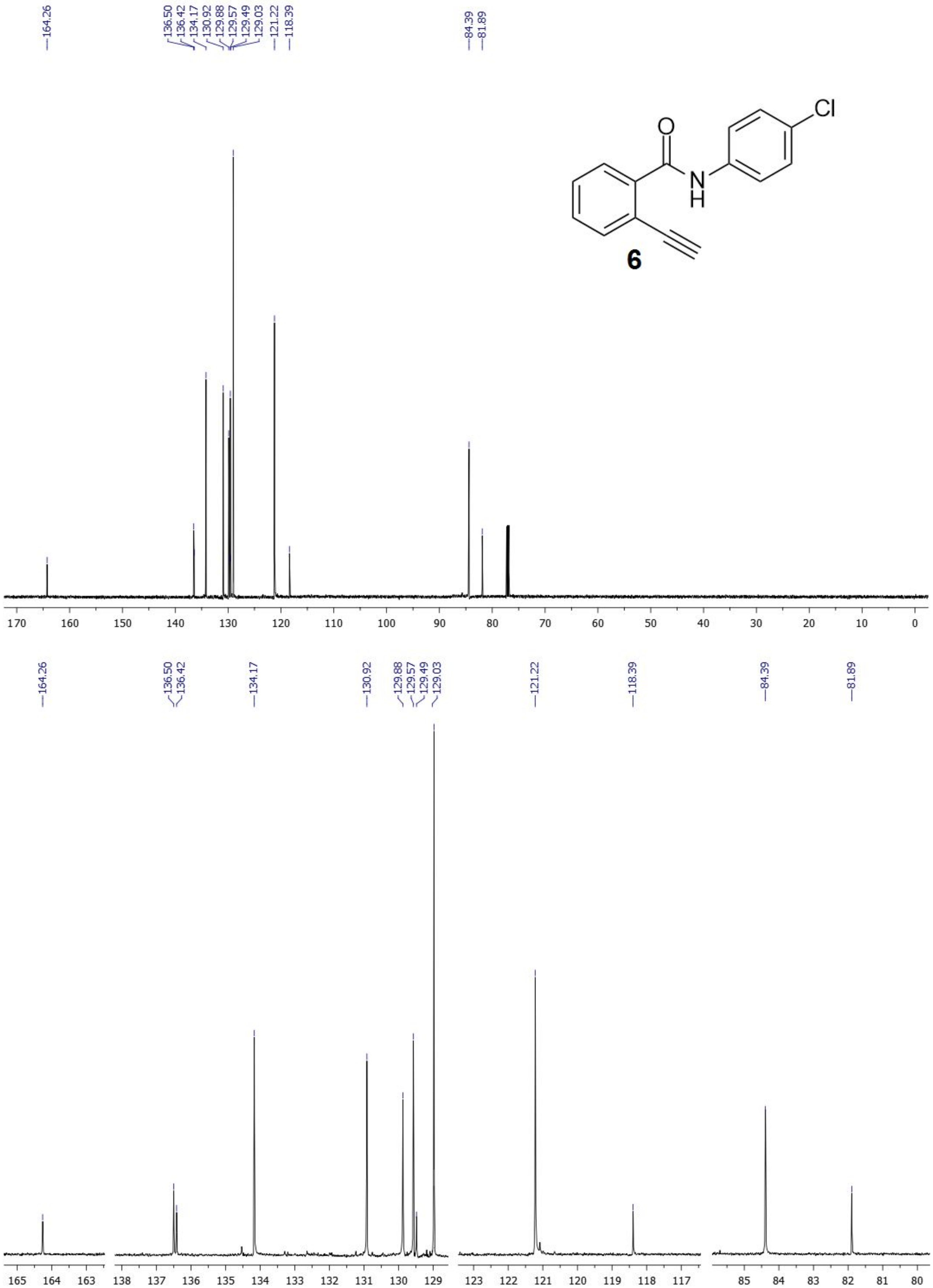

Figure S12. ^{13}C-NMR spectrum (150 MHz, $CDCl_3$) of *N*-(4-chlorophenyl)-2-ethynylbenzamide (**6**): full scale spectrum (top) and spectrum expansions (bottom).

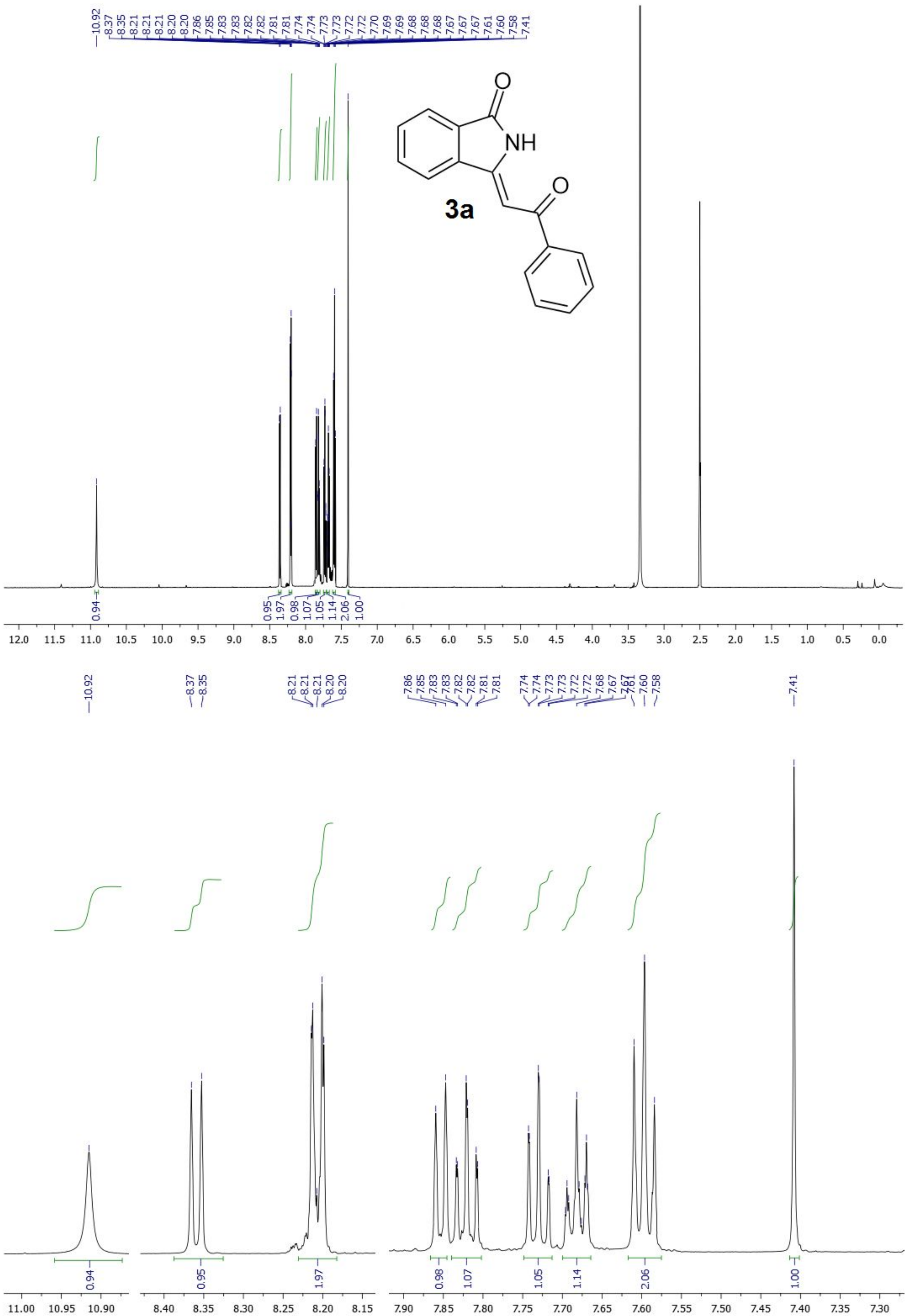

Figure S13. ^{1}H-NMR spectrum (600 MHz, $CDCl_3$) of (*Z*)-3-(2-oxo-2-phenylethylidene)isoindolin-1-one (**3a**): full scale spectrum (top) and spectrum expansions (bottom).

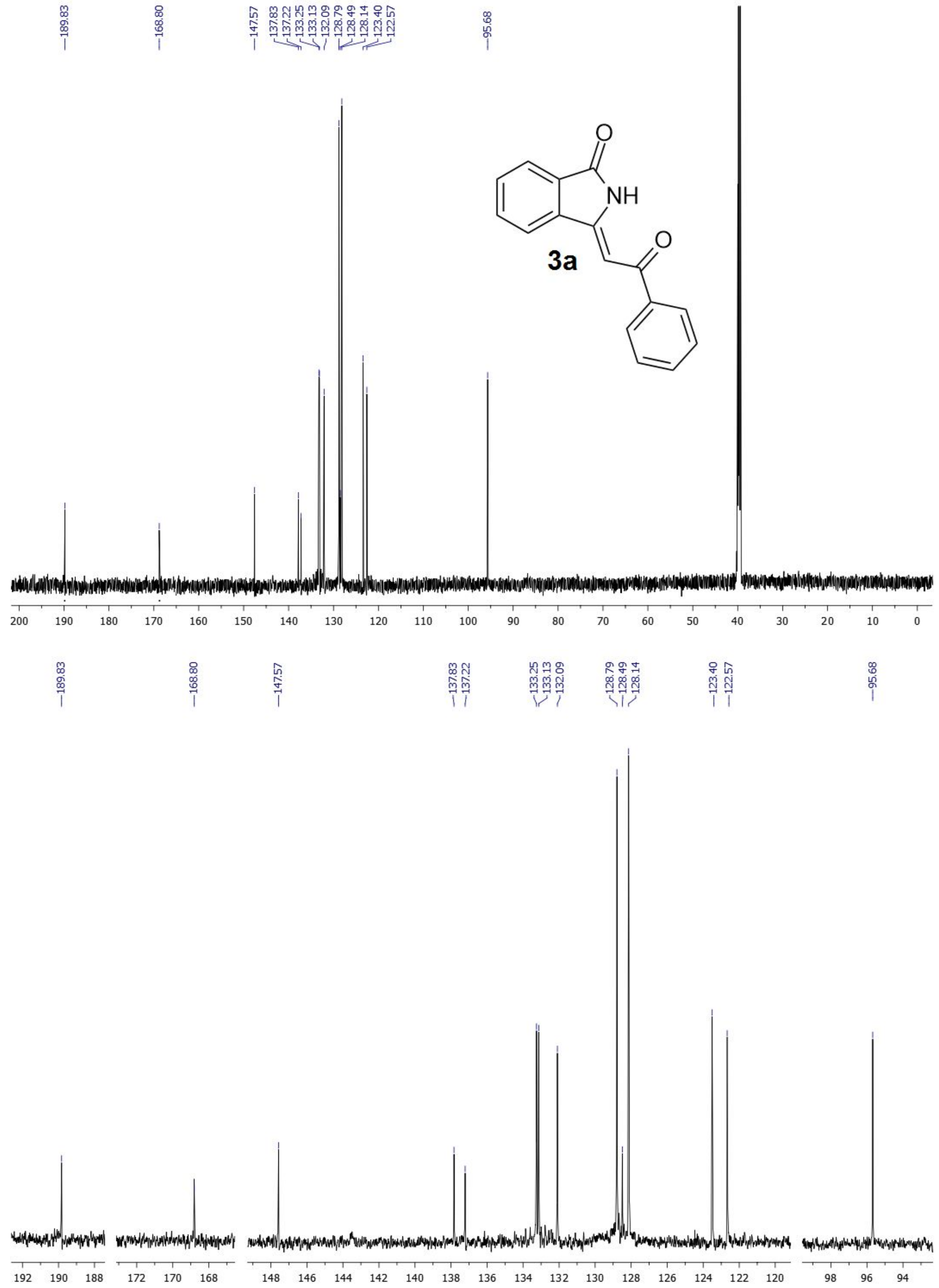

Figure S14. ^{13}C-NMR spectrum (150 MHz, $CDCl_3$) of (*Z*)-3-(2-oxo-2-phenylethylidene)isoindolin-1-one (**3a**): full scale spectrum (top) and spectrum expansions (bottom).

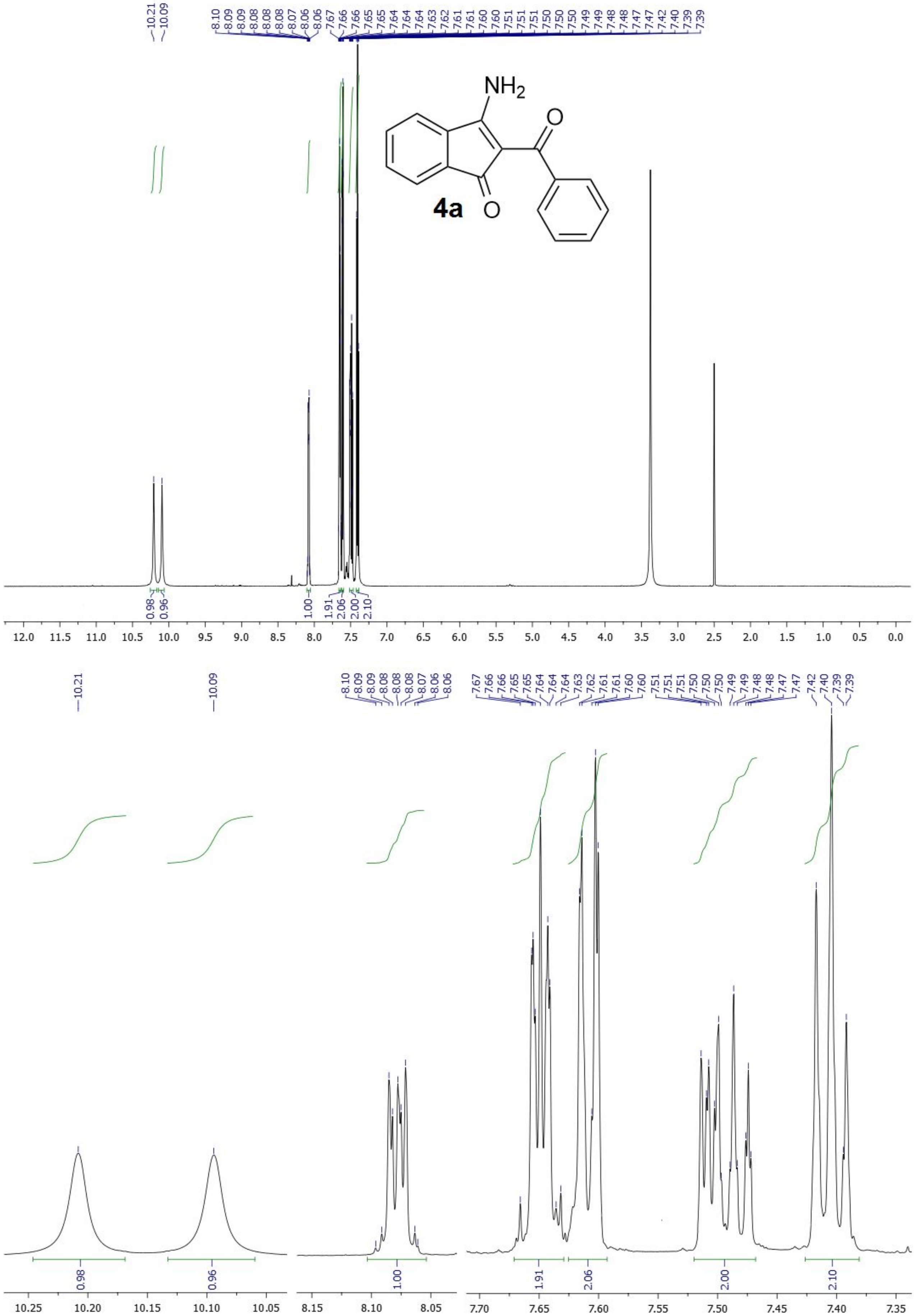

Figure S15. ^{1}H-NMR spectrum (600 MHz, $CDCl_3$) of 3-amino-2-benzoyl-1*H*-inden-1-one (**4a**): full scale spectrum (top) and spectrum expansions (bottom).

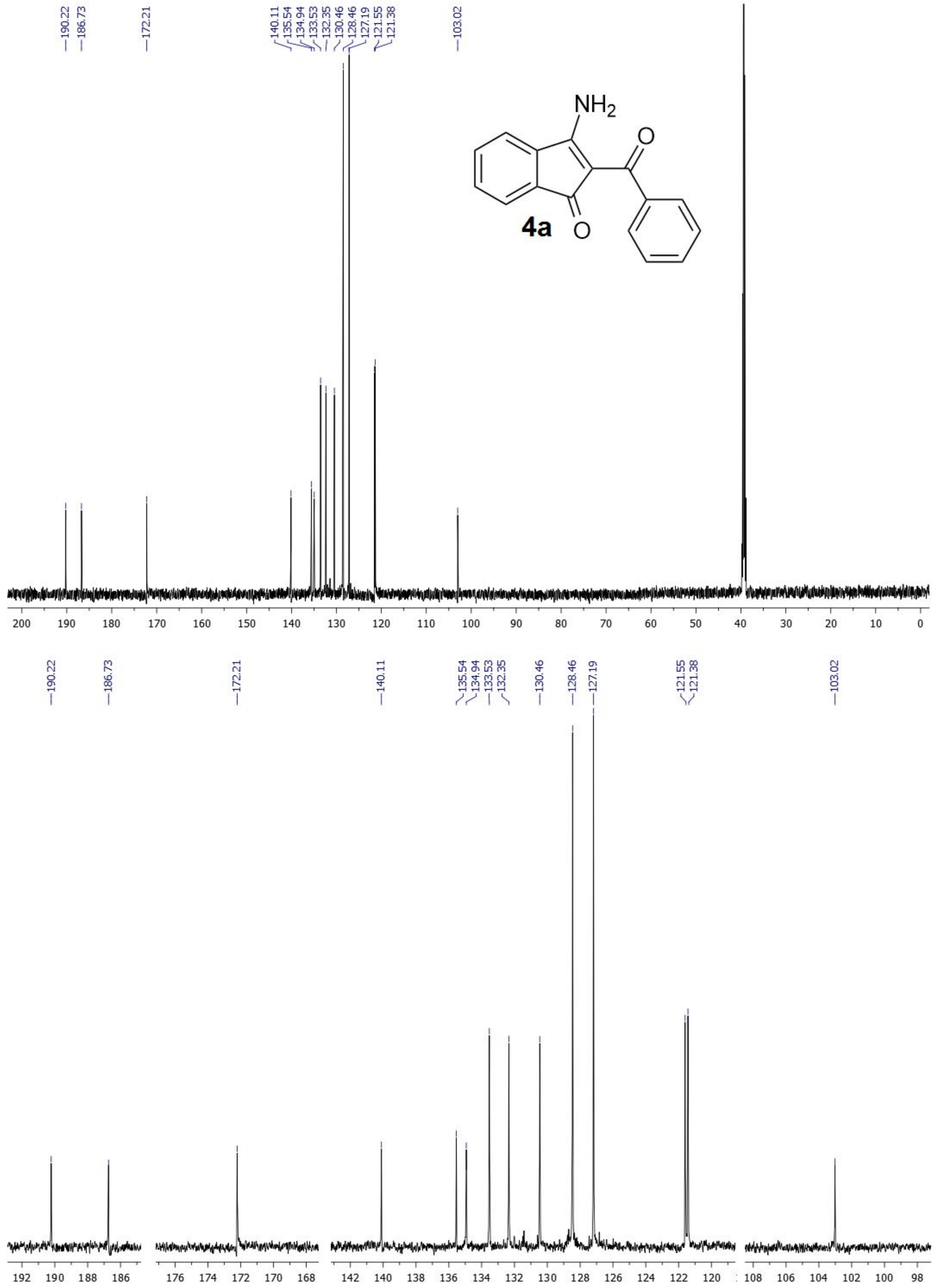

Figure S16. ^{13}C-NMR spectrum (150 MHz, $CDCl_3$) of 3-amino-2-benzoyl-1*H*-inden-1-one (**4a**): full scale spectrum (top) and spectrum expansions (bottom).

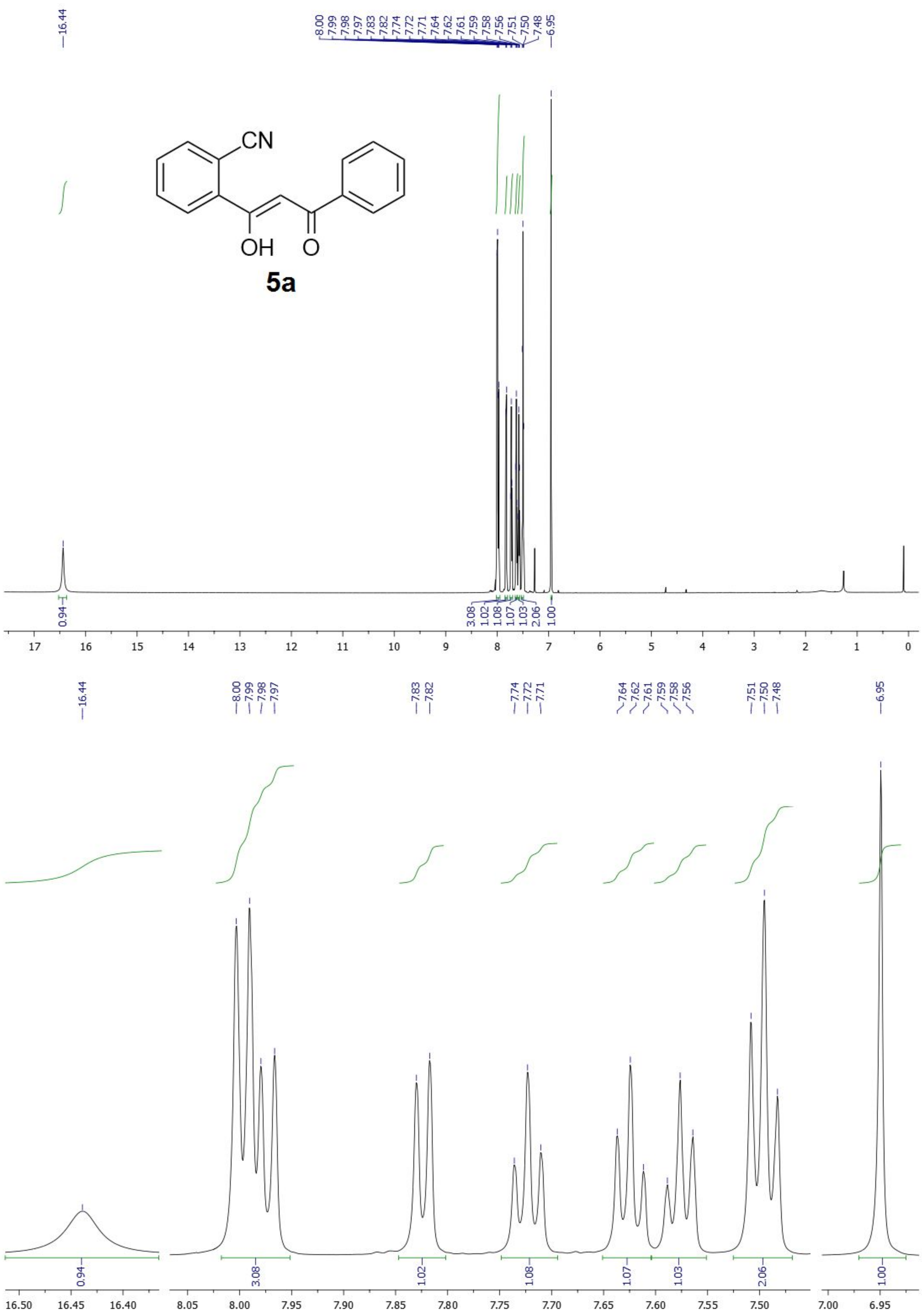

Figure S17. ^{1}H-NMR spectrum (600 MHz, $CDCl_3$) of (*Z*)-2-(1-hydroxy-3-oxo-3-phenylprop-1-en-1-yl)benzonitrile (**5a**): full scale spectrum (top) and spectrum expansions (bottom).

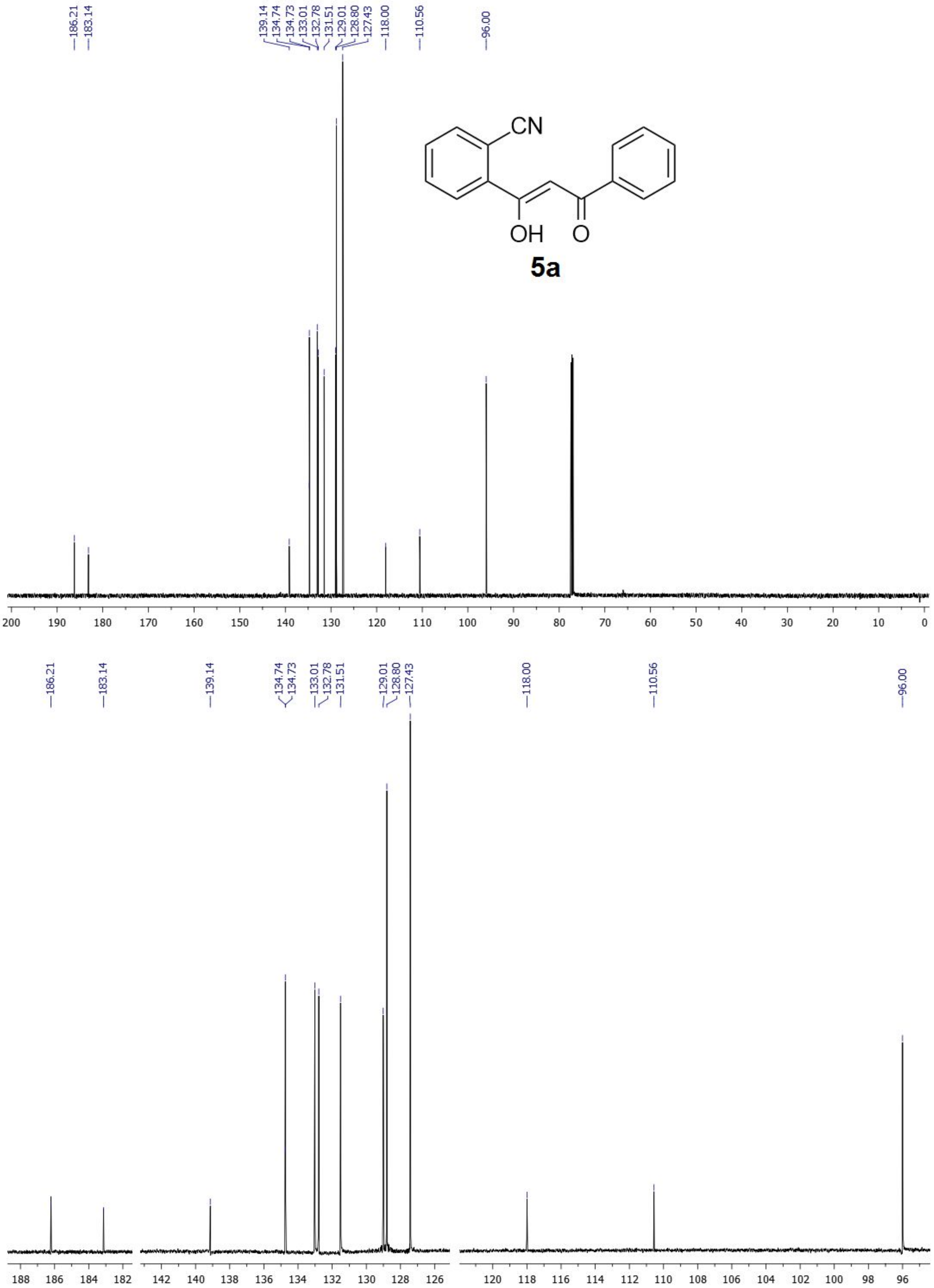

Figure S18. ^{13}C-NMR spectrum (150 MHz, $CDCl_3$) of (*Z*)-2-(1-hydroxy-3-oxo-3-phenylprop-1-en-1-yl)benzonitrile (**5a**): full scale spectrum (top) and spectrum expansions (bottom).

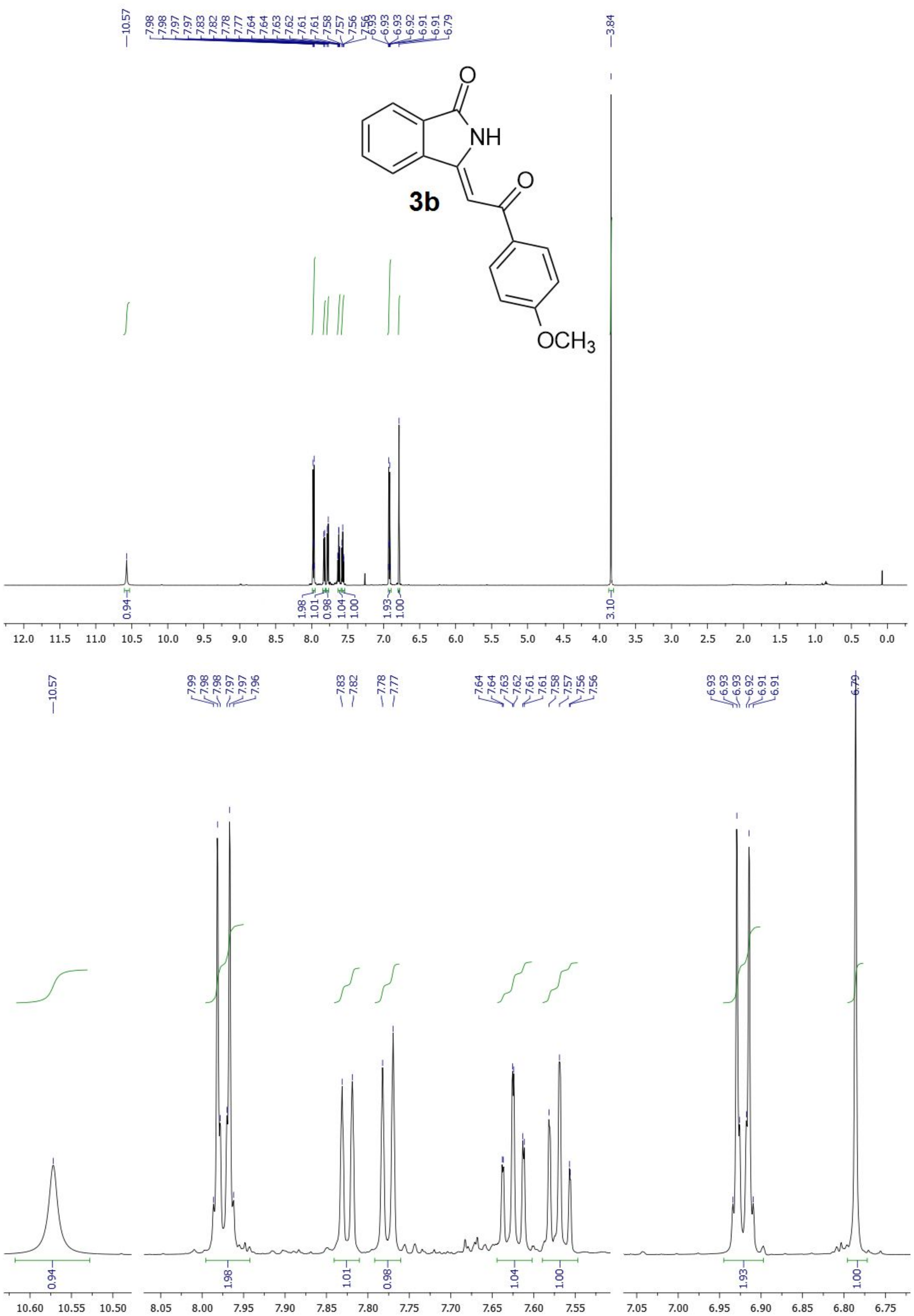

Figure S19. ^{1}H-NMR spectrum (600 MHz, $CDCl_3$) of (*Z*)-3-(2-(4-methoxyphenyl)-2-oxoethylidene)isoindolin-1-one (**3b**): full scale spectrum (top) and spectrum expansions (bottom).

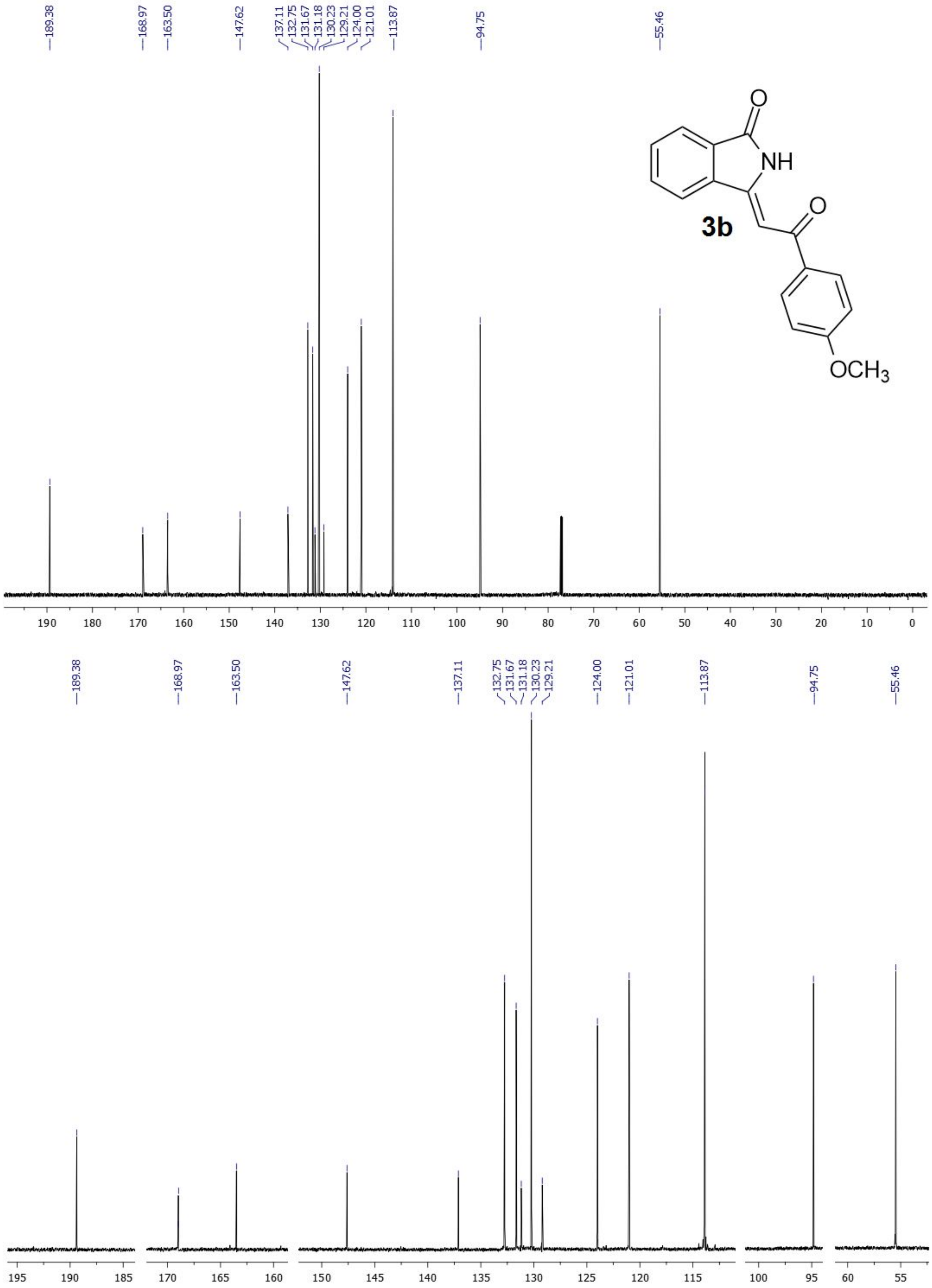

Figure S20. ^{13}C-NMR spectrum (150 MHz, $CDCl_3$) of (*Z*)-3-(2-(4-methoxyphenyl)-2-oxoethylidene)isoindolin-1-one (**3b**): full scale spectrum (top) and spectrum expansions (bottom).

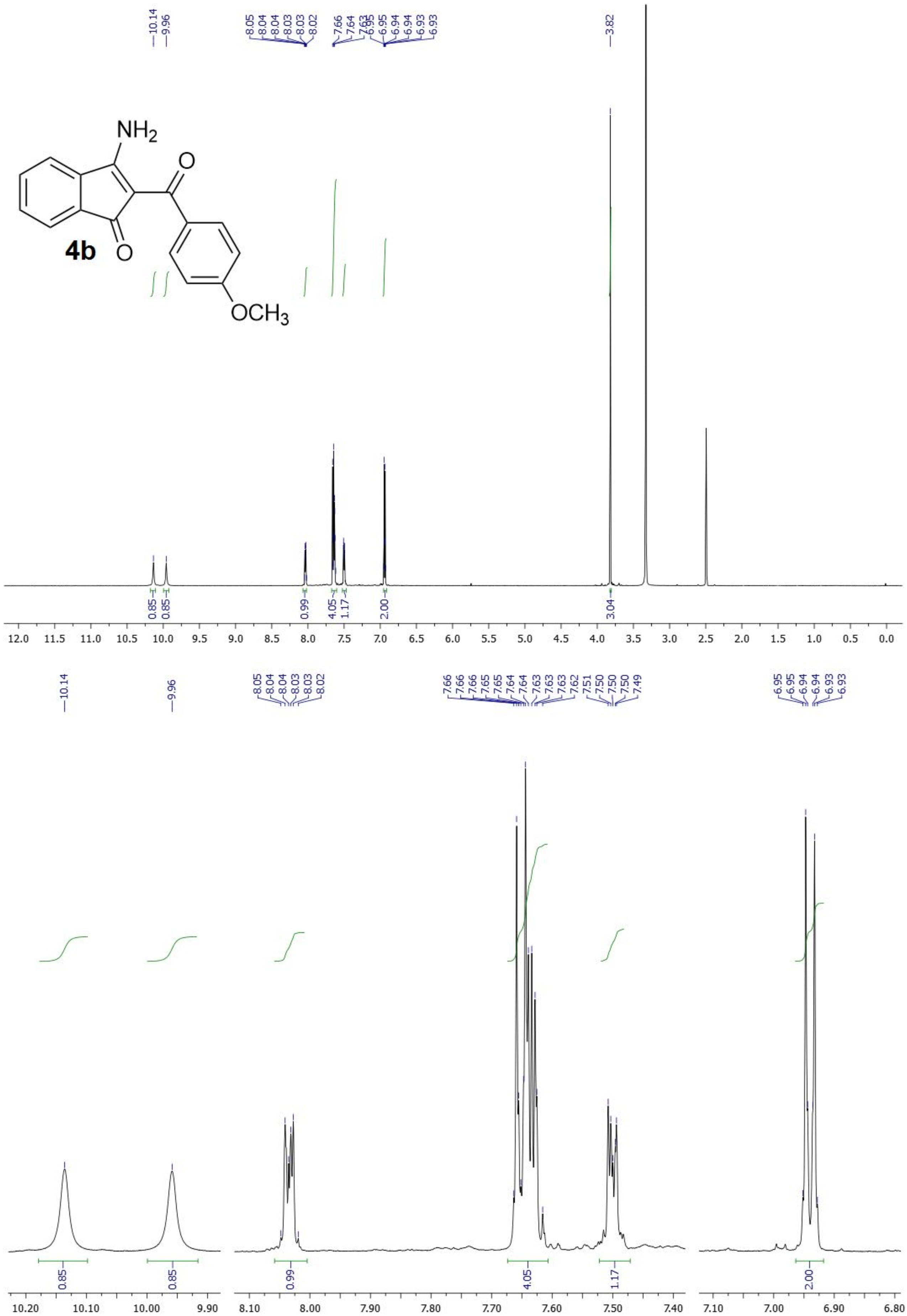

Figure S21. ^{1}H-NMR spectrum (600 MHz, $CDCl_3$) of 3-amino-2-(4-methoxybenzoyl)-1*H*-inden-1-one (**4b**): full scale spectrum (top) and spectrum expansions (bottom).

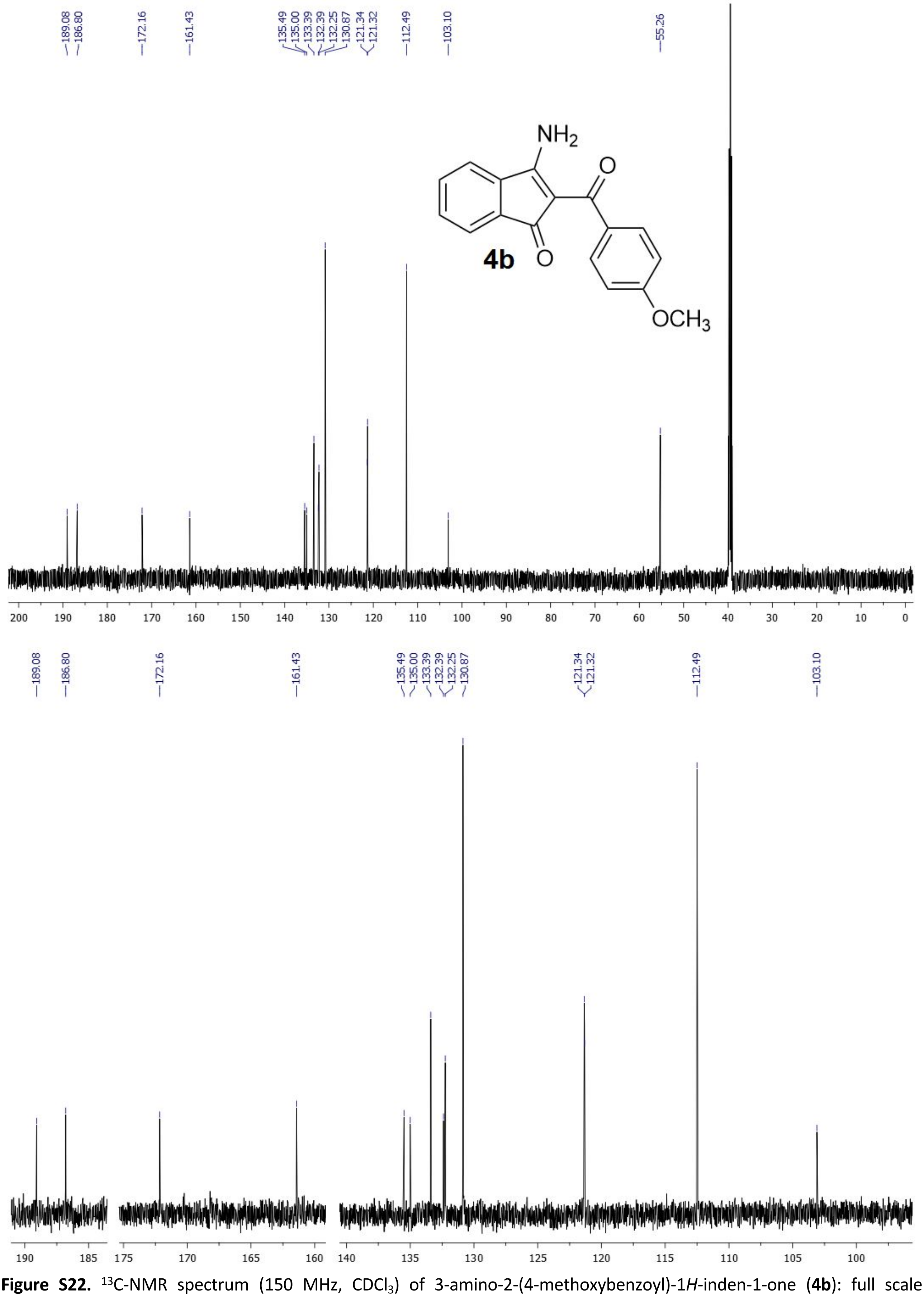

Figure S22. ^{13}C-NMR spectrum (150 MHz, $CDCl_3$) of 3-amino-2-(4-methoxybenzoyl)-1*H*-inden-1-one (**4b**): full scale spectrum (top) and spectrum expansions (bottom).

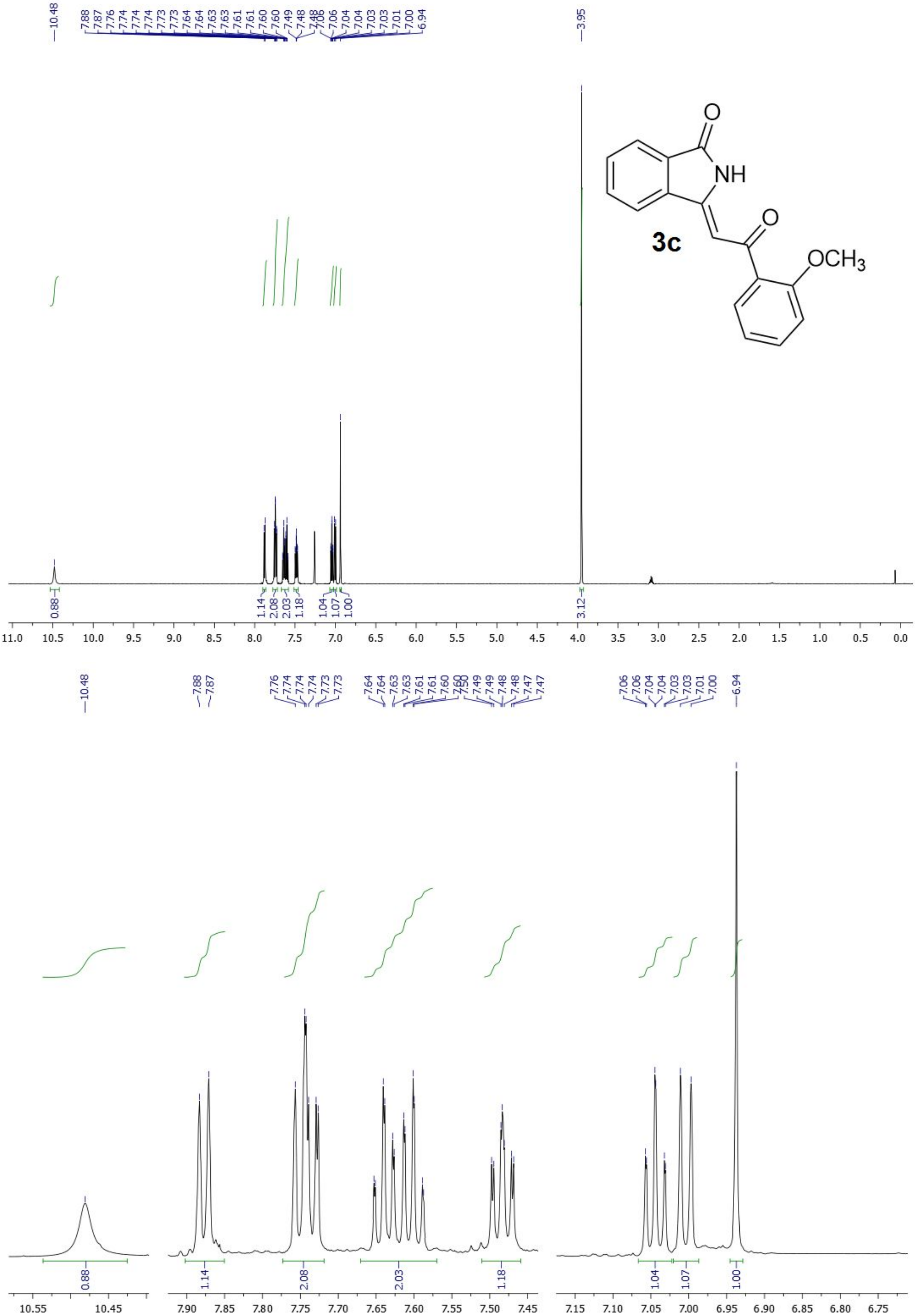

Figure S23. ^{1}H-NMR spectrum (600 MHz, $CDCl_3$) of (*Z*)-3-(2-(2-methoxyphenyl)-2-oxoethylidene)isoindolin-1-one (**3c**): full scale spectrum (top) and spectrum expansions (bottom).

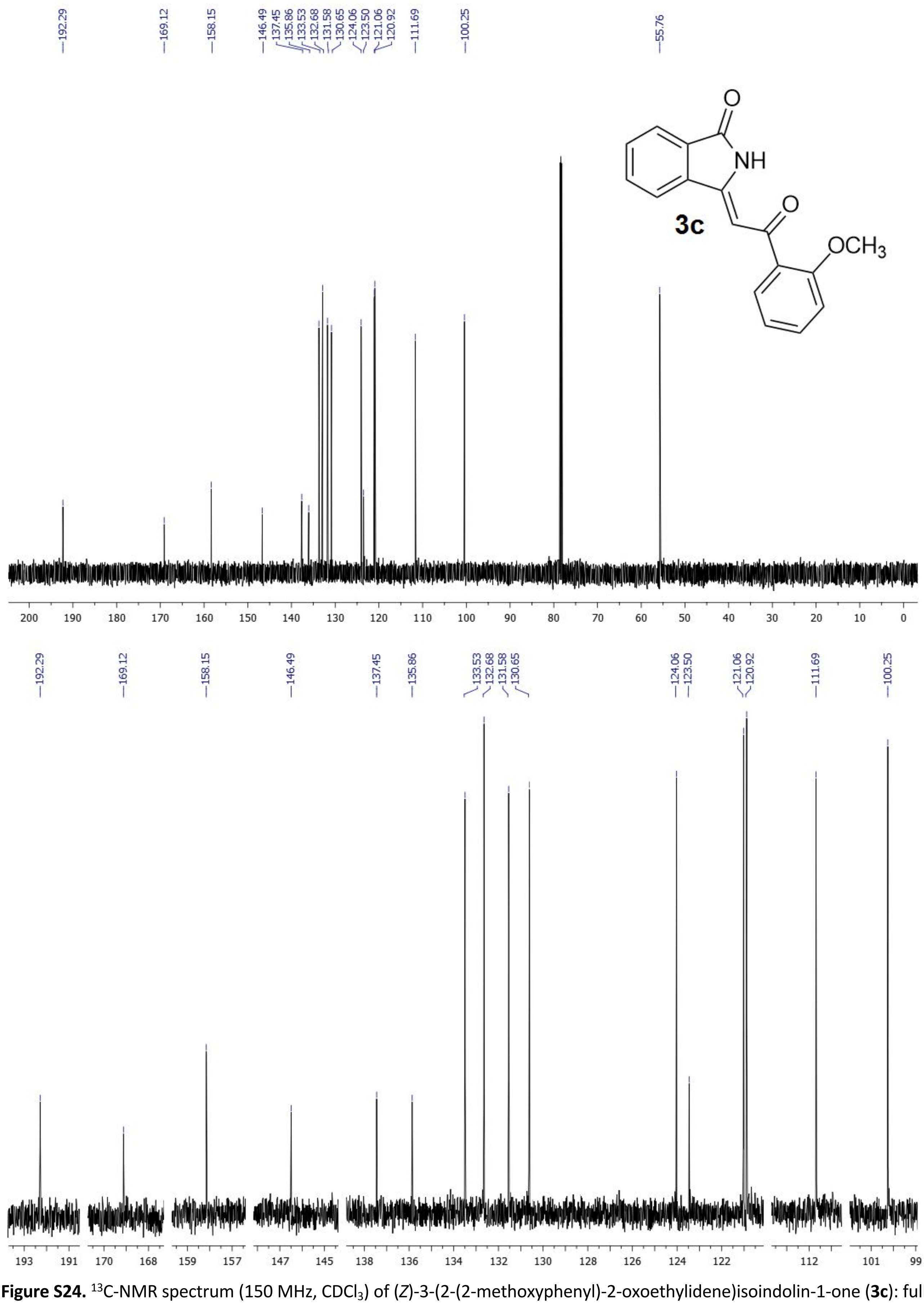

Figure S24. ^{13}C-NMR spectrum (150 MHz, $CDCl_3$) of (*Z*)-3-(2-(2-methoxyphenyl)-2-oxoethylidene)isoindolin-1-one (**3c**): full scale spectrum (top) and spectrum expansions (bottom).

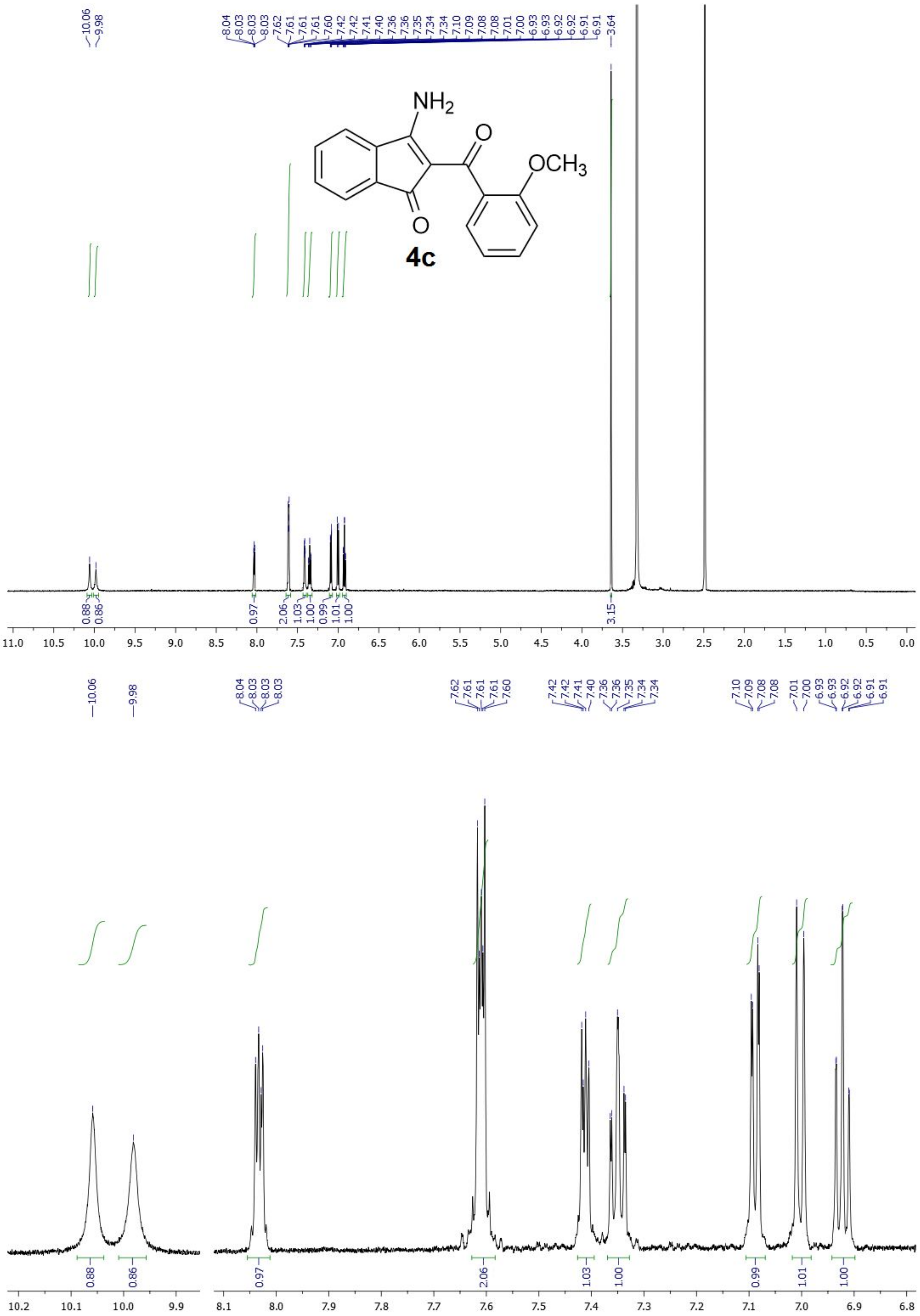

Figure S25. ^{1}H-NMR spectrum (600 MHz, $CDCl_3$) of 3-amino-2-(2-methoxybenzoyl)-1*H*-inden-1-one (**4c**): full scale spectrum (top) and spectrum expansions (bottom).

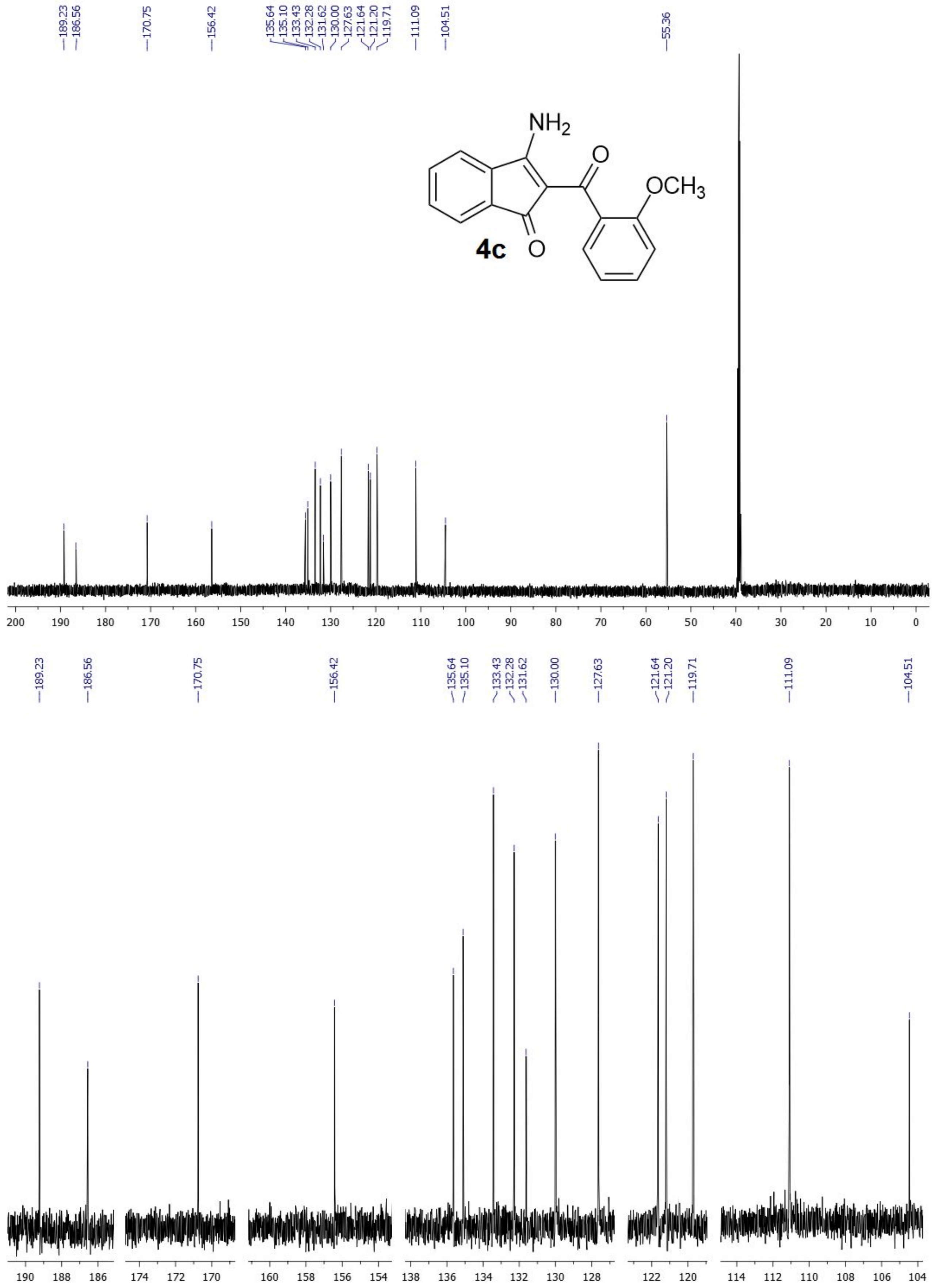

Figure S26. ^{13}C-NMR spectrum (150 MHz, $CDCl_3$) of 3-amino-2-(2-methoxybenzoyl)-1*H*-inden-1-one (**4c**): full scale spectrum (top) and spectrum expansions (bottom).

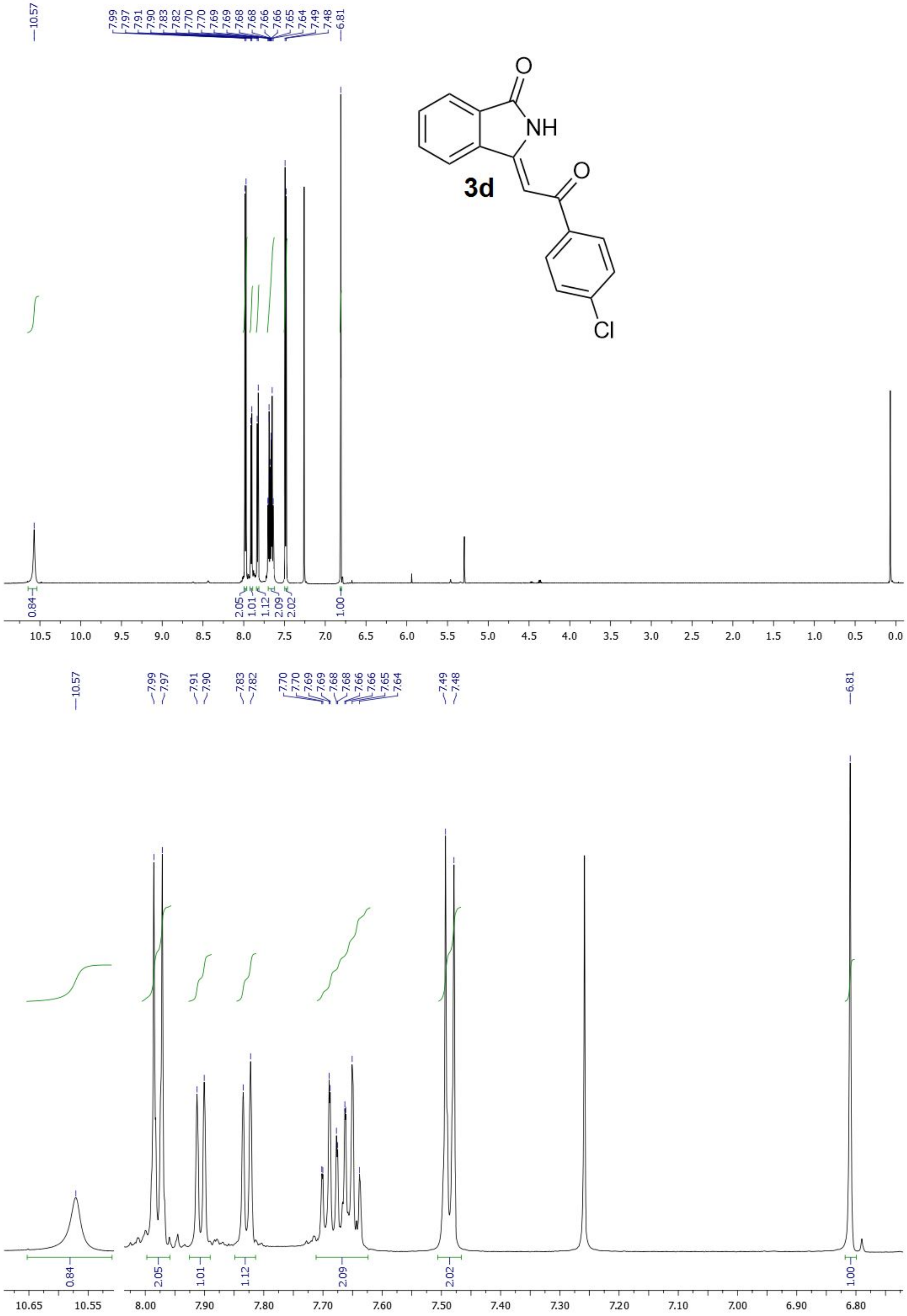

Figure S27. ¹H-NMR spectrum (600 MHz, $CDCl_3$) of (*Z*)-3-(2-(4-chlorophenyl)-2-oxoethylidene)isoindolin-1-one (**3d**): full scale spectrum (top) and spectrum expansions (bottom).

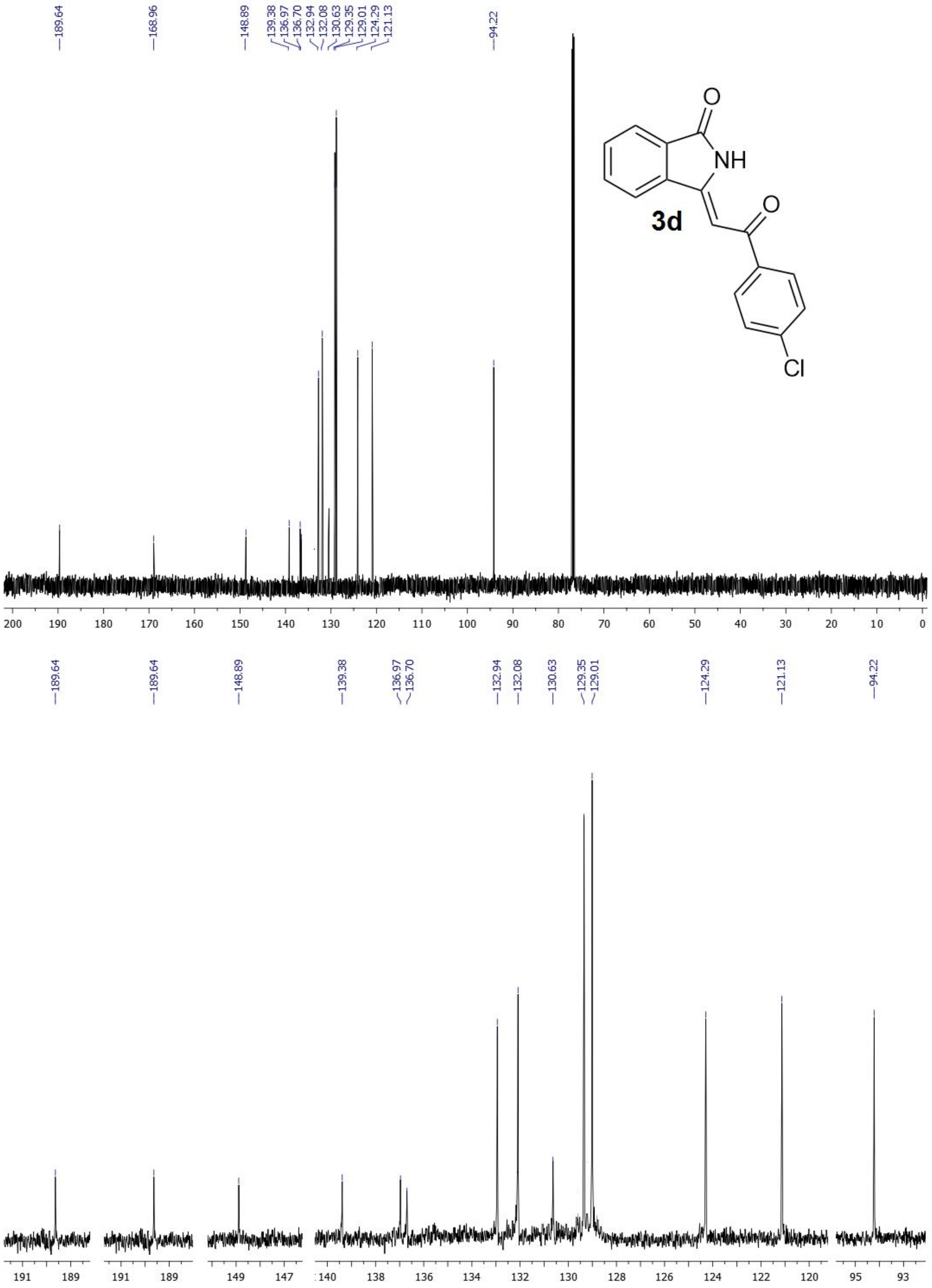

Figure S28. ^{13}C-NMR spectrum (150 MHz, $CDCl_3$) of (*Z*)-3-(2-(4-chlorophenyl)-2-oxoethylidene)isoindolin-1-one (**3d**): full scale spectrum (top) and spectrum expansions (bottom).

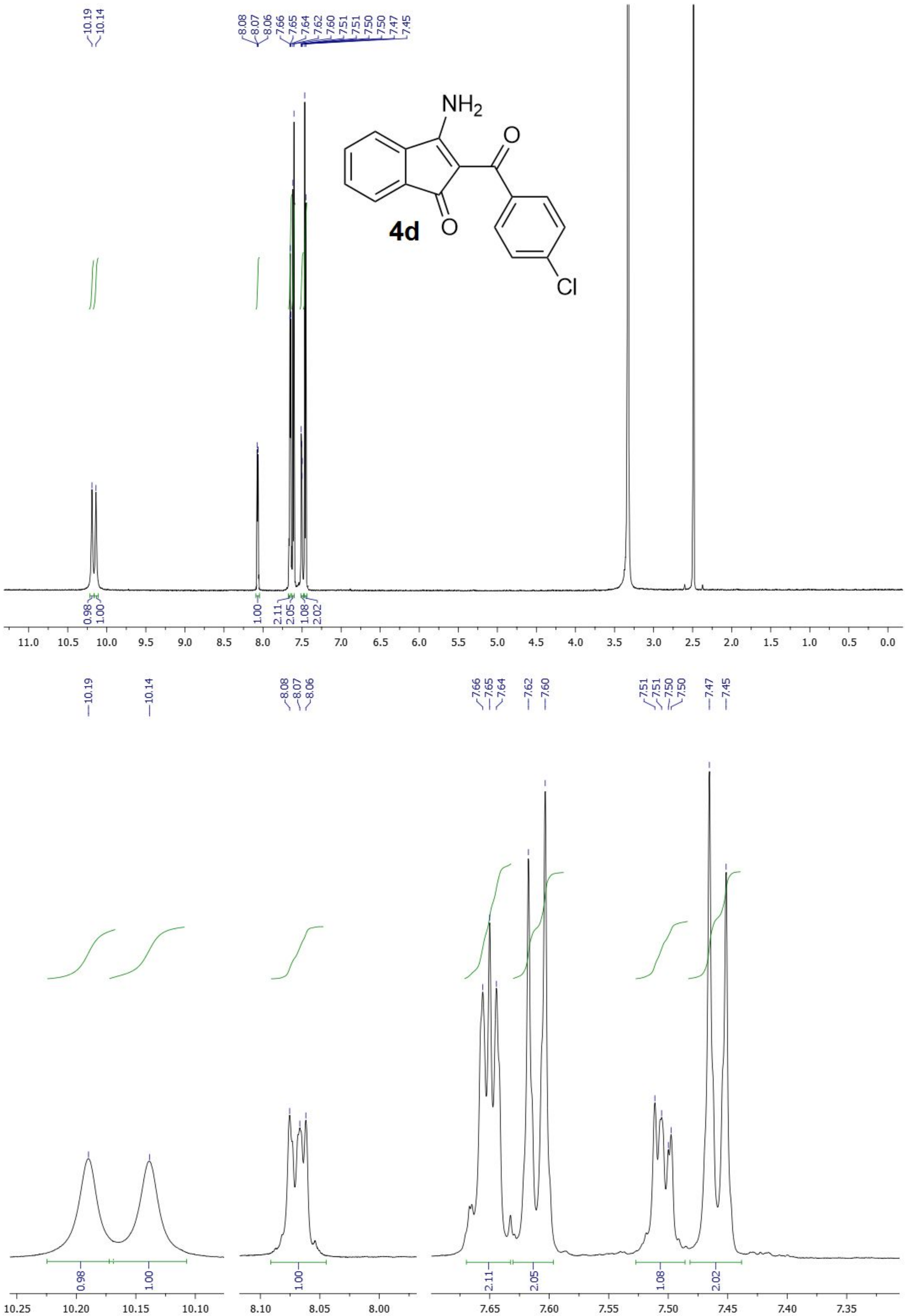

Figure S29. ^{1}H-NMR spectrum (600 MHz, $CDCl_3$) of 3-amino-2-(4-chlorobenzoyl)-1*H*-inden-1-one (**4d**): full scale spectrum (top) and spectrum expansions (bottom).

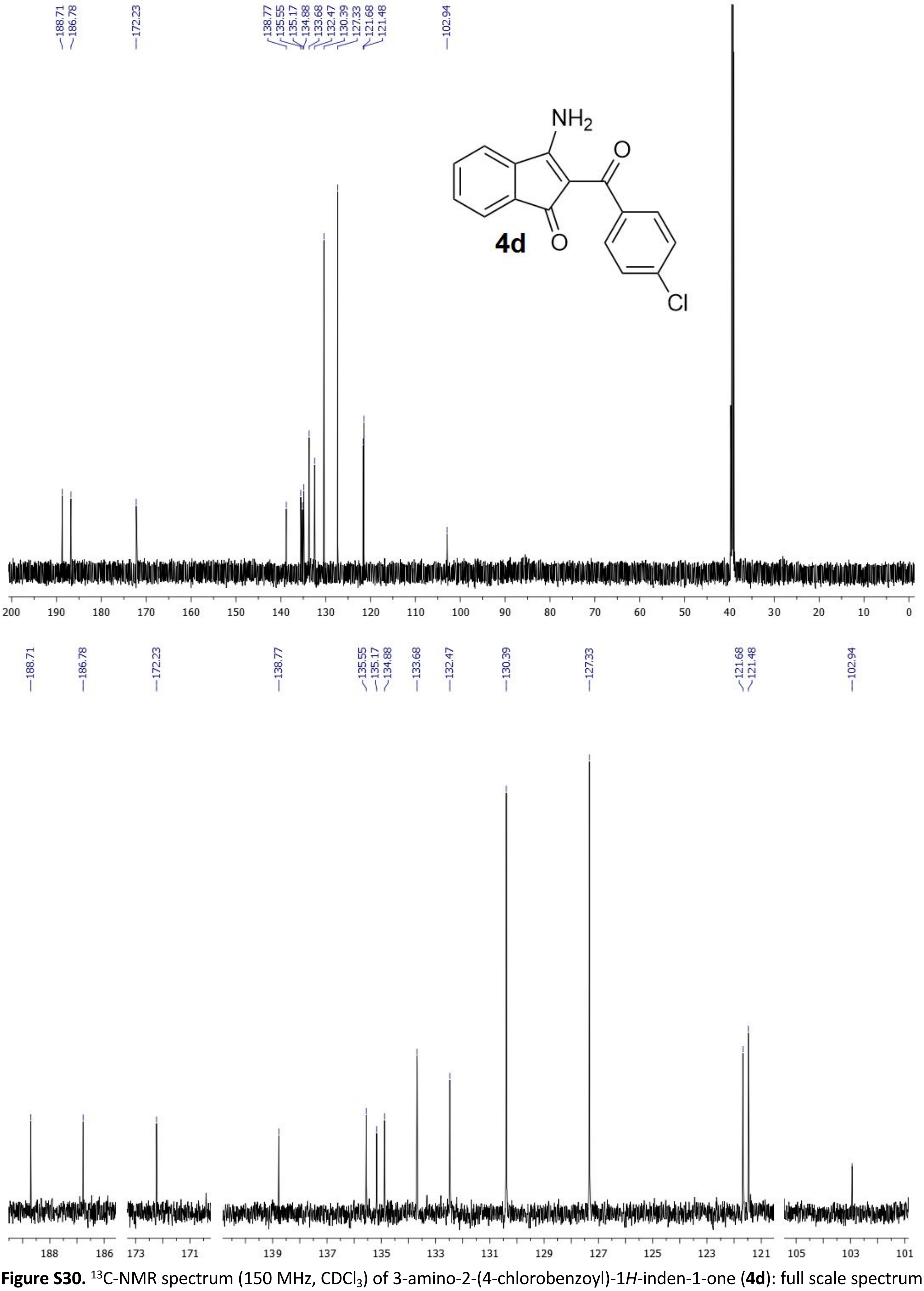

Figure S30. ^{13}C-NMR spectrum (150 MHz, $CDCl_3$) of 3-amino-2-(4-chlorobenzoyl)-1*H*-inden-1-one (**4d**): full scale spectrum (top) and spectrum expansions (bottom).

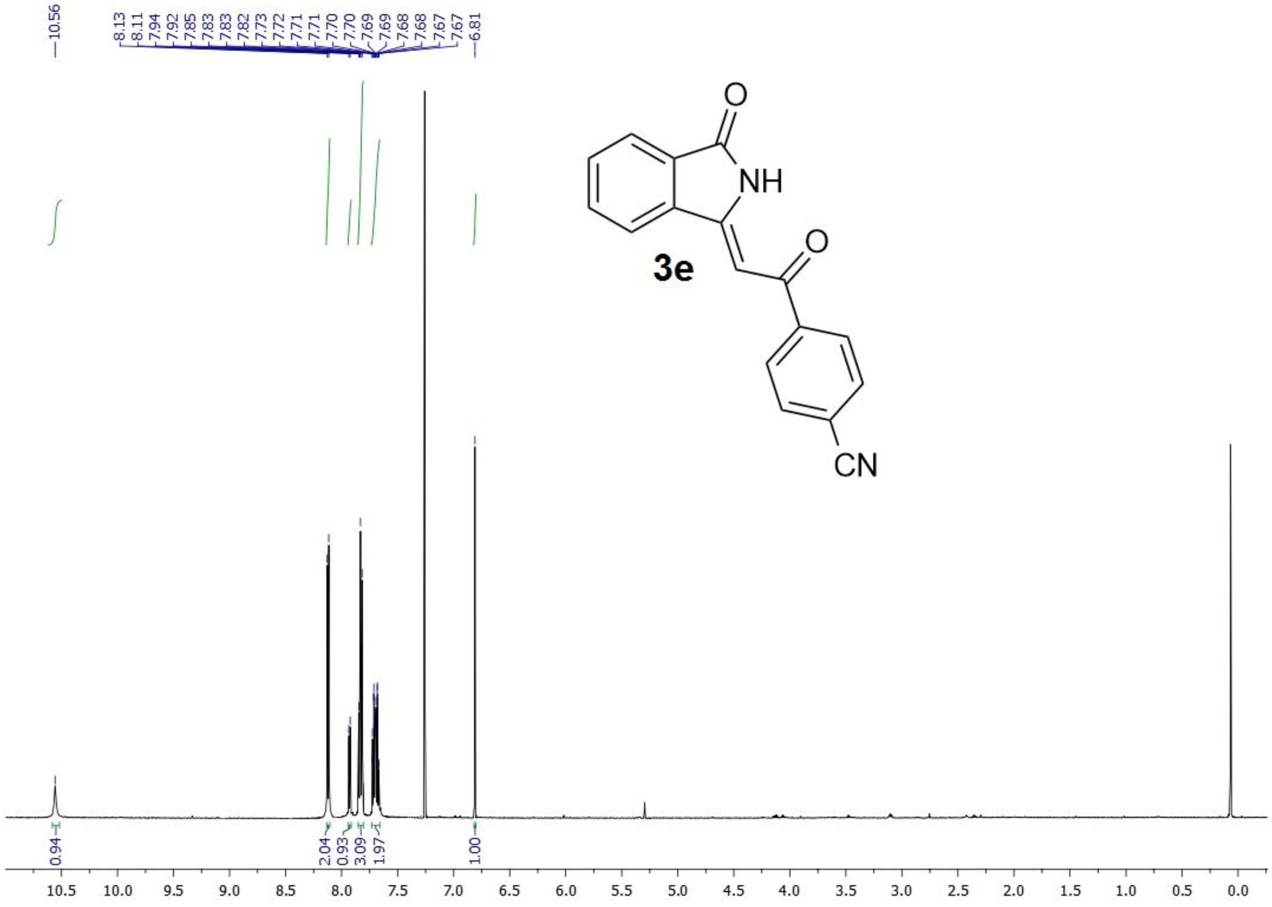

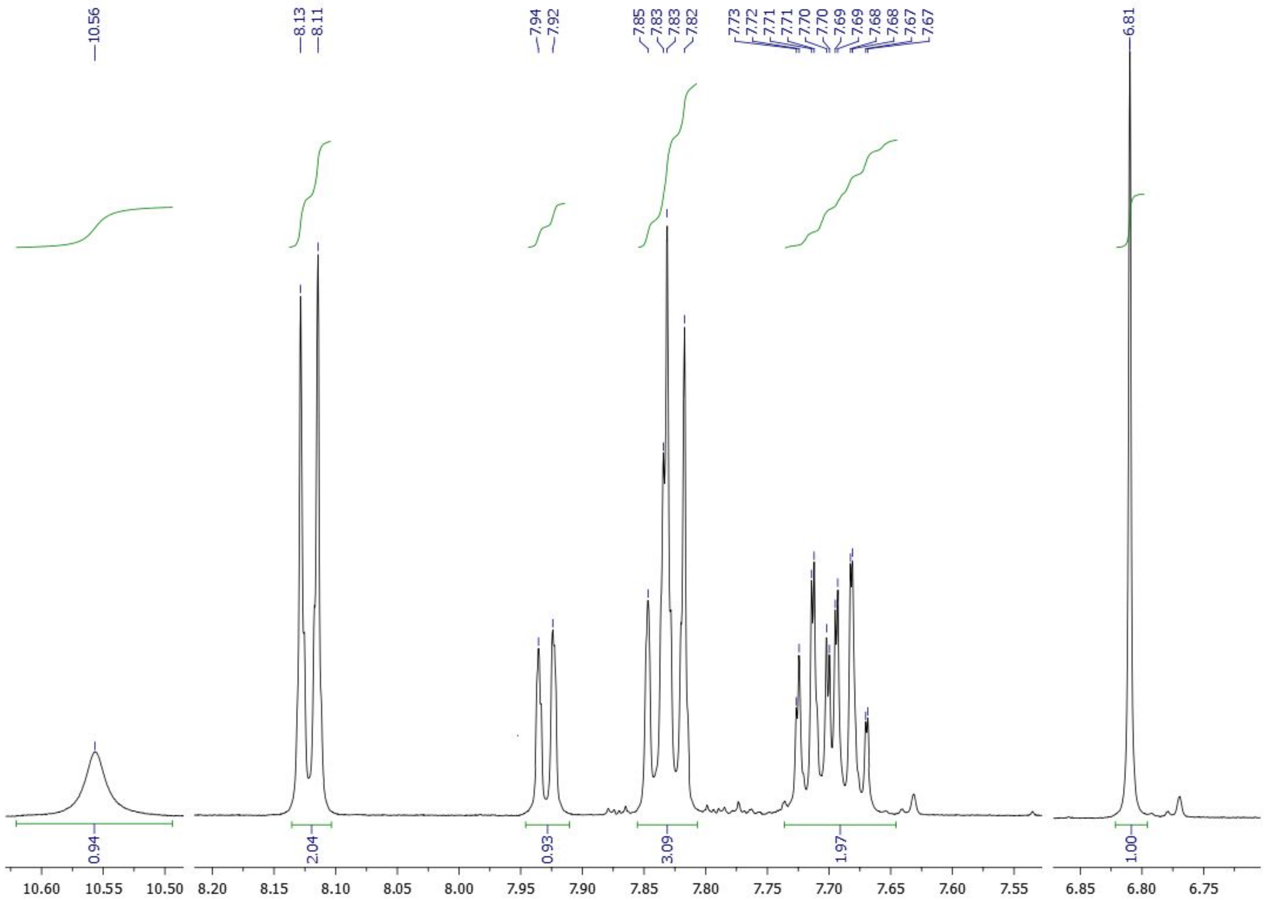

Figure S31. ^{1}H-NMR spectrum (600 MHz, $CDCl_3$) of (*Z*)-4-(2-(3-oxoisoindolin-1-ylidene)acetyl)benzonitrile (**3e**): full scale spectrum (top) and spectrum expansions (bottom).

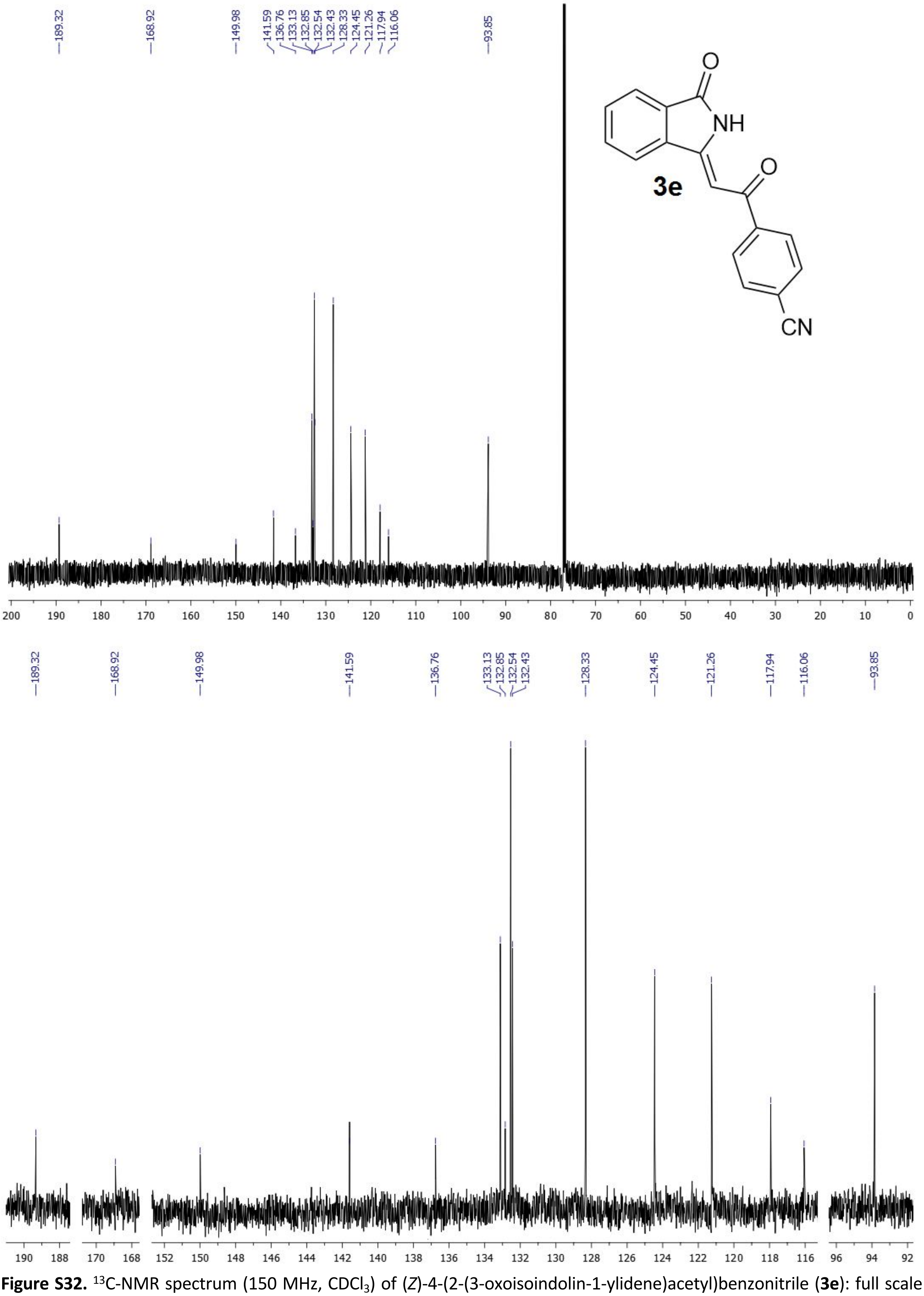

Figure S32. ^{13}C-NMR spectrum (150 MHz, $CDCl_3$) of (*Z*)-4-(2-(3-oxoisoindolin-1-ylidene)acetyl)benzonitrile (**3e**): full scale spectrum (top) and spectrum expansions (bottom).

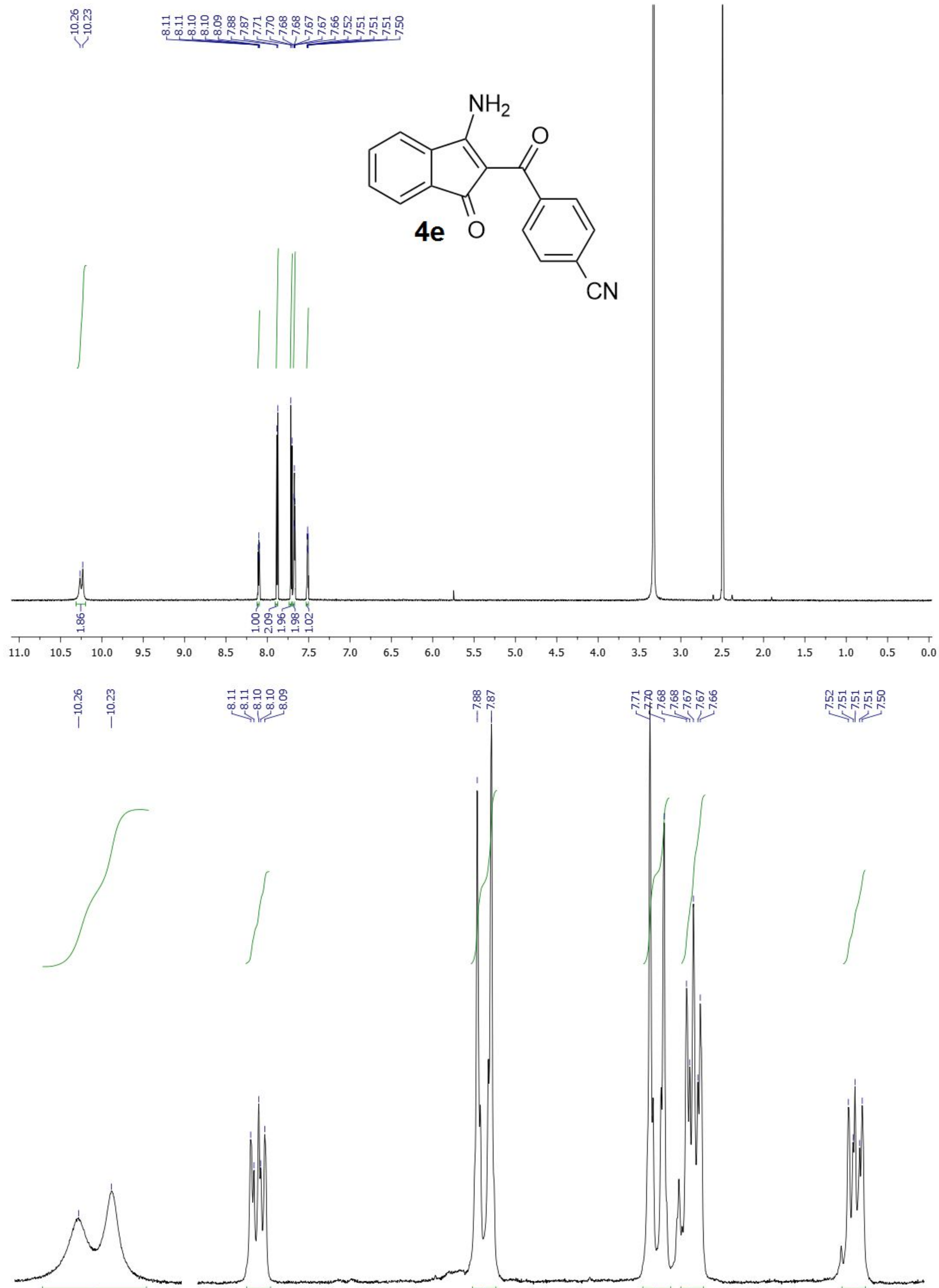

Figure S33. ^{1}H-NMR spectrum (600 MHz, $CDCl_3$) of 4-(3-amino-1-oxo-1*H*-indene-2-carbonyl)benzonitrile (**4e**): full scale spectrum (top) and spectrum expansions (bottom).

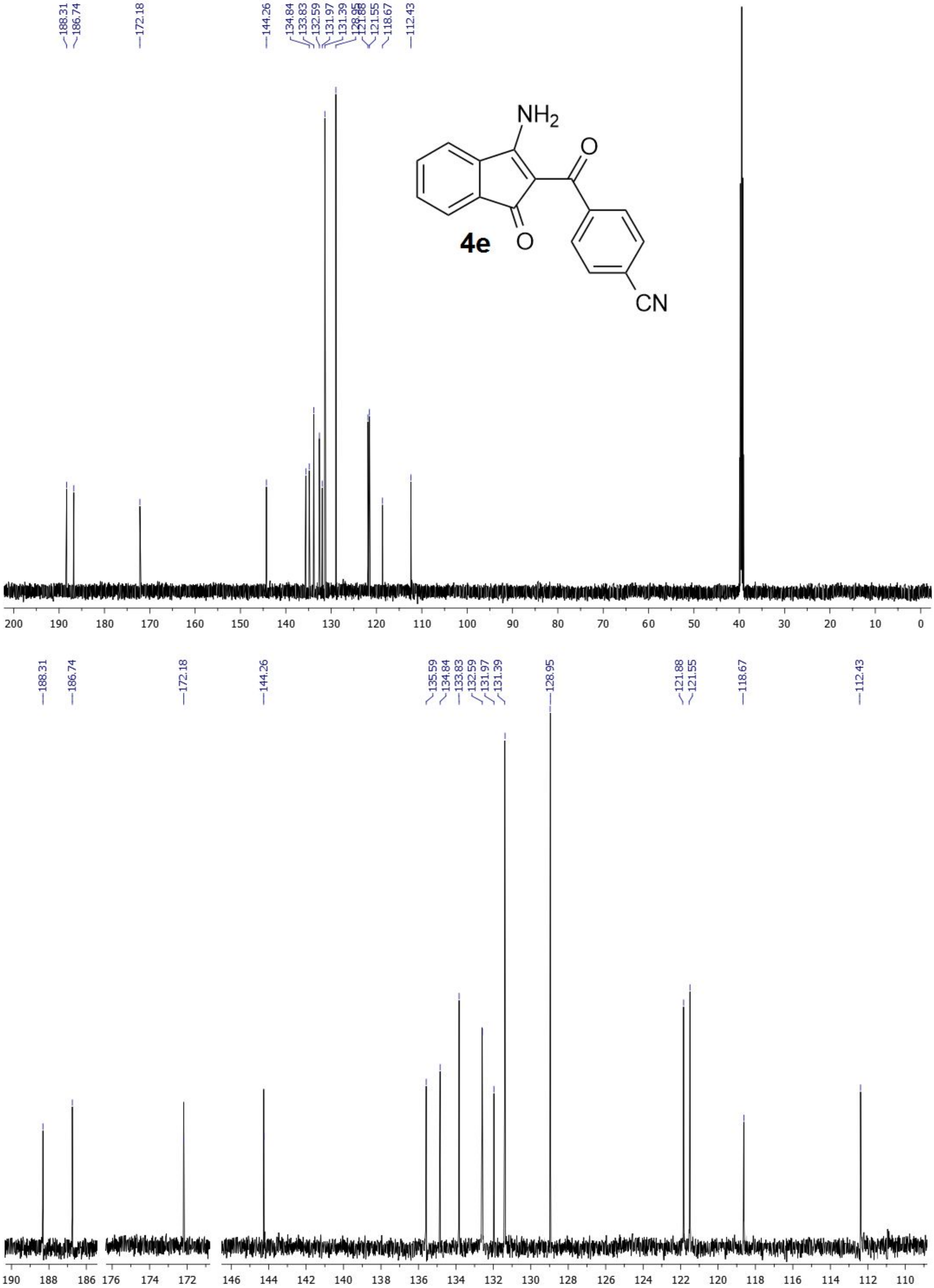

Figure S34. ^{13}C-NMR spectrum (150 MHz, $CDCl_3$) of 4-(3-amino-1-oxo-1*H*-indene-2-carbonyl)benzonitrile (**4e**): full scale spectrum (top) and spectrum expansions (bottom).

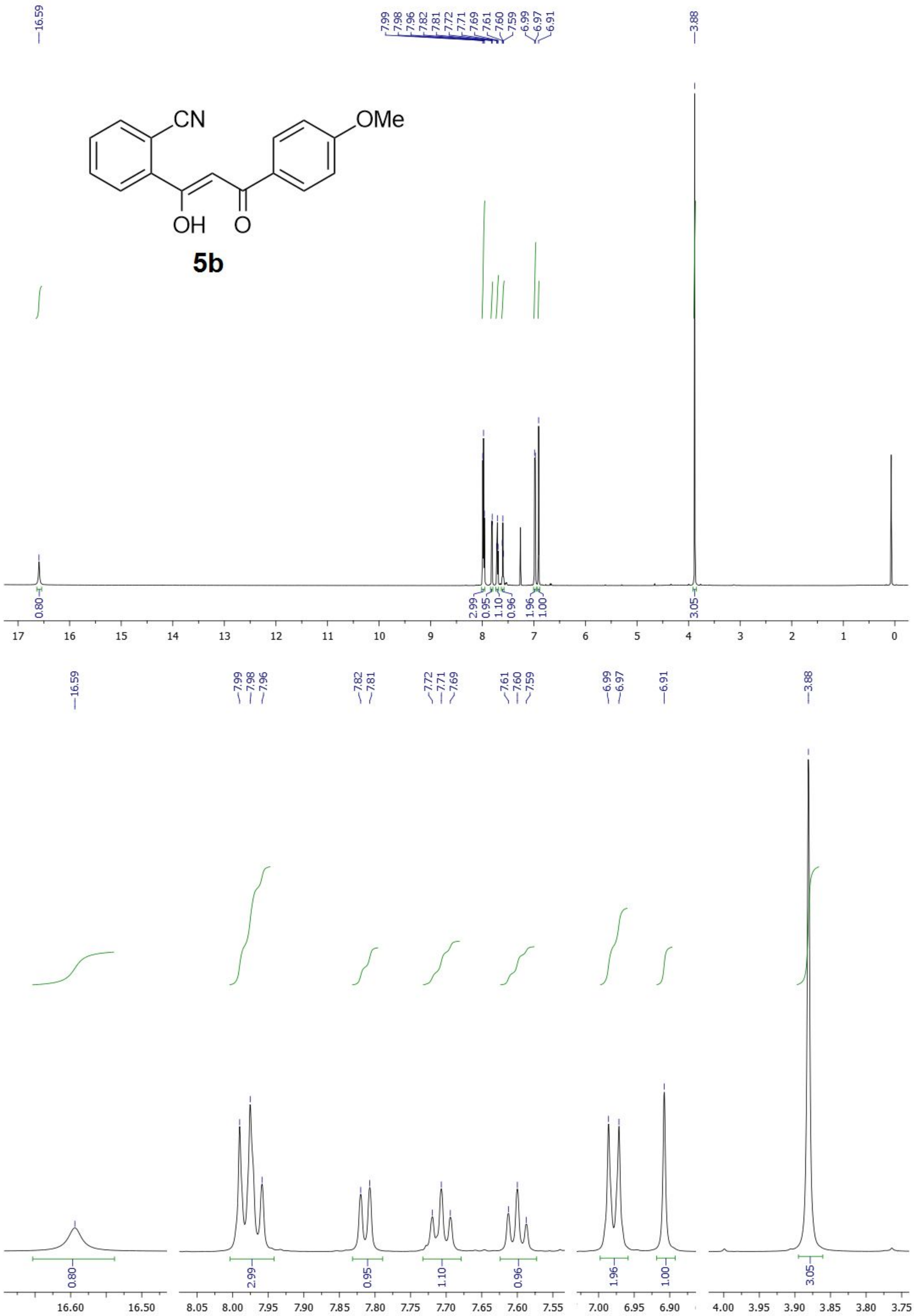

Figure S35. ^{1}H-NMR spectrum (600 MHz, $CDCl_3$) of (*Z*)-2-(1-hydroxy-3-(4-methoxyphenyl)-3-oxoprop-1-en-1-yl)benzonitrile (**5b**): full scale spectrum (top) and spectrum expansions (bottom).

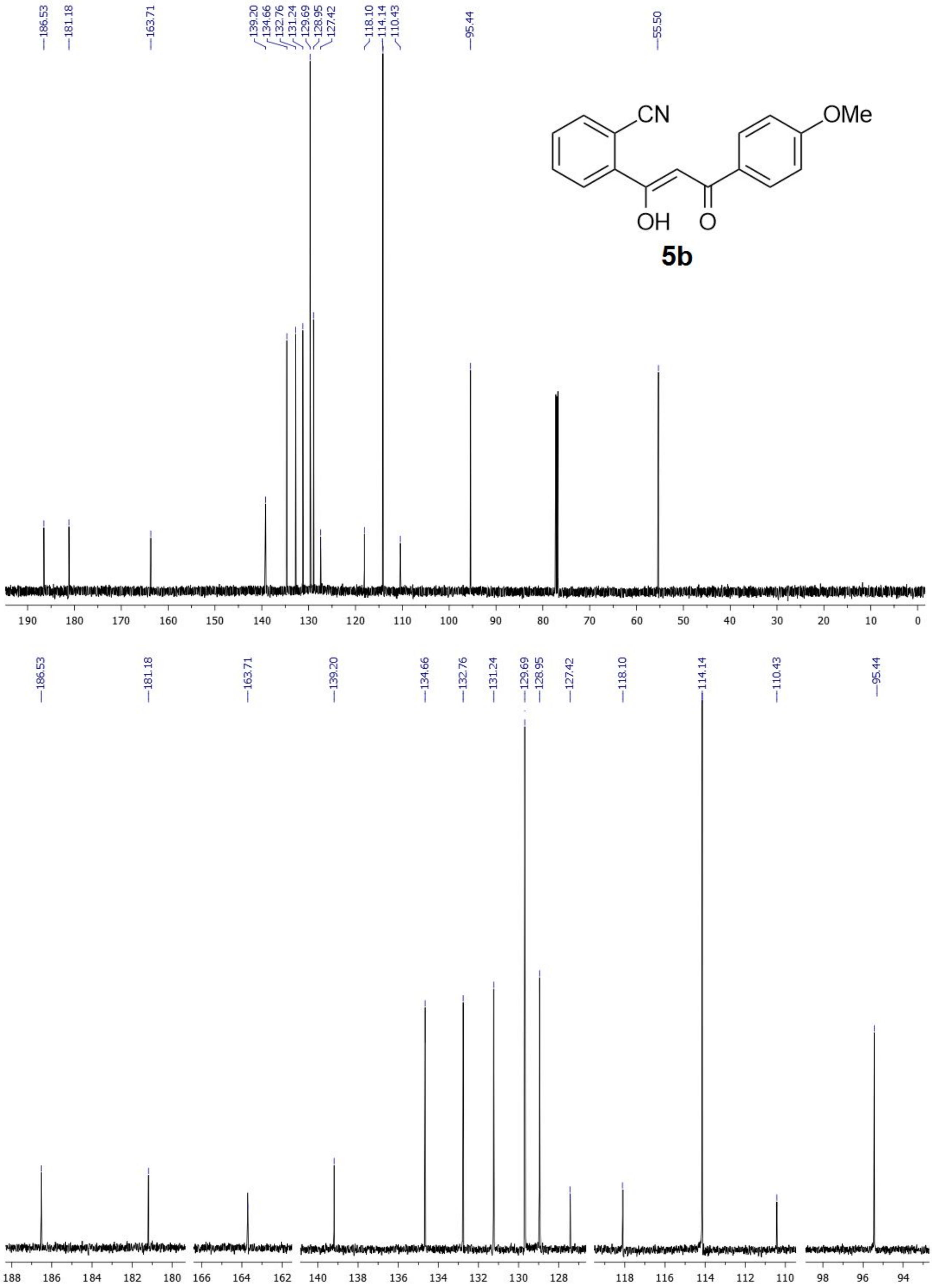

Figure S36. ^{13}C-NMR spectrum (150 MHz, $CDCl_3$) of (*Z*)-2-(1-hydroxy-3-(4-methoxyphenyl)-3-oxoprop-1-en-1-yl)benzonitrile (**5b**): full scale spectrum (top) and spectrum expansions (bottom).

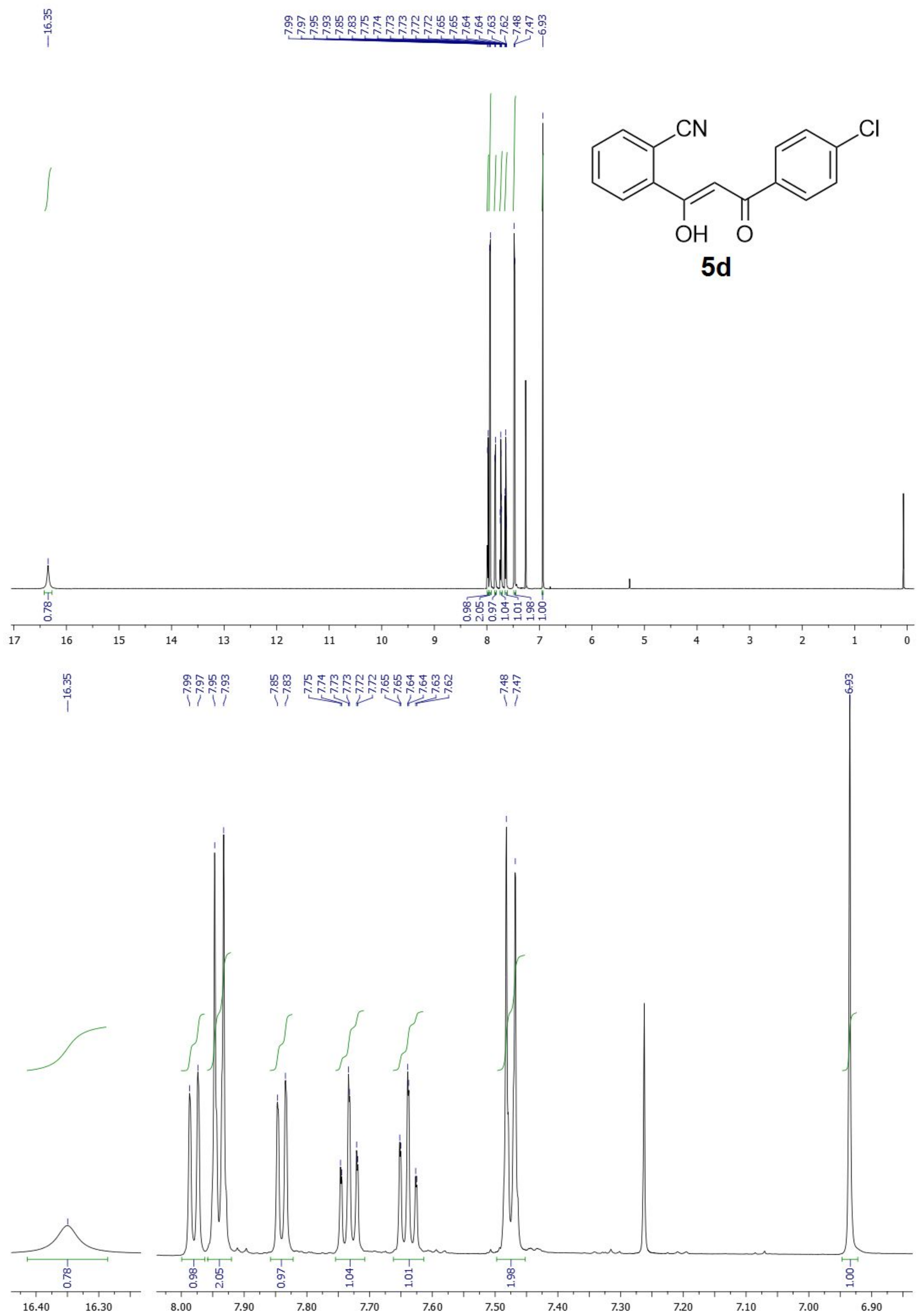

Figure S37. ^{1}H-NMR spectrum (600 MHz, $CDCl_3$) of (*Z*)-2-(3-(4-chlorophenyl)-1-hydroxy-3-oxoprop-1-en-1-yl)benzonitrile (**5d**): full scale spectrum (top) and spectrum expansions (bottom).

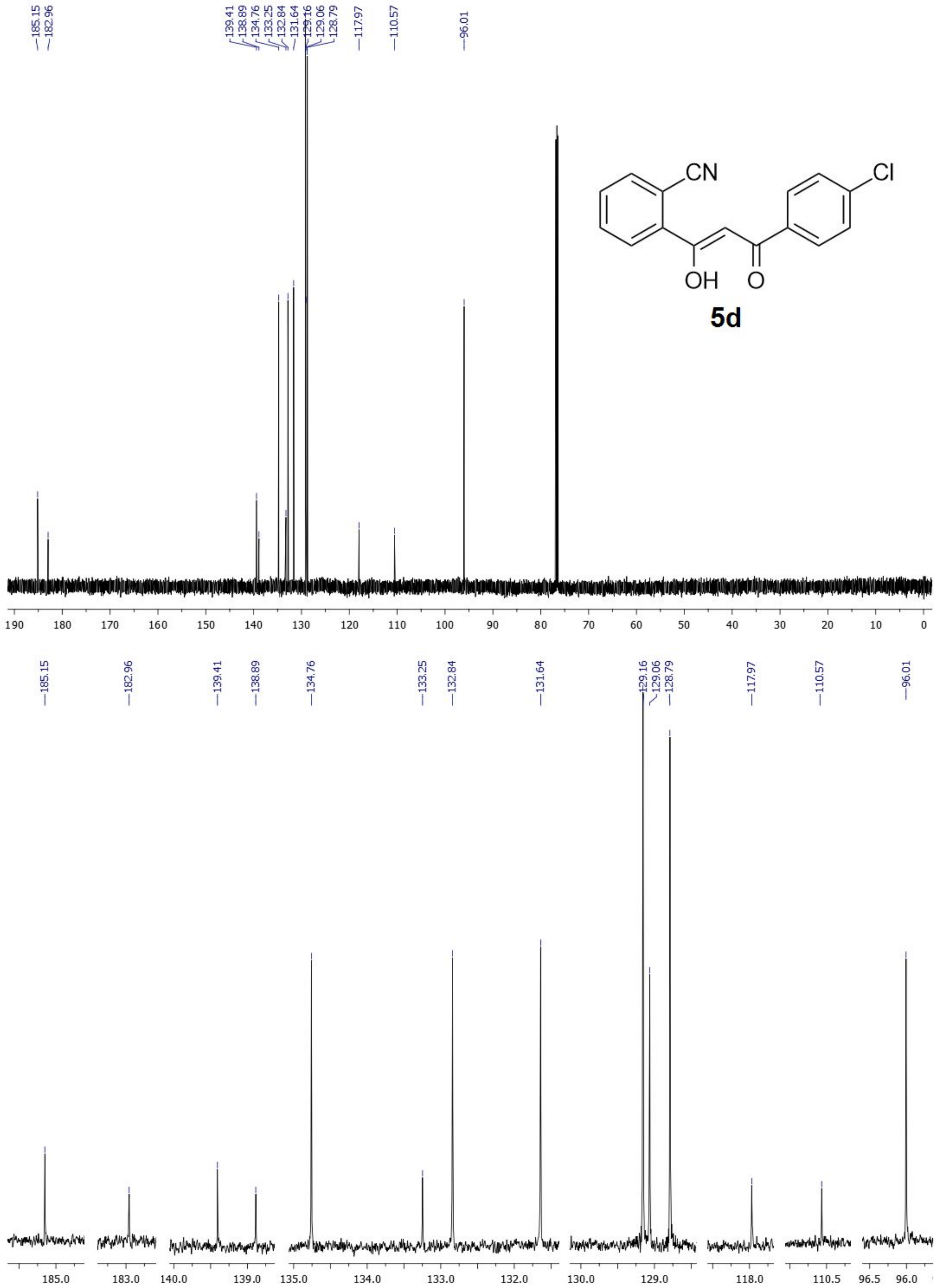

Figure S38. ^{13}C-NMR spectrum (150 MHz, $CDCl_3$) of (*Z*)-2-(3-(4-chlorophenyl)-1-hydroxy-3-oxoprop-1-en-1-yl)benzonitrile (**5d**): full scale spectrum (top) and spectrum expansions (bottom).

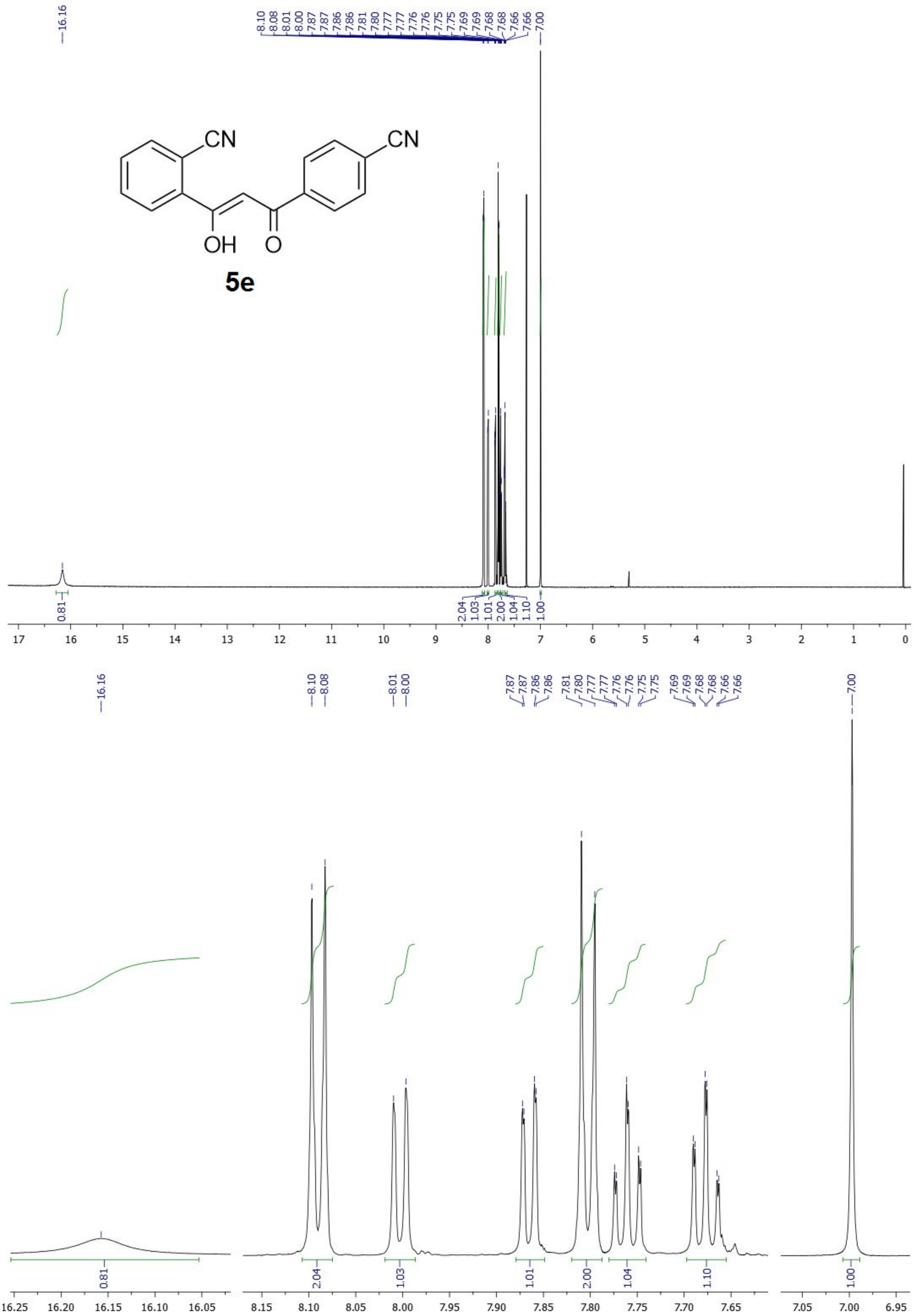

Figure S39. ^{1}H-NMR spectrum (600 MHz, $CDCl_3$) of (*Z*)-2-(3-(4-cyanophenyl)-1-hydroxy-3-oxoprop-1-en-1-yl)benzonitrile (**5e**): full scale spectrum (top) and spectrum expansions (bottom).

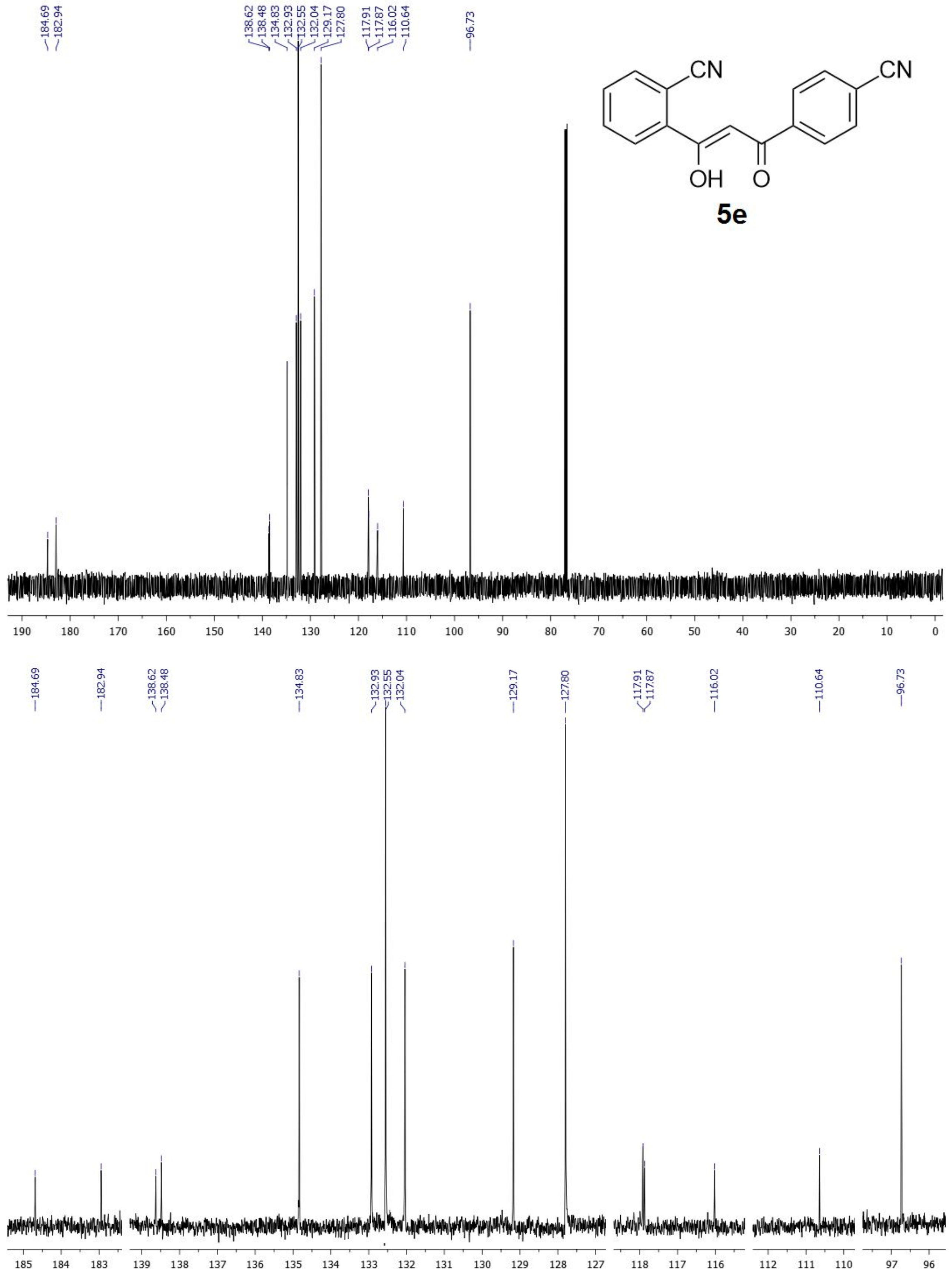

Figure S40. ^{13}C-NMR spectrum (150 MHz, $CDCl_3$) of (*Z*)-2-(3-(4-cyanophenyl)-1-hydroxy-3-oxoprop-1-en-1-yl)benzonitrile (**5e**): full scale spectrum (top) and spectrum expansions (bottom).

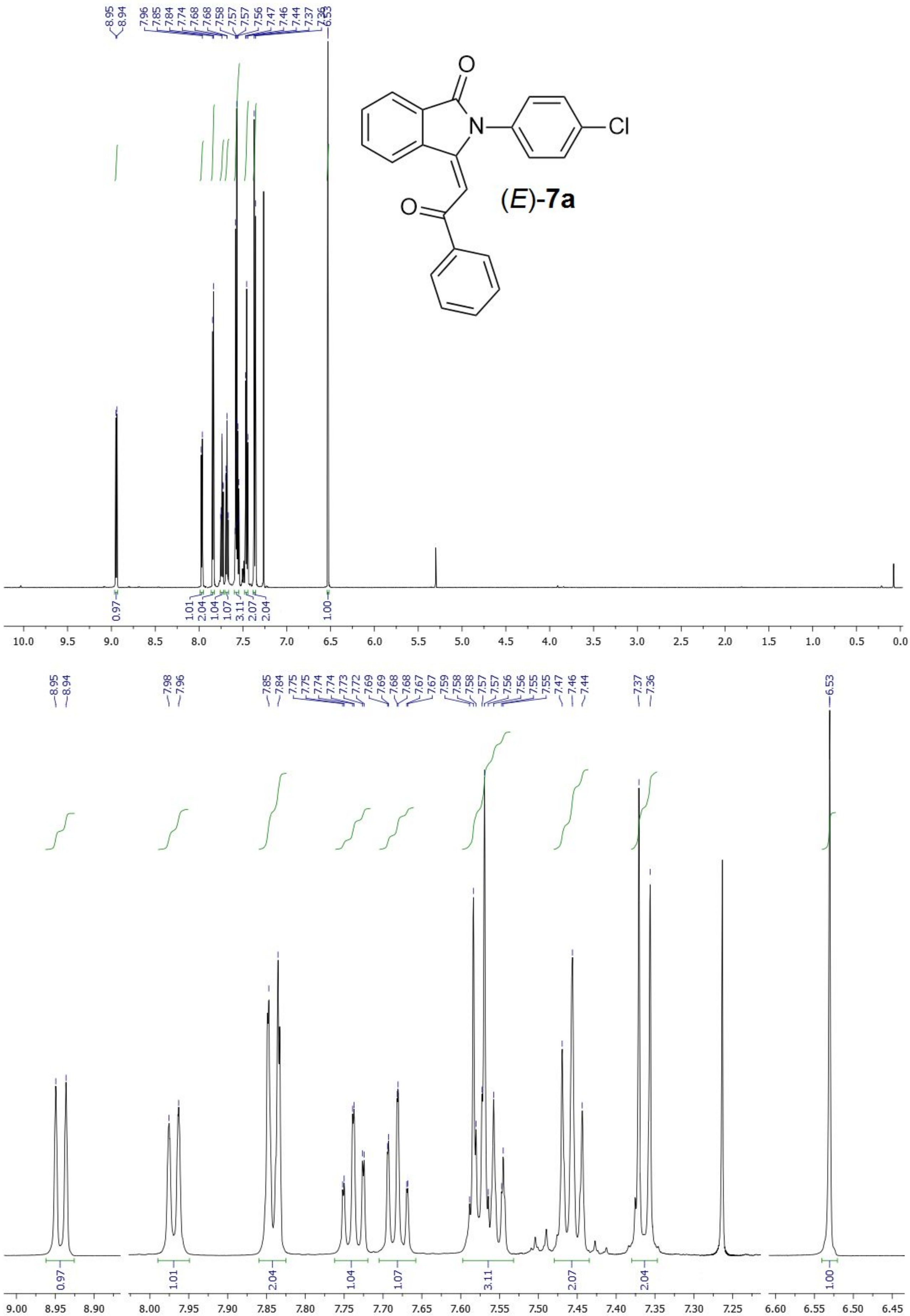

Figure S41. ^{1}H-NMR spectrum (600 MHz, $CDCl_3$) of (*E*)-2-(4-chlorophenyl)-3-(2-oxo-2-phenylethylidene)isoindolin-1-one ((*E*)-**7a**): full scale spectrum (top) and spectrum expansions (bottom).

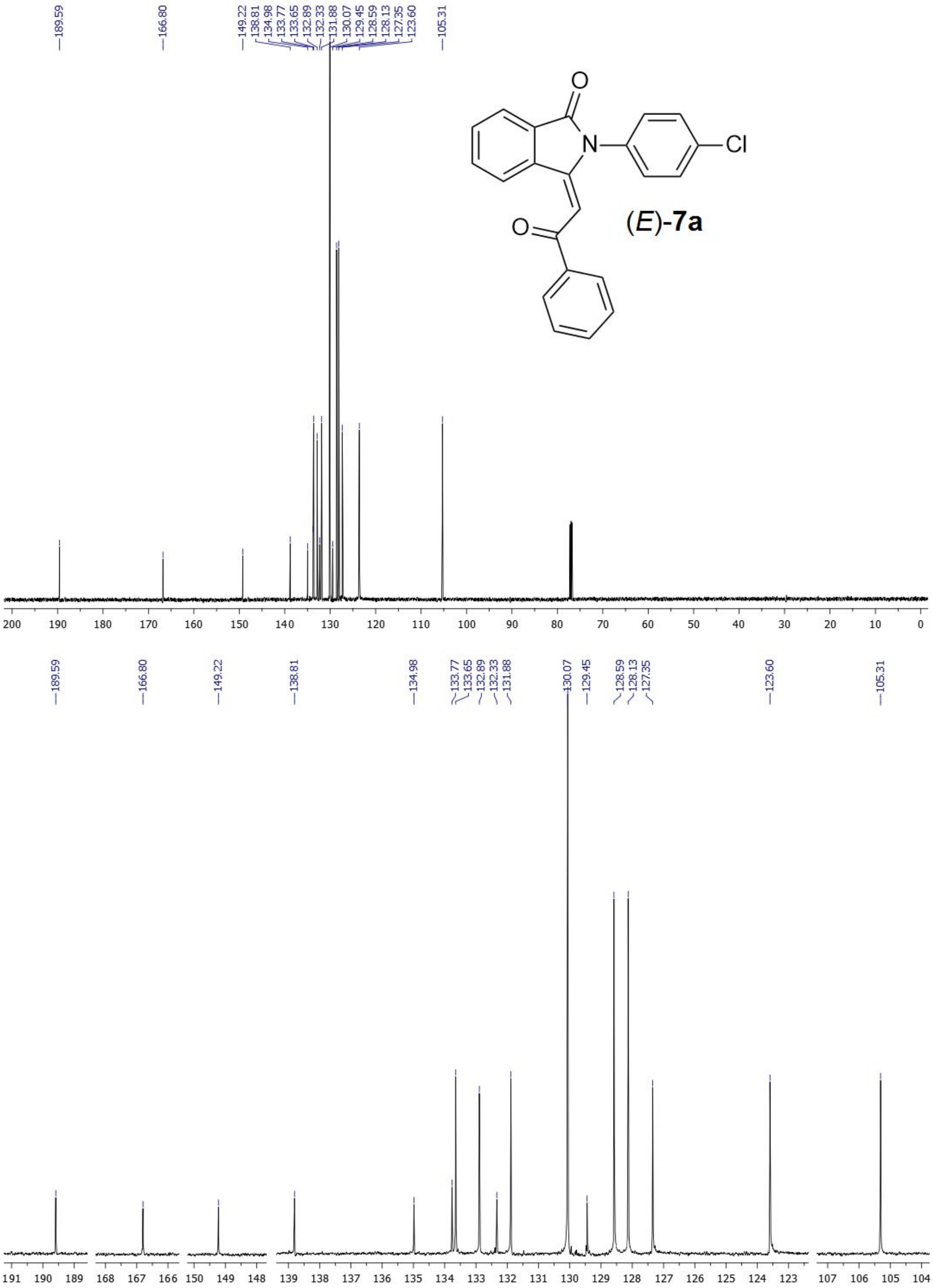

Figure S42. ^{13}C-NMR spectrum (150 MHz, $CDCl_3$) of (*E*)-2-(4-chlorophenyl)-3-(2-oxo-2-phenylethylidene)isoindolin-1-one ((*E*)-**7a**): full scale spectrum (top) and spectrum expansions (bottom).

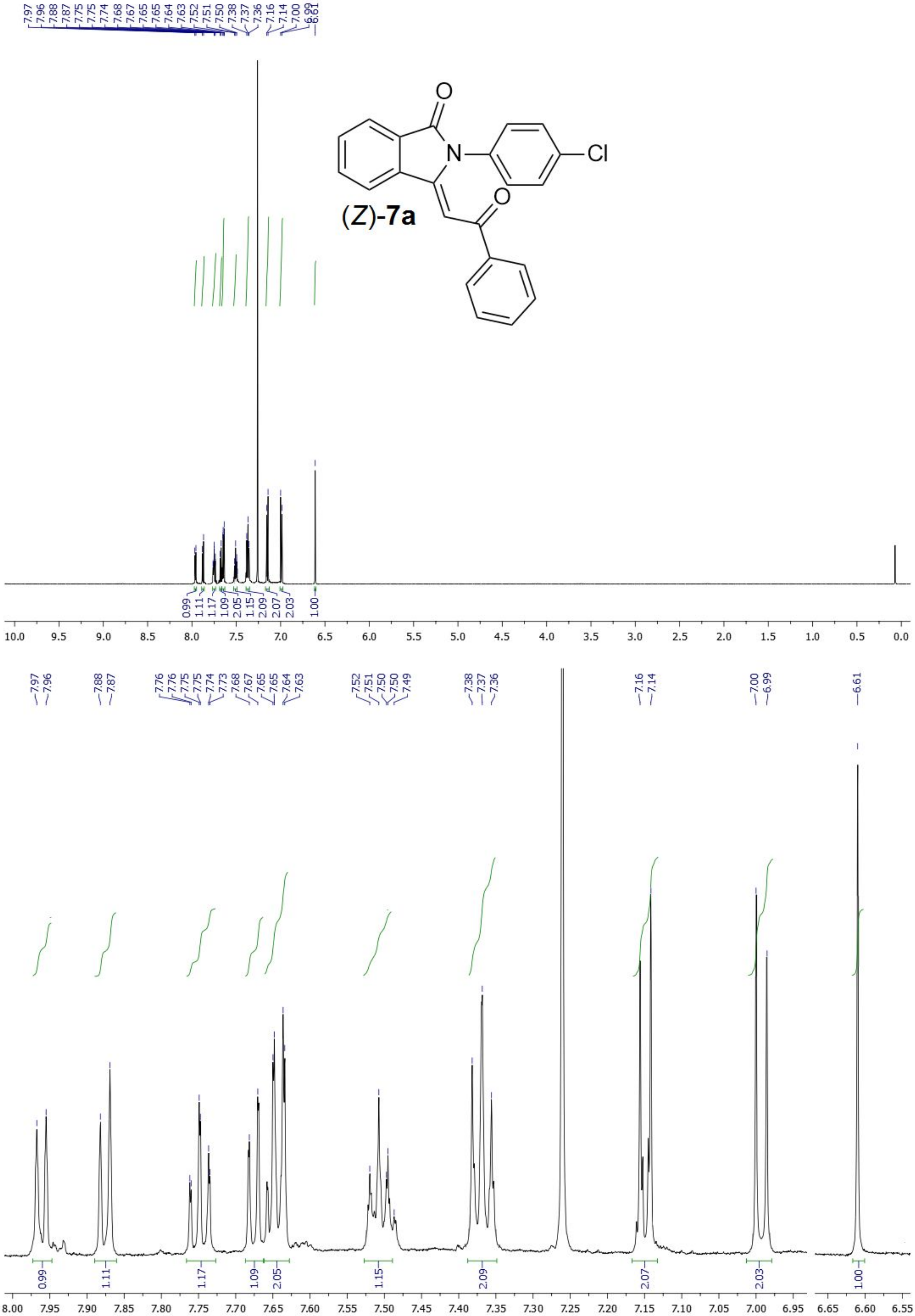

Figure S43. ^{1}H-NMR spectrum (600 MHz, $CDCl_3$) of (*Z*)-2-(4-chlorophenyl)-3-(2-oxo-2-phenylethylidene)isoindolin-1-one ((*Z*)-**7a**): full scale spectrum (top) and spectrum expansions (bottom).

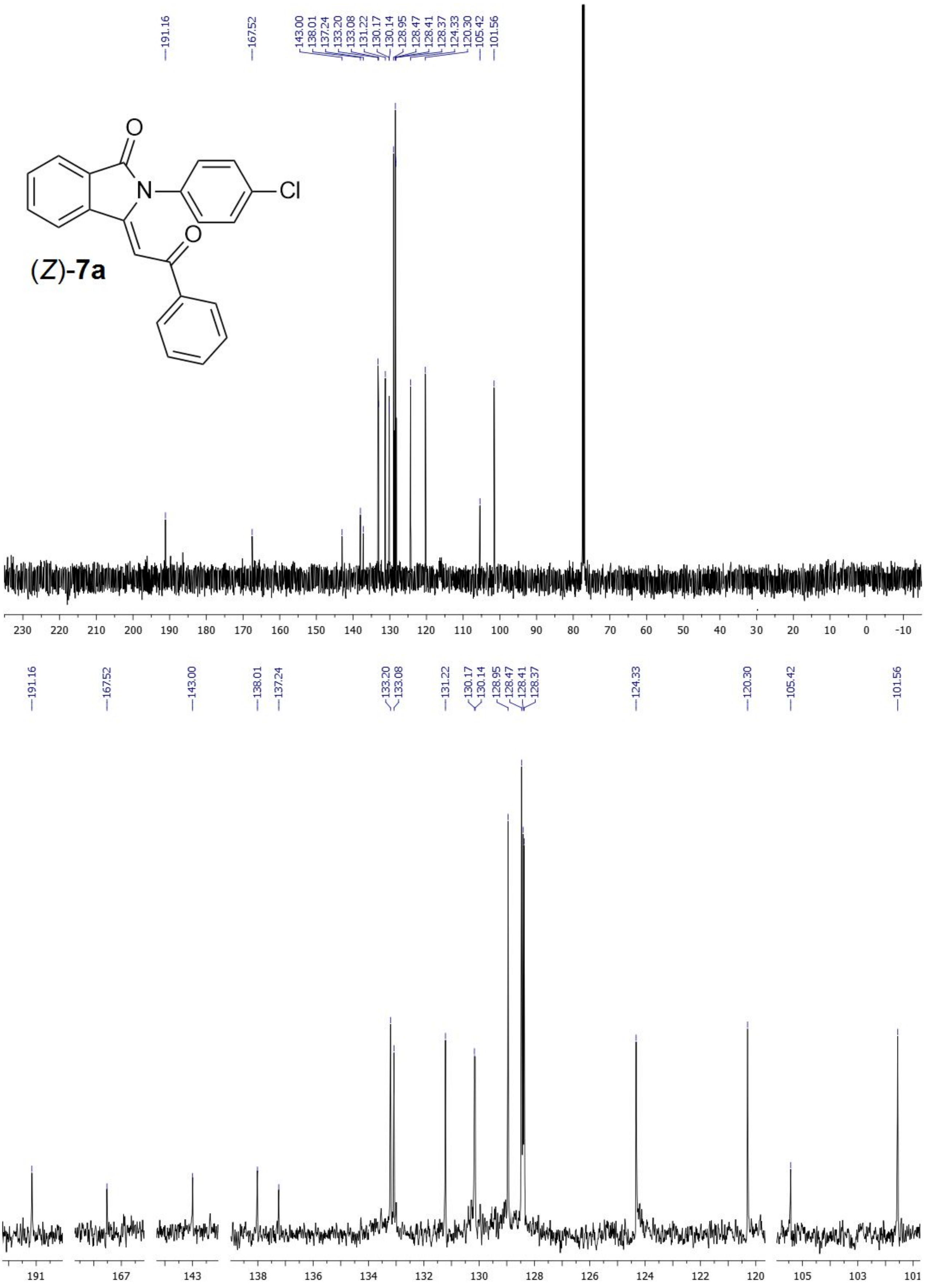

Figure S44. ^{13}C-NMR spectrum (150 MHz, $CDCl_3$) of (*Z*)-2-(4-chlorophenyl)-3-(2-oxo-2-phenylethylidene)isoindolin-1-one ((*Z*)-**7a**): full scale spectrum (top) and spectrum expansions (bottom).

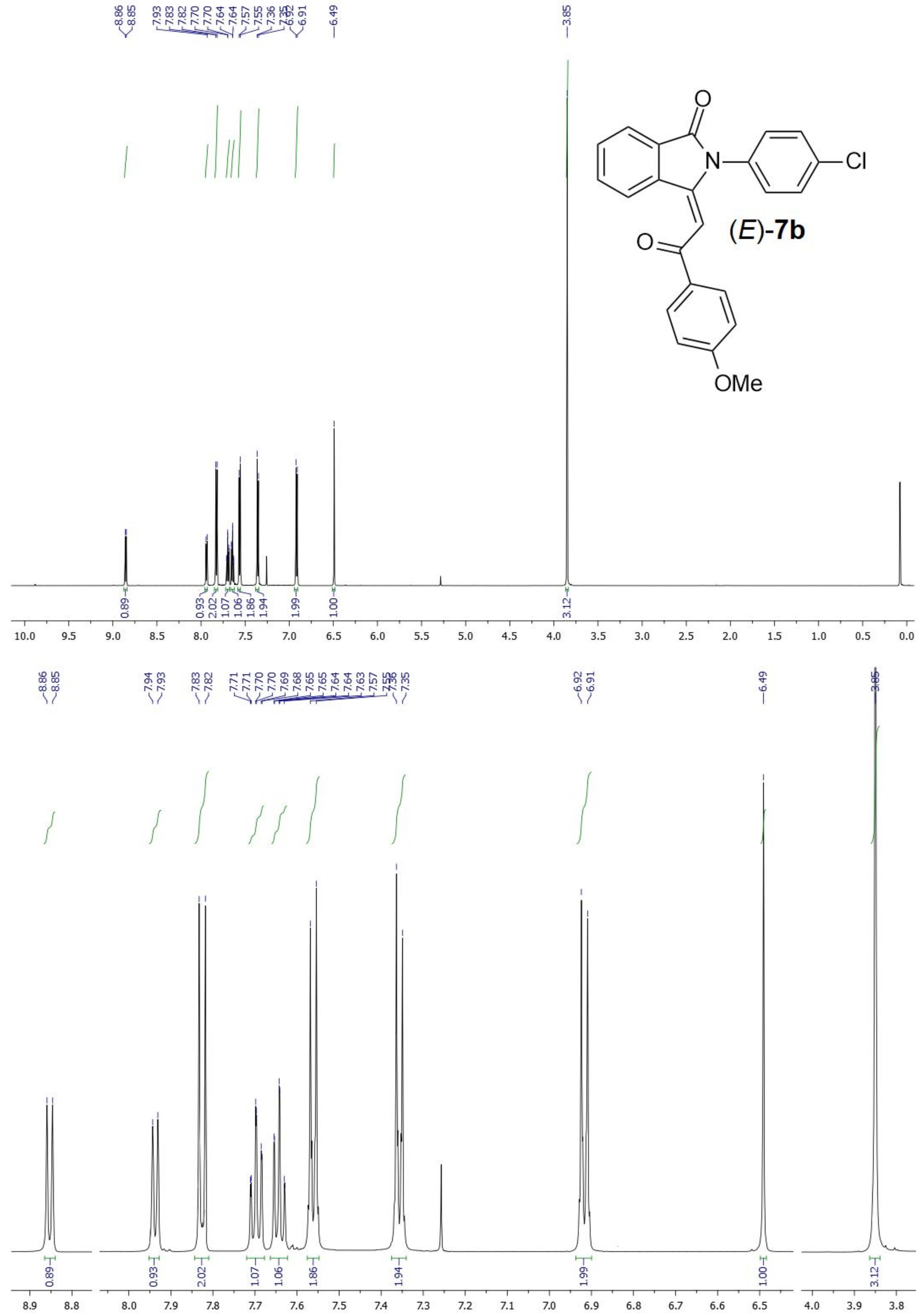

Figure S45. ^{1}H-NMR spectrum (600 MHz, $CDCl_3$) of (*E*)-2-(4-chlorophenyl)-3-(2-(4-methoxyphenyl)-2-oxoethylidene) isoindolin-1-one ((*E*)-**7b**): full scale spectrum (top) and spectrum expansions (bottom).

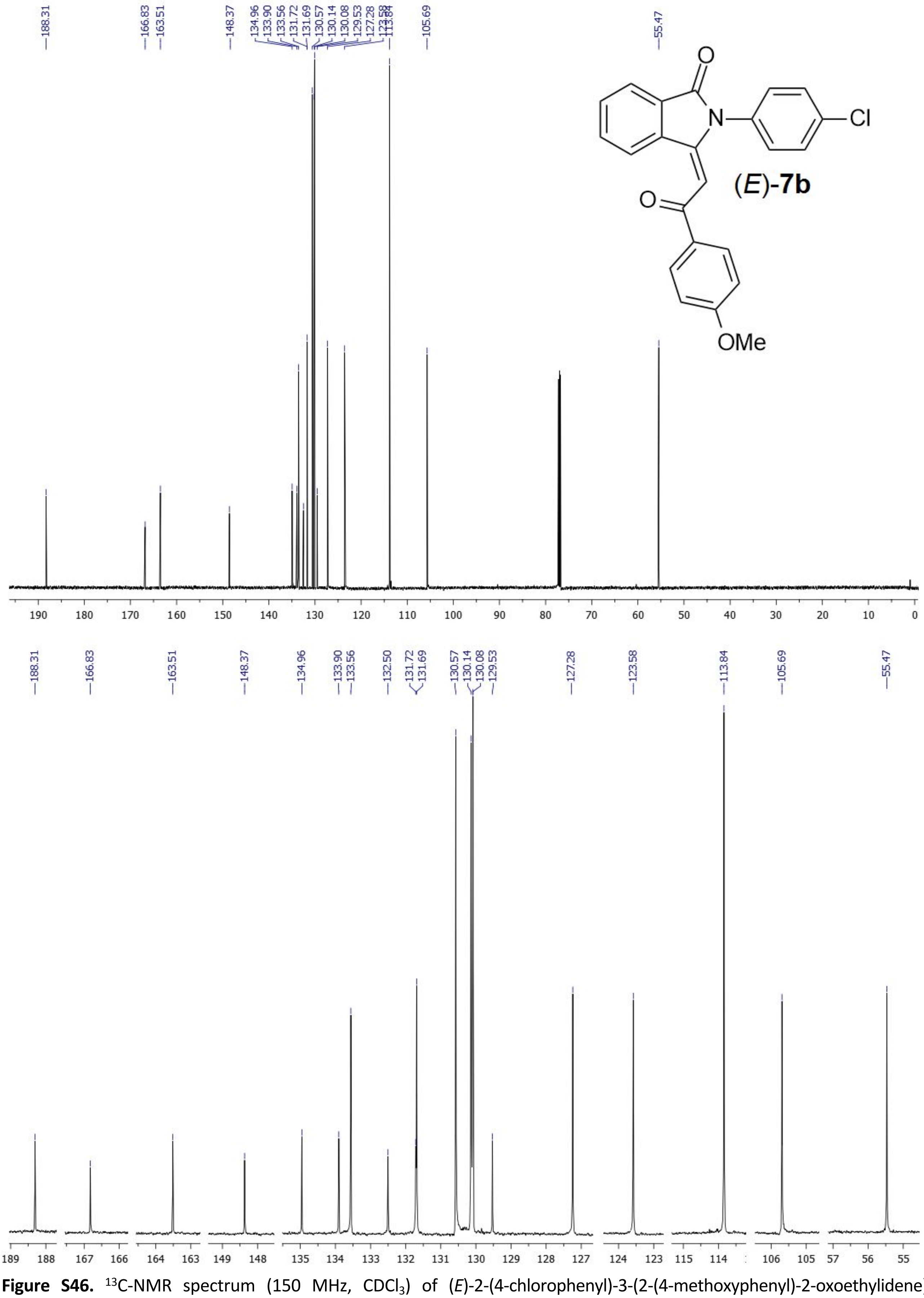

Figure S46. ^{13}C-NMR spectrum (150 MHz, $CDCl_3$) of (*E*)-2-(4-chlorophenyl)-3-(2-(4-methoxyphenyl)-2-oxoethylidene) isoindolin-1-one ((*E*)-**7b**): full scale spectrum (top) and spectrum expansions (bottom).

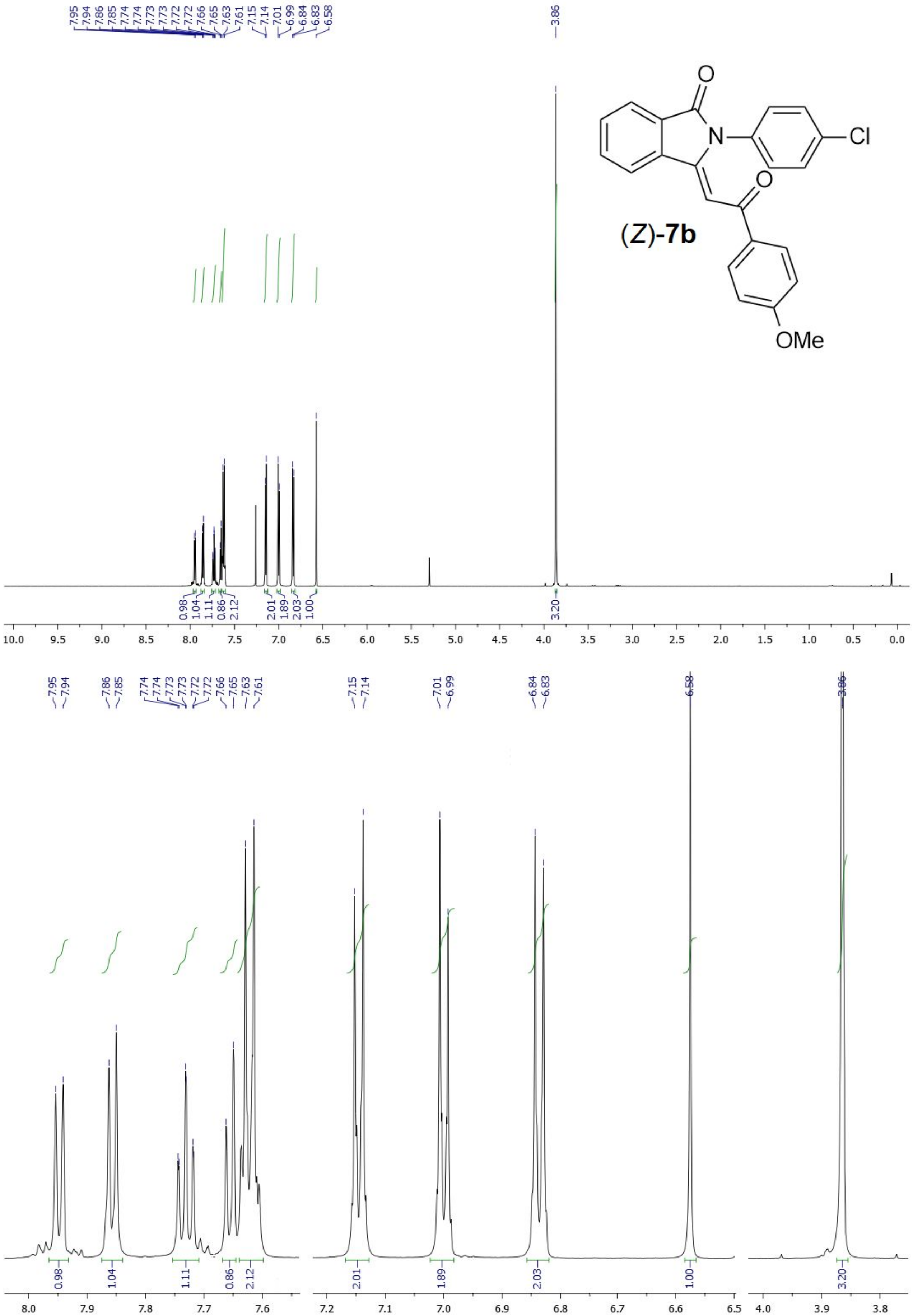

Figure S47. ^{1}H-NMR spectrum (600 MHz, $CDCl_3$) of (*Z*)-2-(4-chlorophenyl)-3-(2-(4-methoxyphenyl)-2-oxoethylidene) isoindolin-1-one ((*Z*)-**7b**): full scale spectrum (top) and spectrum expansions (bottom).

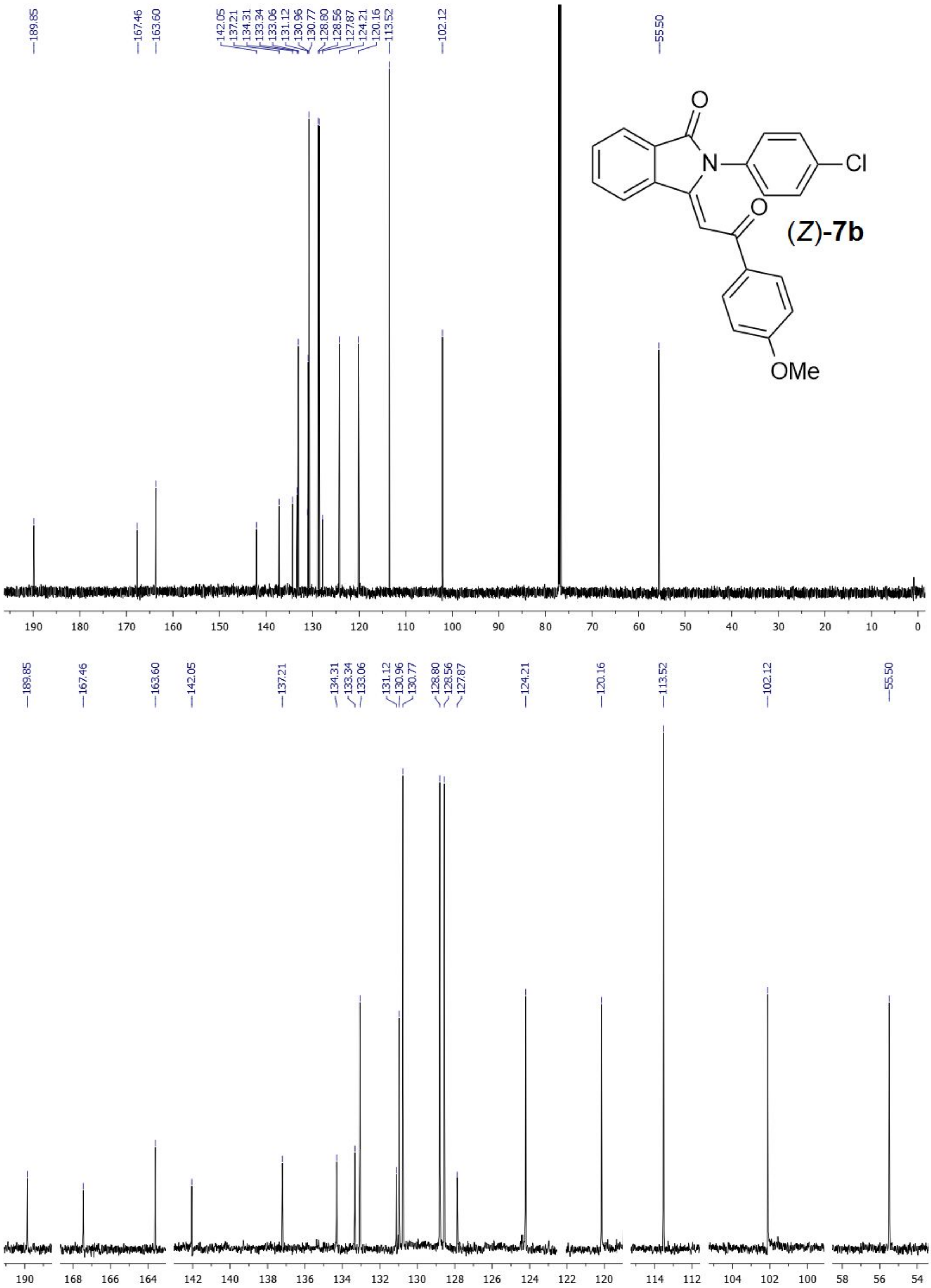

Figure S48. ^{13}C-NMR spectrum (150 MHz, $CDCl_3$) of (*Z*)-2-(4-chlorophenyl)-3-(2-(4-methoxyphenyl)-2-oxoethylidene) isoindolin-1-one ((*Z*)-**7b**): full scale spectrum (top) and spectrum expansions (bottom).

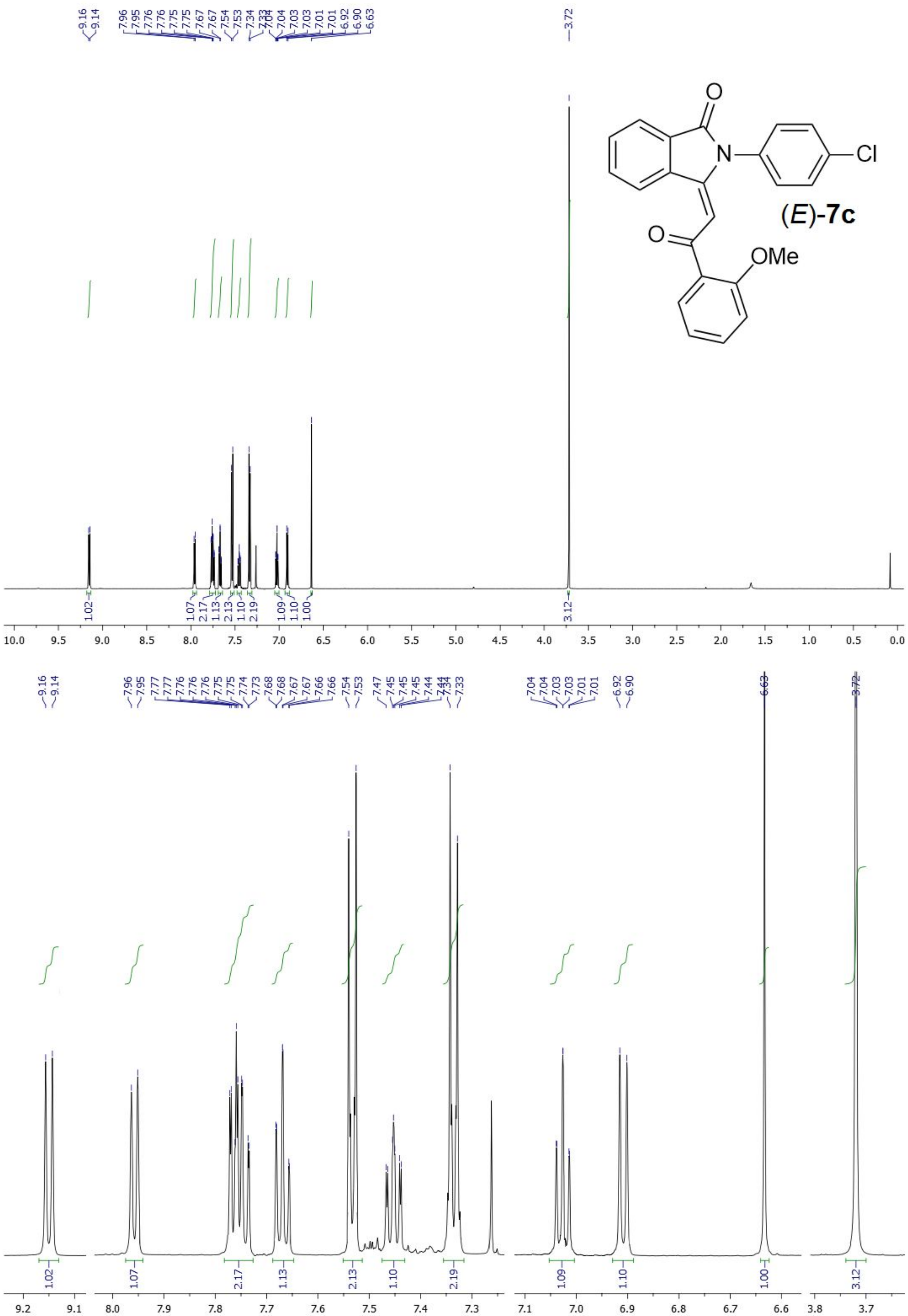

Figure S49. ^{1}H-NMR spectrum (600 MHz, $CDCl_3$) of (*E*)-2-(4-chlorophenyl)-3-(2-(2-methoxyphenyl)-2-oxoethylidene) isoindolin-1-one ((*E*)-**7c**): full scale spectrum (top) and spectrum expansions (bottom).

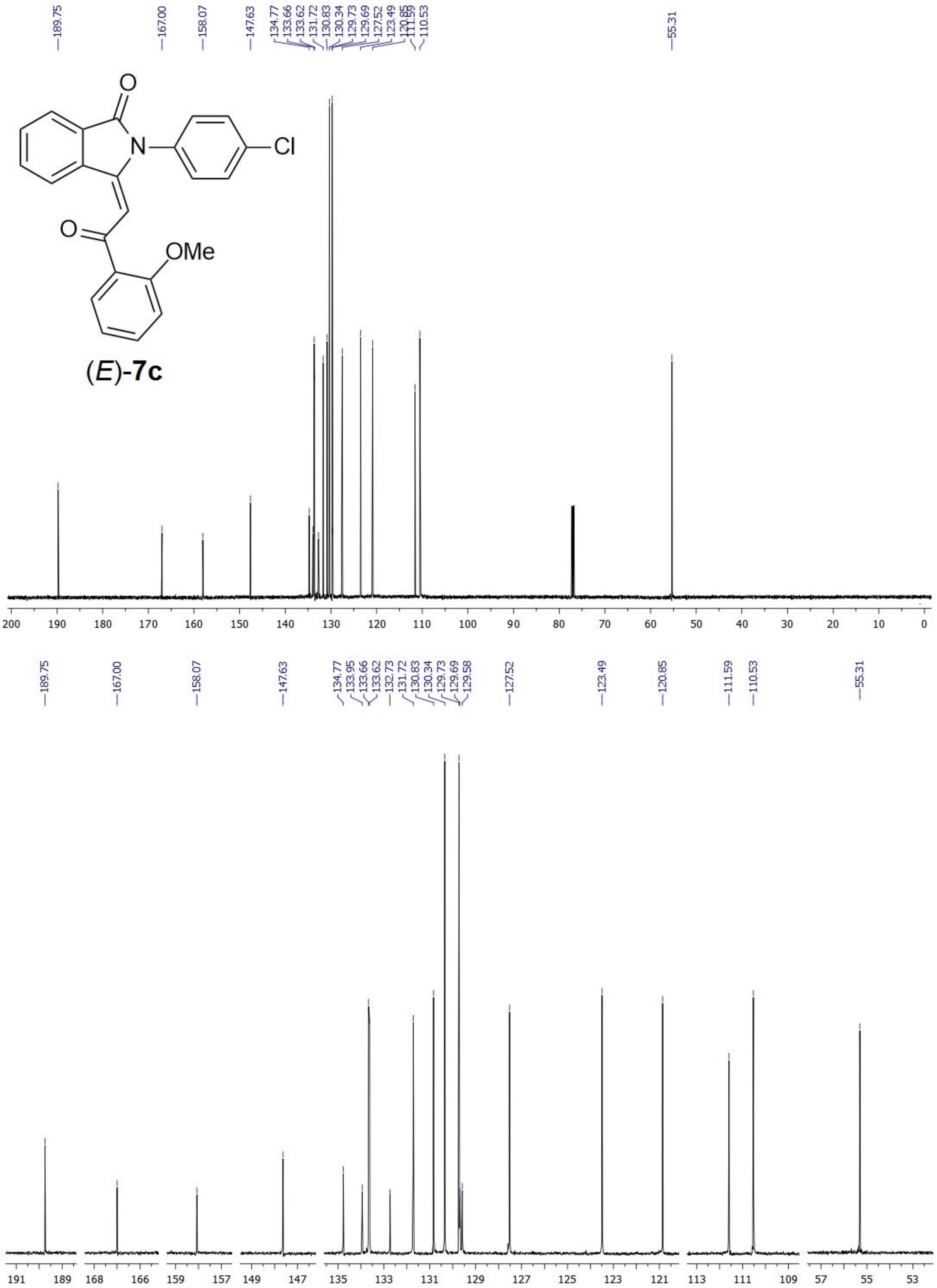

Figure S50. ^{13}C-NMR spectrum (150 MHz, $CDCl_3$) of (*E*)-2-(4-chlorophenyl)-3-(2-(2-methoxyphenyl)-2-oxoethylidene) isoindolin-1-one ((*E*)-**7c**): full scale spectrum (top) and spectrum expansions (bottom).

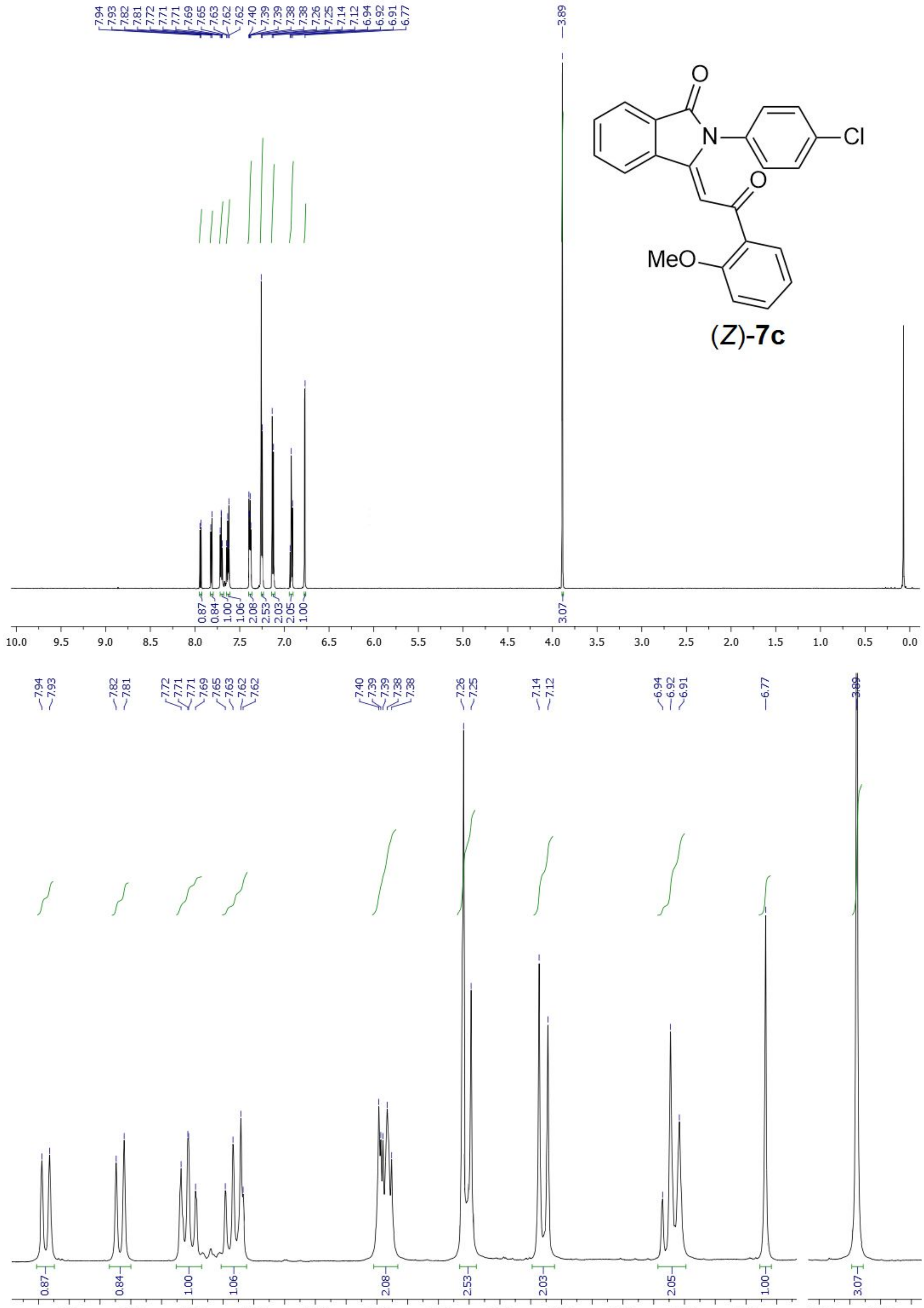

Figure S51. ^{1}H-NMR spectrum (600 MHz, $CDCl_3$) of (*Z*)-2-(4-chlorophenyl)-3-(2-(2-methoxyphenyl)-2-oxoethylidene) isoindolin-1-one ((*Z*)-**7c**): full scale spectrum (top) and spectrum expansions (bottom).

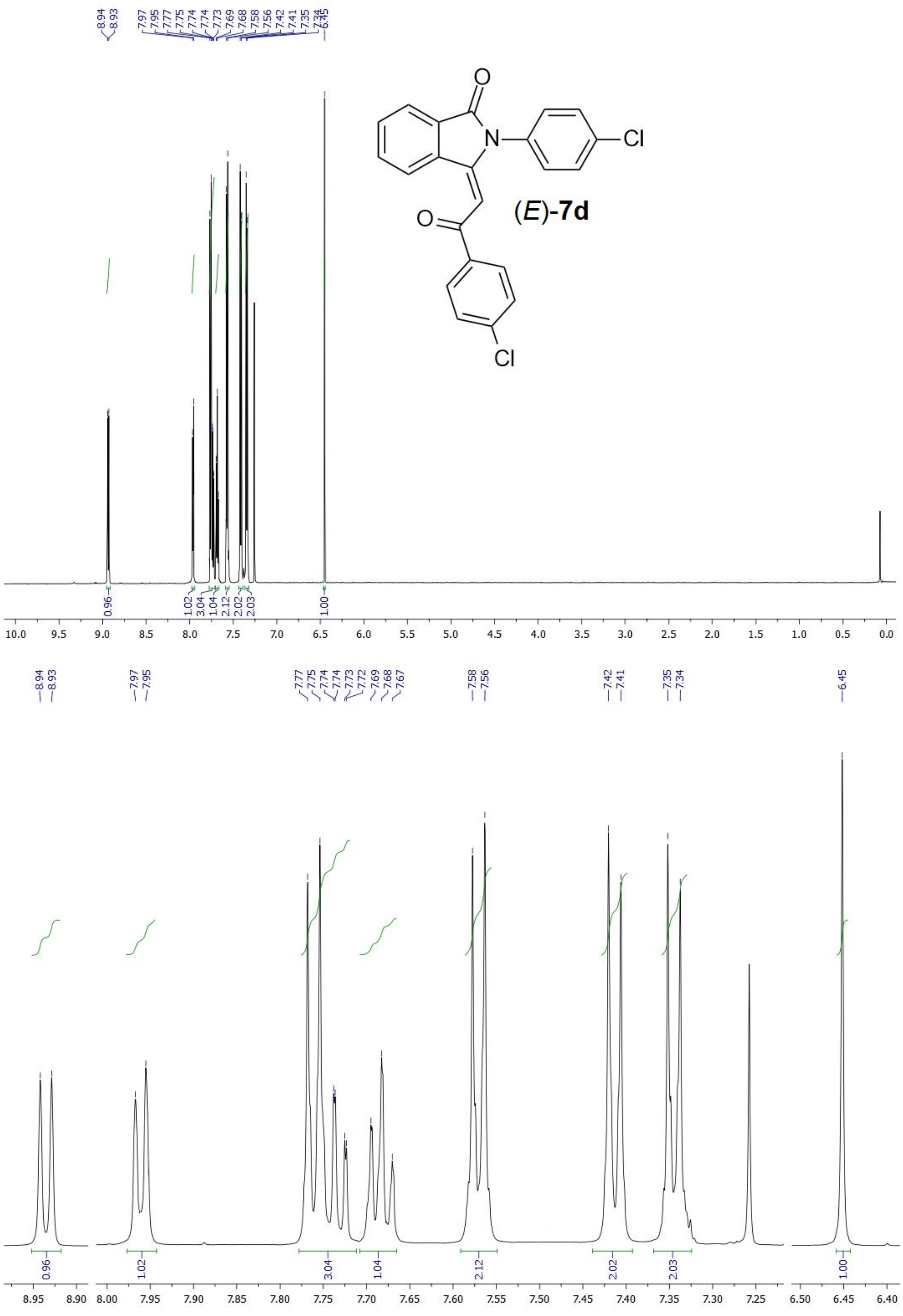

Figure S52. ^{1}H-NMR spectrum (600 MHz, $CDCl_3$) of (*E*)-2-(4-chlorophenyl)-3-(2-(4-chlorophenyl)-2-oxoethylidene)isoindolin-1-one ((*E*)-**7d**): full scale spectrum (top) and spectrum expansions (bottom).

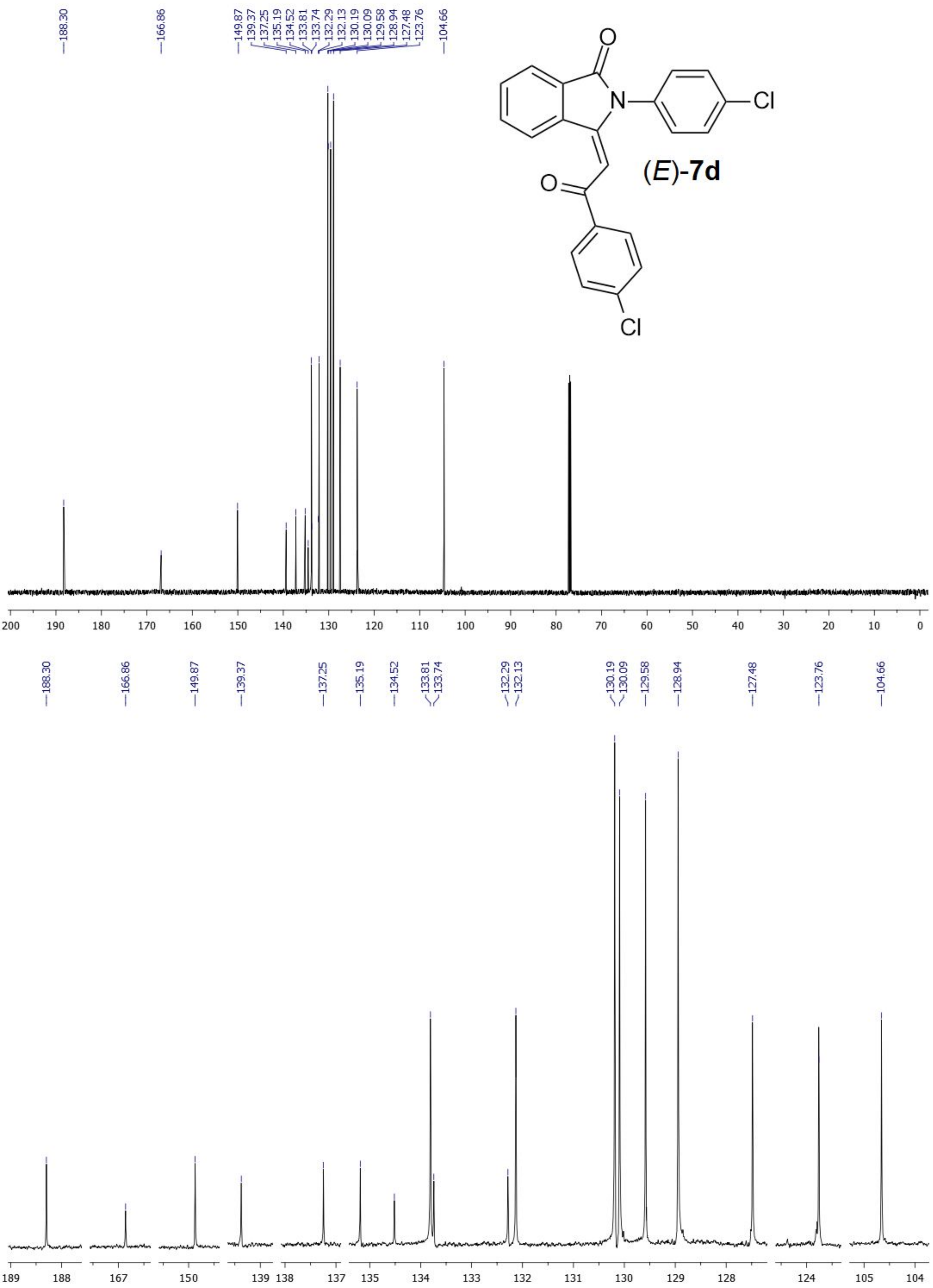

Figure S53. ^{13}C-NMR spectrum (150 MHz, $CDCl_3$) of (*E*)-2-(4-chlorophenyl)-3-(2-(4-chlorophenyl)-2-oxoethylidene)isoindolin-1-one ((*E*)-**7d**): full scale spectrum (top) and spectrum expansions (bottom).

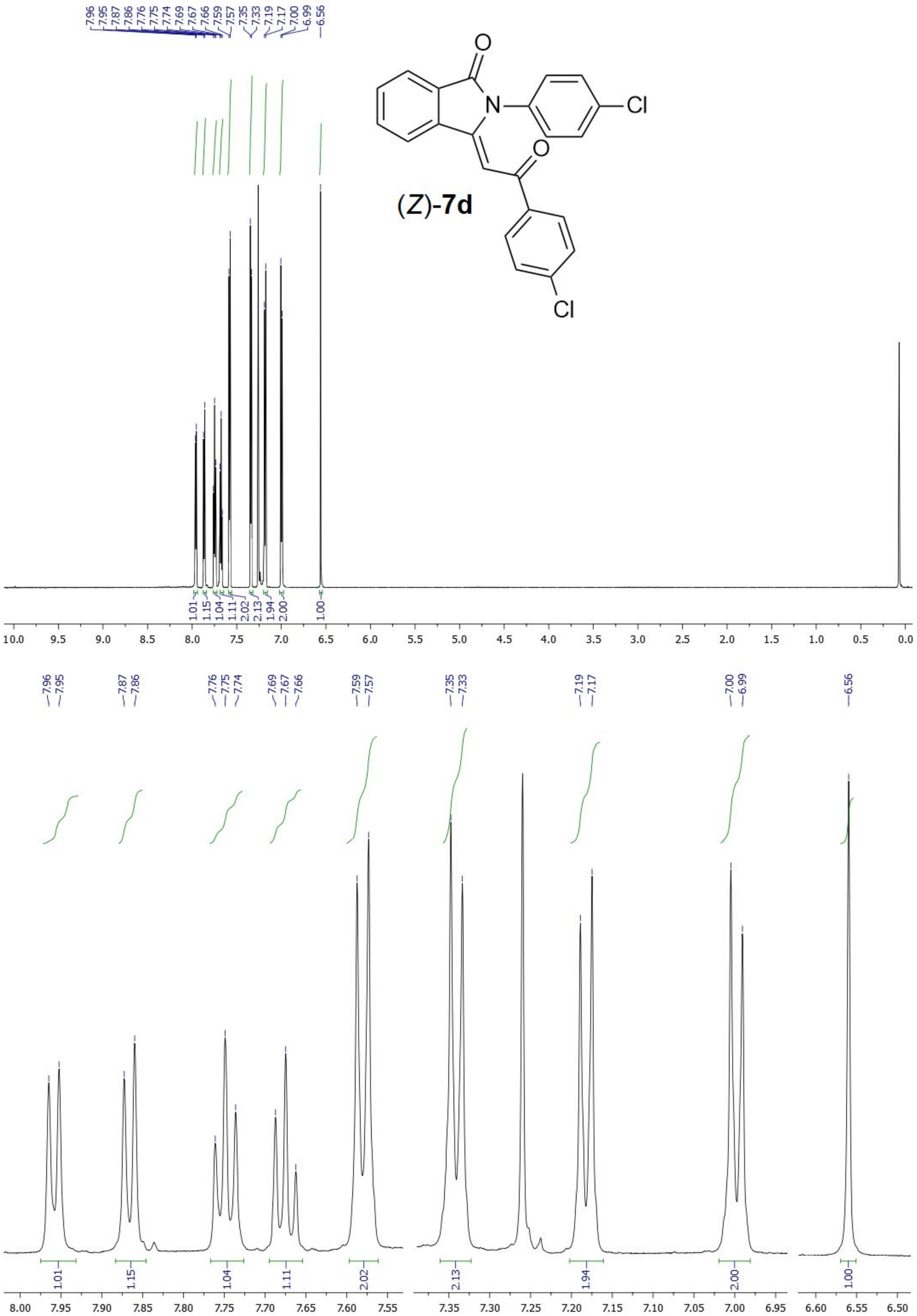

Figure S54. ^{1}H-NMR spectrum (600 MHz, $CDCl_3$) of (*Z*)-2-(4-chlorophenyl)-3-(2-(4-chlorophenyl)-2-oxoethylidene)isoindolin-1-one ((*Z*)-**7d**): full scale spectrum (top) and spectrum expansions (bottom).

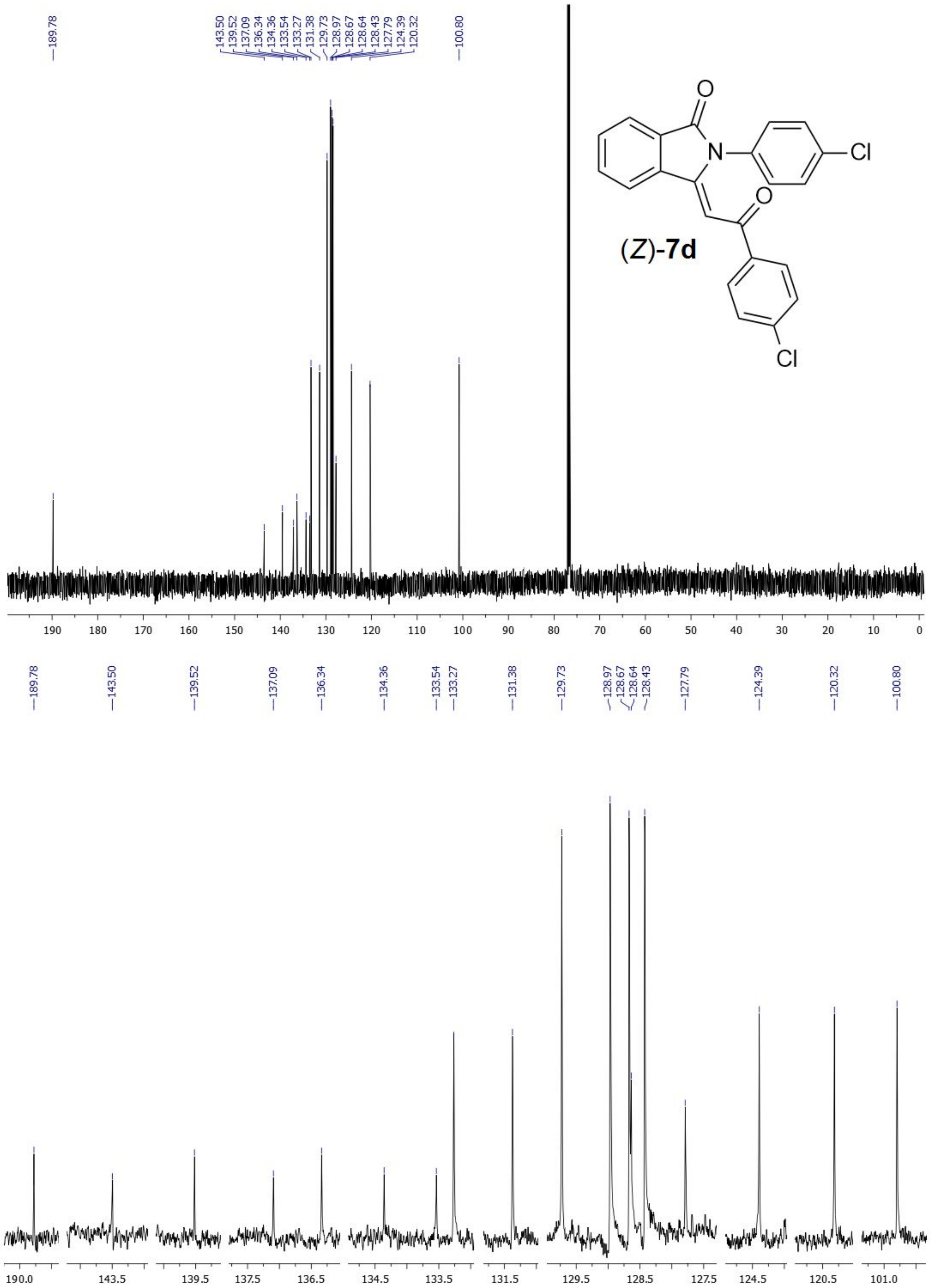

Figure S55. ^{13}C-NMR spectrum (150 MHz, $CDCl_3$) of (*Z*)-2-(4-chlorophenyl)-3-(2-(4-chlorophenyl)-2-oxoethylidene)isoindolin-1-one ((*Z*)-**7d**): full scale spectrum (top) and spectrum expansions (bottom).

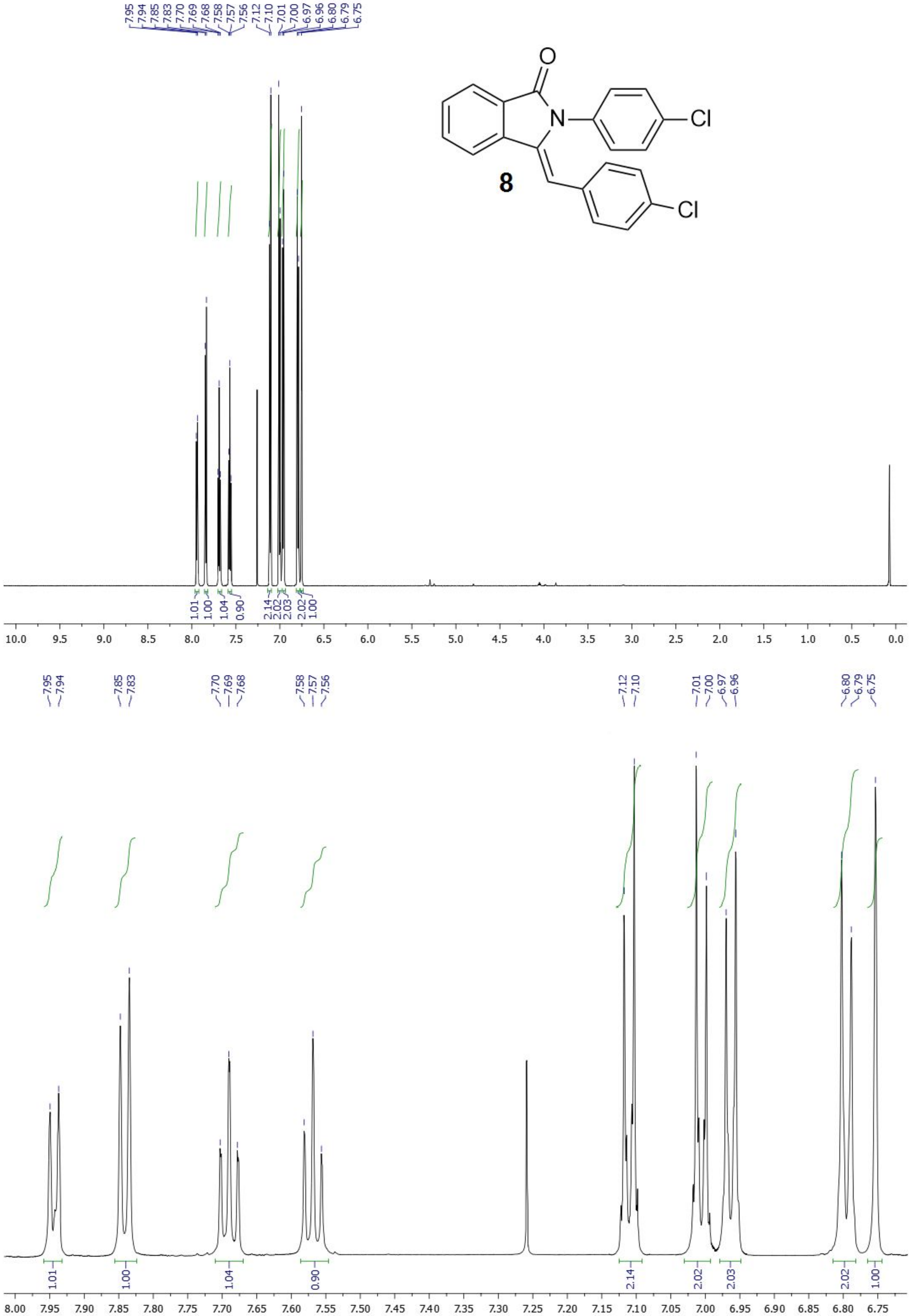

Figure S56. ^{1}H-NMR spectrum (600 MHz, $CDCl_3$) of (*Z*)-3-(4-chlorobenzylidene)-2-(4-chlorophenyl)isoindolin-1-one (**8**): full scale spectrum (top) and spectrum expansions (bottom).

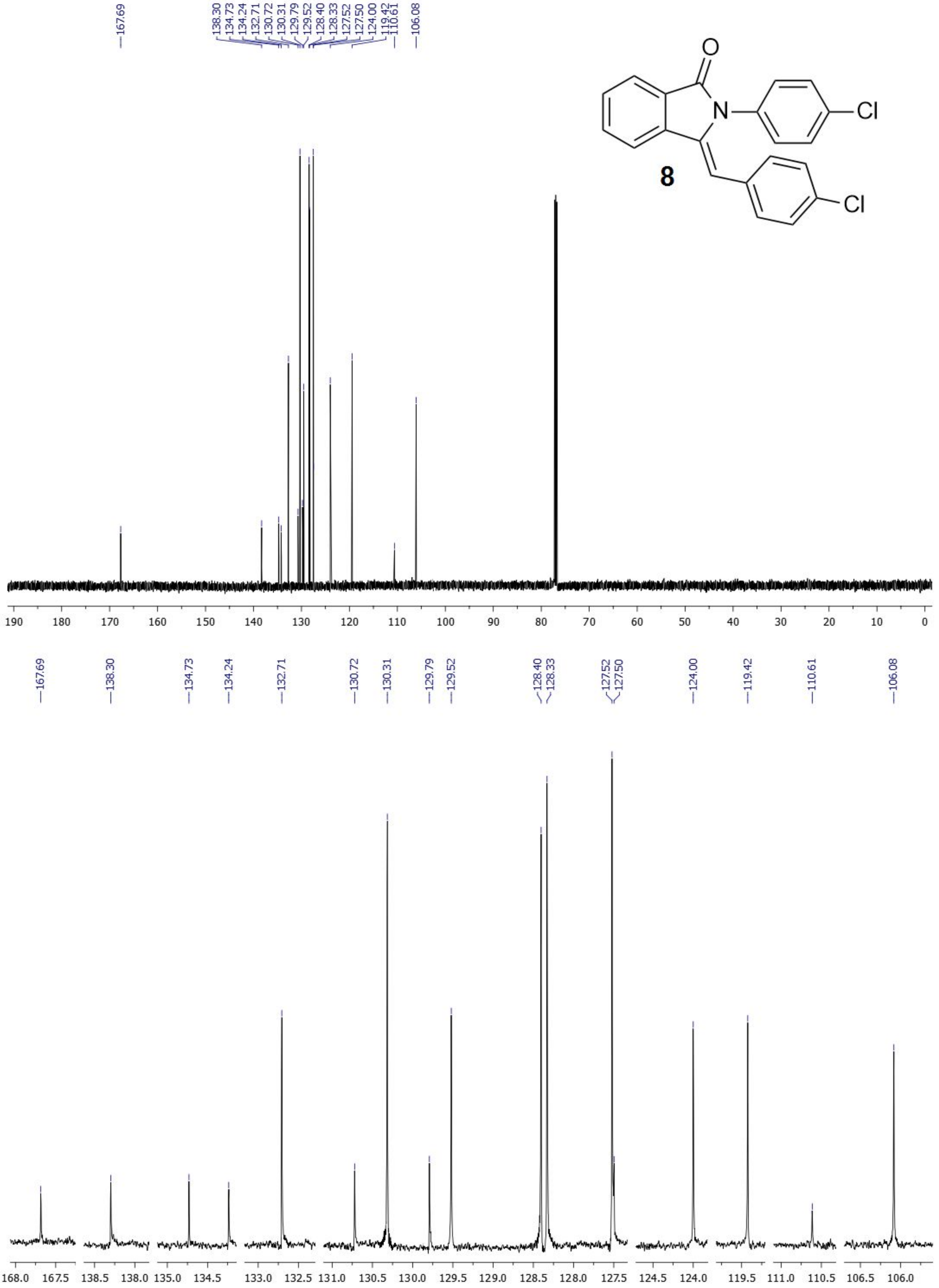

Figure S57. ^{13}C-NMR spectrum (150 MHz, $CDCl_3$) of (*Z*)-3-(4-chlorobenzylidene)-2-(4-chlorophenyl)isoindolin-1-one (**8**): full scale spectrum (top) and spectrum expansions (bottom).

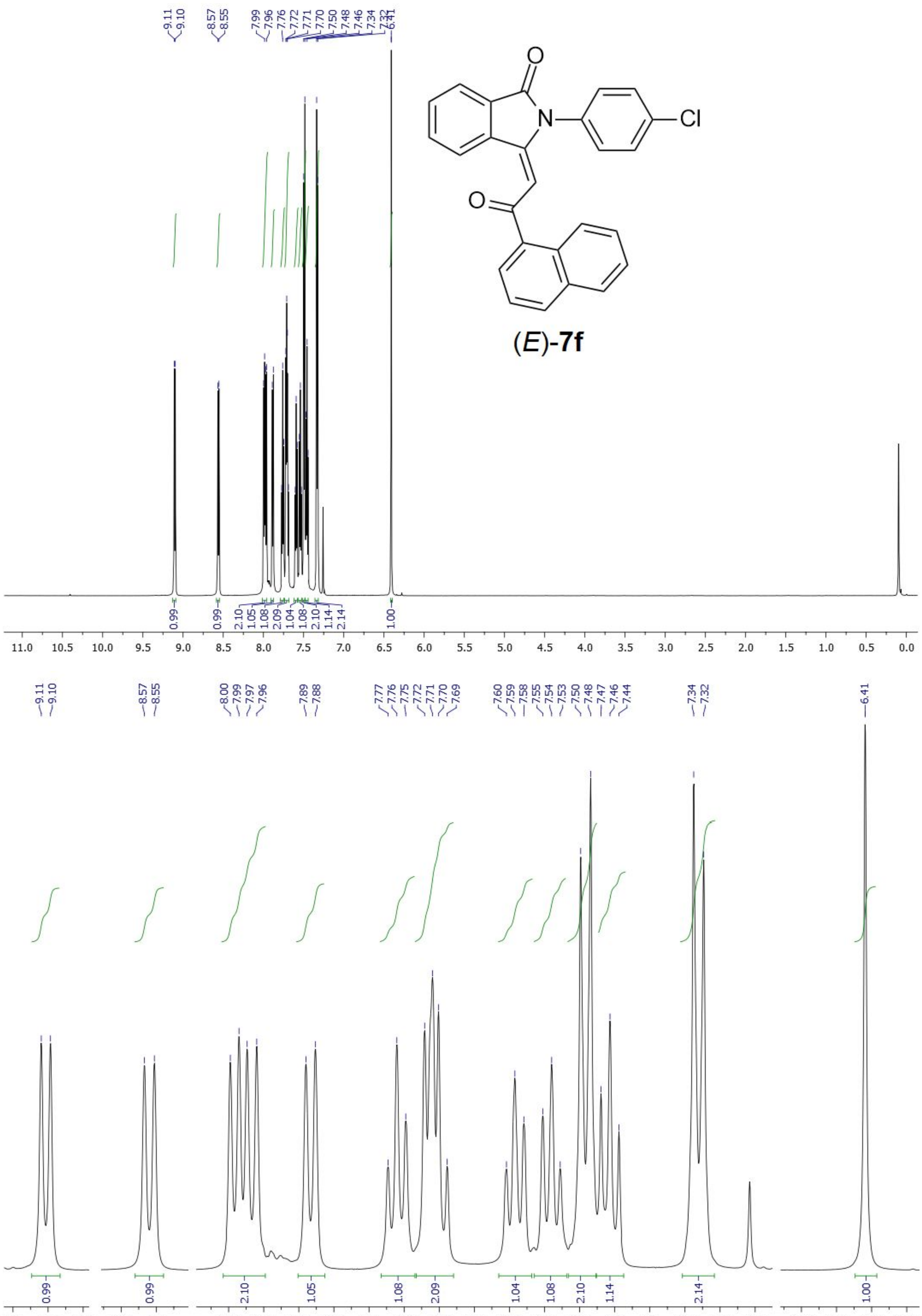

(*E*)-**7f**

Figure S58. ^{1}H-NMR spectrum (600 MHz, $CDCl_3$) of (*E*)-2-(4-chlorophenyl)-3-(2-(naphthalen-1-yl)-2-oxoethylidene)isoindolin-1-one ((*E*)-**7f**): full scale spectrum (top) and spectrum expansions (bottom).

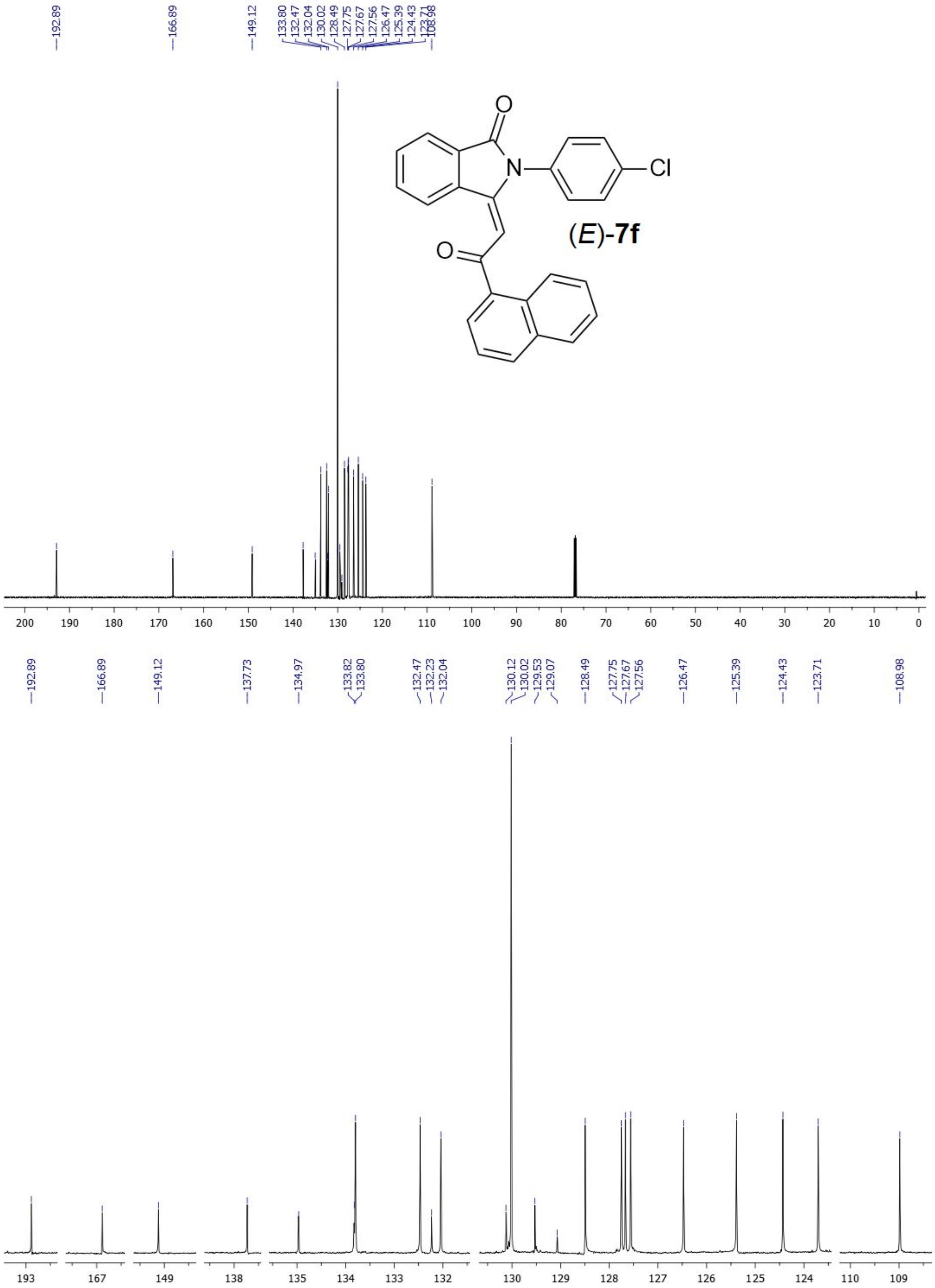

Figure S59. ^{13}C-NMR spectrum (150 MHz, $CDCl_3$) of (*E*)-2-(4-chlorophenyl)-3-(2-(naphthalen-1-yl)-2-oxoethylidene)isoindolin-1-one ((*E*)-**7f**): full scale spectrum (top) and spectrum expansions (bottom).

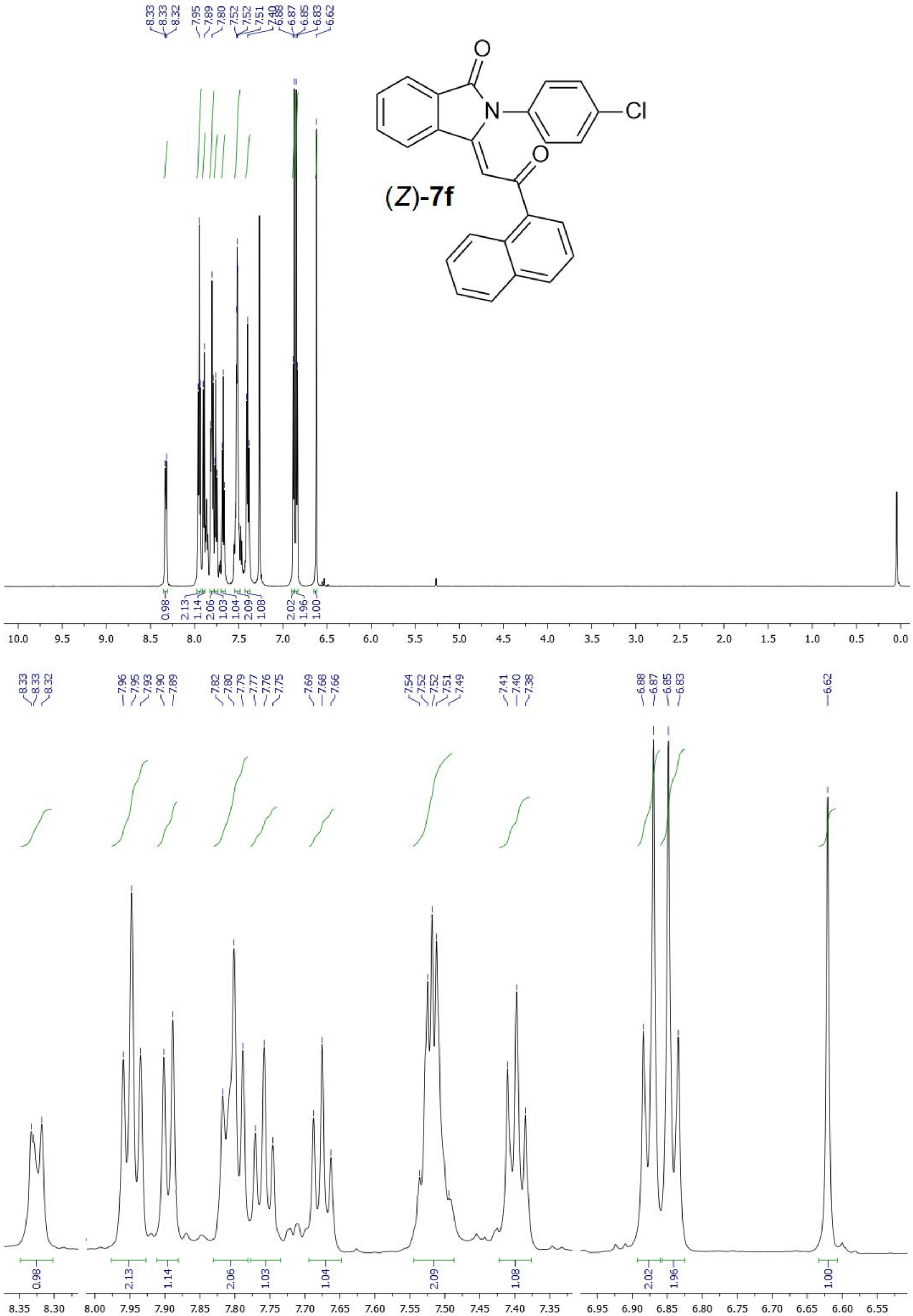

Figure S60. ^{1}H-NMR spectrum (600 MHz, $CDCl_3$) of (*Z*)-2-(4-chlorophenyl)-3-(2-(naphthalen-1-yl)-2-oxoethylidene)isoindolin-1-one ((*Z*)-**7f**): full scale spectrum (top) and spectrum expansions (bottom).

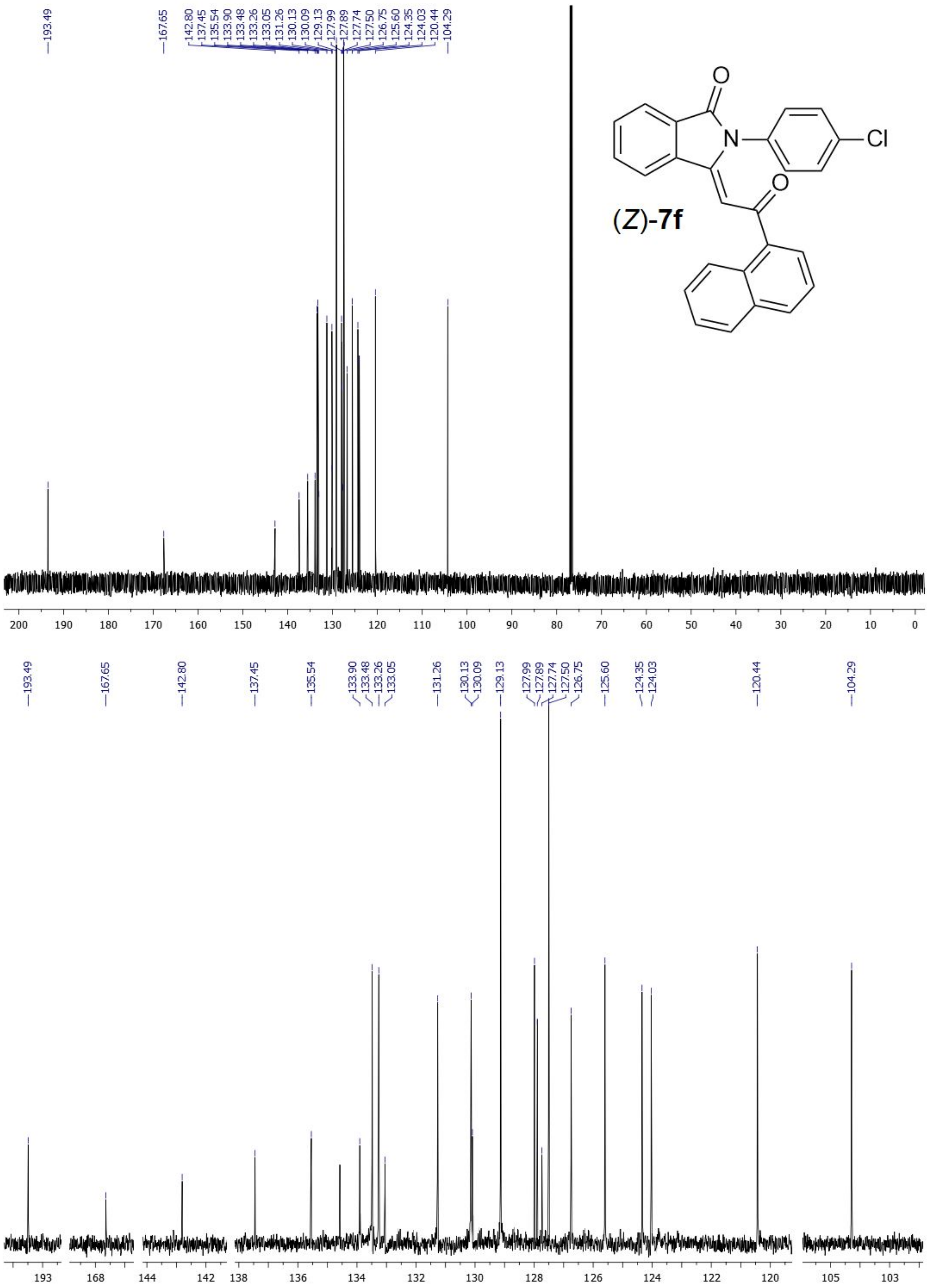

Figure S61. ^{13}C-NMR spectrum (150 MHz, $CDCl_3$) of (*Z*)-2-(4-chlorophenyl)-3-(2-(naphthalen-1-yl)-2-oxoethylidene)isoindolin-1-one ((*Z*)-**7f**): full scale spectrum (top) and spectrum expansions (bottom).

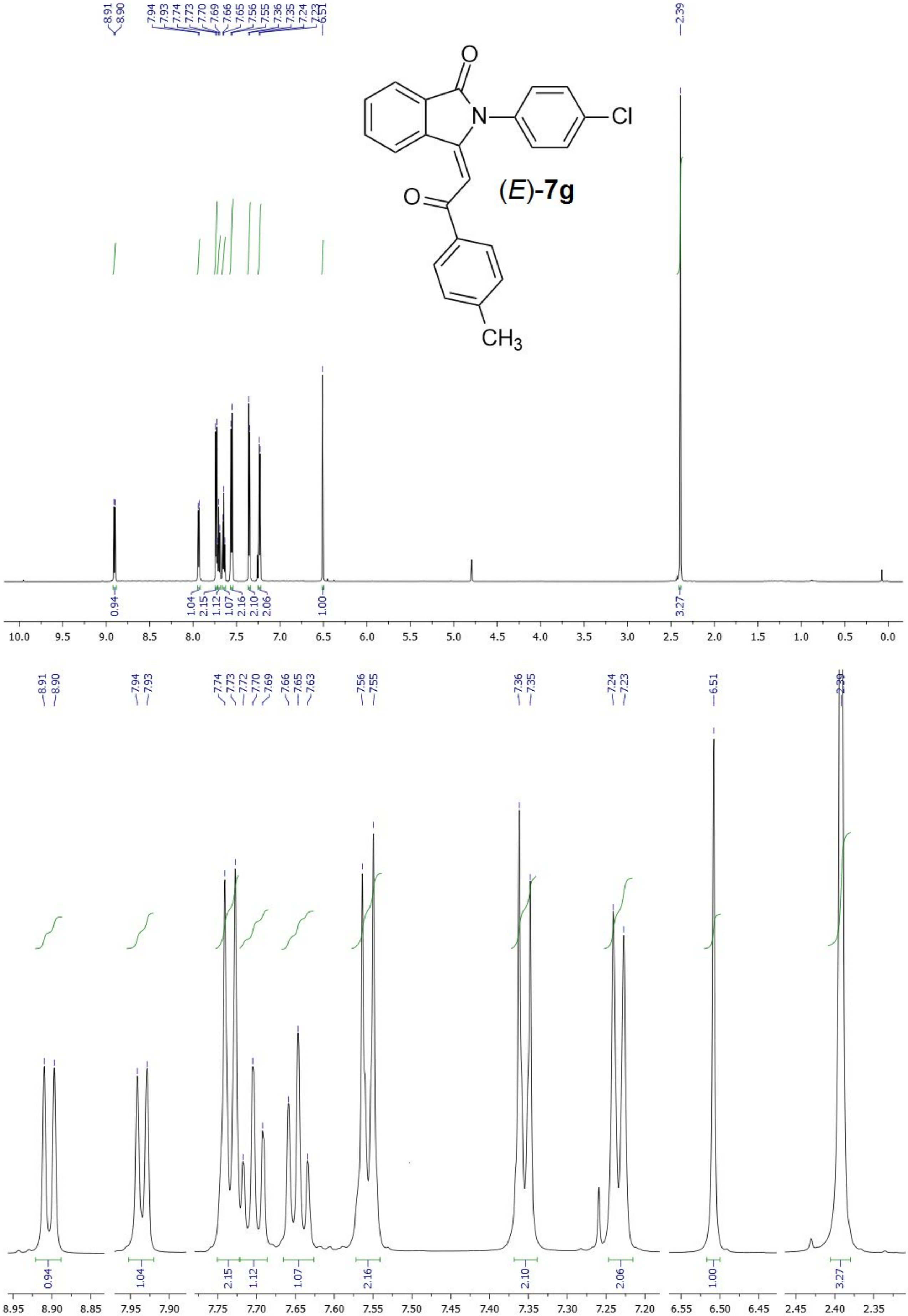

Figure S62. ^{1}H-NMR spectrum (600 MHz, $CDCl_3$) of (*E*)-2-(4-chlorophenyl)-3-(2-oxo-2-(*p*-tolyl)ethylidene)isoindolin-1-one ((*E*)-**7g**): full scale spectrum (top) and spectrum expansions (bottom).

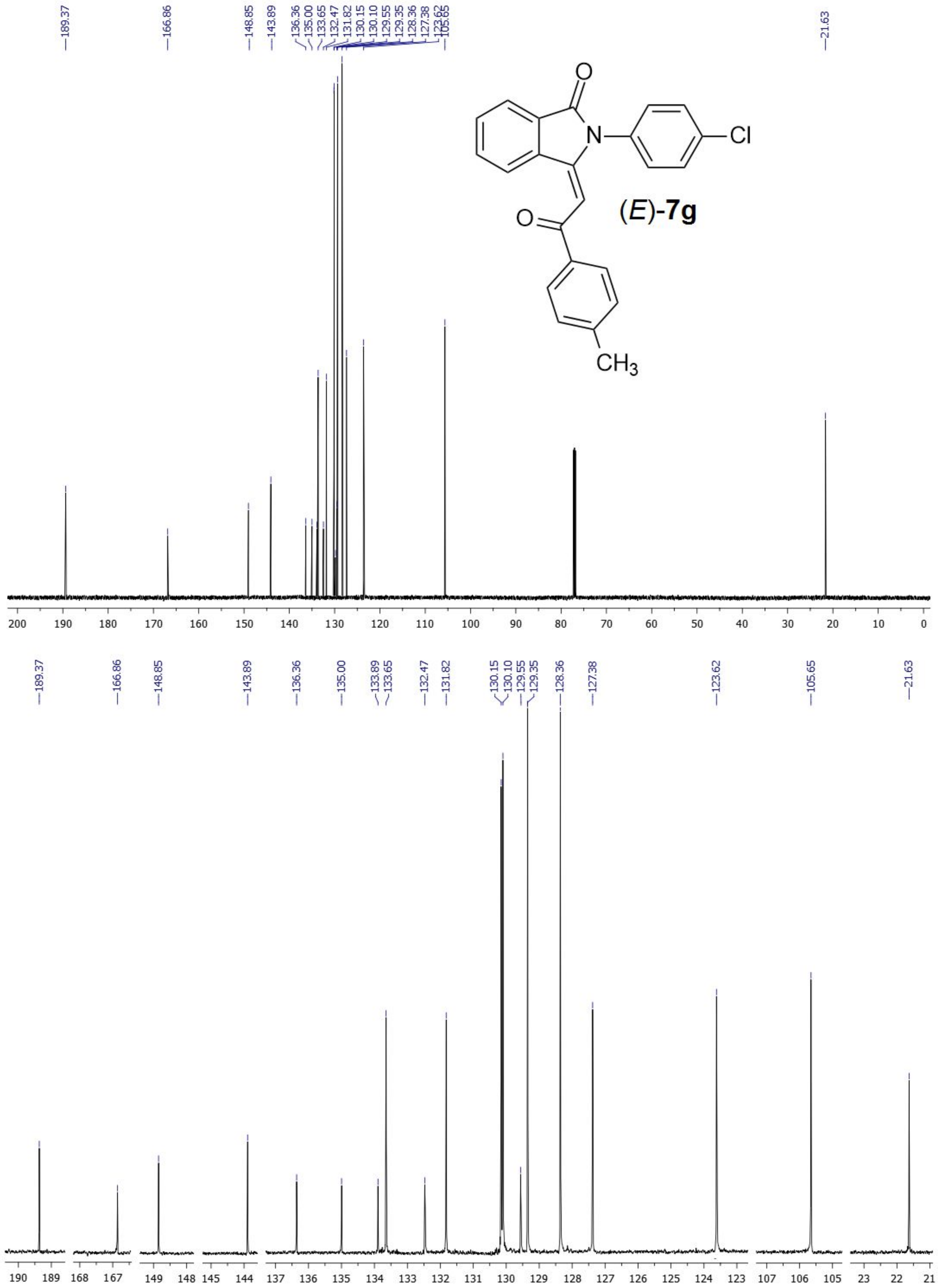

Figure S63. ^{13}C-NMR spectrum (150 MHz, $CDCl_3$) of (*E*)-2-(4-chlorophenyl)-3-(2-oxo-2-(*p*-tolyl)ethylidene)isoindolin-1-one ((*E*)-**7g**): full scale spectrum (top) and spectrum expansions (bottom).

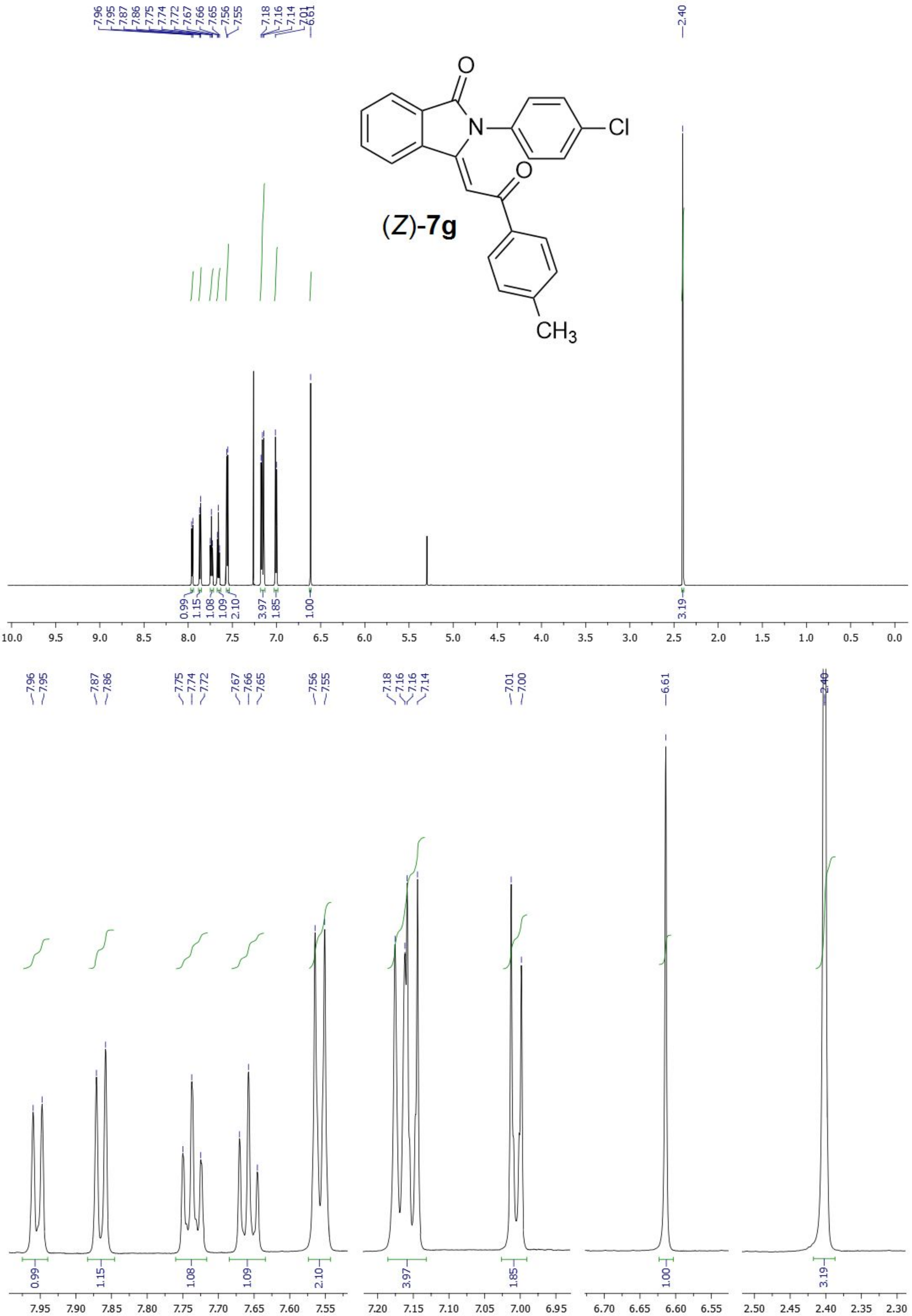

Figure S64. ^{1}H-NMR spectrum (600 MHz, $CDCl_3$) of (*Z*)-2-(4-chlorophenyl)-3-(2-oxo-2-(*p*-tolyl)ethylidene)isoindolin-1-one ((*Z*)-**7g**): full scale spectrum (top) and spectrum expansions (bottom).

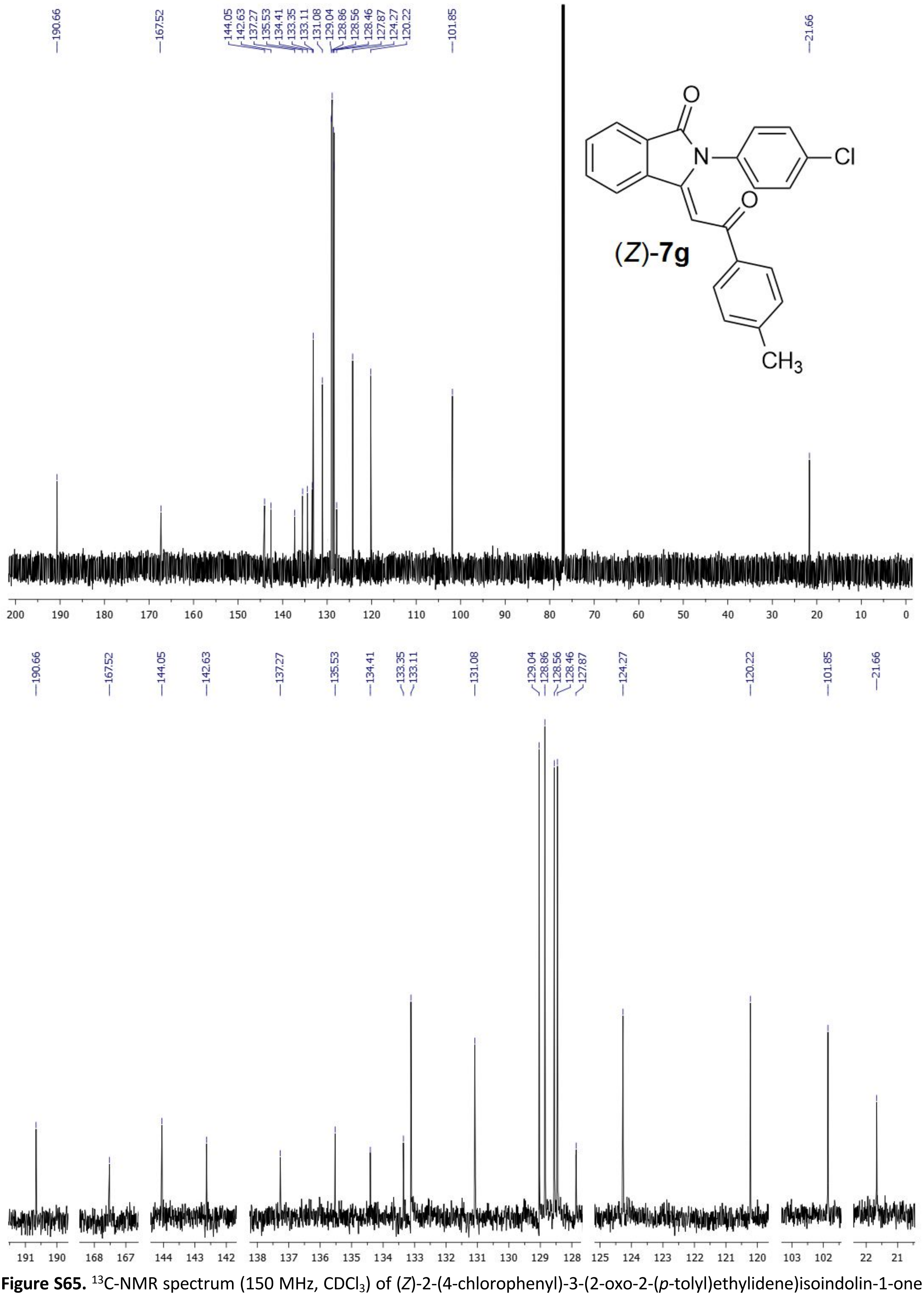

Figure S65. ^{13}C-NMR spectrum (150 MHz, $CDCl_3$) of (*Z*)-2-(4-chlorophenyl)-3-(2-oxo-2-(*p*-tolyl)ethylidene)isoindolin-1-one ((*Z*)-**7g**): full scale spectrum (top) and spectrum expansions (bottom).

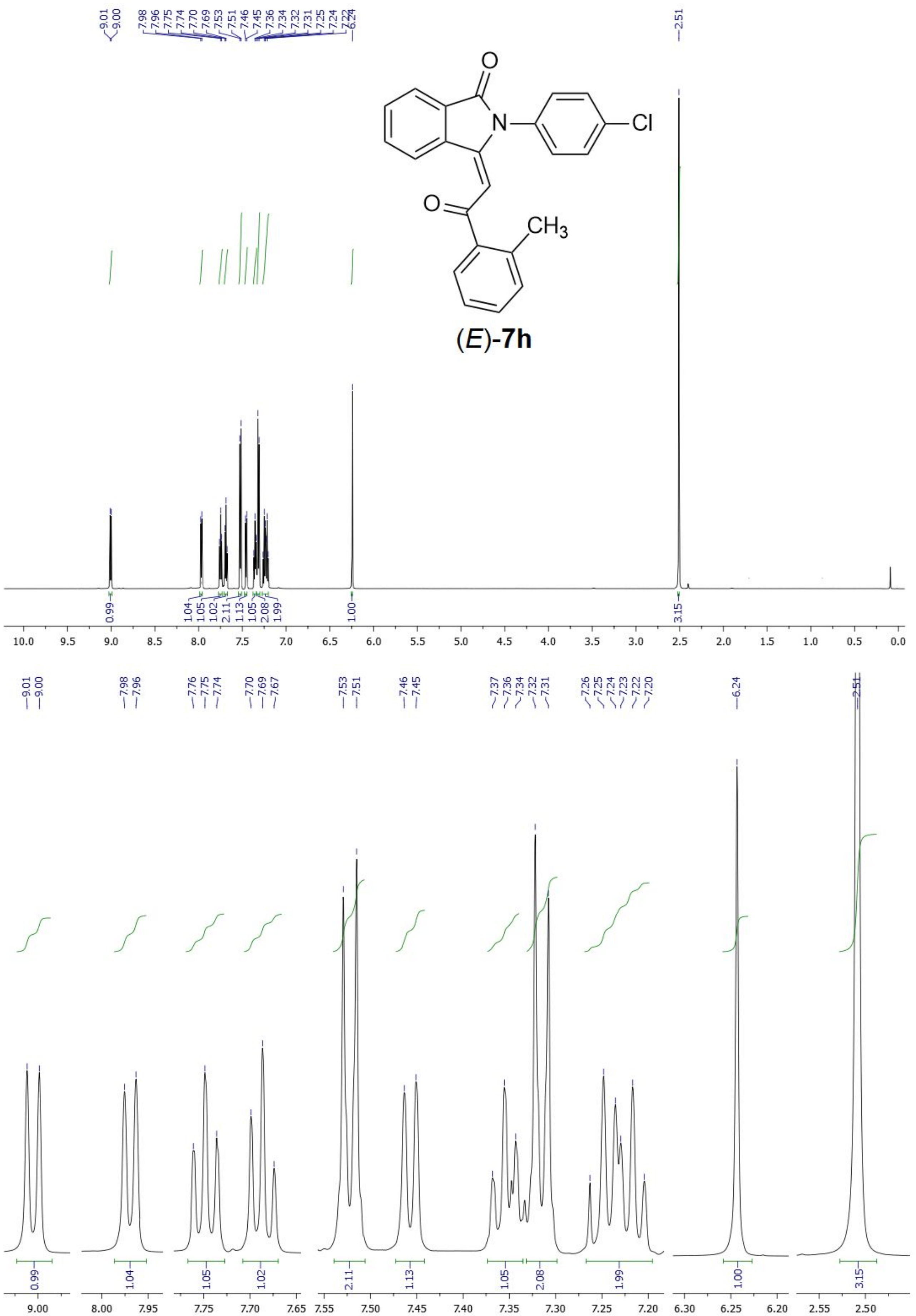

Figure S66. ^{1}H-NMR spectrum (600 MHz, $CDCl_3$) of (*E*)-2-(4-chlorophenyl)-3-(2-oxo-2-(*o*-tolyl)ethylidene)isoindolin-1-one ((*E*)-**7h**): full scale spectrum (top) and spectrum expansions (bottom).

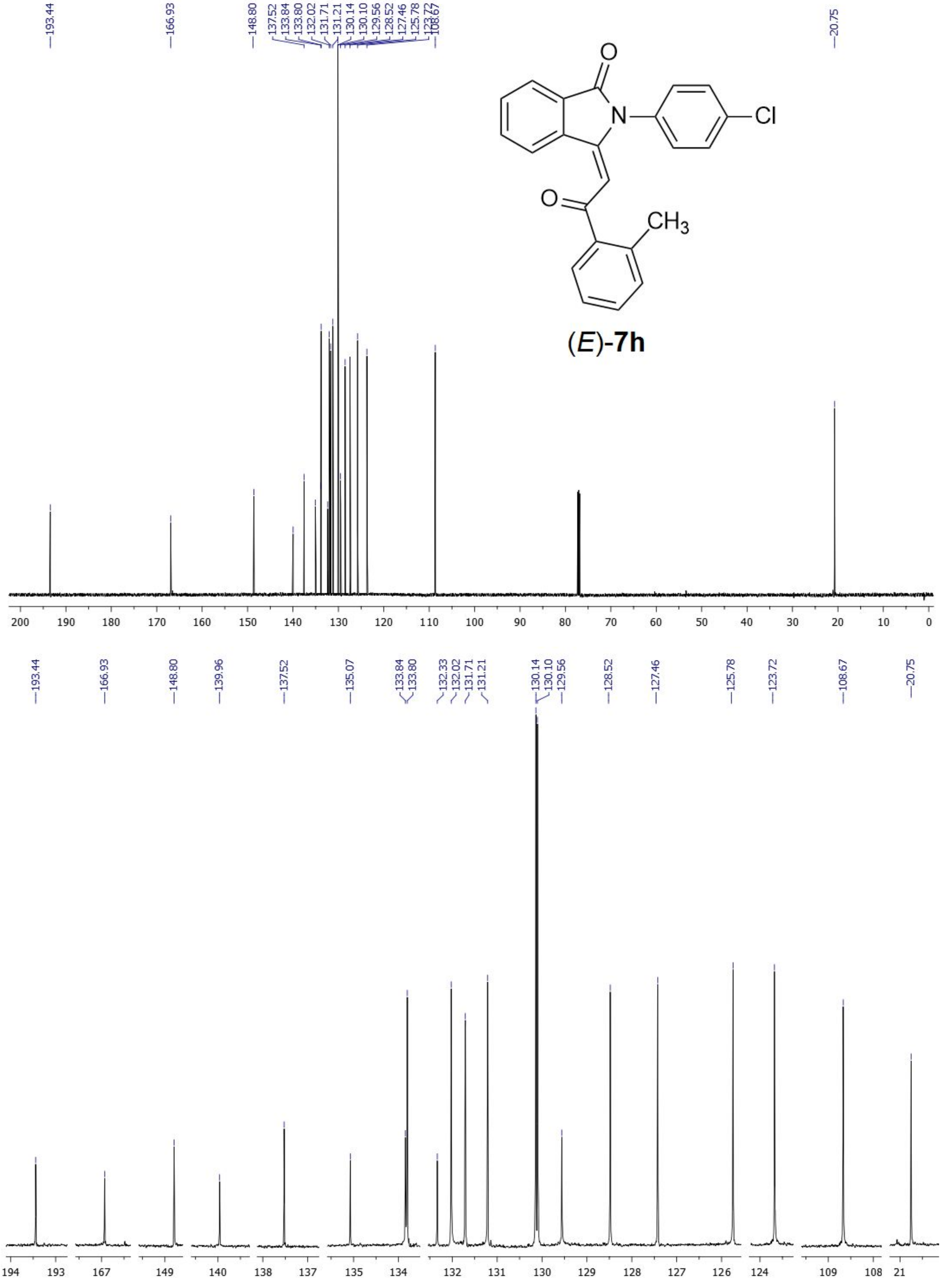

Figure S67. ^{13}C-NMR spectrum (150 MHz, $CDCl_3$) of (*E*)-2-(4-chlorophenyl)-3-(2-oxo-2-(*o*-tolyl)ethylidene)isoindolin-1-one ((*E*)-**7h**): full scale spectrum (top) and spectrum expansions (bottom).

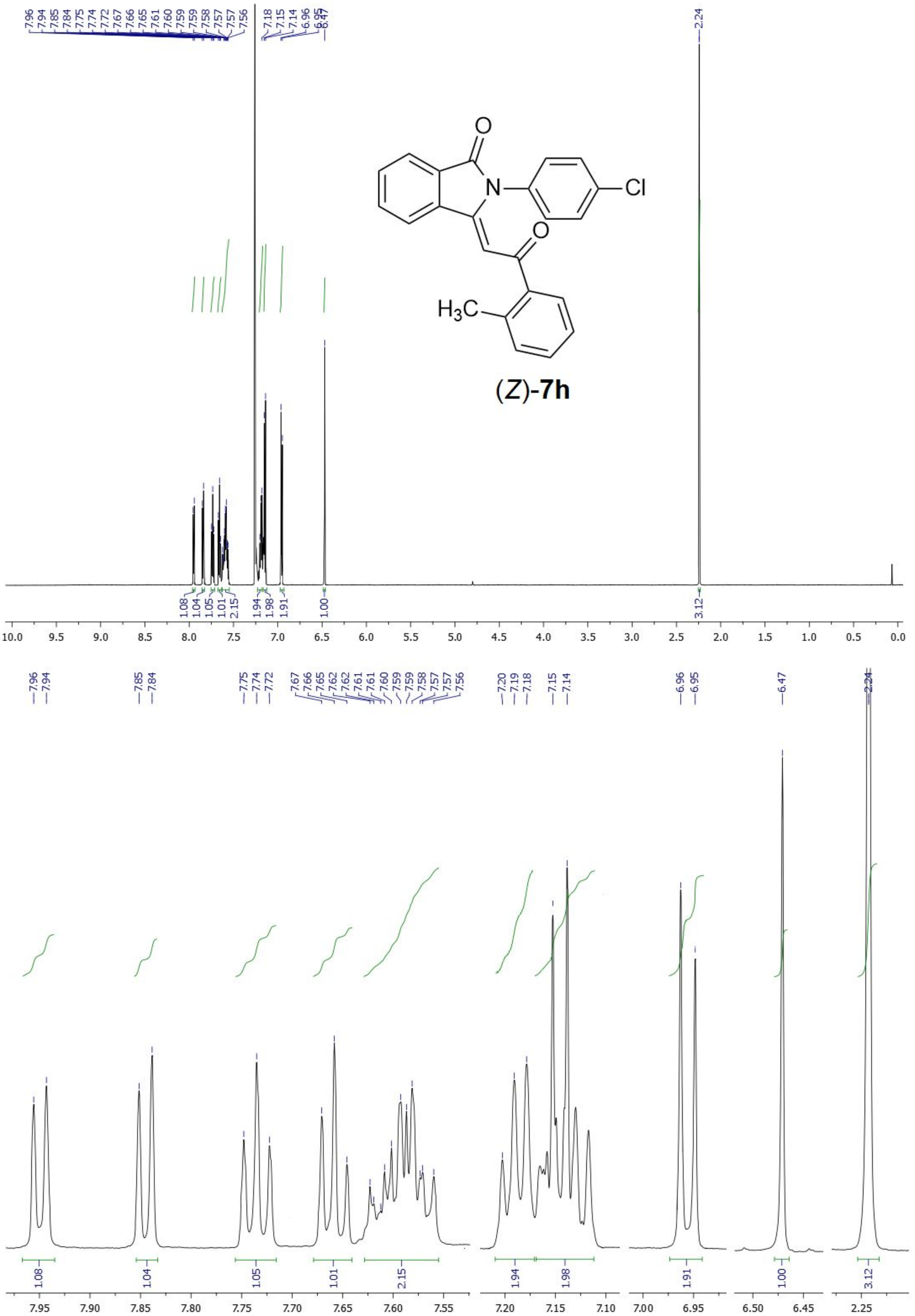

Figure S68. ^{1}H-NMR spectrum (600 MHz, $CDCl_3$) of (*Z*)-2-(4-chlorophenyl)-3-(2-oxo-2-(*o*-tolyl)ethylidene)isoindolin-1-one ((*Z*)-**7h**): full scale spectrum (top) and spectrum expansions (bottom).

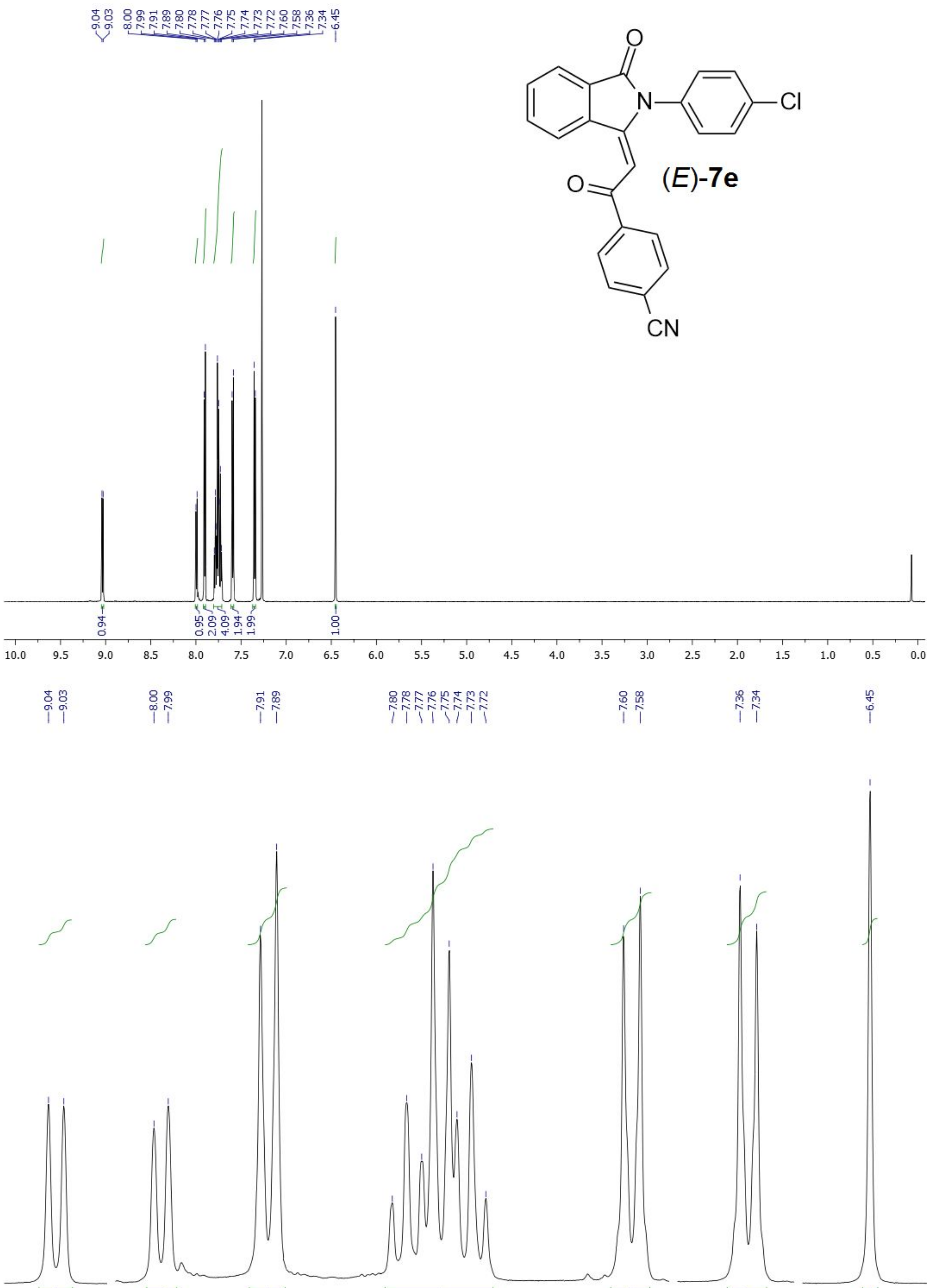

Figure S69. ^{1}H-NMR spectrum (600 MHz, $CDCl_3$) of (*E*)-4-(2-(2-(4-chlorophenyl)-3-oxoisoindolin-1-ylidene)acetyl)benzonitrile ((*E*)-**7e**): full scale spectrum (top) and spectrum expansions (bottom).

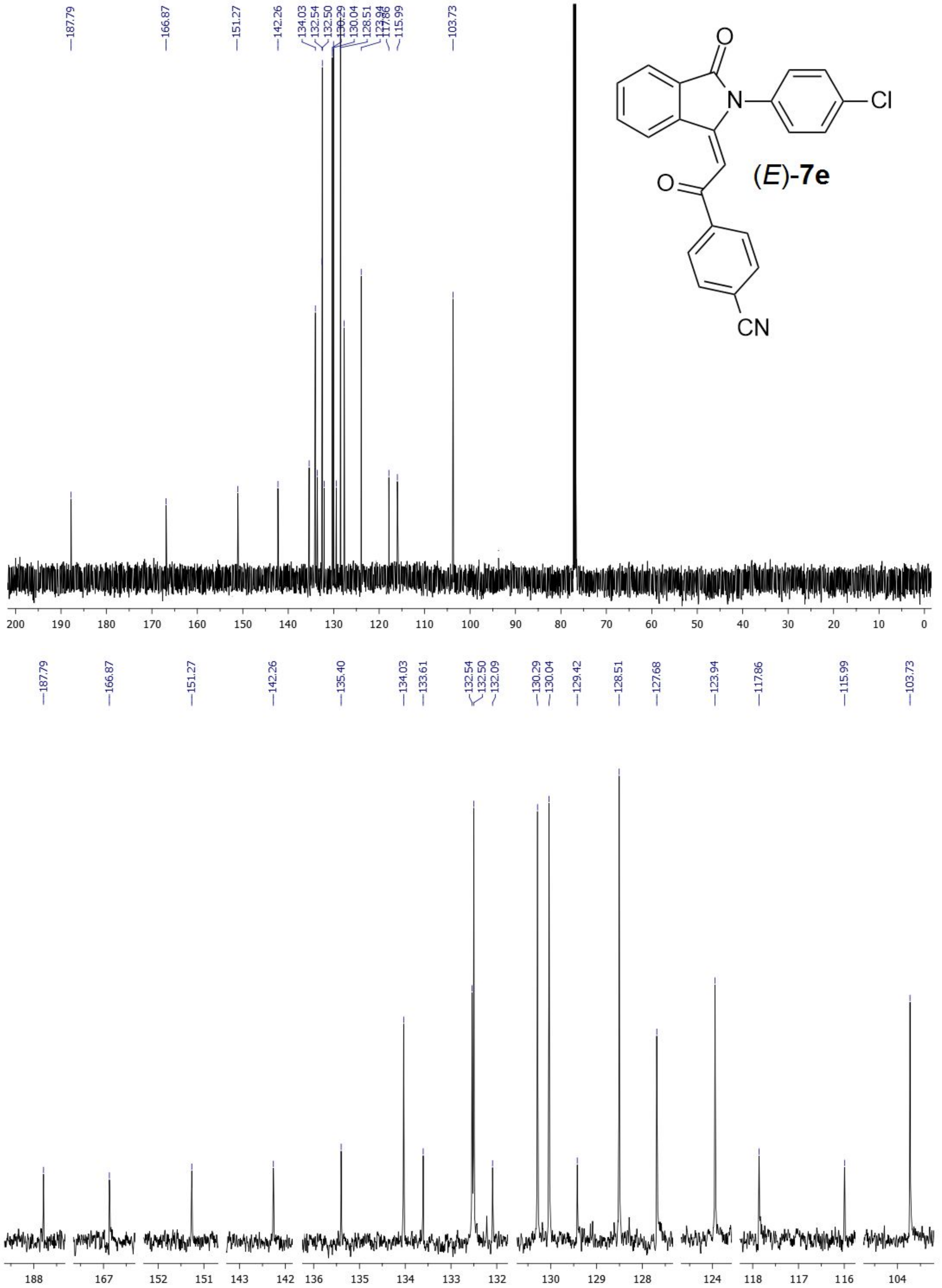

Figure S70. ^{13}C-NMR spectrum (150 MHz, $CDCl_3$) of (*E*)-4-(2-(2-(4-chlorophenyl)-3-oxoisoindolin-1-ylidene)acetyl)benzonitrile ((*E*)-**7e**): full scale spectrum (top) and spectrum expansions (bottom).

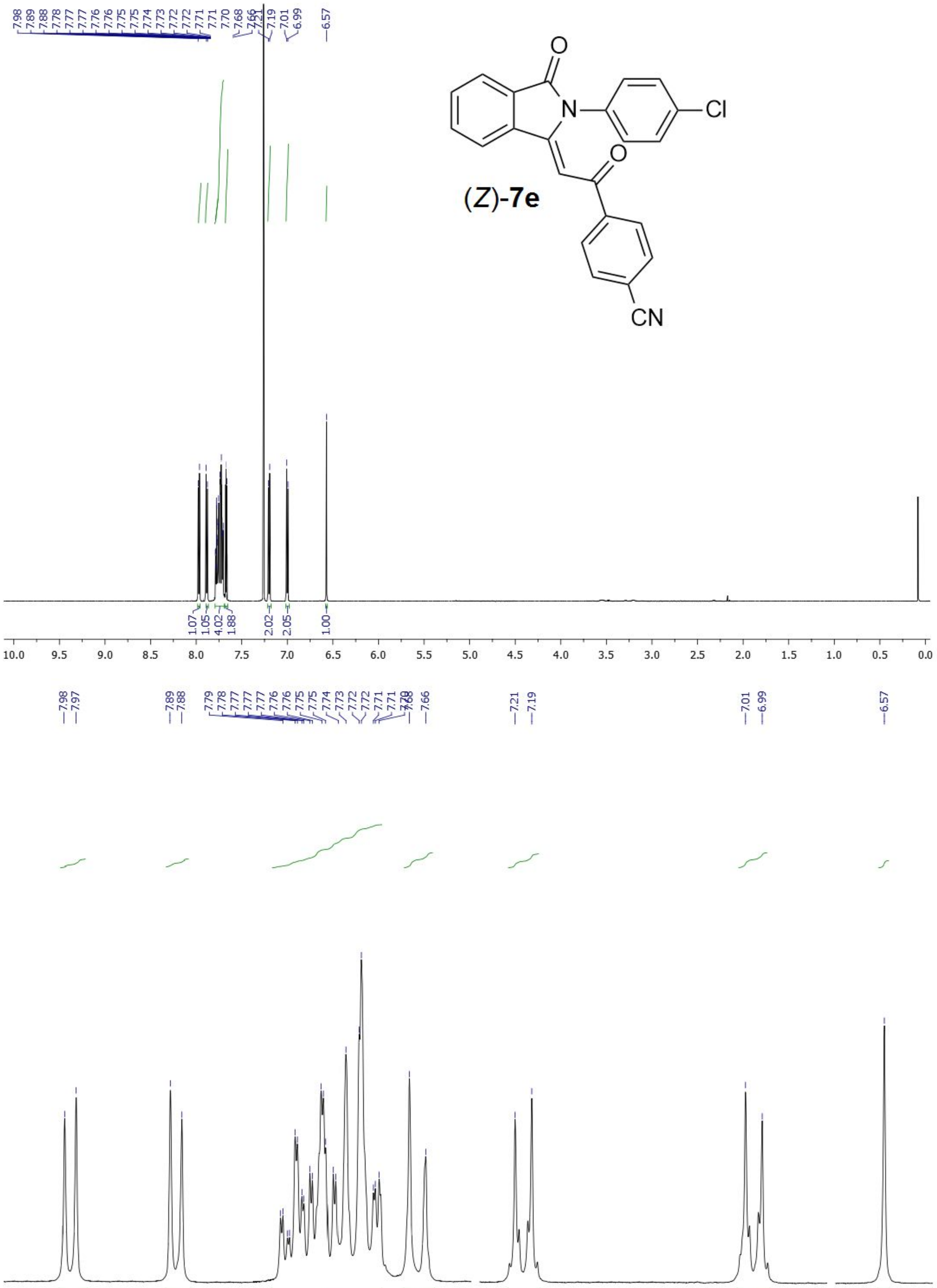

Figure S71. ^{1}H-NMR spectrum (600 MHz, $CDCl_3$) of (*Z*)-4-(2-(2-(4-chlorophenyl)-3-oxoisoindolin-1-ylidene)acetyl)benzonitrile ((*Z*)-**7e**): full scale spectrum (top) and spectrum expansions (bottom).

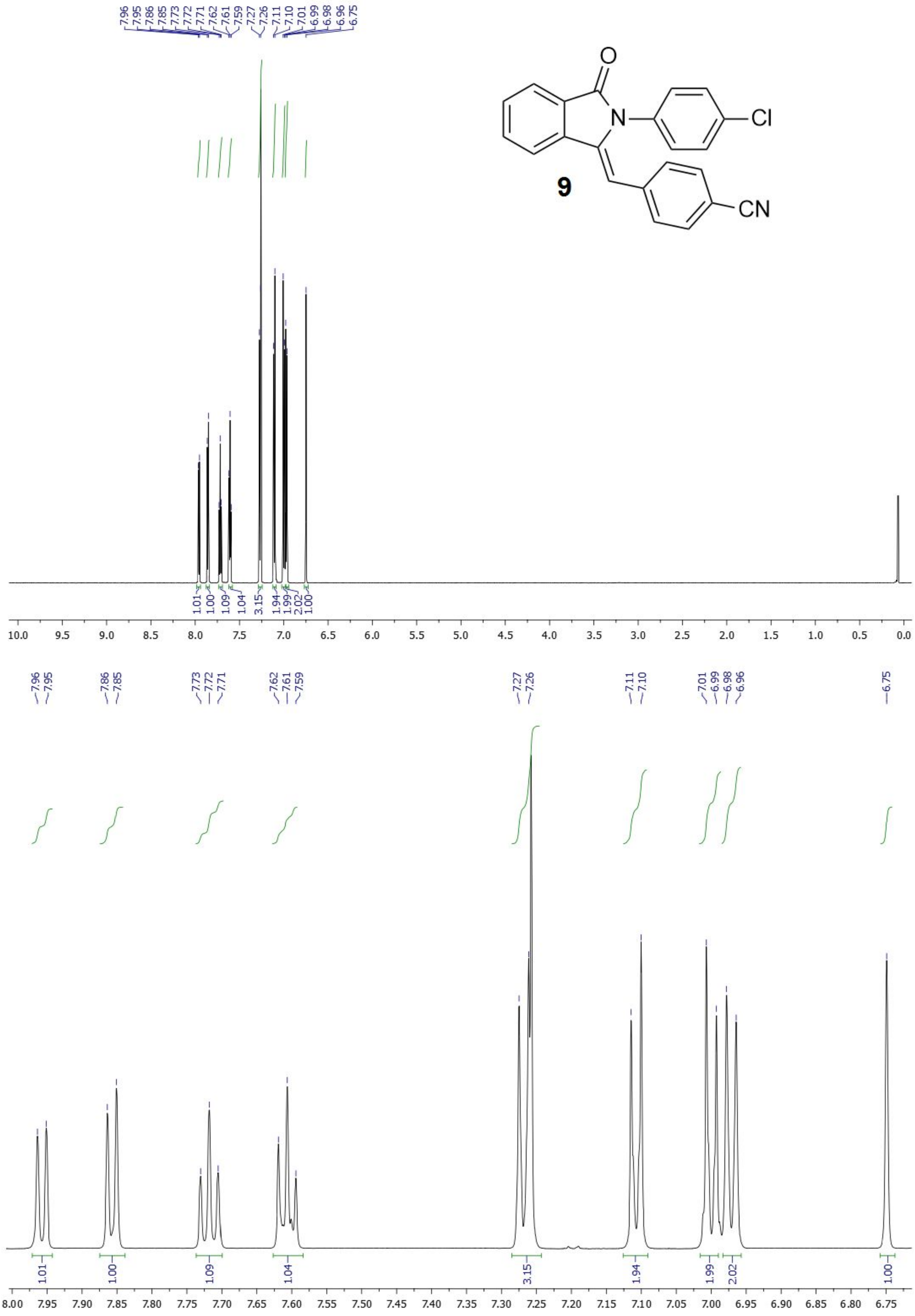

Figure S72. ^{1}H-NMR spectrum (600 MHz, $CDCl_3$) of (*Z*)-4-((2-(4-chlorophenyl)-3-oxoisoindolin-1-ylidene)methyl)benzonitrile (**9**): full scale spectrum (top) and spectrum expansions (bottom).

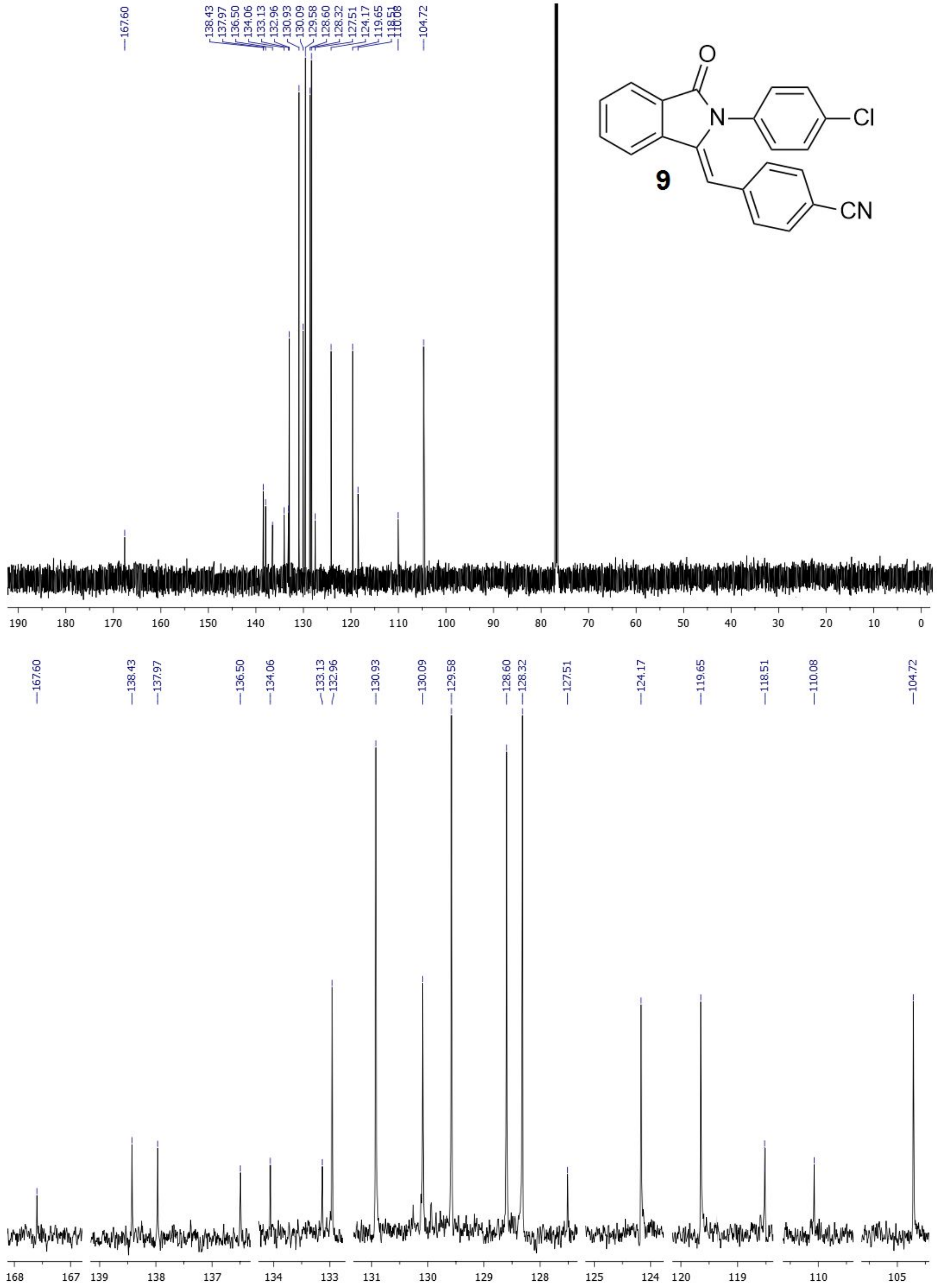

Figure S73. ^{13}C-NMR spectrum (150 MHz, $CDCl_3$) of (*Z*)-4-((2-(4-chlorophenyl)-3-oxoisoindolin-1-ylidene)methyl)benzonitrile (**9**): full scale spectrum (top) and spectrum expansions (bottom).

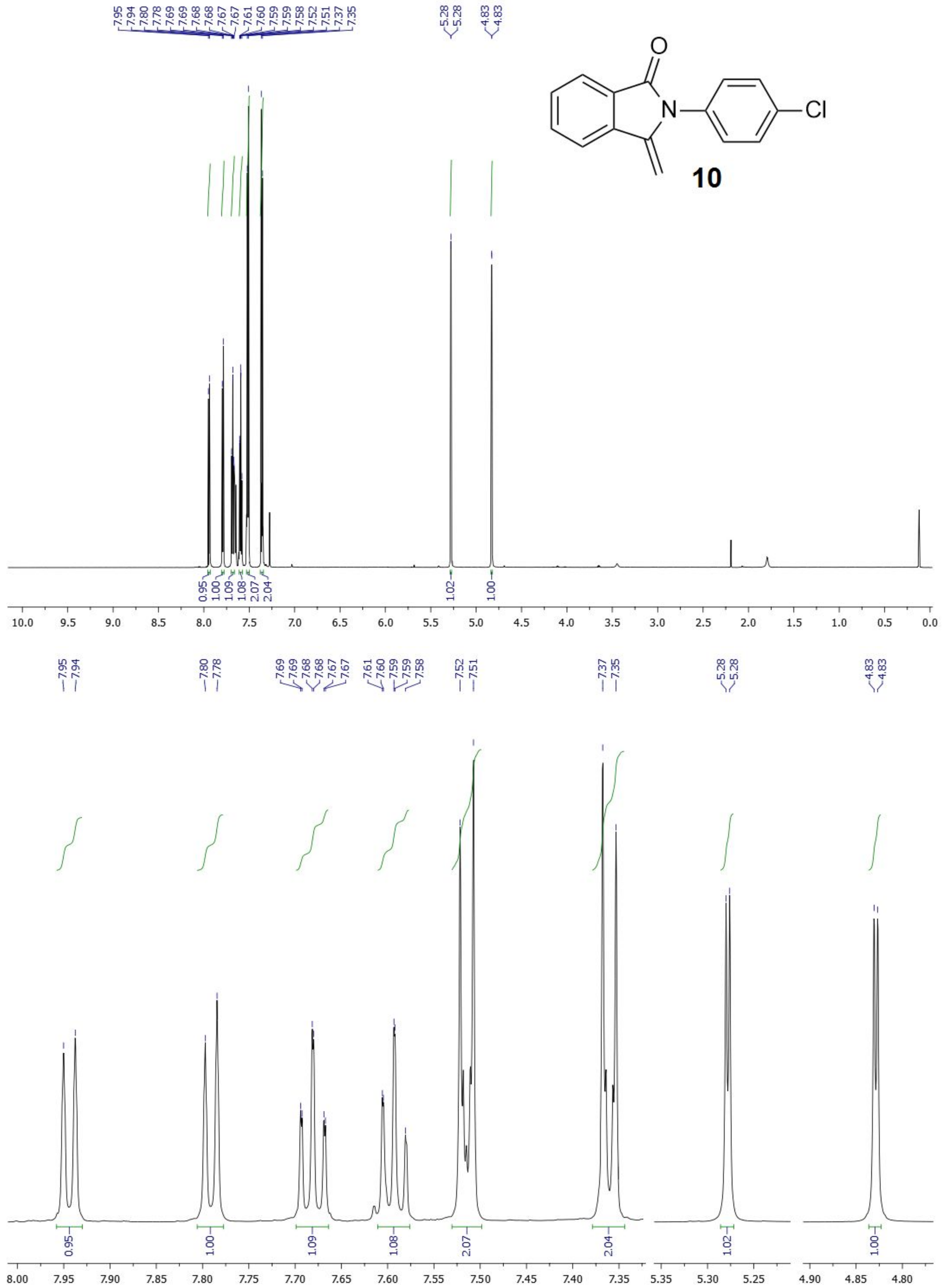

Figure S74. ^{1}H-NMR spectrum (600 MHz, $CDCl_3$) of 2-(4-chlorophenyl)-3-methyleneisoindolin-1-one (**10**): full scale spectrum (top) and spectrum expansions (bottom).

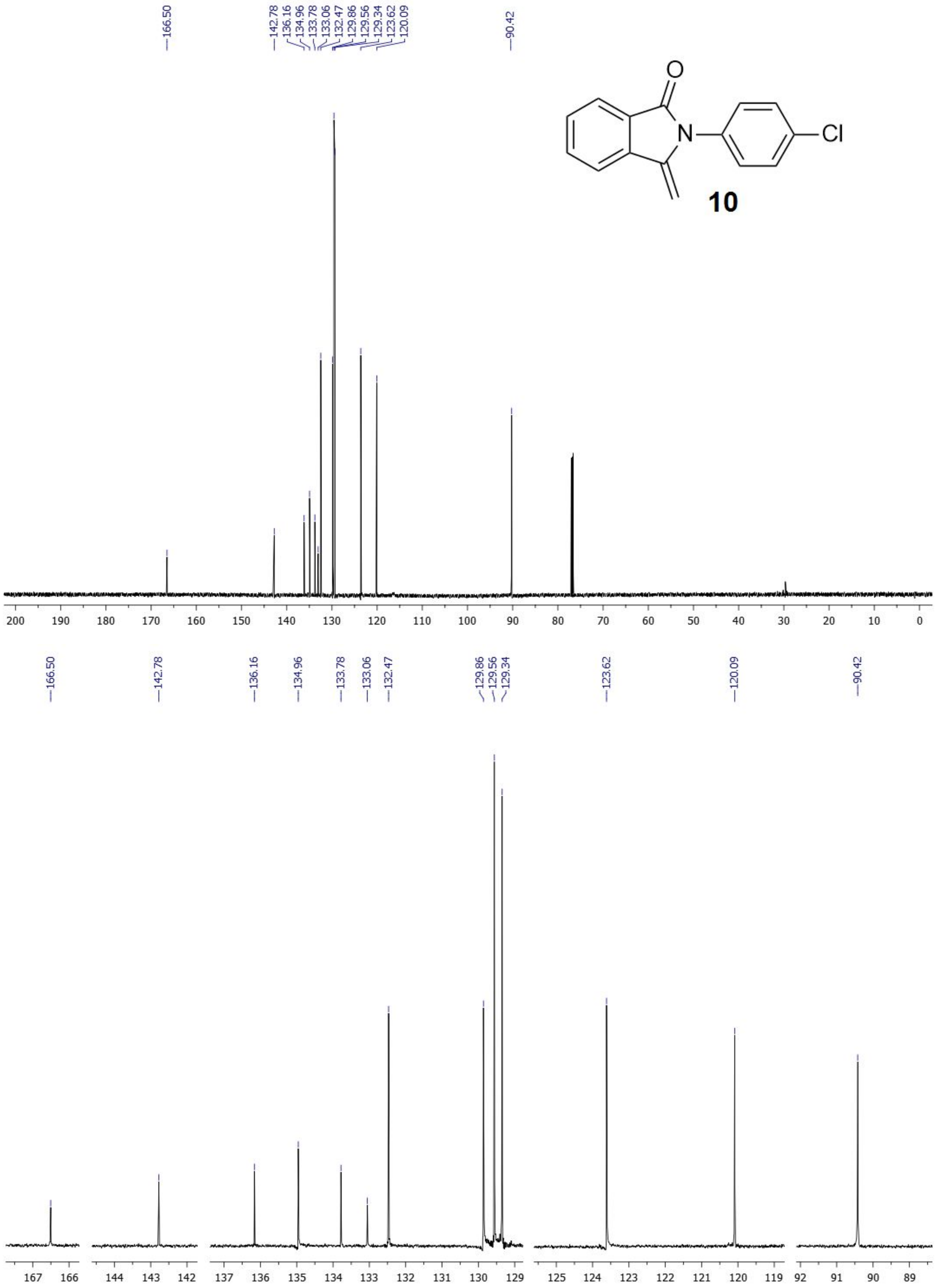

Figure S75. ^{13}C-NMR spectrum (150 MHz, $CDCl_3$) of 2-(4-chlorophenyl)-3-methyleneisoindolin-1-one (**10**): full scale spectrum (top) and spectrum expansions (bottom).
